# Automated Assembly of Polyglucuronic Acids for Structural Explorations

**DOI:** 10.1021/jacs.5c21268

**Published:** 2026-02-11

**Authors:** Sandhya N. Mardhekar, Dominik Weh, Martina Delbianco, Peter H. Seeberger

**Affiliations:** ⊥ Department of Biomolecular Systems, 28321 Max-Planck-Institute of Colloids and Interfaces, Am Mühlenberg 1, 14476 Potsdam, Germany; § Institute of Chemistry and Biochemistry, Freie Universität Berlin, Arnimallee 22, 14195 Berlin, Germany

## Abstract

Polyuronic acids are important biopolymers in marine organisms, where they contribute to extracellular matrix modulation, cell signaling, and carbon cycling. However, the intrinsic structural heterogeneity of polyuronic acids has hindered efforts to establish clear structure–function relationships. Here, we report an automated glycan assembly (AGA) approach that enables the precise synthesis of β-(1–4)-linked d-glucuronic acid (GlcA) oligomers with defined chain lengths and glycosidic linkage stereochemistry. Molecular dynamics simulations revealed a characteristic 2-fold helical conformation, with rigidity in short oligomers and enhanced flexibility emerging in longer sequences. The calcium binding behavior of these oligomers was explored by NMR titrations, revealing diffuse electrostatic binding rather than localized chelation. Polyglucuronic acid (PGA) oligomers are a well-defined molecular model for dissecting ion-mediated interactions and provide a framework for designing uronic acid-based glycomaterials with tunable properties.

## Graphical abstract

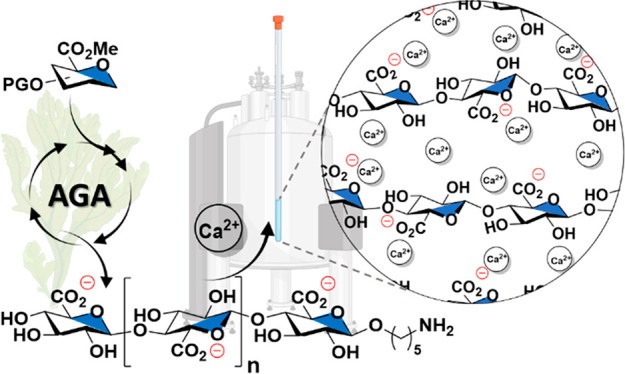

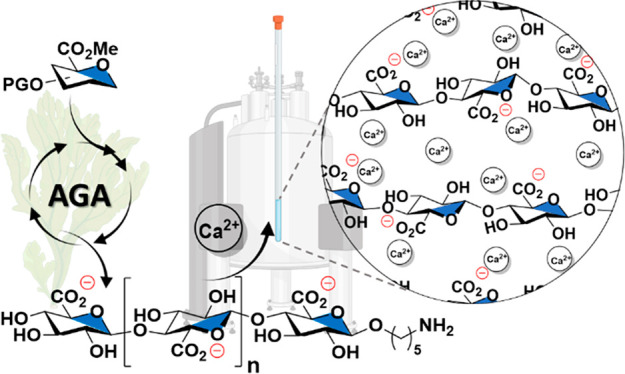

## Introduction

Uronic acids are key anionic components of natural polysaccharides with their carboxylate groups imparting ion binding, hydration, and molecular interactions that support extracellular scaffolding, molecular recognition, and carbon cycling.^1–3^ Uronic-acid-containing polysaccharides, such as alginic acid,^4,5^ pectin,^6^ and polyglucuronic acid (PGA),^7,8^ form a distinct family of polyanionic glycans. Their biomaterial properties are inherently linked to their residue composition, ionic charge density, glycosidic linkage, and conformational dynamics that govern interactions with ions, proteins, and hydrated matrices.^9–11^ This interplay of molecular structure and properties is exemplified by alginic acid from brown algae, composed of β-(1–4)-d-mannuronic acid (ManA) and α-l-guluronic acid (GulA) residues, whose tunable gelation and mechanical properties underpin applications in biomaterials^12–14^ and in the food industries.^15^ Similarly, in pectin, built from α-(1–4)-d-galacturonic acid (GalA) residues, the chemical structure governs mechanical integrity and hydration properties.^16,17^ Among these, PGA, a linear polymer composed of β-(1–4)-d-glucuronic acid (GlcA) residues, has been prepared chemically^18^ and/or extracted from green algae (*Ulva*),^19^ bacteria (*Sinorhizobium meliloti*),^20,21^ and fungi (*Mucor rouxii*)^7^ (Figure 1a). Its high carboxylate density and biodegradability make PGA a promising biomaterial for 3D bioprinting^22^ and tissue engineering applications.^23–25^

**Figure 1:**
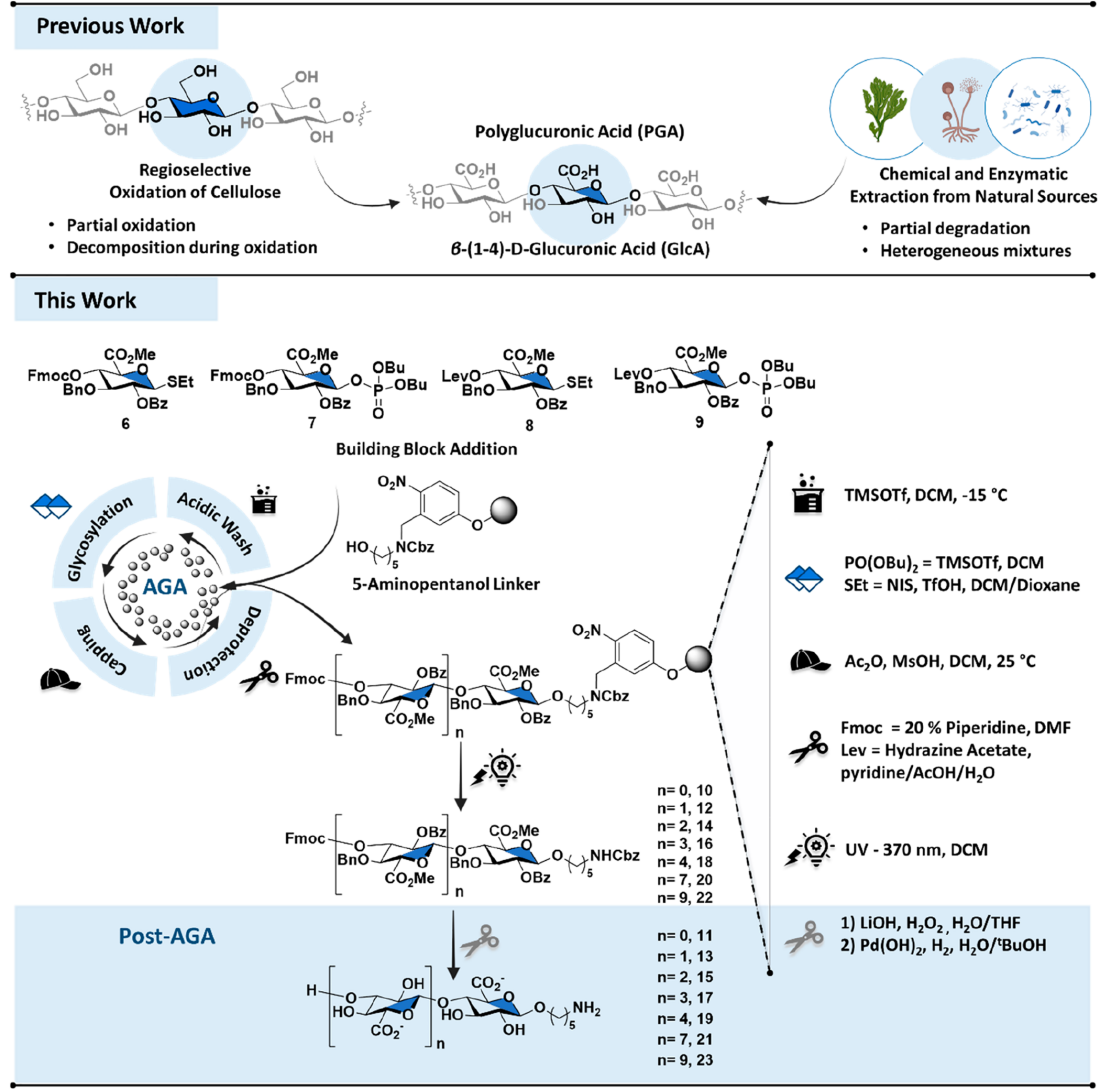
a) Methods to access polyglucuronic acid (PGA): Regioselective oxidation of cellulose and extraction routes (previous work). b) Automated glycan assembly (AGA) of protected oligomers using uronic acids for glycosylations, followed by post-AGA global deprotection to obtain well-defined polyglucuronic acid oligomers (this work).

A central feature of PGA and related polyuronides is the ability to bind divalent cations, particularly calcium.^26^ In alginic acid, this interaction is well understood, with calcium binding through an egg-box mechanism,^27^ which promotes gelation.^28,29^ However, insights into the calcium-binding behavior of PGA remain limited, relying largely on naturally extracted materials and computational models.^30^ The lack of access to pure, sequence-defined oligomers from natural sources is the key bottleneck hindering systematic studies of PGA structure–function relationships.

We report the first collection of synthetic sequence-defined PGA oligomers. Using automated glycan assembly (AGA), we achieved precise control over chain length and stereochemistry (Figure 1b), enabling systematic exploration of PGA’s conformational behavior and calcium-binding interactions. These insights are a basis for the rational design of PGA-based biomaterials for biomedical applications.

## Results and Discussion

### Design and Synthesis of a Glucuronic Acid Building Block

An effective building block (BB) for the automated glycan assembly of PGA oligomers should be a suitably reactive protected glycuronate donor and, upon incorporation into the growing solid phase chain, a good acceptor. Frequently, glucose is oxidized following glycosylation to introduce uronic acid residues into oligomers^31^ (Figure 1) and circumvent the low reactivity of uronic acid building blocks during glycosylations.^32^ However, the oxidation step is often incomplete and results in glycosidic bond cleavage.^33^ To overcome these limitations, we employed GlcA building blocks. Although the strong electron-withdrawing C6-carboxylate reduces the nucleophilicity of the acceptor,^34,35^ we designed a protecting group pattern that balanced stability and reactivity of the GlcA BB, enabling efficient iterative AGA.

GlcA building blocks **6–9** were designed with orthogonal protecting groups to provide both stereocontrol during glycosylation and selective deprotection during assembly. A benzoyl (Bz) ester was placed at C-2 to ensure the formation of trans-glycosides through neighboring-group participation, while a nonparticipating benzyl (Bn) ether was installed at C-3. Finally, the C-5 carboxylate was masked as a methyl ester (CO_2_Me). All protecting groups could be globally removed following AGA. The C-4 hydroxyl group, serving as the chain elongation site, was initially masked as a levulinoyl (Lev) ester.^36^ However, inefficient deprotection in longer oligomers, requiring repeated 30 min treatments extending the overall AGA cycle time, motivated the exploration of alternatives. Replacing Lev with a 9-fluorenylmethoxycarbonyl (Fmoc) group enabled rapid cleavage employing two 9 min cycles for longer chains, substantially improving the overall synthesis time and allowing for reliable access to extended oligomers. This protecting-group architecture 2-O-Bz (participating), 3-O-Bn (nonparticipating), 4-O-Fmoc (temporary), and 6-CO_2_Me reliably directed β-(1–4) glycosylations while providing orthogonal deprotection handles for iterative syntheses (Figure S1). GlcA donors were prepared as thioglycosides (**6**, **8**) and dibutyl phosphates (**7**, **9**). Initial AGA attempts to enhance the glycosylation efficiency of the thioglycosides by variations in temperature and employing double coupling cycles gave poor yields, consistent with the reduced reactivity of disarmed uronate donors. Switching to the more reactive glycosyl dibutyl phosphate donors^37^ improved coupling efficiency. Systematic optimization of the glycosylation conditions, including variation in temperature and coupling cycles, established that two cycles of glycosylation, each with five equivalents of building block **7** achieved maximal efficiency and stereoselectivity by performing the glycosylation step at −15 °C for 30 min followed by a 30-minute incubation at 0 °C (Table S2).

### Automated Glycan Assembly

AGA was performed on Merrifield polystyrene resin functionalized with a 5-aminopentanol photolabile linker on a 0.015 mmol scale. This linker proved advantageous, as flow photocleavage released the oligomers with an amino group at the reducing end, providing a versatile handle for downstream conjugation and biophysical assays.^38,39^ All syntheses were achieved on a custom-built AGA synthesizer with repetitive cycles.^40,41^ Each cycle includes four steps, (i) acidic wash–resin activation with trimethylsilyl triflate (TMSOTf), (ii) glycosylation–SEt donor activation promoted by *N*-iodosuccinimide (NIS) and triflic acid (TfOH) and phosphate donor activation promoted by TMSOTf, (iii) capping–blocking of unreacted acceptors with acetic anhydride (Ac_2_O) and methanesulfonic acid (MsOH), (iv) temporary protecting group cleavage–Lev ester hydrolysis promoted by hydrazine acetate in pyridine/AcOH/H_2_O and Fmoc carbonate hydrolysis promoted by 20% piperidine in DMF for further chain elongation (SI, Section 3.3). The reaction progress was carefully monitored by micro-photocleavage of a small portion of resin in DCM followed by analysis with HPLC-MS and MALDI-TOF. The number of coupling cycles was adjusted according to the target length, ranging from one cycle for compound **11** to 10 cycles for **23**. Normal-phase HPLC analysis revealed minor deletion sequences in the crude of **22**, as typically observed during iterative glycan assembly (SI, Section 4.7). Overall, this strategy enabled the synthesis of a series of fully protected β-(1–4)-linked glucuronic acid oligomers as long as decasaccharide **22** (Figure 2).

**Figure 2:**
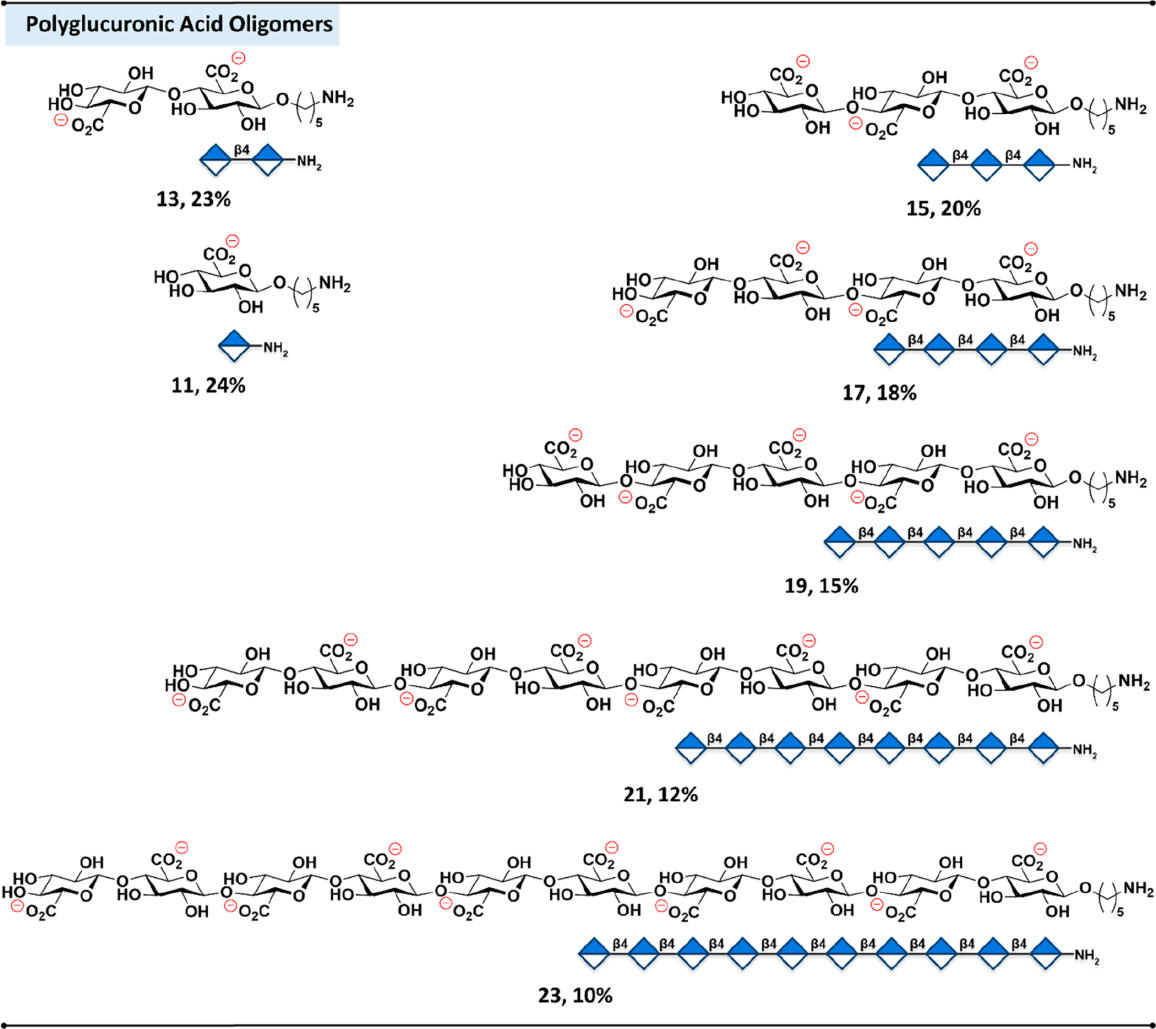
Polyglucuronic acid oligomers synthesized by AGA.

### Post-AGA

Following AGA, the fully protected oligomers were released from the resin using a flow-based photoreactor,^43^ purified by normal-phase HPLC, and characterized by NMR spectroscopy (SI, Section 4). Global deprotection was accomplished in two sequential stages: base-mediated saponification by lithium hydroxide (LiOH, 1 M) and hydrogen peroxide (H_2_O_2_, 30%) in H_2_O/THF (1:1, v/v) to cleave methyl esters and benzoyl ester protecting groups, followed by hydrogenolysis of the remaining benzyl ethers using palladium hydroxide (Pd­(OH)_2_) in H_2_O/^t^BuOH (1:1, v/v). Post-AGA treatment cleanly unmasked all carboxylates, hydroxyls, and amino groups, affording the fully deprotected PGA oligomers that were purified using Sephadex LH-20 with H_2_O/MeOH (1:1, v/v) as eluent (SI, Section 3.4) in overall yields of 10–25% (Figure 2). NMR analysis confirmed the presence of β-linkages (^1^H, 4–4.5 ppm, d, *J* = ∼7.8 Hz and ^13^C at ∼100 ppm), closely matching with β-(1–4)-d-polyglucuronic acid extracted from mutant strain M5Nl CS of *Rhizobium meliloti*.^44^

### Molecular Dynamics Simulations

Single-molecule atomistic MD simulations were performed to investigate the conformational preferences of polyglucuronic acid oligomers. All the modeled structures were simulated for 500 ns unless otherwise specified, employing a modified version of the GLYCAM06 carbohydrate force field.^45^ The MD simulation revealed that all oligomers predominantly adopt a 2-fold helical conformation, with alternating carboxylate groups oriented on opposite sides of the polymer axis (Figure 4b), generating a polyanionic surface that is well positioned for ion coordination.^46^ Ramachandran plots of dihedral angles (Φ/Ψ) show that the β-(1–4) glycosidic linkages exhibit a standard *exo-syn* conformation, as observed for cellulose,^47^ and puckering analysis confirmed that all glucuronic acid residues remain in the ^4^C_1_ chair conformation (SI, Section 5.3). Up to pentasaccharide **19**, the chains remained relatively rigid. Increasing the chain length in **21** and **23** is associated with a slight enhancement in conformational flexibility, as evidenced by the broader distribution of the radius of gyration (Rog) (Figure 3a,b). Despite this increased flexibility, the 2-fold helical scaffold remained preserved across all chain lengths.

**Figure 3:**
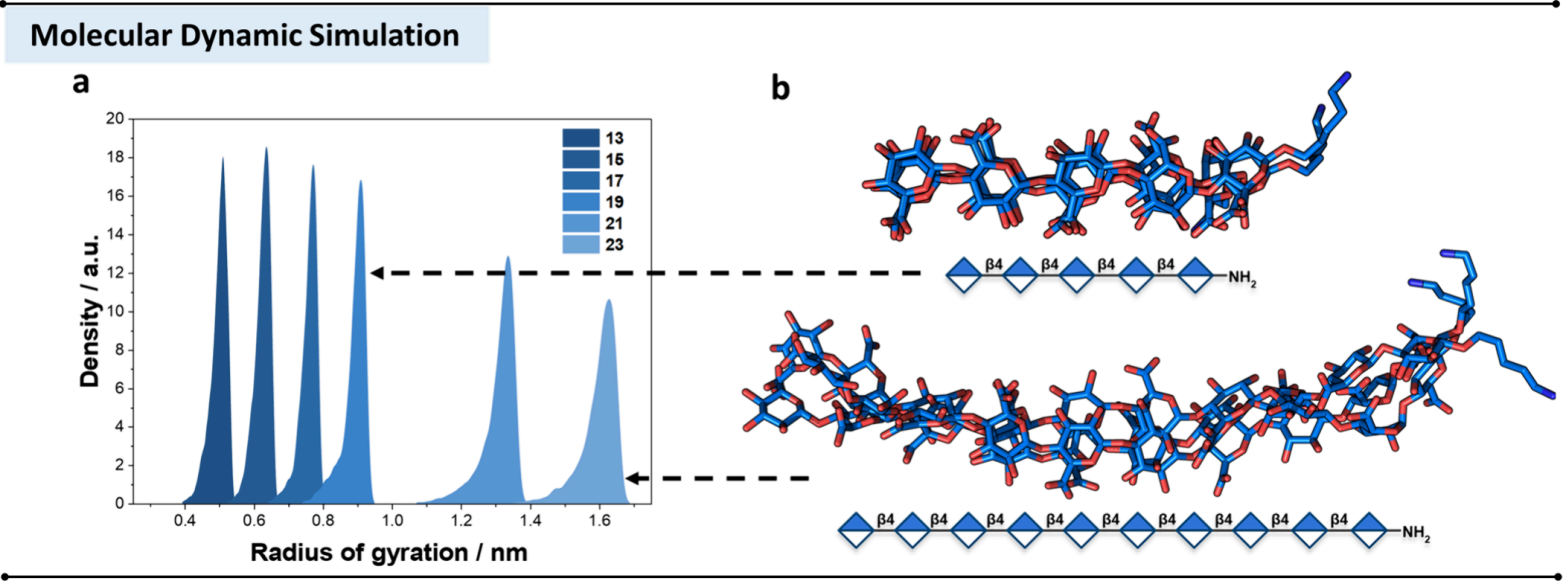
a) Radius of gyration plots for **13**–**23** extracted from MD simulations. b) Representative snapshots of **19** and **23** extracted from the MD simulation with the conformation clustering algorithm GlycanAnalysisPipeline.^42^

**Figure 4:**
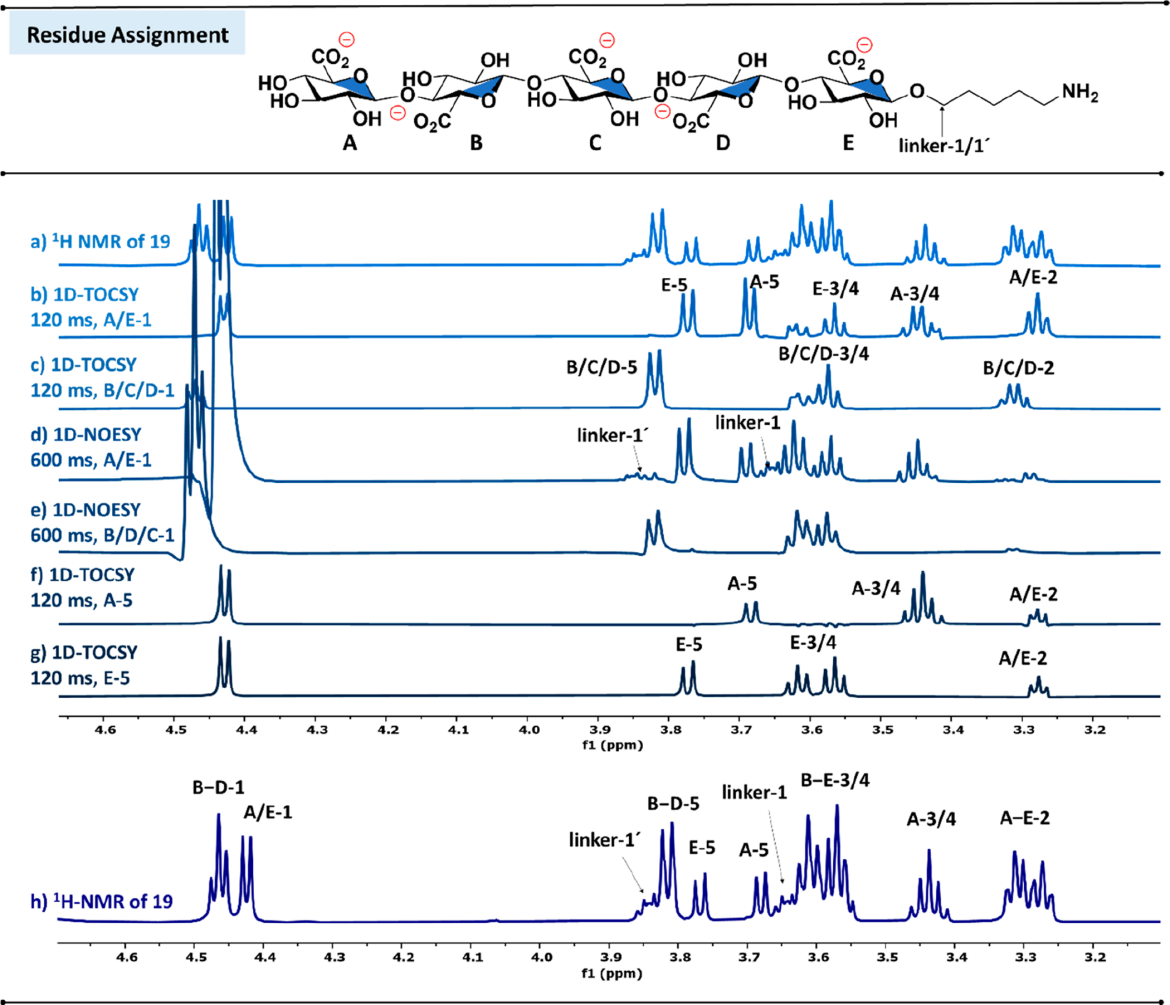
Proton resonance assignment of glycan residues for **19**. Only the glycan region is shown in the spectra. Letters denote individual glycan residues, and numbers indicate the protons attached to each carbon of the corresponding residue, starting with the proton at the anomeric carbon (labeled as 1). Labels linker 1 and linker 1′ refer to the two protons bound to the secondary carbon of the 5-aminopentanol linker that is covalently attached via a glycosidic bond to glucuronic acid residue E. a) ^1^H NMR of **19**. b) and c) Selective 1D TOCSY (pulse program: selmlgp; mixing time d9 = 120 ms) of B/C/D-1 and A/E-1 allowing assignment of proton resonances within the same spin system. The complete 1D-TOCSY sequence (mixing times d9 = 20, 40, 60, 80, 120 ms) is available in the SI. d) and e) Selective 1D NOESY (pulse program: selnogp; mixing time d8 = 600 ms) of A/E-1 and B/C/D-1 indicating A/E-1 as terminal residues due to NOE with proton resonances of closest secondary carbons of the 5-aminopentanol linker (linker-1 and linker-1′). f) and g) Selective 1D-TOCSY of A-5 and E-5 that allowed distinguishing protons 3 and 4 of glycan residues A and E. h) ^1^H NMR spectra of **19** showing the full assignment of proton resonances indicating two different sets of anomeric proton resonances.

### NMR Structural and Calcium Binding Analysis

Calcium binding is an essential functional characteristic of uronic acid polysaccharides in gel networks.^48^ In alginic acid, cooperative coordination of Ca^2+^ by the α-(1–4)-glucuronic acid generates stable egg-box junctions that underpin rigid and thermally resilient gels,^49,50^ whereas the β-(1–4)-mannuronic acids engage in weaker and more reversible Ca^2+^ interactions.^51,52^ Less is known about how PGA interacts with calcium. Computational modeling of PGA suggests a different mode of interaction, where Ca^2+^ associates through diffuse electrostatic interactions, forming a dynamic cloud of favorable positions along the polymer chain.^7^ To investigate how Ca^2+^ binding translates into defined β-(1–4)-PGA oligomers, we examined interactions of Ca^2+^ with sequence-defined oligomers using NMR spectroscopy. Titrations of the PGA pentasaccharide **19** and decasaccharide **23** were performed at constant oligomer concentration (2 mM) and TRIS-*d*_11_ buffer (10 mM) while adding CaCl_2_ to reach a concentration from 2 to 20 mM. Precipitation was observed at concentrations of CaCl_2_ exceeding 10 mM.

Proton resonances of the glycan residues were assigned using ^1^H NMR in combination with selective one-dimensional (1D) total correlation spectroscopy (TOCSY), nuclear Overhauser effect spectroscopy (NOESY) experiments, and heteronuclear single quantum coherence spectroscopy (HSQC) (Figure 4h). ^1^H NMR revealed two separate sets of anomeric protons at 4.46 ppm (B/C/D-1) and 4.42 ppm (A/E-1) that were integrated in a ratio of 3/2. We initially tentatively assigned the two sets to internal (B/C/D) and terminal residues (A/E). Selective 1D-TOCS*Y* experiments (Figure 4b and c) irradiating B/C/D-1 and A/E-1 were performed to assign all of the remaining resonances of the two different sets of spin systems. Selective 1D-NOESY experiments upon irradiation of A/E-1 (Figure 4d) showed an NOE cross peak between A/E-1 and the CH_2_-protons of the 5-aminopentanol linker alkyl chain (linker-1 and linker-1′). Such an NOE was absent in 1D-NOESY upon selective irradiation of B/C/D-1 (Figure 4e). In addition, the selective irradiation of A/E-1 in 1D-TOCSY experiments revealed two separate resonance sets for protons attached to ring carbons C-3, C-4, and C-5, further assigned with additional 1D-TOCSY experiments (Figure 4f and g). Similar chemical shifts were observed for E-3/4 and B/C/D-3/4, whereas A-3/4 was shifted upfield due to the absence of a glycosidic linkage at ring carbon C-4. These experiments supported our initial assignments of two sets of anomeric resonances. Ca^2+^ addition induced uniform downfield shifts for all proton resonances in the ^1^H NMR (Figure 5), consistent with proton-deshielding effects through electrostatic interactions with nearby hydroxyl and carboxylate groups. These perturbations were evenly distributed along the backbone but slightly more pronounced at the chain termini (residue A), suggesting the initiation of binding at the more exposed residue.

**Figure 5:**
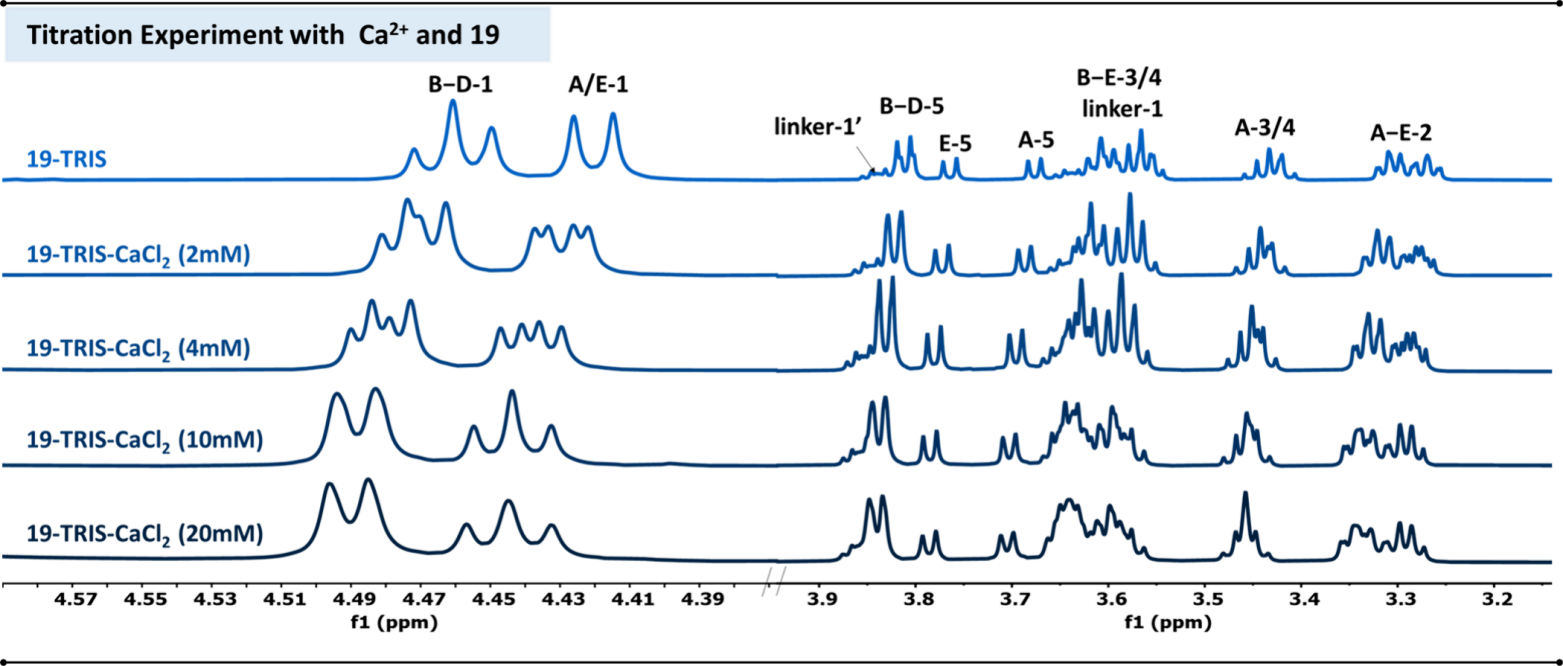
a) Titration experiments to evaluate possible interactions of compound **19** with Ca^2+^. The concentration of **19** and Tris d_11_ buffer was kept constant at 2 and 10 mM, respectively, while Ca^2+^ concentration was increased in steps of 2, 4, 10, and 20 mM. Two spectral regions of the ^1^H NMR spectra are shown, comprising all detectable glycan protons: left: 4.56–4.25 ppm (anomeric region); right: 4.00–3.10 ppm.

Compound **23** displayed similar, but attenuated effects (Figure S41). Relative to guluronic acid polymers, the perturbations in PGA were less pronounced and more closely resembled those in polymannuronic acid, indicating diffuse, nonspecific electrostatic interactions rather than localized chelation^53^ Overall, MD simulations and NMR titration experiments provide molecular-level insights into how β-(1–4)-linked PGA oligomers engage with Ca^2+^.

## Conclusions

Glycosylation conditions for glucuronic acid building blocks were established for the efficient β-selective synthesis of linear polyglucuronic acid oligomers up to decasaccharides through iterative AGA cycles. Structural analysis by molecular dynamics simulations revealed that oligomers predominantly adopt a 2-fold helical conformation providing a defined scaffold for probing ion interactions. Complementary NMR titration experiments demonstrated that calcium binding occurs via diffuse electrostatic interactions along the PGA backbone, rather than through discrete, site-specific chelation, consistent with the uniform perturbations observed across the NMR spectra. The collection of synthetic sequence-defined PGA oligomers enables controlled studies of ion interactions and conformational dynamics.

## Supplementary Material



## Notes

Experimental section: automated glycan assembly synthetic protocols, characterization data including HPLC, HR-MS, and NMR, MD simulation details, Ca^2+^ binding NMR titration data (PDF)
