# Supplementary material for: Automated Assembly of Polyglucuronic Acids for Structural Explorations
Source: J Am Chem Soc. 2026 Feb 11;148(7):7588–95. doi: 10.1021/jacs.5c21268 (PMC12951461; doi:10.1021/jacs.5c21268)
Supplement: Supplementary file 1 [file ja5c21268_si_001.pdf]

Supporting Information

Automated Assembly of Polyglucuronic Acids for Structural Explorations

Sandhya N. Mardhekar,[⊥] Dominik Weh,[⊥,§] Martina Delbianco,[⊥] Peter H. Seeberger*[,⊥,§]

[⊥]Department of Biomolecular Systems, Max-Planck-Institute of Colloids and Interfaces, Am Mühlenberg 1, 14476 Potsdam, Germany

[§]Department of Chemistry and Biochemistry, Freie Universität Berlin, Arnimallee 22, 14195, Berlin, Germany

Email: peter.seeberger@mpikg.mpg.de

Supporting Information

Table of Contents

1 General materials and methods 4
2 Building block synthesis for AGA 5
3 Automated Glycan Assembly 30
3.1 General materials and methods 30
3.2 Preparation of stock solutions 30
3.3 Modules for automated synthesis 31
3.3.1 **Module A: Resin preparation** 32
3.3.2 **Module B: Acidic wash with TMSOTf solution** 32
3.3.3 **Module C: Thioglycosides glycosylation** 32
3.3.4 **Module D: Glycosyl phosphate glycosylation** 33
3.3.5 **Module E: Capping** 34
3.3.6 **Module F: Lev deprotection** 35
3.3.7 **Module G: Fmoc deprotection** 35
3.4 Post-AGA manipulations 36
3.4.1 **Module H: Cleavage from solid support** 36
3.4.2 **Module I: Micro-cleavage from solid support** 36
3.4.3 **Module J: Off-resin hydrolysis** 36
3.4.4 **Module K: Hydrogenolysis** 36
3.4.5 **Module L: Purification/Analysis** 37
4 Oligosaccharides syntheses 38
Figure S2. Oligosaccharides synthesized by AGA. 38
4.1 Synthesis and analytical data of 11 39
4.2 Synthesis and analytical data of 13 47
4.3 Synthesis and analytical data of 15 55
4.4 Synthesis and analytical data of 17 63
4.5 Synthesis and analytical data of 19 71
4.6 Synthesis and analytical data of 21 80
4.7 Synthesis and analytical data of 23 89
5 Structural Analysis 98
5.1 General materials and methods for molecular dynamics simulations 98

5.2 General materials and methods for NMR titrations 98
5.3 Molecular Dynamics 100
5.3.1 Compound **13** 100
5.3.2 Compound **15** 101
5.3.3 Compound **17** 102
5.3.4 Compound **19** 104
5.3.5 Compound **21** 105
5.3.6 Compound **23** 107
5.4 NMR titration study 110
5.4.1 NMR characterization and Ca^{2+} titration study of **19** 110
5.4.2 NMR characterization and Ca^{2+} titration study of **23** 117
6 References 121

1 General materials and methods

All chemicals used were reagent grade and used as supplied unless otherwise noted. The automated syntheses were performed on a home-built synthesizer developed at the Max Planck Institute of Colloids and Interfaces.[1] Analytical thin-layer chromatography (TLC) was performed on Merck silica gel 60 F254 plates (0.25 mm). Compounds were visualized by UV irradiation or dipping the plate in a staining solution (sugar stain: 10% H_2SO_4 in EtOH; CAM: 48 g/L ammonium molybdate, 60 g/L ceric ammonium molybdate in 6% H_2SO_4 aqueous solution). Analysis and purification by normal phase HPLC were performed using the Agilent 1260 series equipped with a multiple wavelength detector (MWD) and an evaporative light scattering detector (ELSD). Products were lyophilized using a Christ Alpha 2-4 LD plus freeze dryer. ^{1}H, ^{13}C and HSQC NMR spectra were recorded on a Varian 400-MR (400 MHz), Varian 600-NMR (600 MHz), Bruker Bio spin AVANCE700 (700 MHz) spectrometer. Spectra were recorded in $CDCl_3$ by using the solvent residual peak chemical shift as the internal standard ($CDCl_3$: 7.26 ppm ^{1}H, 77.0 ppm ^{13}C) or in D_2O using the solvent as the internal standard in ^{1}H NMR (D_2O: 4.79 ppm ^{1}H). ^{1}H NMR spectra for all compounds were recorded without ^{13}C decoupling. Weak intensity ^{13}C resonances were derived from the respective HSQC cross peaks. ^{1}H NMR integrals of the resonances corresponding to residues at the reducing end are reported as non-integer numbers and the sum of the integrals of α and β anomers is set to 1. High resolution mass spectra were obtained using a Xevo G2-XS QTof mass spectrometer (HRMS, Waters) and a MALDI-TOF autoflexTM (Bruker).

2 Building block synthesis for AGA

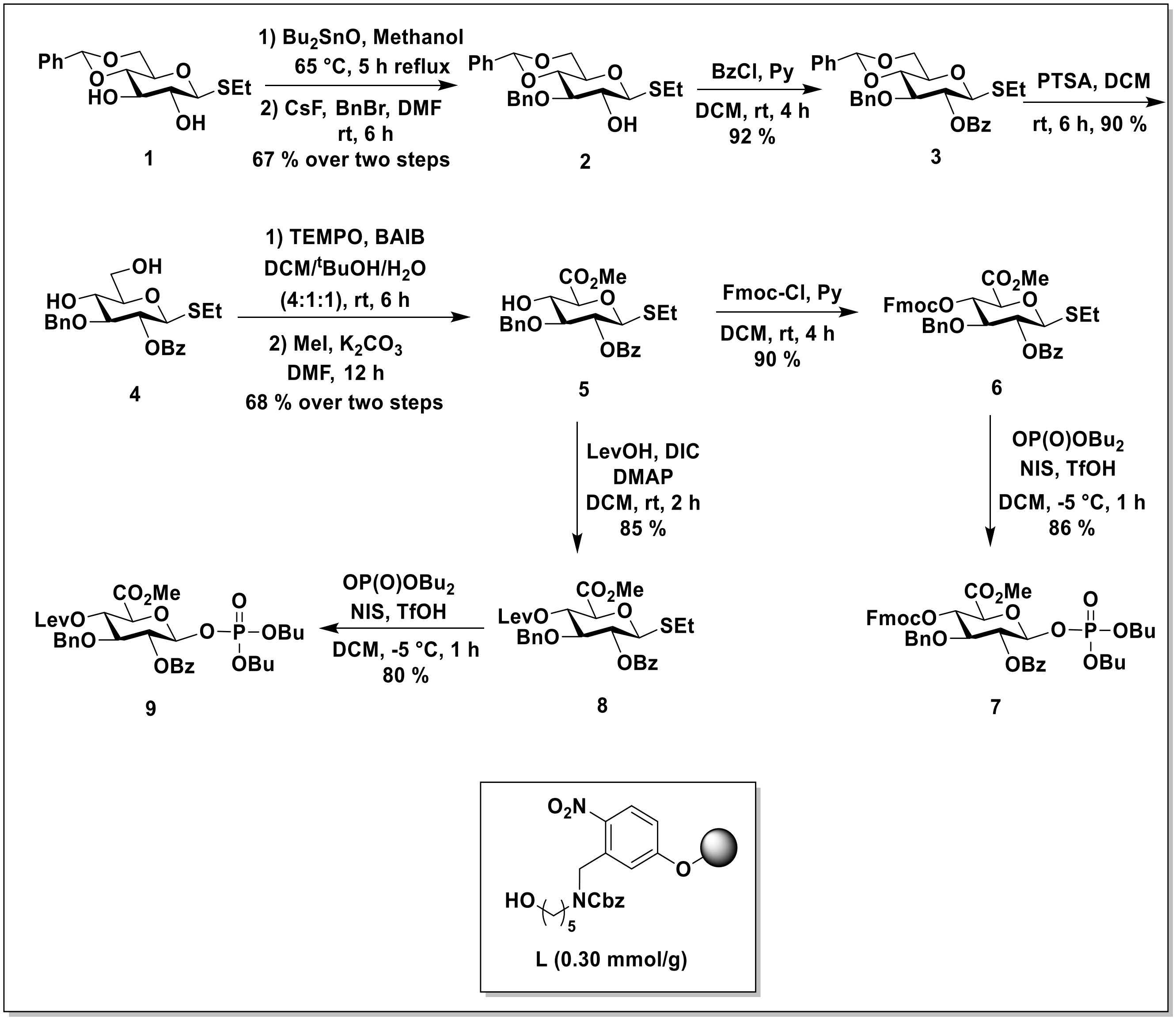

Figure S1. Synthesis of glucuronic acid building blocks.

Compound **1** was purchased from GlycoUniverse GmbH**,** and subsequently modified according to the following procedures to synthesize required building blocks. Merrifield resin equipped with photocleavable linkers **L** (loading 0.30 mmol/g) was prepared according to previously reported procedures.[1]

Synthesis of compound 2

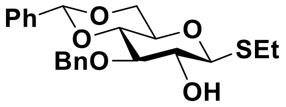

A mixture of diol thioglycoside, **1** (15.0 g, 48.0 mmol) and dibutyltin oxide ($^nBu_2SnO$, 10.1 g, 72.1 mmol) dissolved in methanol (120 mL) was heated under reflux for five hours. After cooling to room temperature, the reaction was concentrated under reduced pressure, and the residue was dissolved in DMF and cesium fluriode (CsF, 3.6 g, 24.0 mmol) and benzyl bromide (BnBr, 5.7 mL, 48.0 mmol) were added. The mixture was refluxed for an additional 6 h (monitored by TLC), The reaction mixture neutralized with water and residue was diluted with EtOAc, washed sequentially with saturated aqueous $NaHCO_3$ and brine. The organic layer was dried over Na_2SO_4, concentrated under reduced pressure, purified by silica gel column chromatography (Hexane/EtOAc) to afford **2** (13 g, 67%) as a white solid.

^{1}H NMR (400 MHz, $CDCl_3$): δ 7.45 – 7.34 (m, 2H), 7.30 – 7.24 (m, 5H), 7.24 – 7.14 (m, 3H), 5.46 (s, 1H), 4.86 (d, *J* = 11.6 Hz, 1H), 4.70 (d, *J* = 11.6 Hz, 1H), 4.34 (d, *J* = 9.7 Hz, 1H), 4.24 (dd, *J* = 10.5, 4.9 Hz, 1H), 3.72 – 3.52 (m, 3H), 3.51 – 3.42 (m, 1H), 3.43 – 3.32 (m, 1H), 2.63 (qd, *J* = 7.4, 3.2 Hz, 2H), 2.52 (d, *J* = 4.5 Hz, 1H), 1.19 (t, *J* = 7.5 Hz, 3H). **^{13}C NMR** (101 MHz, $CDCl_3$): δ 138.32, 137.24, 129.07, 128.52, 128.33, 128.12, 127.90, 126.06, 101.27, 86.60, 81.59, 81.24, 74.76, 73.00, 70.75, 68.68, 24.63, 15.31. **HRMS QTOF-MS**: calcd. $C_{22}H_{26}NaO_5S$ for $[M+Na]^+$ 425.1399, found 425.1392.

^{1}H NMR of compound 2 (400 MHz, $CDCl_3$)

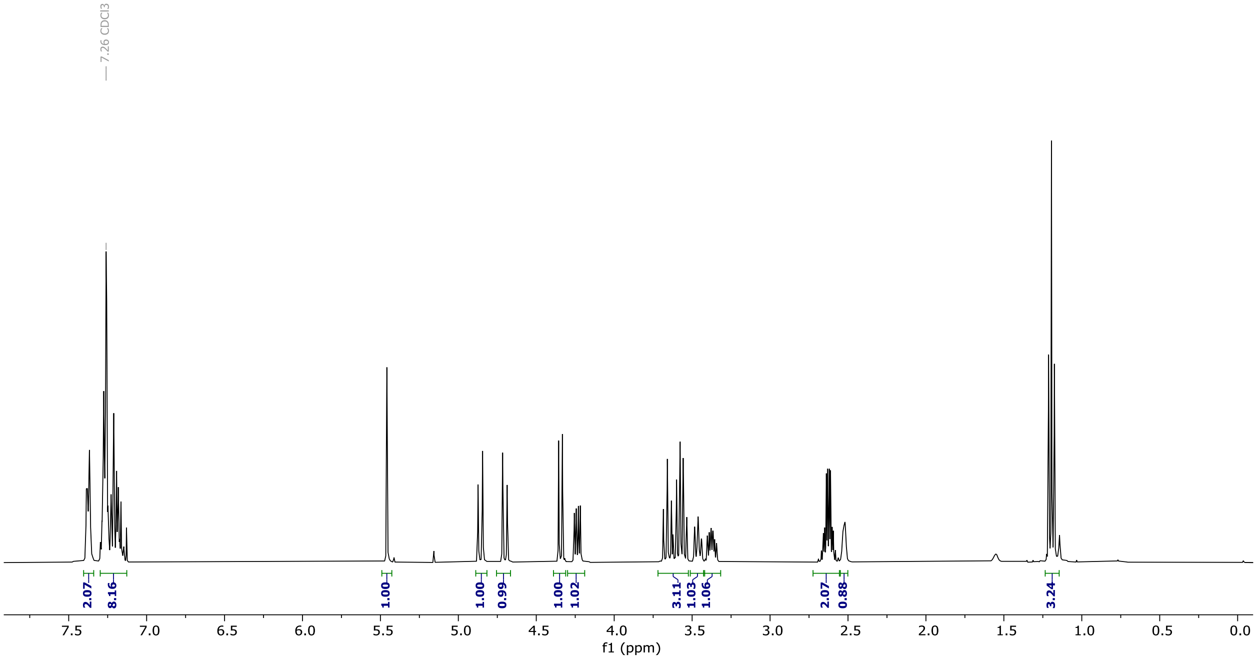

^{13}C NMR of compound 2 (101 MHz, $CDCl_3$)

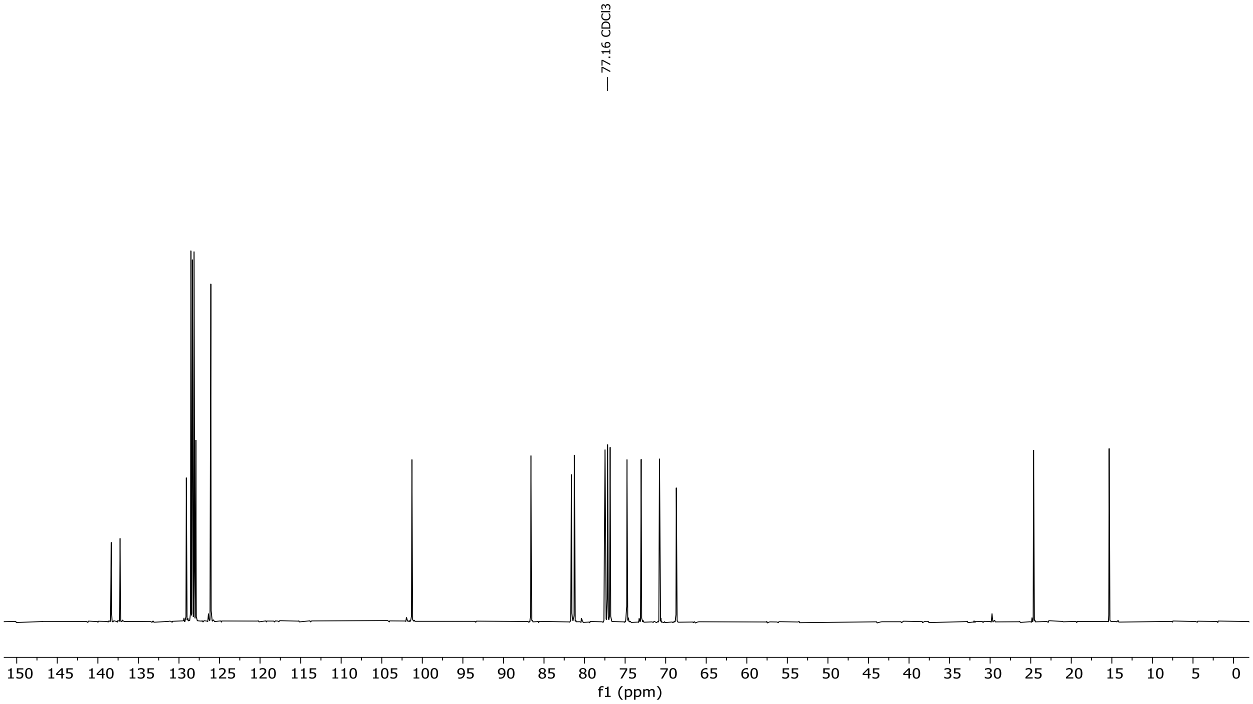

DEPT-135 NMR of compound 2 (101 MHz, $CDCl_3$)

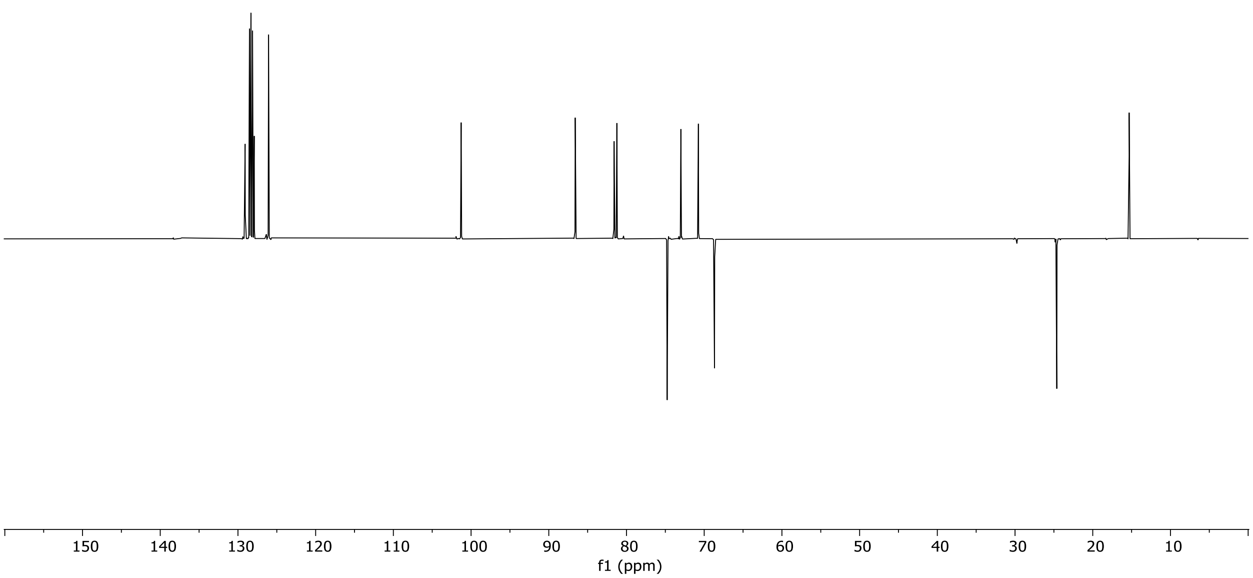

^{1}H-^{13}C HSQC NMR of compound 2 (600 MHz, $CDCl_3$)

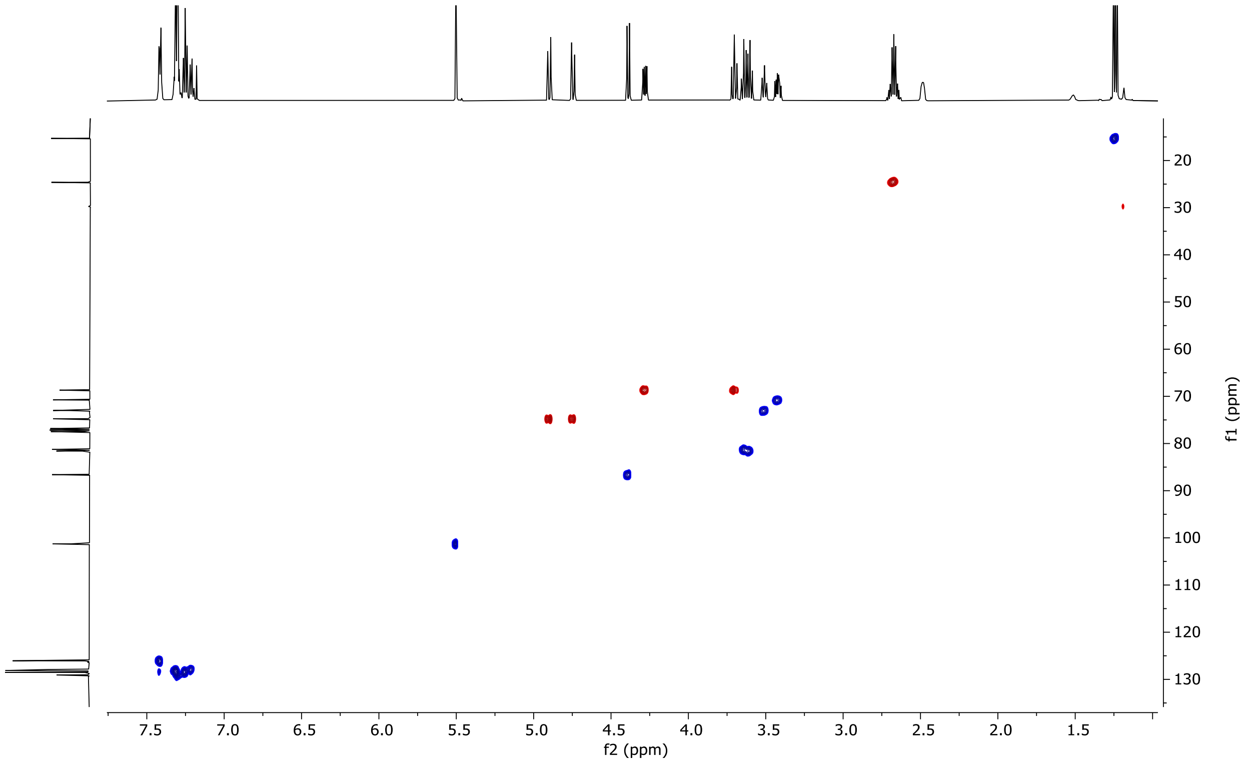

^{1}H-^{1}H COSY NMR of compound 2 (600 MHz, $CDCl_3$)

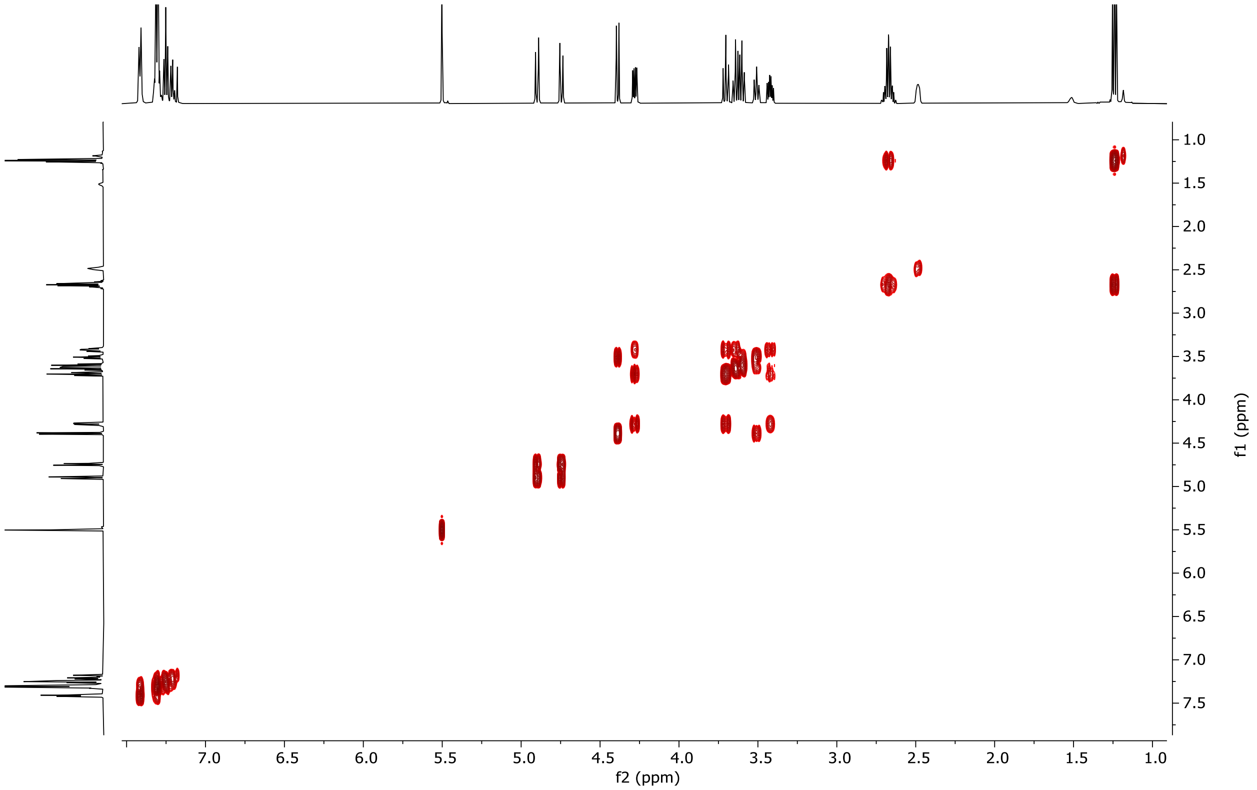

Synthesis of compound 3

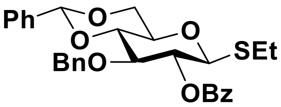

Compound **2** (12.0 g, 29.8 mmol) was dissolved in dichloromethane (DCM, 90 mL) and treated with benzoyl chloride (6.9 mL, 59.6 mmol) and 4-dimethylaminopyridine (DMAP, 7.2 g, 59.6 mmol). The reaction mixture was stirred at room temperature for four hours (monitored by TLC). The reaction was diluted with ethyl acetate (EtOAc), washed sequentially with saturated aqueous $NaHCO_3$, H_2O, and dried over Na_2SO_4. After filtration and concentration under reduced pressure, the crude residue was purified by flash column chromatography (Hexane/EtOAc) to afford **3** (13.8 g, 92%) as a white solid.

^{1}H NMR (400 MHz, $CDCl_3$): δ 8.03 (dd, *J* = 8.1, 1.5 Hz, 2H), 7.66 – 7.57 (m, 1H), 7.56 – 7.52 (m, 2H), 7.50 – 7.38 (m, 5H), 7.18 – 7.07 (m, 5H), 5.64 (s, 1H), 5.36 (m, 1H), 4.85 (d, *J* = 11.9 Hz, 1H), 4.72 (m, 1H), 4.64 (d, *J* = 10.1 Hz, 1H), 4.43 (dd, *J* = 10.5, 4.9 Hz, 1H), 3.97 – 3.80 (m, 3H), 3.63 – 3.53 (m, 1H), 2.74 (qd, *J* = 7.5, 3.3 Hz, 2H), 1.24 (t, *J* = 7.5 Hz, 3H). **^{13}C NMR** (101 MHz, $CDCl_3$): δ 165.25, 137.82, 137.25, 133.30, 130.00, 129.76, 129.14, 128.46, 128.39, 128.25, 128.14, 127.67, 126.09, 101.31, 84.35, 81.71, 79.22, 74.28, 71.89, 70.76, 68.70, 24.11, 14.90. **HRMS QTOF-MS**: calcd. $C_{29}H_{30}NaO_6S$ for $[M+Na]^+$ 529.1661, found 529.1663.

^{1}H NMR of compound 3 (400 MHz, $CDCl_3$)

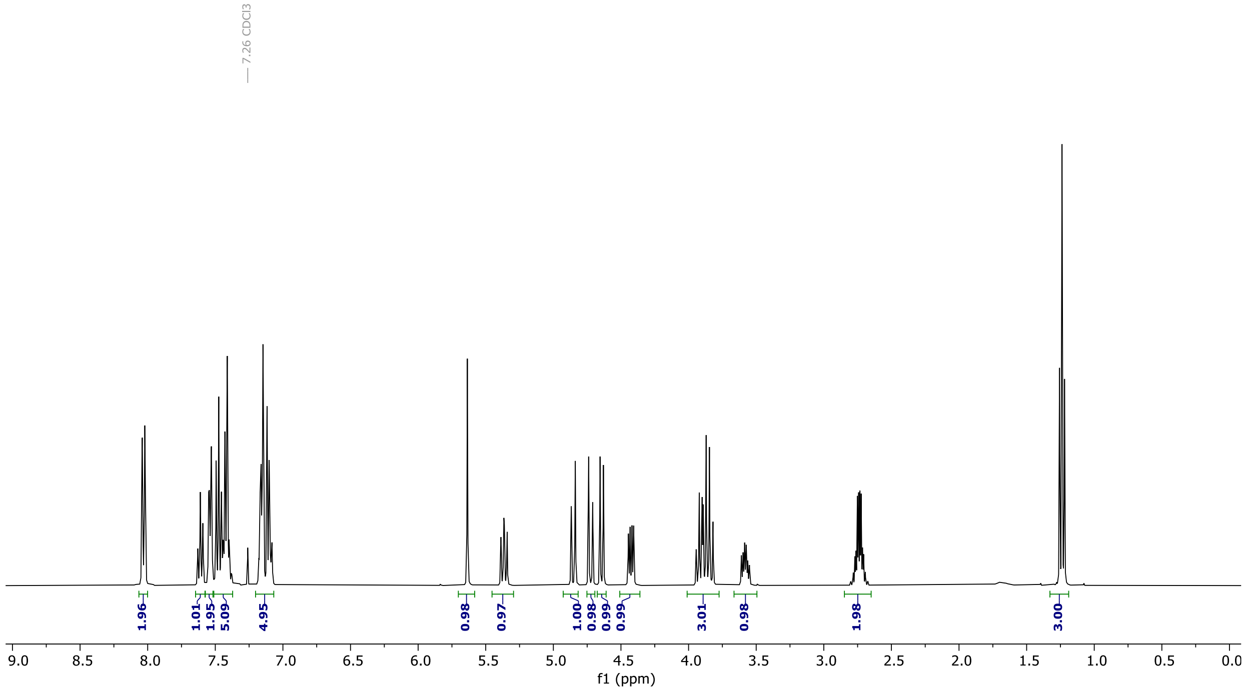

^{13}C NMR of compound 3 (101 MHz, $CDCl_3$)

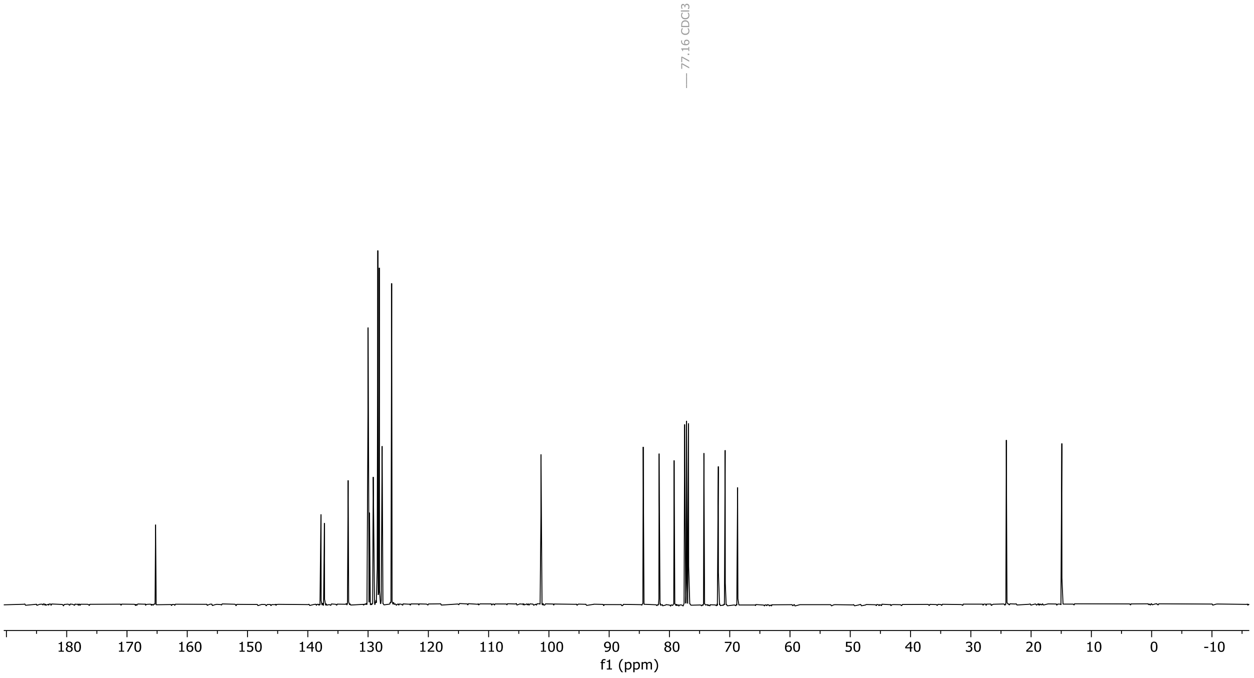

DEPT-135 NMR of compound 3 (101 MHz, $CDCl_3$)

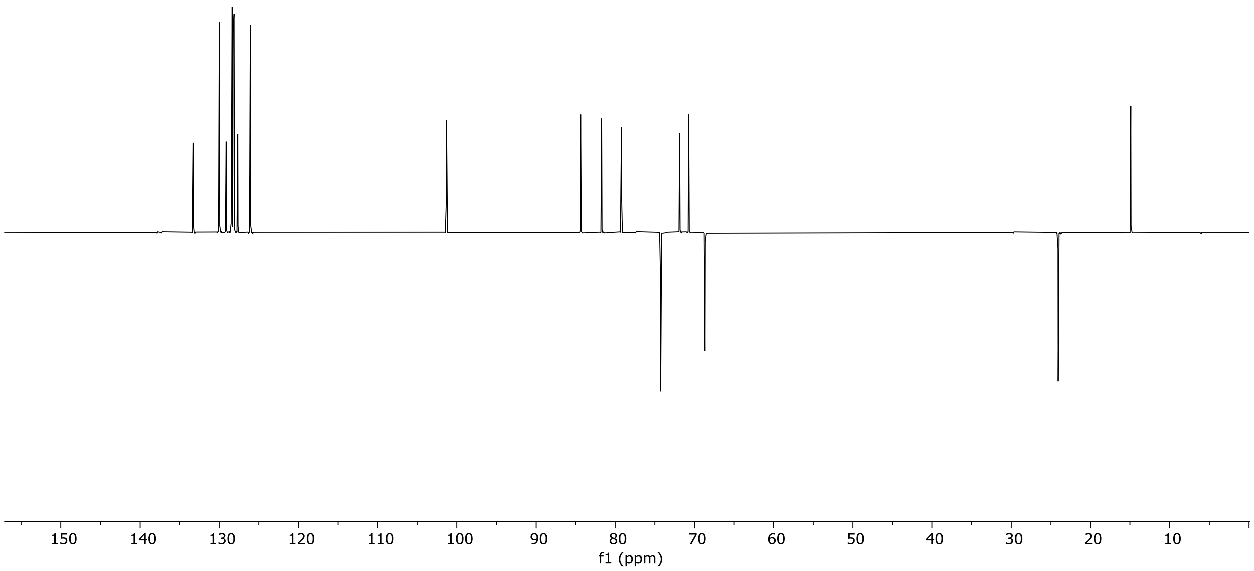

^{1}H-^{13}C HSQC NMR of compound 3 (600 MHz, $CDCl_3$)

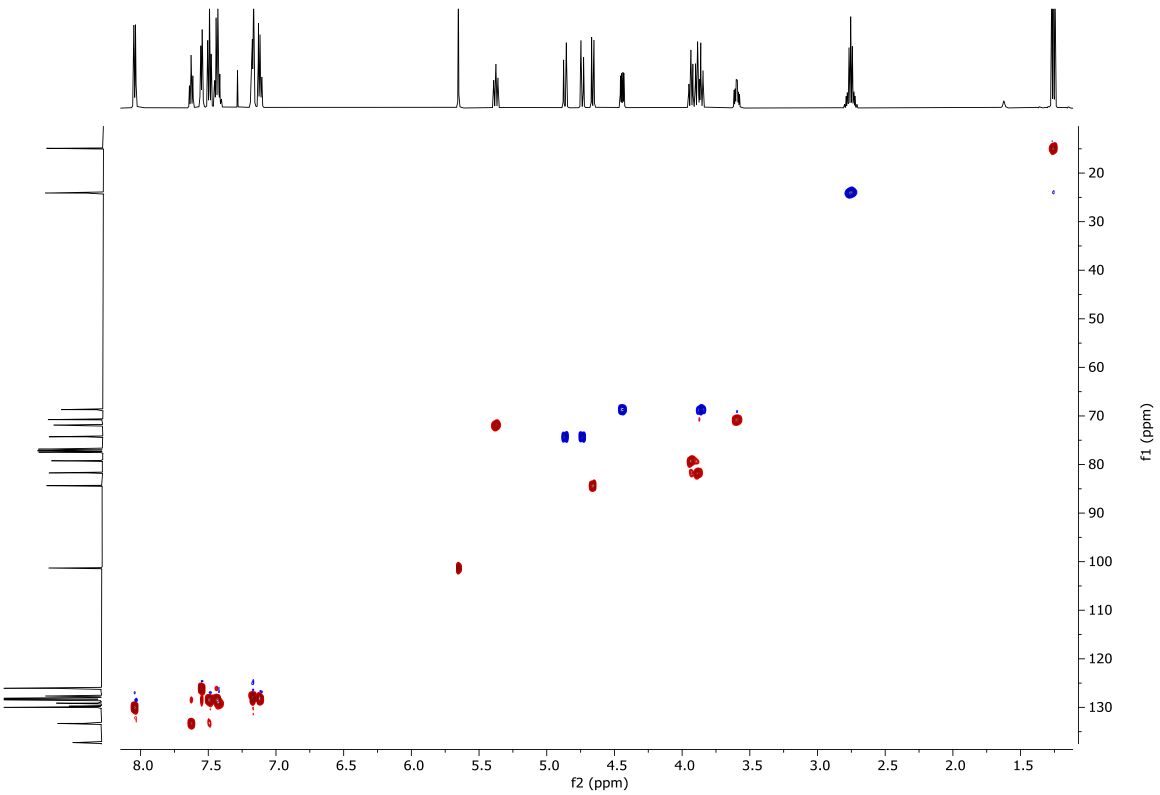

^{1}H-^{1}H COSY NMR of compound 3 (600 MHz, $CDCl_3$)

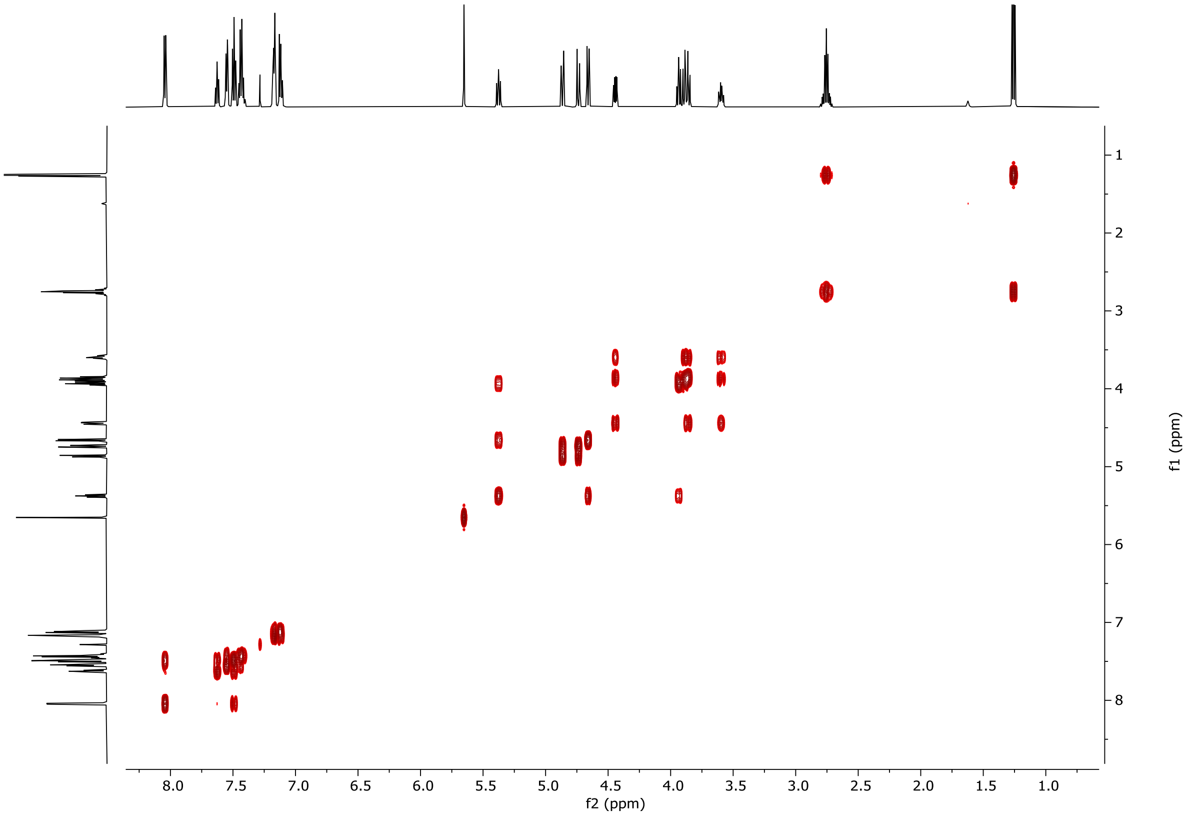

Synthesis of compound 4

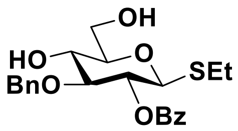

Compound **3** (13.0 g, 25.6 mmol) was dissolved in a 1:1 mixture of DCM/MeOH (120 mL), and *p*-toluenesulfonic acid (*p*-TSA, 5.8 g, 30.8 mmol) was added. The reaction mixture was stirred at room temperature for six hours until completion (monitored by TLC). The reaction mixture neutralized with triethyl amine, concentrated under reduced pressure, and the residue was purified by silica gel chromatography (Hexane/EtOAc) to afford **4** (9.66 g, 90%).

^{1}H NMR (400 MHz, $CDCl_3$): δ 8.07 (dd, *J* = 8.6, 1.3 Hz, 2H), 7.66 – 7.57 (m, 1H), 7.51 – 7.44 (m, 2H), 5.33 – 5.25 (m, 1H), 4.79 – 4.67 (m, 2H), 4.62 (d, *J* = 10.0 Hz, 1H), 3.96 (dd, *J* = 12.0, 3.4 Hz, 1H), 3.89 – 3.70 (m, 3H), 3.52 – 3.45 (m, 1H), 2.73 (qd, *J* = 7.5, 3.9 Hz, 2H), 1.24 (t, *J* = 7.5 Hz, 3H). **^{13}C NMR** (101 MHz, $CDCl_3$): δ 165.42, 137.86, 133.40, 129.76, 128.56, 128.54, 128.11, 127.98, 83.81, 83.78, 79.67, 74.88, 72.27, 70.48, 62.48, 14.95. **HRMS QTOF-MS**: calcd. $C_{22}H_{26}NaO_6S$ for $[M+Na]^+$ 441.1348, found 441.1334.

^{1}H NMR of compound 4 (400 MHz, $CDCl_3$)

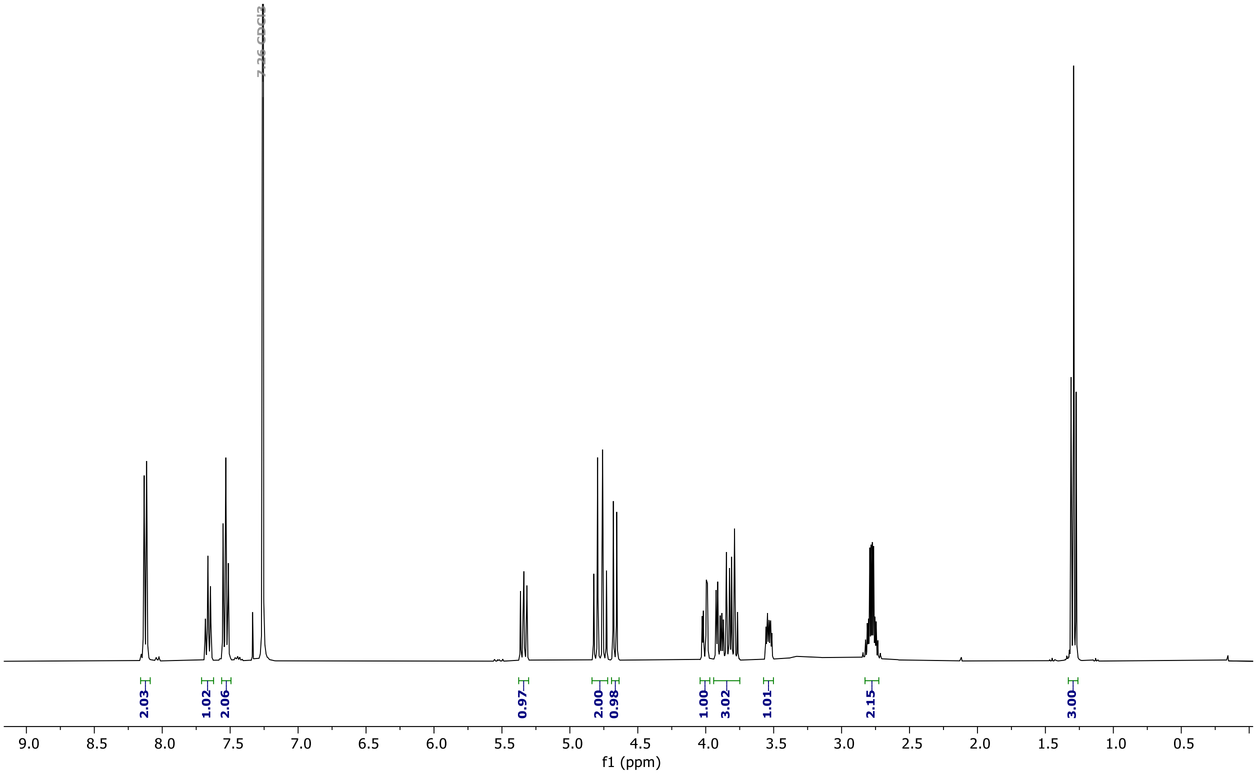

^{13}C NMR of compound 4 (101 MHz, $CDCl_3$)

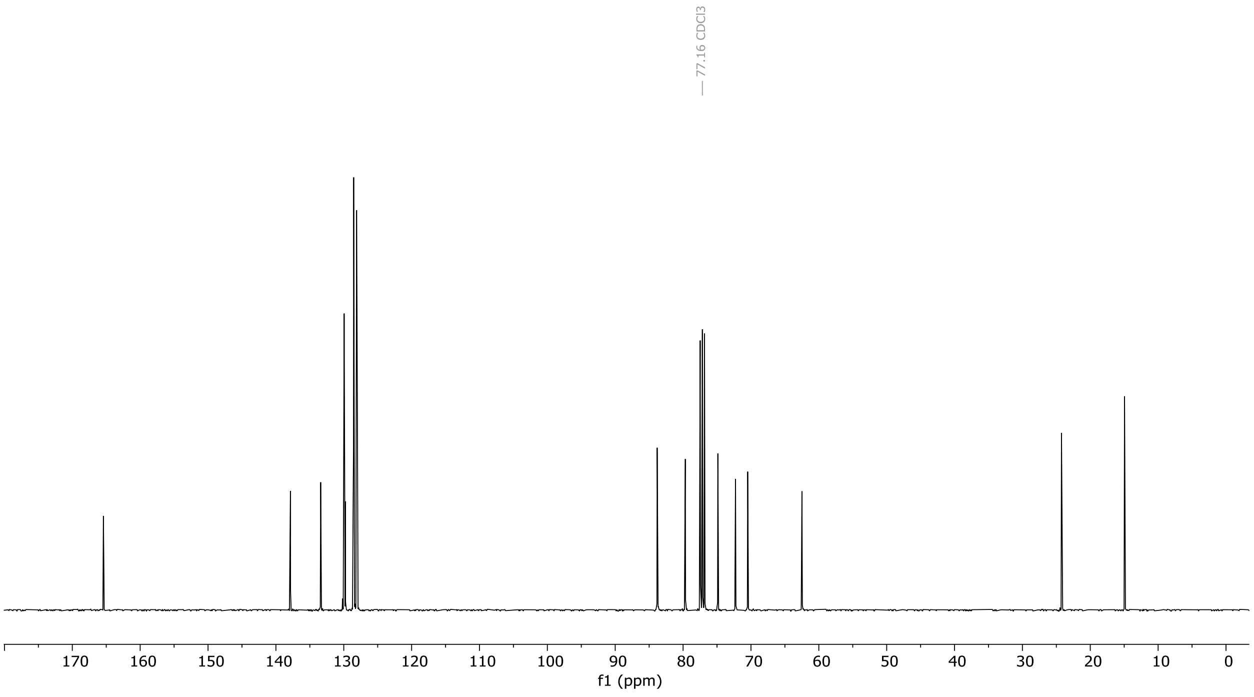

DEPT-135 NMR of compound 4 (101 MHz, $CDCl_3$)

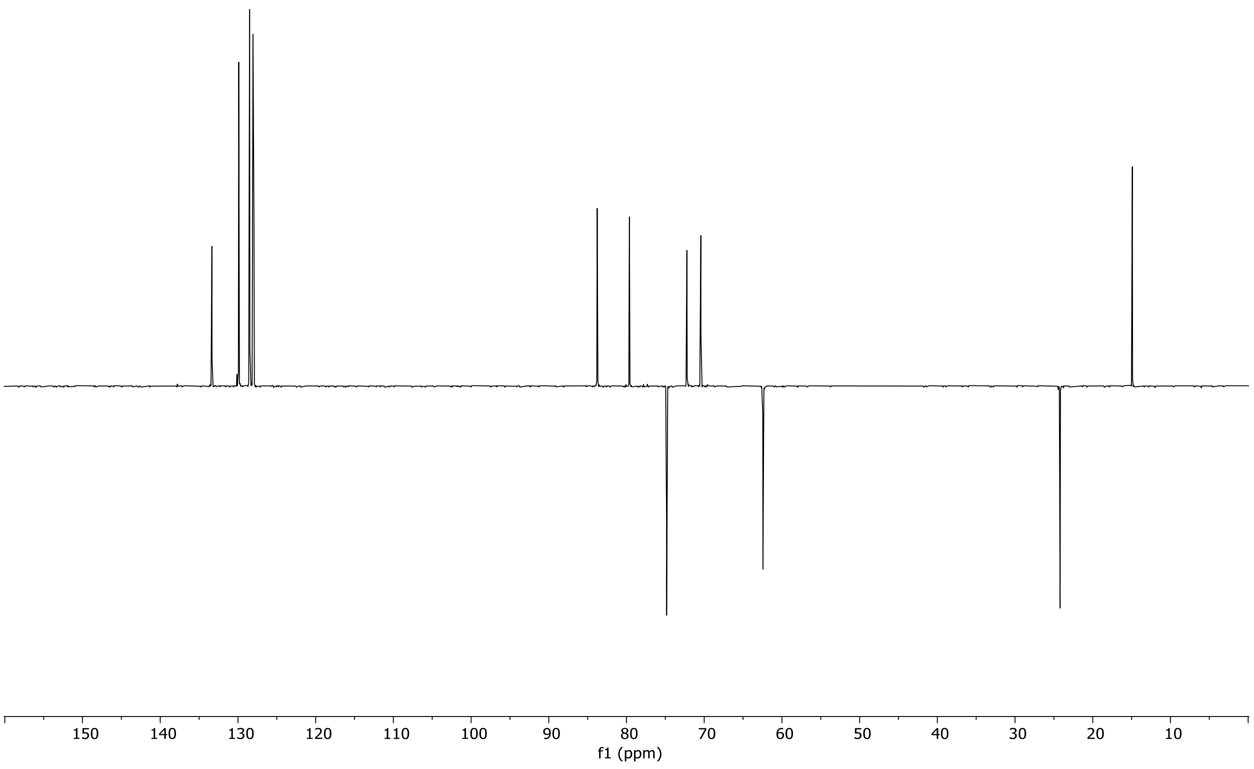

^{1}H-^{13}C HSQC NMR of compound 4 (600 MHz, $CDCl_3$)

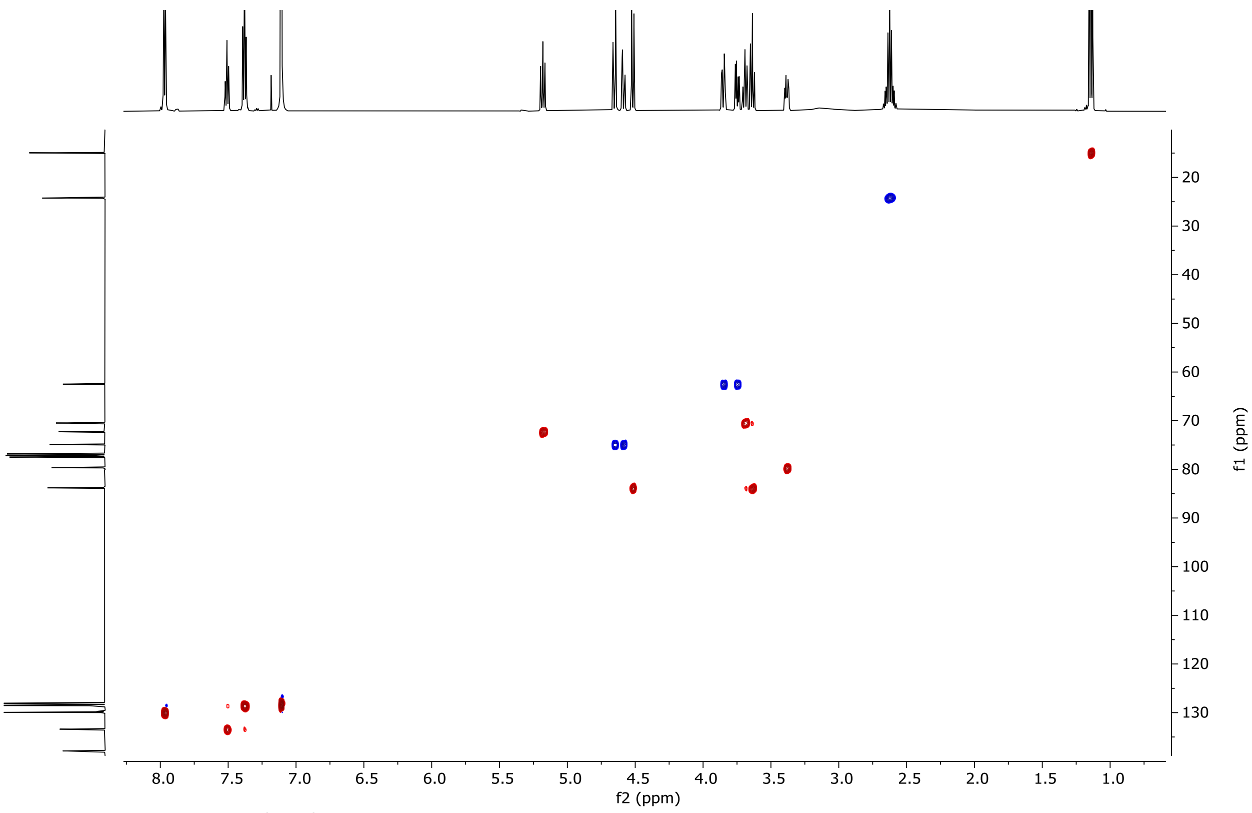

^{1}H-^{1}H COSY NMR of compound 4 (600 MHz, $CDCl_3$)

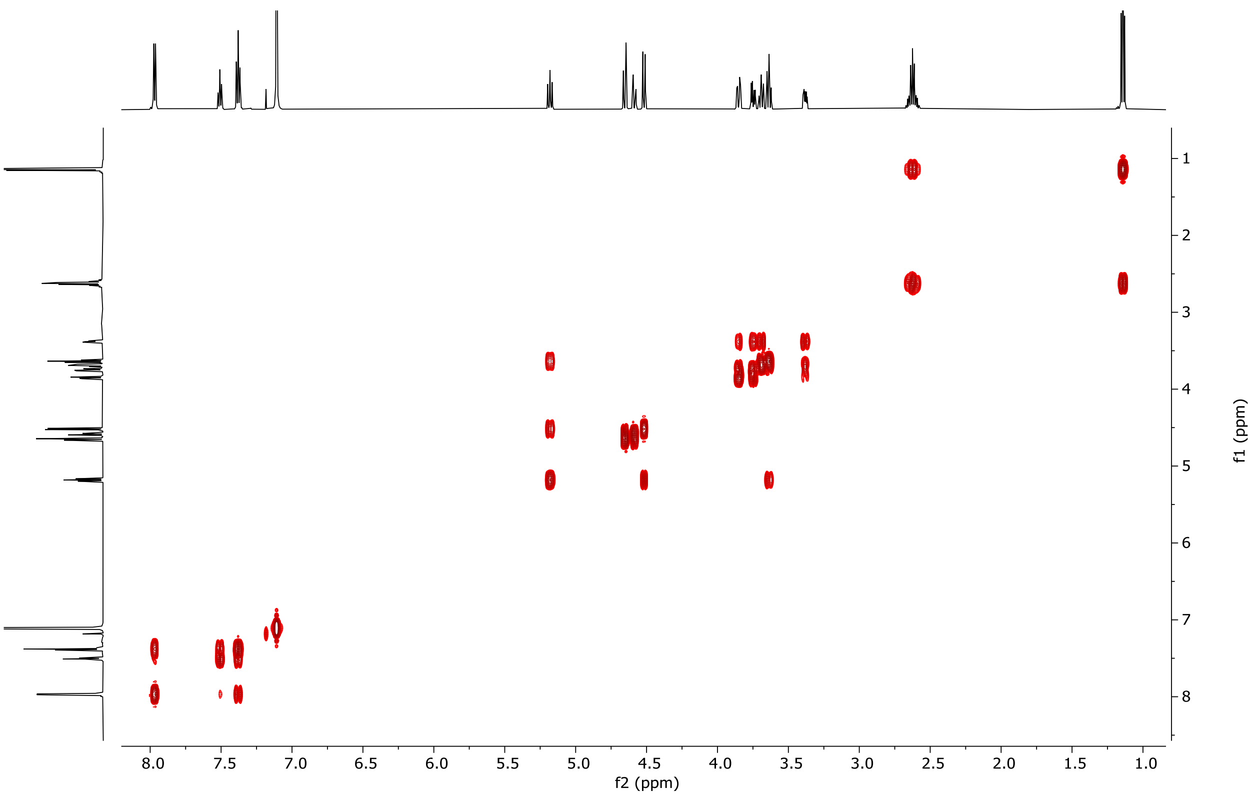

Synthesis of compound 5

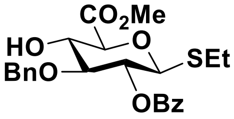

Compound **4** (9.0 g, 21.5 mmol) was dissolved in a mixture of DCM/ ^{t}BuOH /H_2O (4:4:1, 90 mL). TEMPO (1.0 g, 6.4 mmol) and BAIB (17.3 g, 53.8 mmol) were added, and the reaction was stirred at room temperature for six hours. The reaction mixture was neutralized with 1 M HCl, diluted with EtOAc, washed with H_2O, and dried over Na_2SO_4. After concentration, the residue was dissolved in anhydrous DMF (90 mL), and methyl iodide (MeI, 2.6 mL, 43.0 mmol) and potassium carbonate (K_2CO_3, 8.6 g, 62.4 mmol) were added. The reaction was stirred at room temperature overnight, (monitored by TLC). then diluted with EtOAc, and washed sequentially with $NaHCO_3$ and brine. The organic layer was dried over Na_2SO_4, concentrated under reduced pressure, and purified by silica gel column chromatography (Hexane/EtOAc) to afford **5** (6.5 g, 68%) as a white solid.

^{1}H NMR (400 MHz, $CDCl_3$): δ 8.08 – 8.00 (m, 2H), 7.66 – 7.57 (m, 1H), 7.52 – 7.43 (m, 2H), 7.24 – 7.11 (m, 5H), 5.31 (dd, *J* = 10.1, 9.1 Hz, 1H), 4.84 – 4.72 (m, 2H), 4.63 (d, *J* = 10.0 Hz, 1H), 4.07 (dd, *J* = 9.8, 8.8 Hz, 1H), 3.97 (d, *J* = 9.8 Hz, 1H), 3.85 (s, 3H), 3.76 (t, *J* = 8.9 Hz, 1H), 2.84 – 2.65 (m, 2H), 1.25 (t, *J* = 7.4 Hz, 3H). **^{13}C NMR** (101 MHz, $CDCl_3$): δ 169.48, 165.28, 137.79, 133.39, 129.95, 129.69, 128.52, 128.42, 128.15, 127.86, 84.42, 82.33, 78.03, 74.84, 72.08, 71.43, 52.96, 24.28, 14.84. **HRMS QTOF-MS**: calcd. $C_{23}H_{26}NaO_7S$ for $[M+Na]^+$ 469.1297, found 469.1285.

^{1}H NMR of compound 5 (400 MHz, $CDCl_3$)

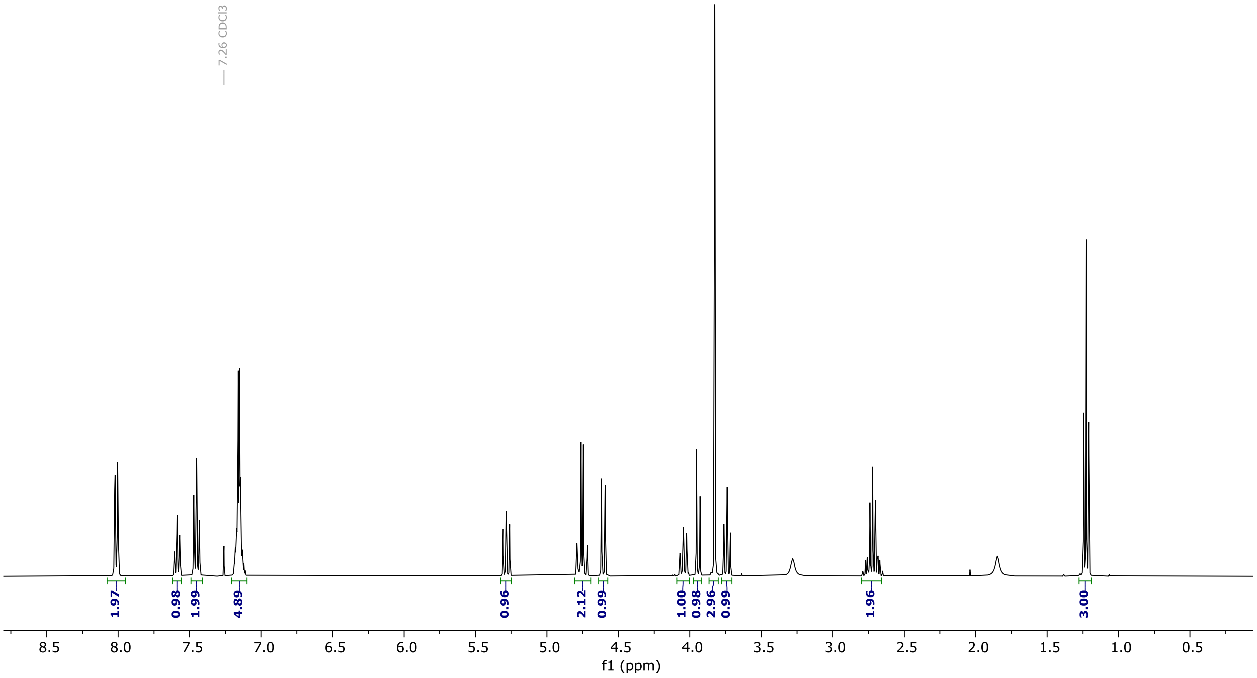

^{13}C NMR of compound 5 (101 MHz, $CDCl_3$)

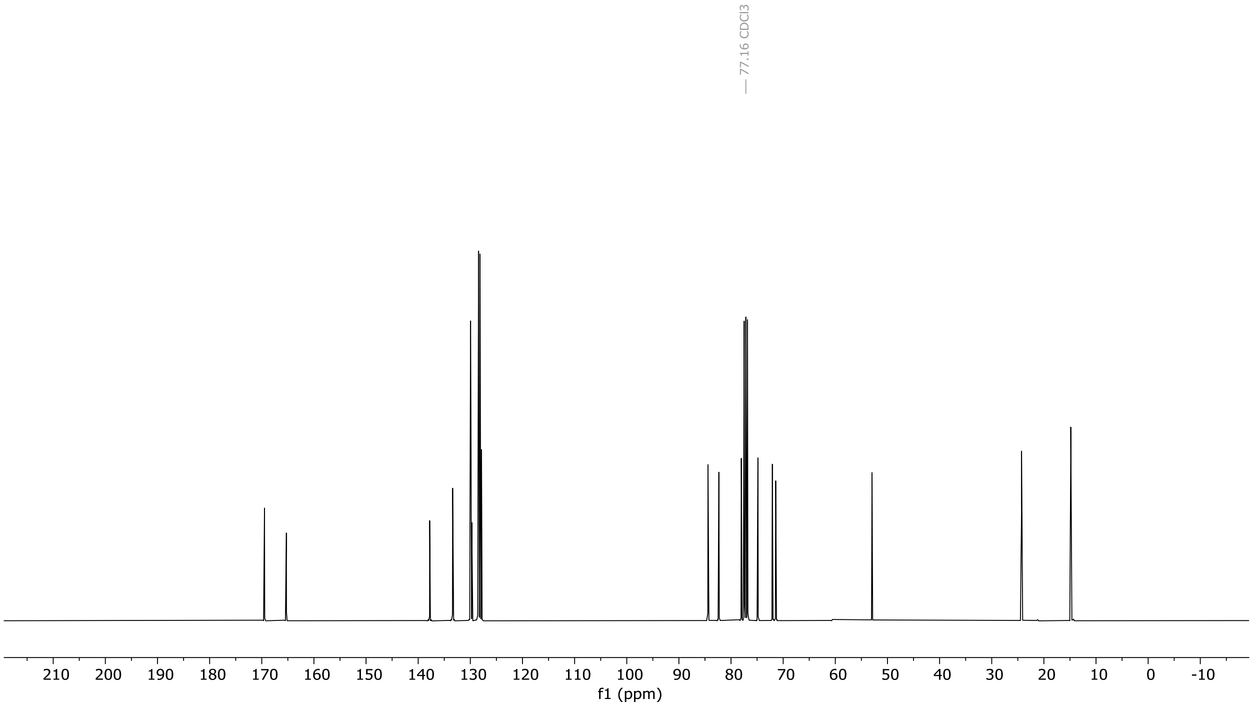

DEPT-135 NMR of compound 5 (101 MHz, $CDCl_3$)

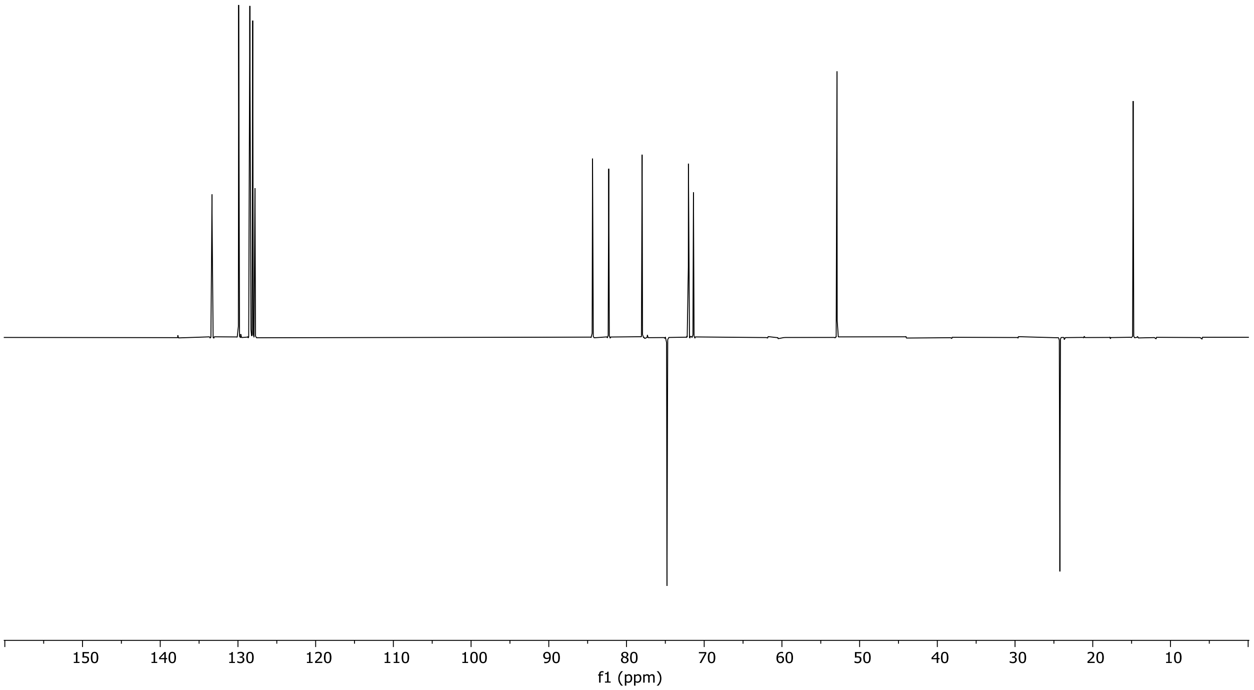

^{1}H-^{13}C HSQC NMR of compound 5 (600 MHz, $CDCl_3$)

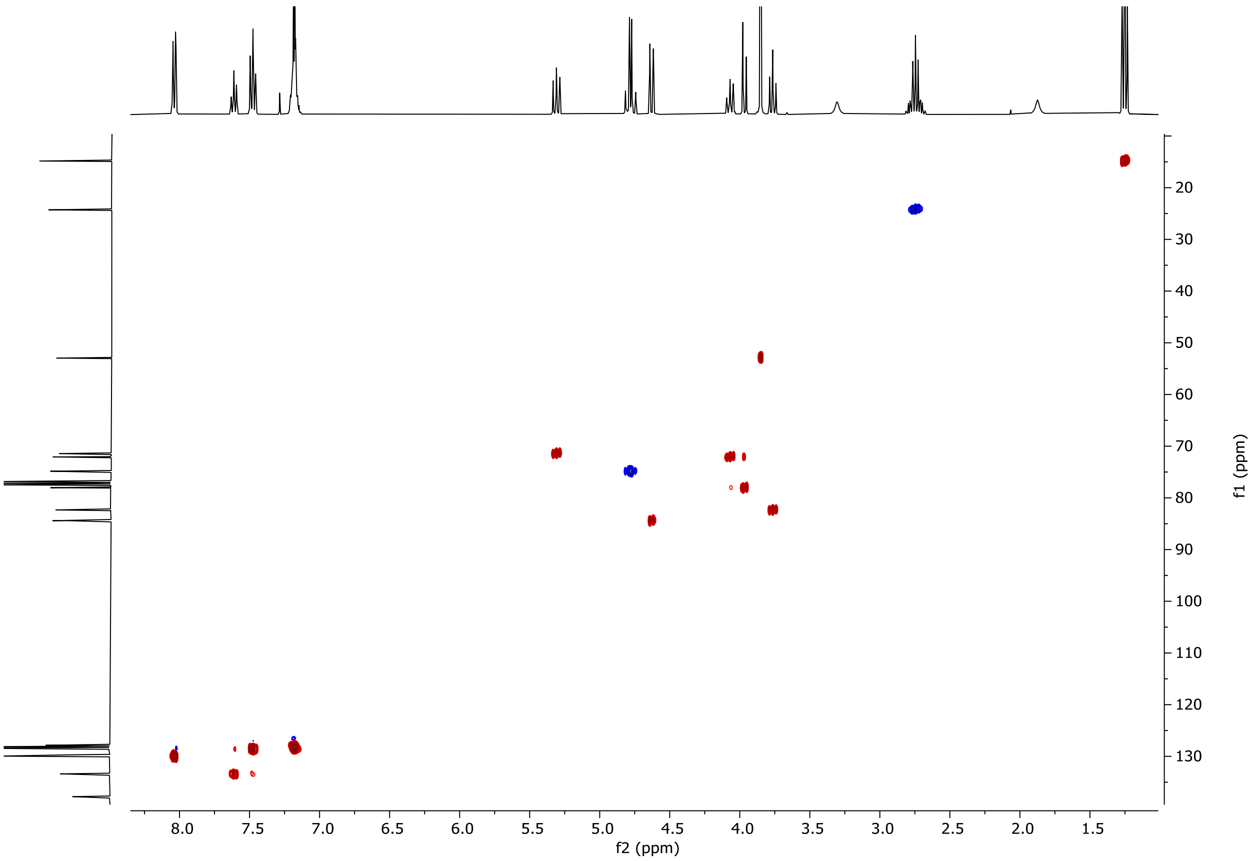

^1H-^1H COSY NMR of compound 5 (600 MHz, $CDCl_3$)

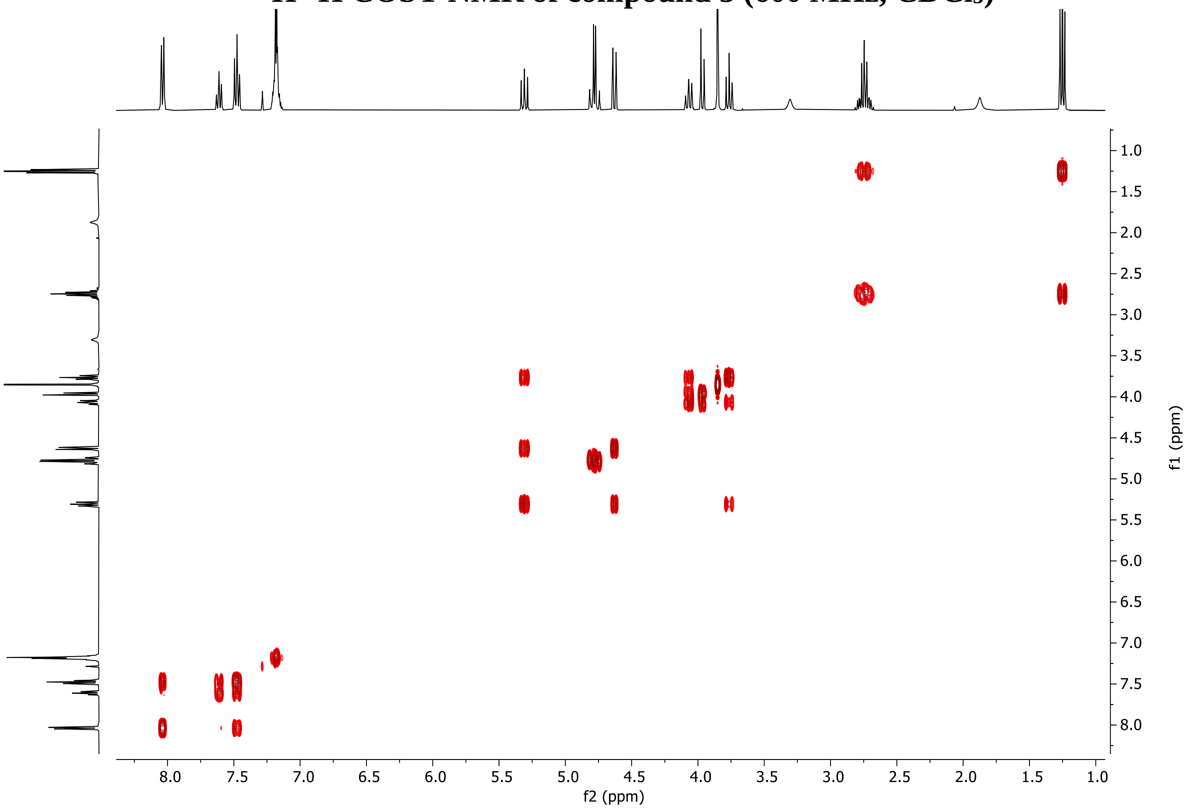

Synthesis of compound 6

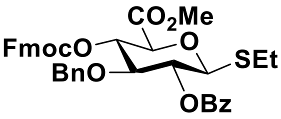

Compound **5** (6.5 g, 14.5 mmol) was dissolved in DCM (70 mL), and 9-fluorenylmethoxycarbonyl (FmocCl, 5.6 g, 21.8 mmol) and pyridine (3.5 mL, 43.7 mmol) were added. The reaction mixture was stirred at room temperature. until completion (monitored by TLC)., the reaction was diluted with EtOAc, washed sequentially with 1 M HCl and saturated brine, and dried over Na_2SO_4. After concentration under reduced pressure, the residue was purified by silica gel chromatography (Hexane/EtOAc) to afford **6** (8.7 g, 90%).

^{1}H NMR (400 MHz, $CDCl_3$): δ 8.03 (dd, *J* = 8.4, 1.3 Hz, 2H), 7.82 – 7.75 (m, 2H), 7.69 – 7.59 (m, 3H), 7.49 (t, *J* = 7.8 Hz, 2H), 7.46 – 7.39 (m, 2H), 7.36 – 7.30 (m, 2H), 7.17 – 7.06 (m, 5H), 5.47 – 5.37 (m, 1H), 5.19 (t, *J* = 9.6 Hz, 1H), 4.71 – 4.57 (m, 3H), 4.50 (dd, *J* = 10.5, 7.0 Hz, 1H), 4.38 (dd, *J* = 10.5, 7.5 Hz, 1H), 4.27 (t, *J* = 7.2 Hz, 1H), 4.15 (d, *J* = 10.0 Hz, 1H), 3.98 (t, *J* = 9.0 Hz, 1H), 3.74 (s, 3H), 2.87 – 2.69 (m, 2H), 1.27 (t, *J* = 7.5 Hz, 3H). **^{13}C NMR** (101 MHz, $CDCl_3$): δ 167.34, 164.96, 154.01, 143.33, 143.01, 141.30, 137.12, 133.41, 129.93, 129.49, 128.48, 128.25, 127.98, 127.79, 127.25, 125.18, 125.09, 120.13, 83.92, 80.10, 76.36, 75.20, 74.50, 71.22, 70.42,

52.93, 46.63, 24.02, 14.73. **HRMS QTOF-MS**: calcd. $C_{38}H_{36}NaO_9S$ for $[M+Na]^+$ 691.1978, found 691.1991.

^{1}H NMR of compound 6 (400 MHz, $CDCl_3$)

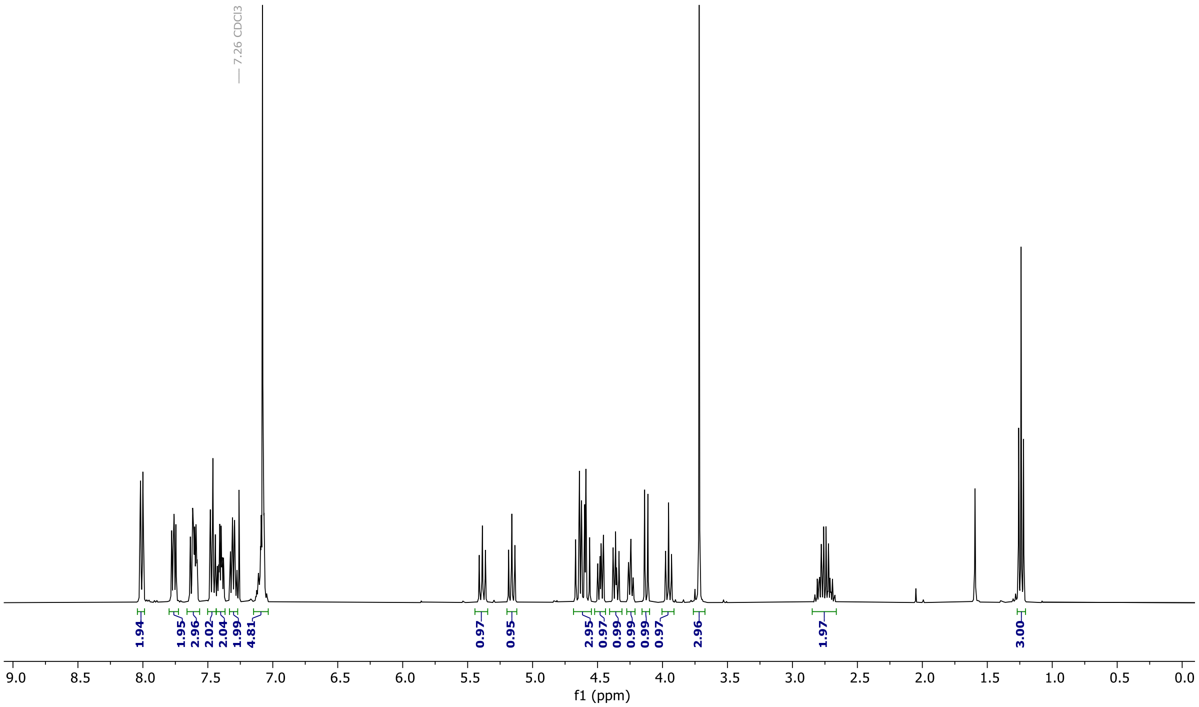

^{13}C NMR of compound 6 (101 MHz, $CDCl_3$)

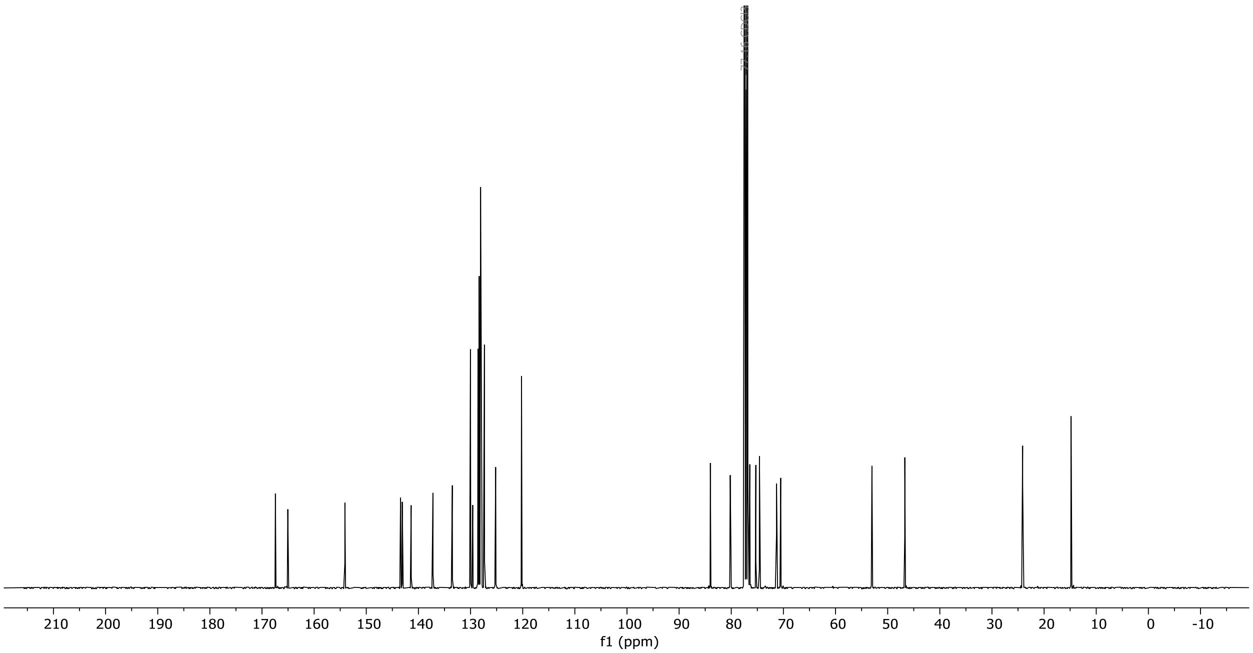

DEPT-135 NMR of compound 6 (101 MHz, $CDCl_3$

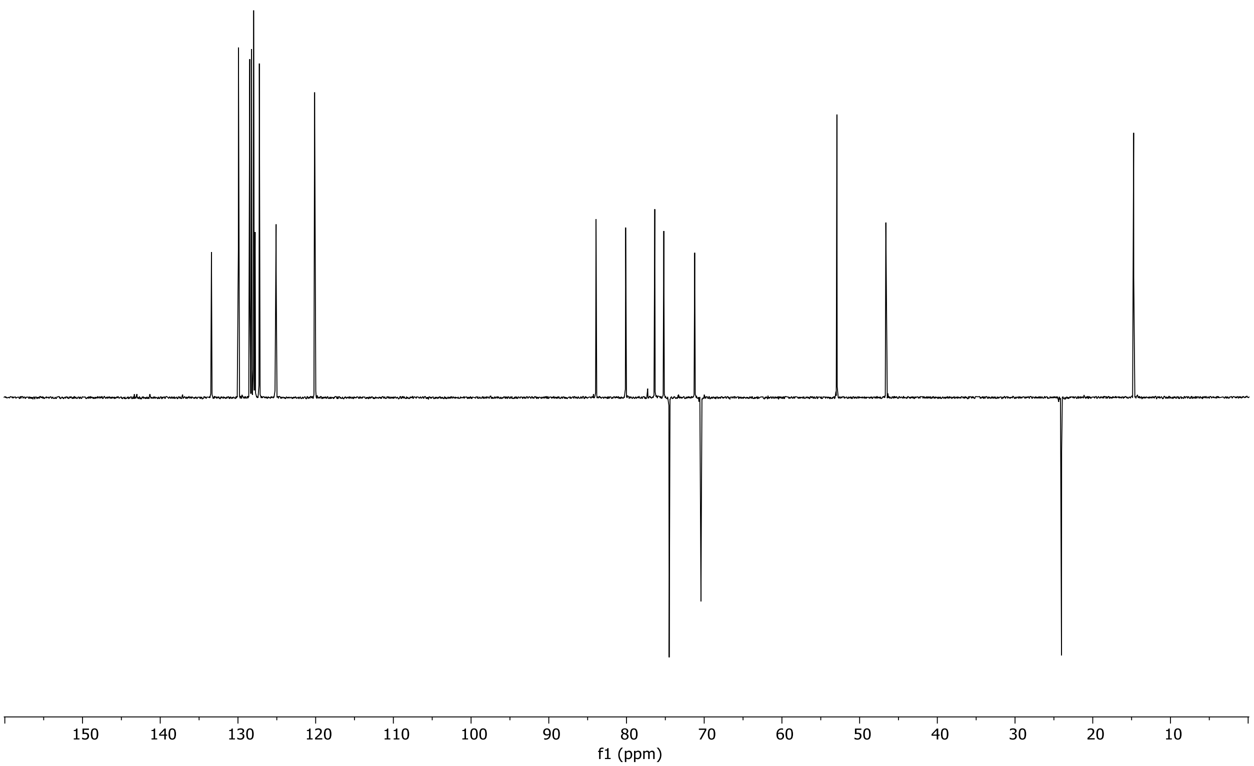

^{1}H-^{13}C HSQC NMR of compound 6 (600 MHz, $CDCl_3$)

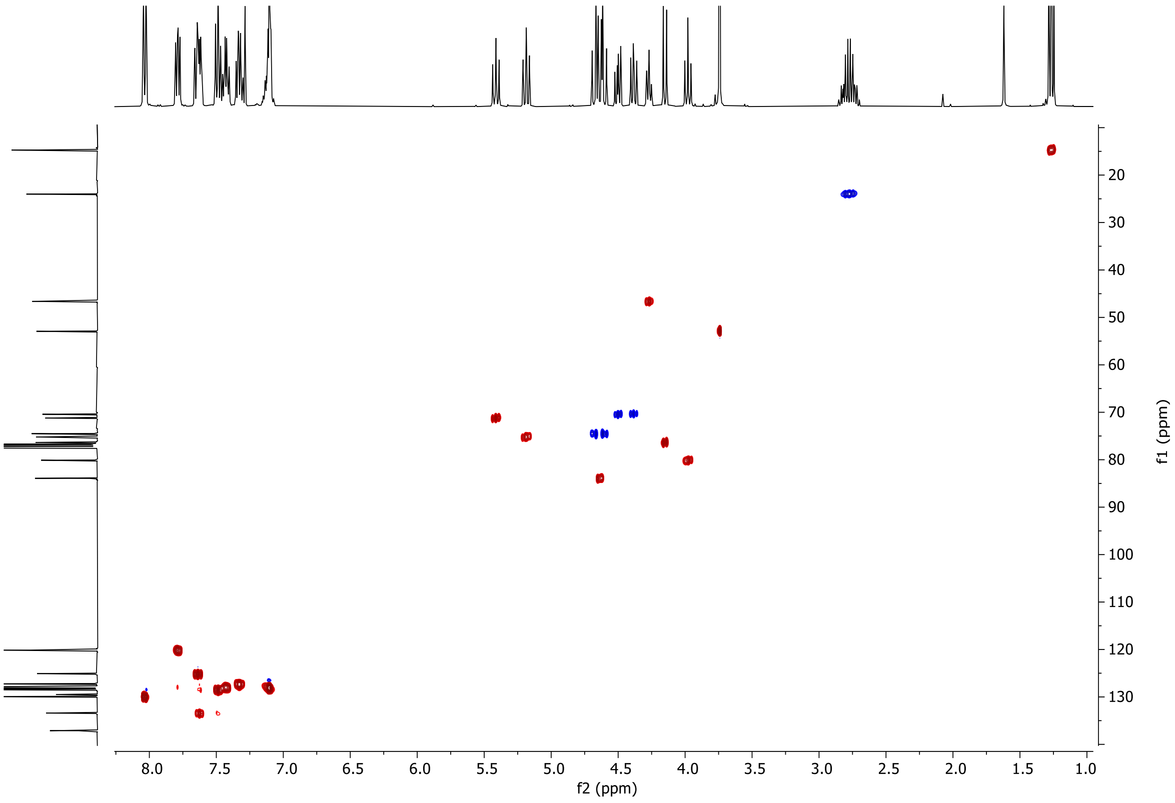

^{1}H-^{1}H COSY NMR of compound 6 (600 MHz, $CDCl_3$)

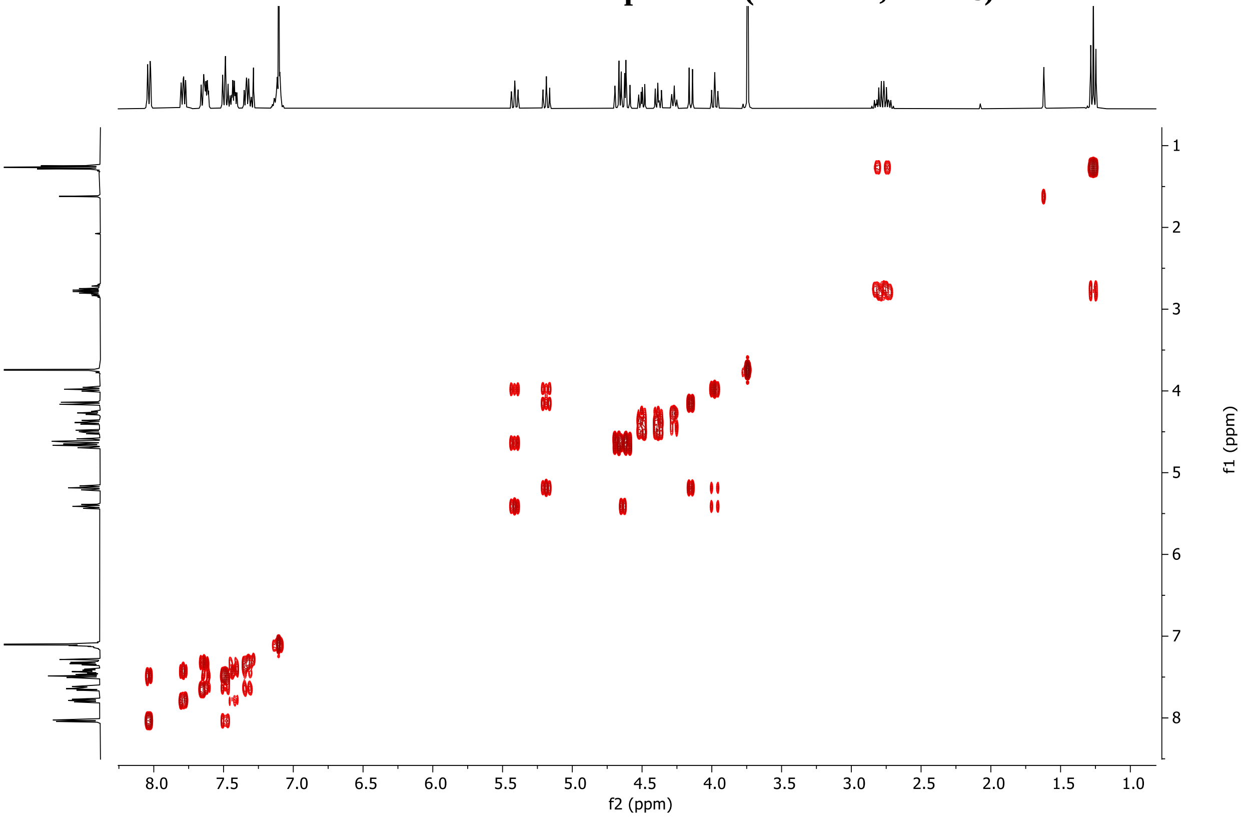

Synthesis of compound 7

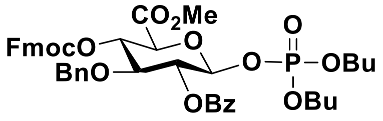

Compound **6** (8.5 g, 12.7 mmol), dibutyl hydrogen phosphate (7.6 mL, 38.1 mmol), and powdered 4 Å molecular sieves (3.0 g, hot gun-dried) were stirred in anhydrous DCM (90 mL) at room temperature for 30 min. *N*-Iodosuccinimide (NIS, 3.4 g, 15.2 mmol) was added in one portion, and the reaction mixture was cooled to -5 °C. After three min, triflic acid (TfOH, 0.1 mL, catalytic amount) was added. The reaction was stirred at -5 °C for one hour (monitored by TLC). The mixture was neutralized with pyridine, diluted with DCM, and filtered to remove the molecular sieves. The filtrate was washed sequentially with saturated $Na_2S_2O_3$, saturated $NaHCO_3$, and brine. The organic layer was dried over Na_2SO_4, concentrated under reduced pressure, and purified by silica gel column chromatography (Hexane/EtOAc) to afford **7** (8.9 g, 86%).

^{1}H NMR (600 MHz, $CDCl_3$): δ 8.02 (d, *J* = 7.4 Hz, 2H), 7.82 – 7.76 (m, 2H), 7.67 – 7.59 (m, 3H), 7.48 (t, *J* = 7.6 Hz, 2H), 7.45 – 7.36 (m, 2H), 7.32 (t, *J* = 7.4 Hz, 2H), 7.16 – 7.03 (m, 5H), 5.43 – 5.34 (m, 2H), 5.32 (d, *J* = 1.9 Hz, 1H), 5.09 (t, *J* = 9.3 Hz, 1H), 4.62 (d, *J* = 11.5 Hz, 1H), 4.56 – 4.49 (m, 2H), 4.46 (dd, *J* = 10.6, 6.9 Hz, 1H), 4.27 (t, *J* = 6.8 Hz, 1H), 4.22 (d, *J* = 9.9 Hz, 1H), 4.02 (p, *J* = 6.6 Hz, 2H), 3.96 (t, *J* = 9.0 Hz, 1H), 3.80 – 3.72 (m, 1H), 3.69 (m, 4H), 1.65 – 1.58 (m, 3H), 1.40 – 1.25 (m, 4H), 1.03 (h, *J* = 7.4 Hz, 2H), 0.90 (t, *J* = 7.4 Hz, 3H), 0.70 (t, *J* = 7.4 Hz, 3H). **^{13}C NMR** (151 MHz, $CDCl_3$): δ 166.76, 164.75, 154.03, 143.32, 143.13, 141.31, 137.09, 133.58, 129.84, 129.13, 128.55, 128.22, 127.82, 127.22, 124.94, 120.09, 120.07, 96.15, 78.46, 74.83, 74.69, 72.57, 72.46, 72.40, 70.25, 68.21, 68.17, 67.99, 67.95, 52.76, 46.69, 32.02, 31.97, 31.80, 31.75, 18.53, 18.25, 13.30, 13.13. **HRMS QTOF-MS**: calcd. $C_{44}H_{49}NaO_{13}P$ for $[M+Na]^+$ 839.2808, found 839.2847.

^{1}H NMR of compound 7 (400 MHz, $CDCl_3$)

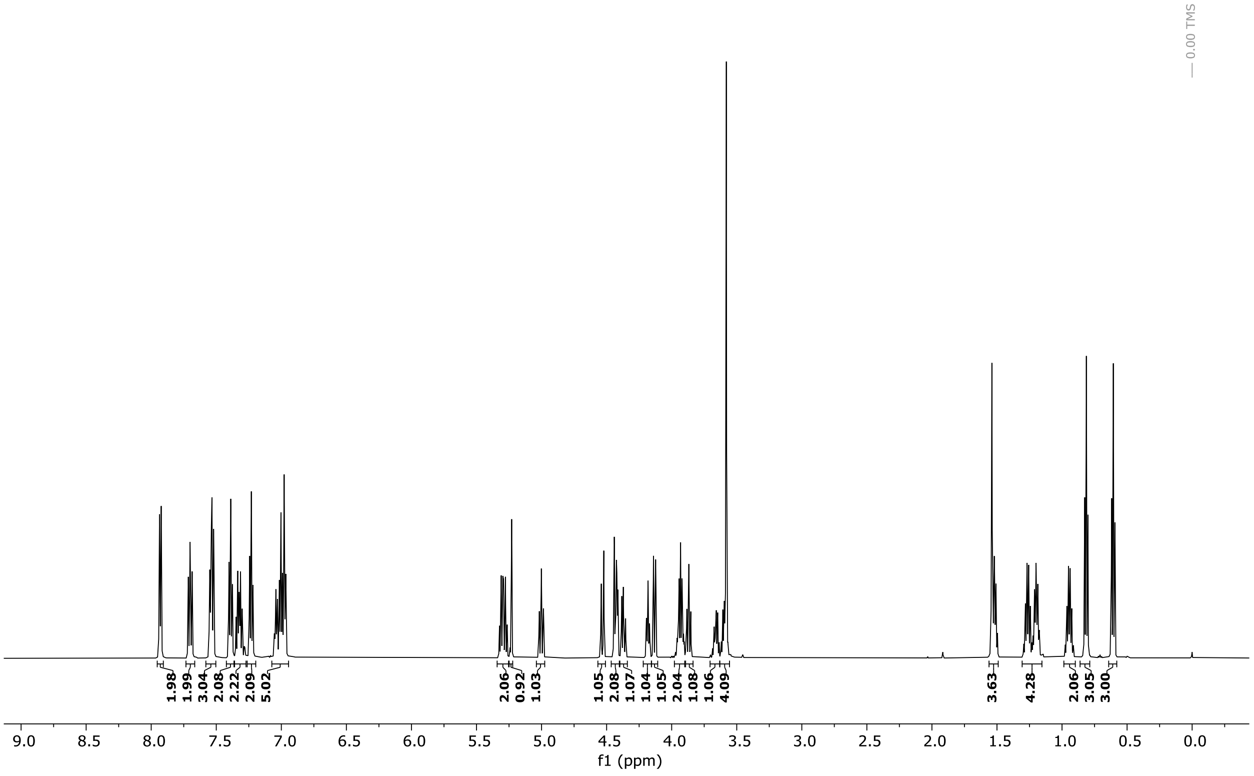

^{13}C NMR of compound 7 (101 MHz, $CDCl_3$)

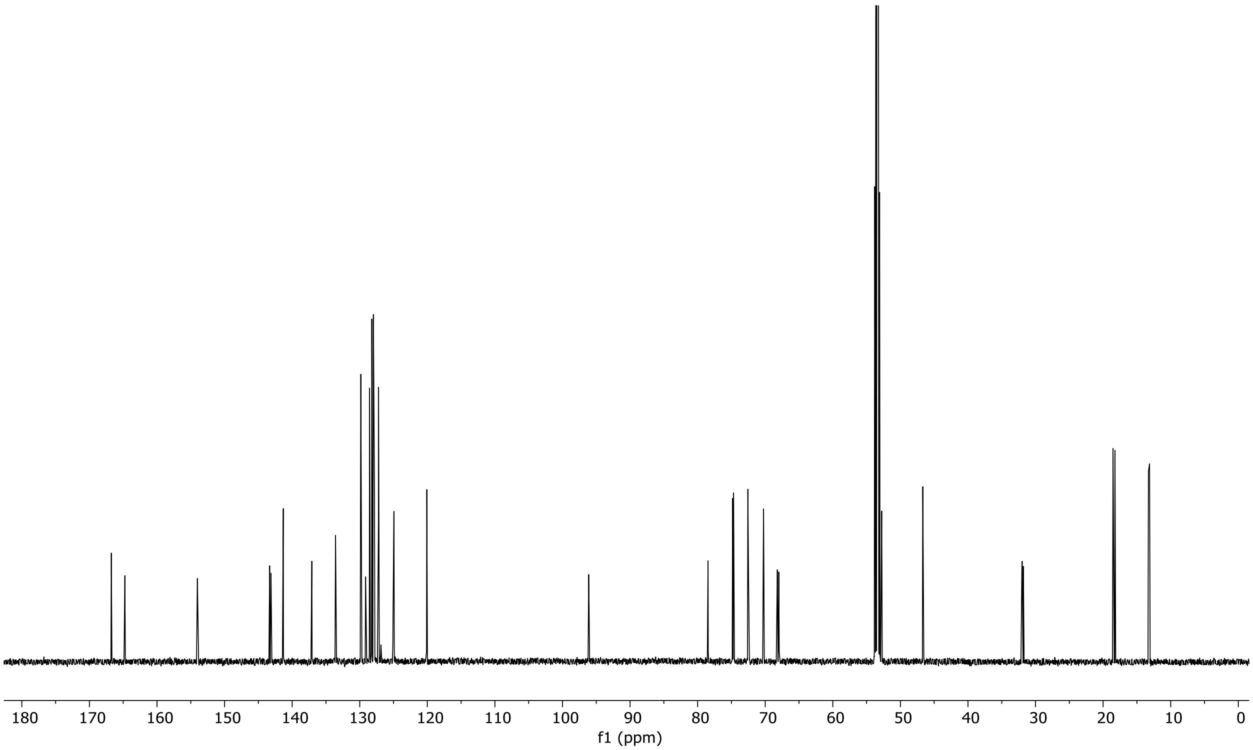

DEPT-135 NMR of compound 7 (101 MHz, $CDCl_3$)

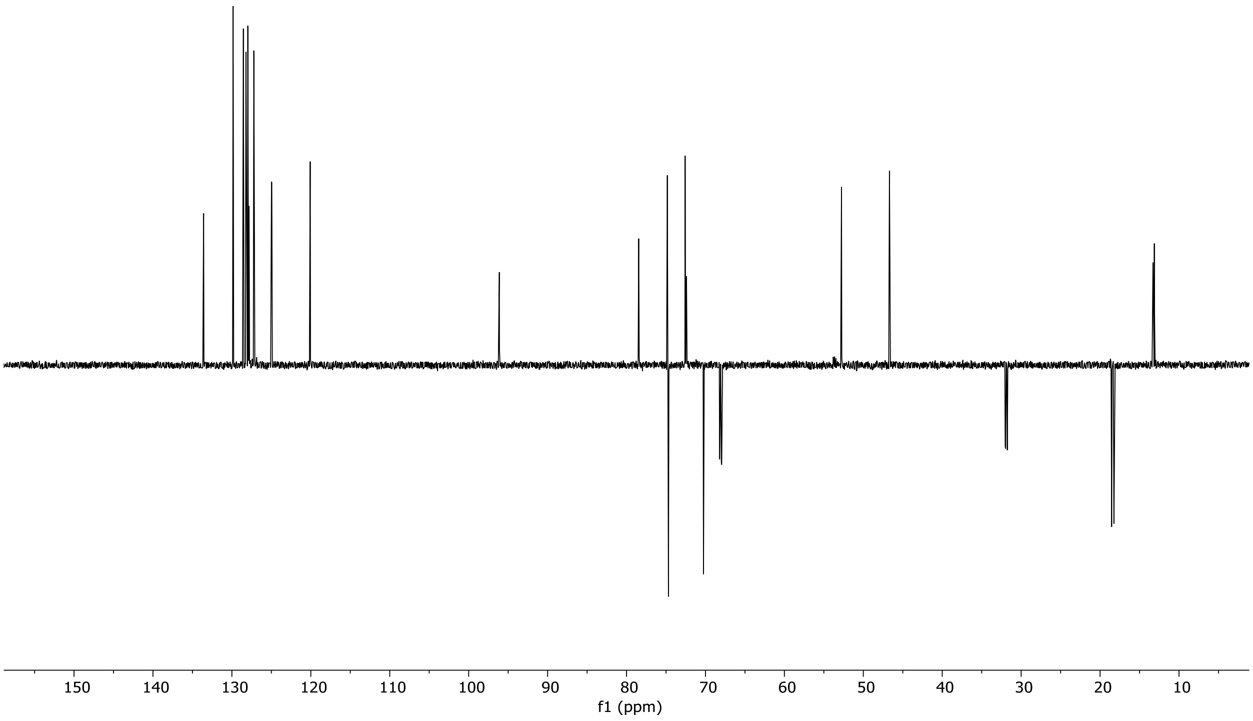

^{1}H-^{13}C HSQC NMR of compound 7 (600 MHz, $CDCl_3$)

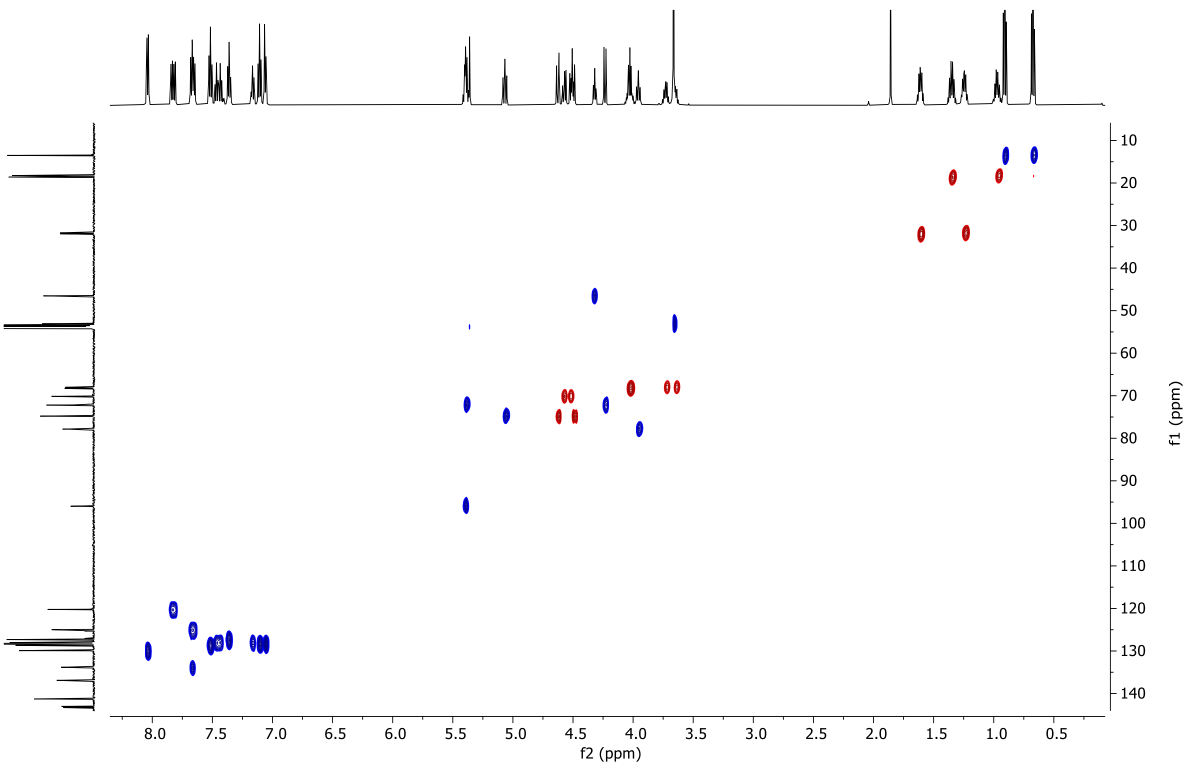

^{1}H-^{1}H COSY NMR of compound 7 (600 MHz, $CDCl_3$)

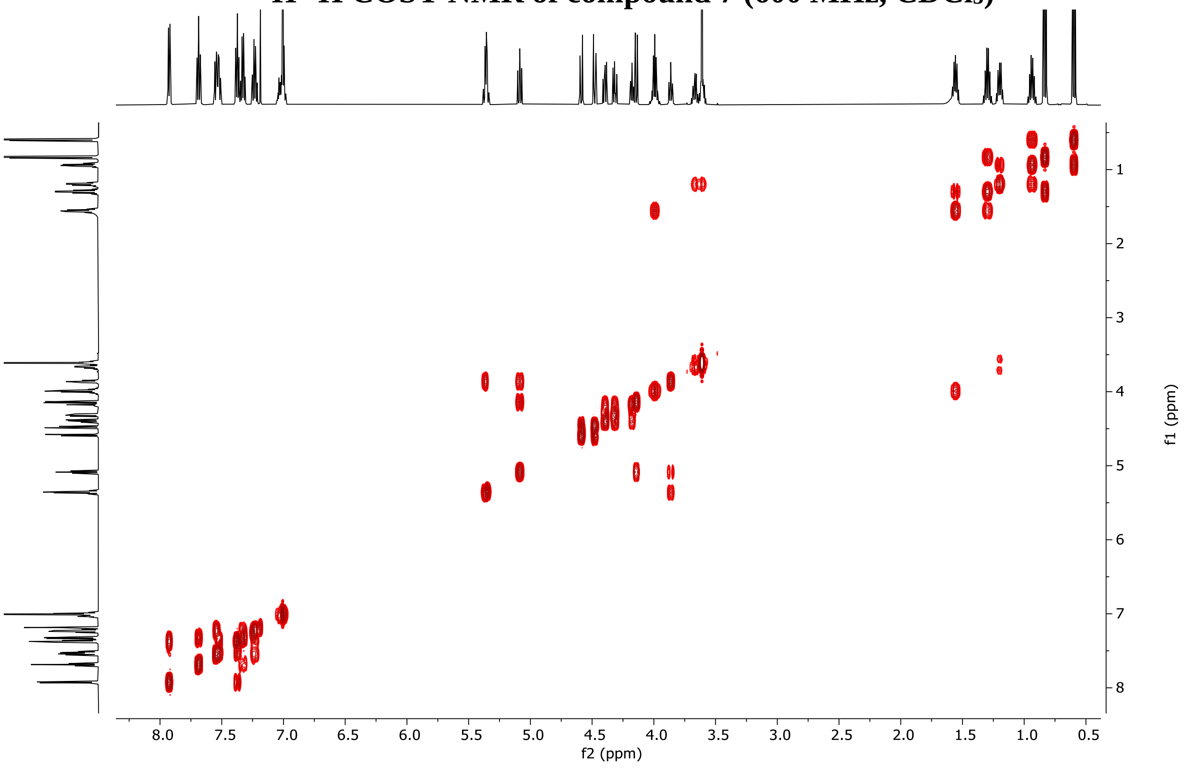

Synthesis of compound 8

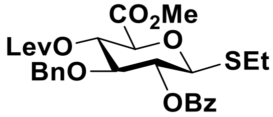

Compound **7** (4 g, 9 mmol) was dissolved in anhydrous DCM (80 mL), levulinic acid (1.82 mL, 18.0 mmol), N, N-dicycohexylcarbodiimide (1.4 g, 10.0 mmol) and catalytic amount of 4-dimethylaminopyridine (0.54 g, 4.4 mmol) were added. The reaction mixture was stirred at room temperature for 2 h. The reaction mixture was filtered through celite and washed with saturated $NaHCO_3$ and brine solution respectively. The organic layer was dried over Na_2SO_4, concentrated under reduced pressure, and purified by silica gel column chromatography (Hexane/EtOAc) to afford **8** (4.14 g, 85%).

^{1}H NMR (700 MHz, $CDCl_3$) δ 8.03 (dd, J = 8.0, 1.6 Hz, 2H), 7.62 (td, J = 7.5, 1.3 Hz, 1H), 7.48 (t, J = 7.6 Hz, 2H), 7.17 (h, J = 3.8 Hz, 5H), 5.40 (t, J = 9.5 Hz, 1H), 5.31 (t, J = 9.6 Hz, 1H), 4.69 – 4.60 (m, 3H), 4.05 (d, J = 9.9 Hz, 1H), 3.94 (t, J = 9.1 Hz, 1H), 3.77 (s, 3H), 2.84 – 2.68 (m, 4H), 2.58 (dt, J = 17.4, 6.5 Hz, 1H), 2.49 (dt, J = 17.3, 6.6 Hz, 1H), 2.20 (s, 3H), 1.26 (t, J = 7.4 Hz, 3H). **^{13}C NMR (176 MHz, $CDCl_3$)** δ 206.09, 171.37, 167.50, 164.97, 137.45, 133.48, 129.90, 129.54, 128.48, 128.28, 127.99, 127.76, 83.77, 80.41, 76.68, 74.34, 71.40, 71.33, 52.95, 37.70, 29.87, 27.72, 23.93, 14.72. **HRMS QTOF-MS**: calcd. $C_{28}H_{32}O_9S$ for $[M+Na]^+$ 544.1768, found 544.1767.

^{1}H NMR of compound 8 (700 MHz, $CDCl_3$)

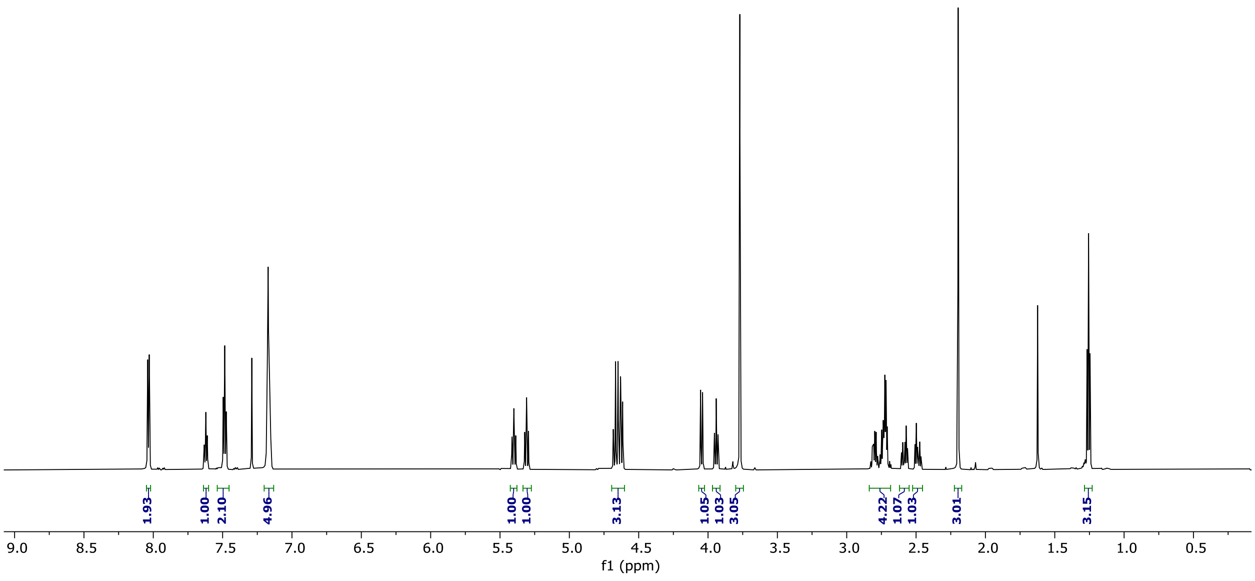

^{13}C NMR of compound 8 (176 MHz, $CDCl_3$)

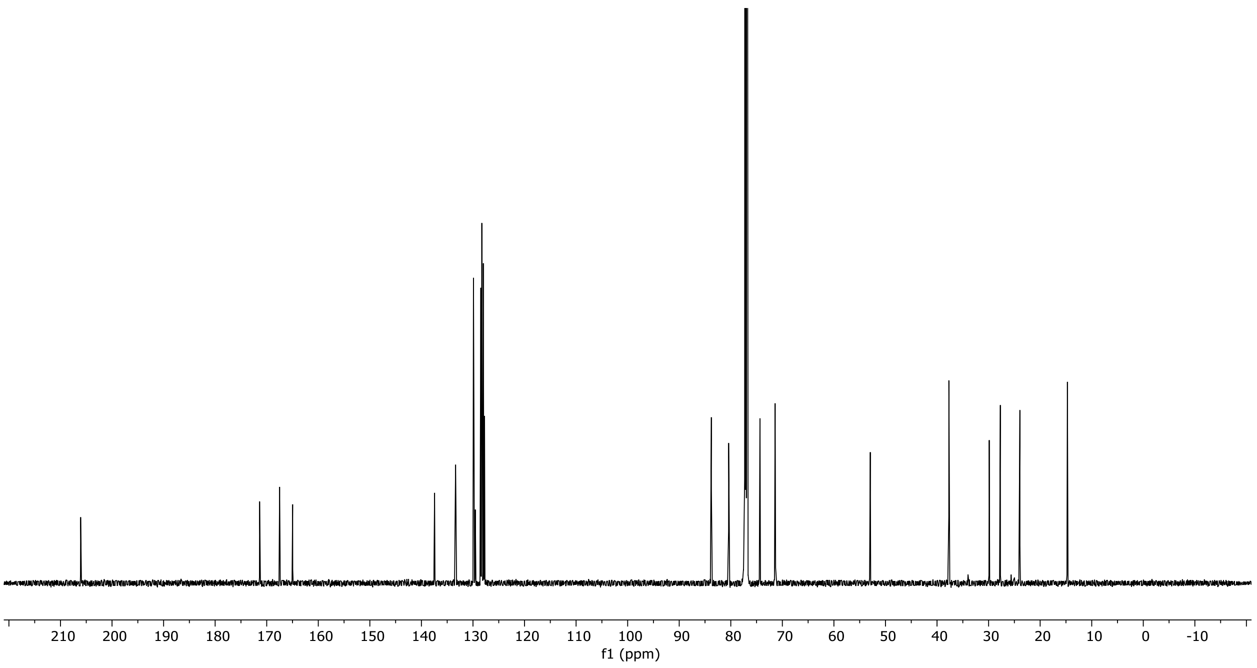

^{1}H-^{13}C HSQC NMR of compound 8 (700 MHz, $CDCl_3$)

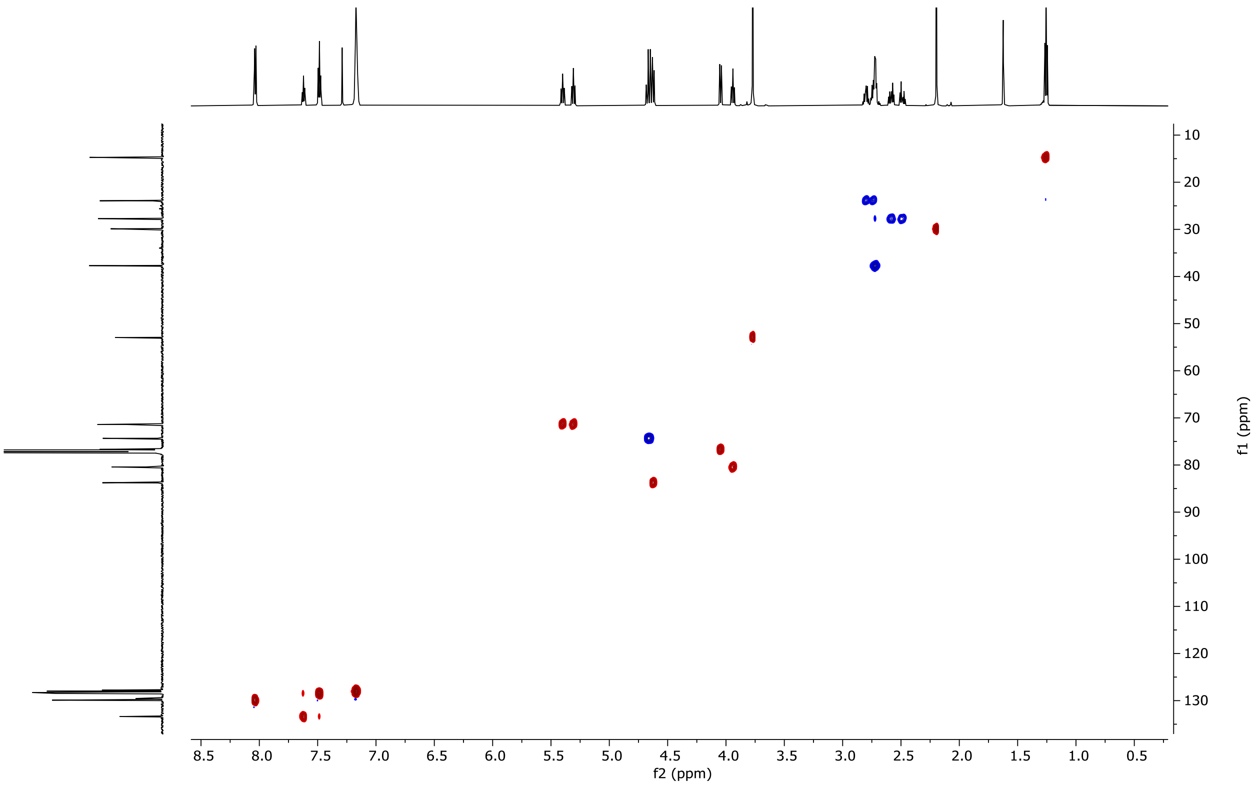

^{1}H-^{1}H COSY NMR of compound 8 (700 MHz, $CDCl_3$)

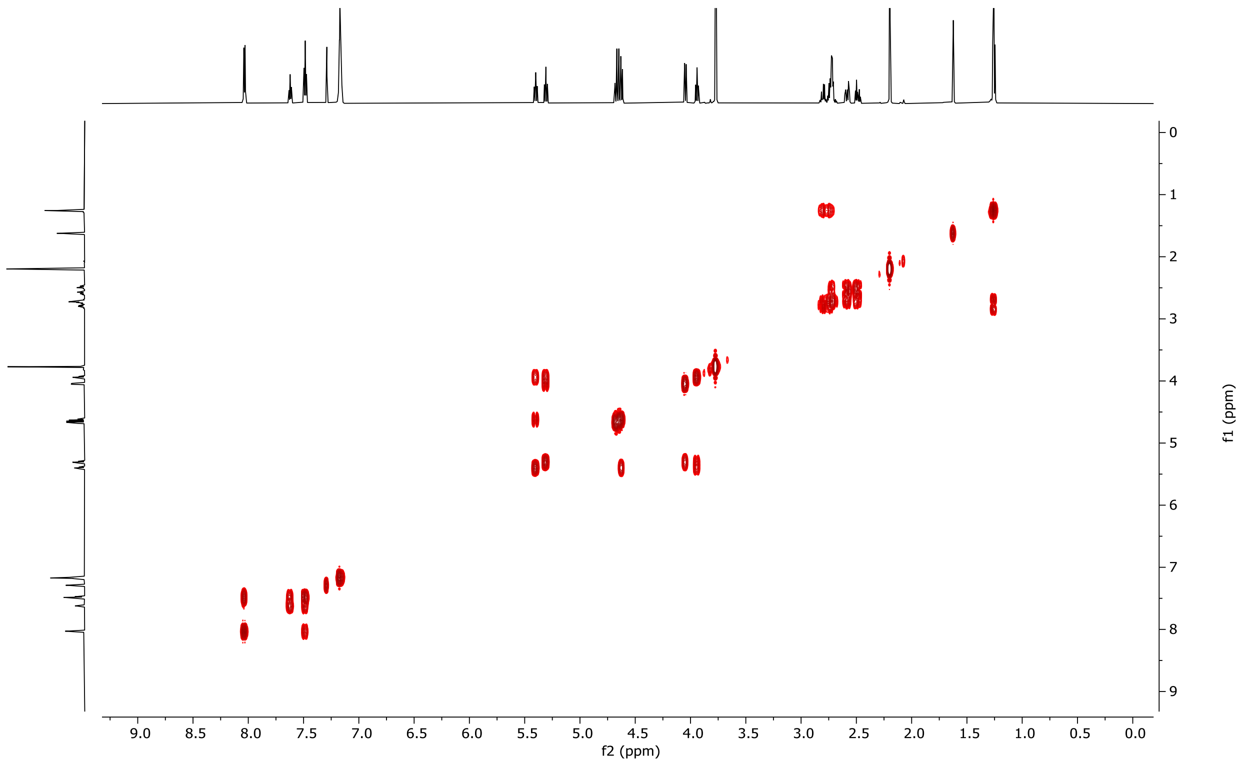

Synthesis of compound 9

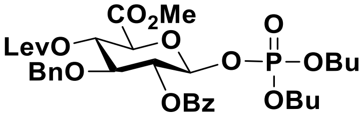

Compound **8** (3 g, 5.5 mmol), dibutyl hydrogen phosphate (3.2 mL, 16.5 mmol), and powdered 4 Å molecular sieves (3.0 g, hot gun-dried) were stirred in anhydrous DCM (90 mL) at room temperature for 30 min. *N*-Iodosuccinimide (NIS, 1.48 g, 6.6 mmol) was added in one portion, and the reaction mixture was cooled to -5 °C. After three min, triflic acid (TfOH, 0.1 mL, catalytic amount) was added. The reaction was stirred at -5 °C for one hour (monitored by TLC). The mixture was neutralized with pyridine, diluted with DCM, and filtered to remove the molecular sieves. The filtrate was washed sequentially with saturated $Na_2S_2O_3$, saturated $NaHCO_3$, and brine.

The organic layer was dried over Na_2SO_4, concentrated under reduced pressure, and purified by silica gel column chromatography (Hexane/EtOAc) to afford **9** (3.3 g, 86%).

^{1}H NMR (400 MHz, $CDCl_3$): δ 8.05 – 7.98 (m, 2H), 7.65 – 7.56 (m, 1H), 7.46 (t, *J* = 7.8 Hz, 2H), 5.50 – 5.38 (m, 2H), 5.30 (dd, *J* = 9.9, 9.1 Hz, 1H), 4.68 (d, 2H), 4.18 – 3.99 (m, 3H), 3.97 – 3.86 (m, 1H), 3.74 (s, 5H), 2.71 (t, *J* = 6.8 Hz, 2H), 2.67 – 2.43 (m, 2H), 2.18 (s, 3H), 1.69 – 1.57 (m, 2H), 1.44 – 1.21 (m, 4H), 1.08 – 0.94 (m, 2H), 0.91 (t, *J* = 7.4 Hz, 3H), 0.67 (t, *J* = 7.4 Hz, 3H). **^{13}C NMR** (101 MHz, $CDCl_3$): δ 206.04, 171.50, 166.95, 164.82, 137.23, 133.61, 129.96, 129.16, 128.57, 128.38, 128.17, 127.92, 96.25, 96.20, 78.59, 74.37, 73.01, 72.50, 72.42, 71.15, 68.34, 68.27, 68.10, 68.04, 52.99, 37.66, 32.06, 31.99, 31.80, 31.73, 29.92, 27.75, 18.60, 18.26, 13.62, 13.42. **HRMS QTOF-MS**: calcd. $C_{34}H_{45}NaO_{13}P$ for $[M+Na]^+$ 715.2495, found 715.2440

^{1}H NMR of compound 9 (400 MHz, $CDCl_3$)

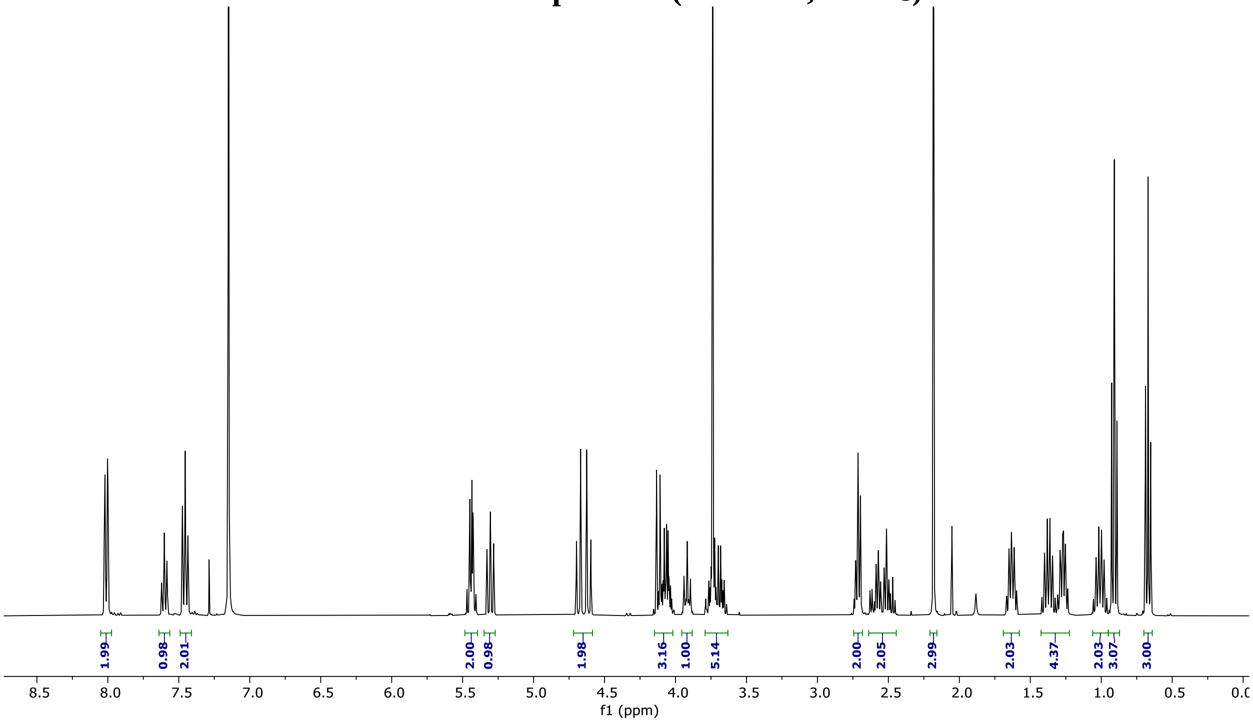

^{13}C NMR of compound 9 (101 MHz, $CDCl_3$)

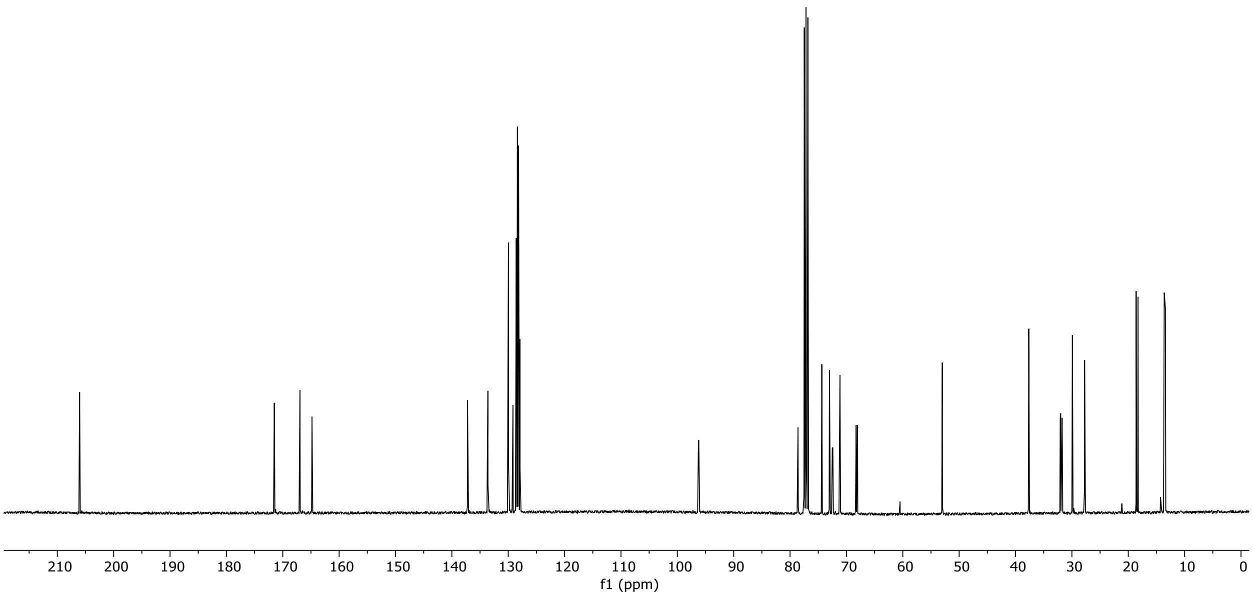

^1H-^{13}C HSQC NMR of compound 9 (400 MHz, $CDCl_3$)

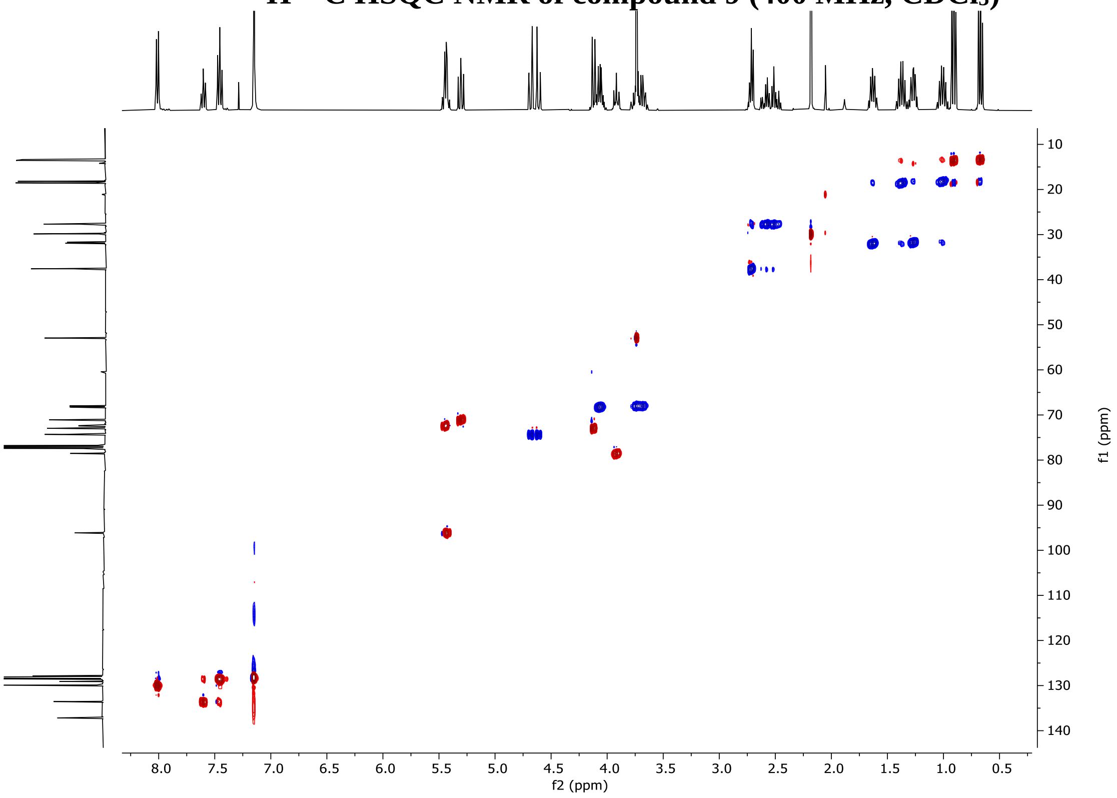

^1H-^1H COSY NMR of compound 9 (400 MHz, $CDCl_3$)

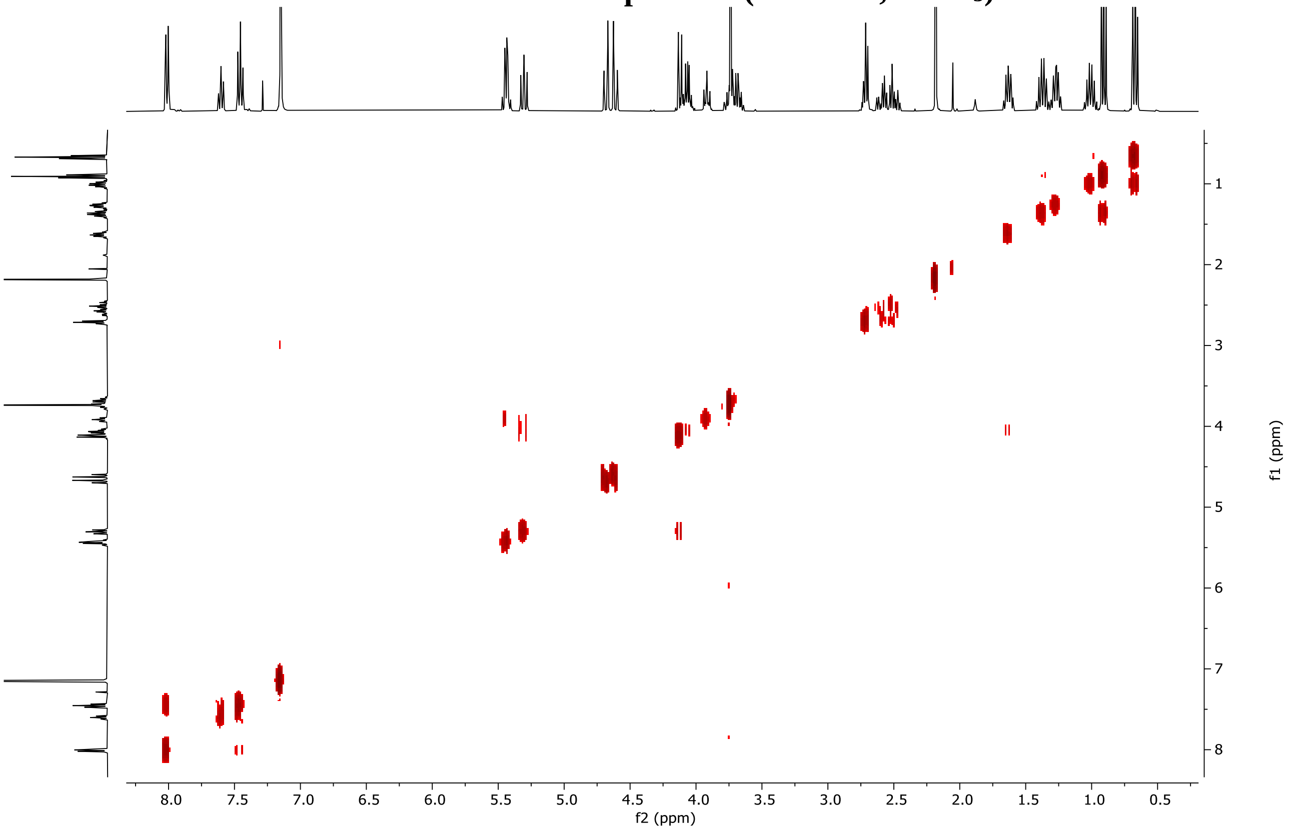

3 Automated Glycan Assembly

3.1 General materials and methods

The automated syntheses were performed using a custom-built synthesizer developed at the Max Planck Institute of Colloids and Interfaces. All solvents used were HPLC-grade. The solvents used for the building block, activator, TMSOTf, and capping solutions were taken from an anhydrous solvent system (J.C. Meyer), The building blocks were co-evaporated three times with toluene and dried for 1 h on high vacuum before use. Oven-dried, argon-flushed flasks were used to prepare all moisture-sensitive solutions. Activator, capping, deprotection, acidic wash, and building block solutions were freshly prepared and kept under argon during the automation run. All yields of products obtained by AGA were calculated based on resin loading. Resin loading was determined following previously established procedures[2].

3.2 Preparation of stock solutions

- **Building block solution**: Between 0.1 mmol of building block were dissolved in anhydrous DCM (1 mL per glycosylation).
- **NIS/TfOH activator solution A**: 1.35 g (6.0 mmol) of recrystallized NIS was dissolved in 40 mL of a 2:1 v/v mixture of anhydrous DCM and anhydrous dioxane. Then triflic acid (55 μL, 0.6 mmol) was added. The solution was kept at 0 °C (ice bath) for the duration of the automation run.
- **TMSOTf solution**: TMSOTf (0.45 mL, 2.49 mmol) was added to anhydrous DCM (40 mL) for glycosyl phosphate activation.
- **Lev deprotection solution**: Hydrazine acetate (550 mg, 5.97 mmol) was dissolved in pyridine/AcOH/H_2O (40mL, v/v, 32:8:2) and sonicated for 10 min
- **Fmoc deprotection solution**: A solution of 20% piperidine in DMF (v/v) was prepared.
- **Capping solution**: A solution of 10% acetic anhydride and 2% methane sulfonic acid in anhydrous DCM (v/v) was prepared.

Optimized glycosylation protocols

Donor	Temp (t_1) / Time (T_1)	Temp (t_2) / Time (T_2)	Observations
SEt (**BB-6**, **BB-8**) Module-C	-35 °C, 30 min	-10 °C, 30 min	No reaction
	-20 °C, 30 min	0 °C, 30 min	No reaction
	0 °C, 30 min	15 °C, 30 min	Less conversion
$PO(OBu)_2$ (**BB-7**, **BB-9**) Module-D	-35 °C, 30 min	-15 °C, 30 min	Delusion Sequences
	-15 °C, 30 min	0 °C, 30 min	Desired Product
	0 °C, 30 min	15 °C, 30 min	Less conversion

Table S2. Glycosylation conditions optimization

3.3 Modules for automated synthesis

3.3.1 **Module A: Resin preparation**

All automated syntheses were performed on a 0.015 mmol scale. Resin L is placed in the reaction vessel and swollen in DCM for 20 min at room temperature prior to the synthesis. During this time, all reagent lines needed for the synthesis are washed and primed. After the swelling, the resin is washed with DMF, THF, and DCM (3 x 2 mL for 25 s)

3.3.2 **Module B: Acidic wash with TMSOTf solution**

The resin was swollen in 2 mL DCM and the temperature of the reaction vessel adjusted to -20 °C. Upon reaching the low temperature, TMSOTf solution (1 mL) was added dropwise to the reaction vessel. After bubbling for 3 min, the acidic solution was drained and the resin washed with 2 mL DCM for 25 s.

Action	Cycles	Solution	Amount	T (°C)	time
Cooling	-	-	-	-15	-
Delivery	1	DCM	2 mL	-15	-
Delivery	1	TMSOTf solution	1 mL	-15	3 min
Wash	1	DCM	2 mL	-15	25 sec

Table S3. Acidic wash module with TMSOTf solution.

3.3.3 **Module C: Thioglycosides glycosylation**

The building block solution (5 equiv. of BB in 1 mL of DCM per glycosylation) was delivered to the reaction vessel. After the set temperature was reached, the reaction was started by dropwise addition of the activator solution (1.0 mL, excess). After completion of the reaction, the solution is drained and the resin was washed with DCM, DCM/Dioxane (1:2, v/v, 2 mL for 20 s), and DCM (2 x 2 mL for 25 s). The temperature of the reaction vessel is increased to25°C for the next module.

Action	Cycles	Solution	Amount	T (°C)	Incubation time
Cooling	-	-	-	-20	-
Delivery	1	BB solution	1 mL	-20	-
Delivery	1	Activator solution A	1 mL	-20	-

Reaction time (t_1, t_2)	1	-	-	-20 to 0	30 min 30 min
Wash	1	DCM	2 mL	0	25 s
Wash	1	DCM/Dioxane	2 mL	0	20 s
Cooling	-	-	-	-20	-
Delivery	1	BB solution	1 mL	-20	-
Delivery	1	Activator solution A	1 mL	-20	-
Reaction time (t_1, t_2)	1	-	-	-20 to 0	30 min 30 min
Wash	1	DCM	2 mL	0	25 s
Wash	1	DCM/Dioxane	2 mL	0	20 s

BB	Equiv.	t_1 (min)	T_1 (°C)	t_2 (min)	T_2 (°C)
**BB-6, BB-8**	5	30	-20	30	0

Table S4. Thioglycosides glycosylation module

3.3.4 Module D: Glycosyl phosphate glycosylation

The building block solution (5 equiv. of BB in 1 mL of DCM per glycosylation) was delivered to the reaction vessel. After the set temperature (T_1) was reached. The reaction was started by dropwise addition of the TMSOTf solution (1.0 mL, stoichiometric). The reaction was performed in two thermal steps. Starting at T_1, the reagents incubate for a time t_1, then the temperature is linearly increased by a rate of 4 °C/min to a T_2, incubating for a t_2 (times and temperature are mentioned in the following table). After completion of the reaction, the solution was drained, and the resin was washed with DCM (6 x 2 mL for 25 sec). The resin was finally washed with DCM (2 x 3 mL for 25 sec).

Action	Cycles	Solution	Amount	T (°C)	Incubation time
Cooling	-	-	-	-15	-

Delivery	1	BB solution	1 mL	-15	-
Delivery	1	TMSOTf solution	1 mL	-15	-
Reaction time (t_1, t_2)	1	-	-	-15<br>to 0	30 min<br>30 min
Wash	1	DCM	2 mL	0	15 s
Cooling	-	-	-	-15	-
Delivery	1	BB solution	1 mL	-15	-
Delivery	1	TMSOTf solution	1 mL	-15	-
Reaction time (t_1, t_2)	1	-	-	-15<br>to 0	30 min<br>30 min
Wash	1	DCM	2 mL	0	25 s
Heating	-	-	-	25	-
Wash	2	DCM	2 mL	>0	25 s

BB	Equiv.	t_1 (min)	T_1 (°C)	t_2 (min)	T_2 (°C)
**BB-7, BB-9**	5	30	-15	30	0

Table S5. Glycosyl phosphate glycosylation module

3.3.5 **Module E: Capping**

The resin was washed with DMF (two times with 2 mL for 25 s) and the temperature of the reaction vessel was adjusted to 25 °C. A pyridine solution (2 ml, 10% in DMF (v/v)) was delivered into the reaction vessel. After 1 min, the reaction solution was drained, and the resin was washed with DCM (3 x 3 mL for 25 sec). Capping solution (2 mL) was delivered into the reaction vessel. After 10 min, the reaction solution was drained, and the capping reaction was repeated once more. Finally, the resin was washed with DCM (3 x 3 mL for 25 sec) and once with DMF for 1 min.

Action	Cycles	Solution	Amount	T (°C)	Incubation time
Wash	3	DCM	2 mL	25	25s
Wash	3	DMF	2 mL	25	25 s
Delivery	1	10% pyridine in DMF	2 mL	25	1 min

Wash	3	DCM	2 mL	25	25 s
Delivery	1	Capping solution	2 mL	25	20 min
Wash	3	DCM	3 mL	25	25 s

Table S6. Capping module

3.3.6 Module F: Lev deprotection

The resin is washed with DCM (three times with 2 mL for 25 s). DCM (1.3 mL) is delivered to the reaction vessel and the temperature of the reaction vessel is adjusted to 30 °C. Lev deprotection solution (2mL) is delivered to the reaction vessel, kept under pulsed Ar bubbling for 30 min. This procedure is repeated twice. The reaction solution is drained and the resin washed with DMF (three times with 3 mL for 25 s) and DCM (five times each with 2 mL for 25 s).

Action	Cycles	Solution	Amount	T (°C)	Incubation time
Wash	3	DMF	2 mL	30	25 s
Deliver	2	Lev depr. solution	2 mL	30	30 min
Wash	1	DMF	2 mL	-	-
Cooling	-	-	-	-15	-
Wash	3	DMF	2 mL	< -15	25 s
Wash	5	DCM	3 mL	< -15	25 s

Table S7. Lev deprotection

3.3.7 Module G: Fmoc deprotection

The resin is washed with DMF (three times with 2 mL for 25 s) and the reaction vessel was adjusted to 25 °C, then it was washed with DMF (3 x 2 mL for 25 s). Fmoc deprotection solution (2 mL) was delivered to the reaction vessel. After 5 min, the reaction solution was drained, and the resin washed with DMF (3 x 3 mL for 25 s) and DCM (5 x 2 mL for 25 s). The temperature of the reaction vessel was decreased to -15 °C for the next module.

Action	Cycles	Solution	Amount	T (°C)	Incubation time
Wash	3	DMF	2 mL	25	15 sec
Delivery	2	Fmoc depr. solution	2 mL	25	5 min
Wash	3	DMF	2 mL		25 s

Wash	5	DCM	2 mL	25	25 s
Cooling	-	-	-	-20	-

Table S8. Fmoc deprotection module

3.4 Post-AGA manipulations

3.4.1 Module H: Cleavage from solid support

The oligosaccharides were cleaved from the solid support using a continuous-flow photoreactor.[7] during this process DCM was utilized as the carrier solvent in to the photoreactor system. The crude residue was then dissolved in 30% EtOAc:Hexane (3 mL) directly loaded to the column YMC-Diol-300-NP column and purified by method L_{1P}.

3.4.2 Module I: Micro-cleavage from solid support

Trace amounts of resin (around 20 beads) were dispersed in DCM (1 mL) and irradiated with an UV lamp (6 W, 356 nm) for 15 min. Acetonitrile was then added to the resin, and the resulting solution was analyzed by MALDI.

3.4.3 Module J: Off-resin hydrolysis

The crude compound obtained from module H was dissolved in 2 mL of a THF/H_2O (1:1) mixture, and 1 M LiOH (1 mL) and 30% H_2O_2 (0.5 mL) was added at 0 °C. The vial was sealed with a septum, and the reaction stirred until completion. Upon completion, the reaction quenched with amberlite IRC120 H^+ resin, after filtration crude was evaporated under high vacuum rotary evaporator.

3.4.4 Module K: Hydrogenolysis

The crude compound obtained from module I was dissolved in 6 mL of a ^{t}BuOH/H_2O (1:1). $Pd(OH)_2/C$ (10-20%$_w$, moistened with water) was added, and the reaction was stirred in a vial equipped with a H_2 balloon. The reaction progress was monitored to avoid undesired side product formation (by ESI-MS negative mode or MALDI). Upon completion, the reaction was filtered (PTFE 0.45 μm 25 mm syringe filter, Fisher scientific) and washed with H_2O, and MeOH (5 mL each). The filtrate was concentrated by lyophilization. Reaction times and solvents are indicated for each synthesis.

3.4.5 **Module L: Purification/Analysis**

Purification of the crude compound obtained from module K was achieved using a method $L_{1D,}$ manual size-exclusion column. The pure compound was analyzed by MS-Q-TOF.

- **Method L_{1P}:** (YMC-Diol-300-NP column, 150 mm x 4.60 mm) flow rate of 1.00 mL/min with hexanes and EtOAc as eluent.
  For **19** [isocratic 20% EtOAc (5 min), linear gradient to 80% EtOAc (30 min), linear gradient to 100% EtOAc (10 min),]. ELSD Detector: 80 °C.
  For **23** [isocratic 20% EtOAc (5 min), linear gradient to 100% EtOAc (30 min), linear gradient to 100% EtOAc (10 min),]. ELSD Detector: 80 °C.

- **Method L_{1D}:** Sephadex® LH-20 column with H_2O/MeOH (1:1) as eluent, isocratic.

4 Oligosaccharides syntheses

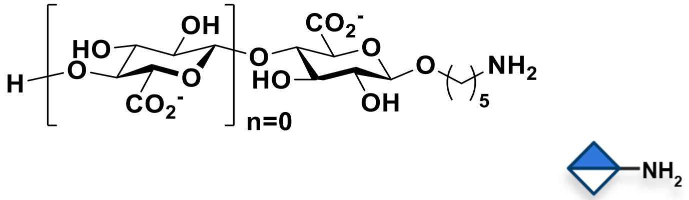

11

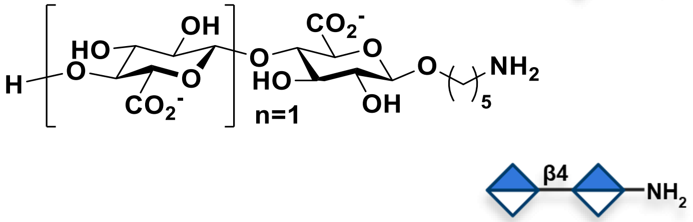

13

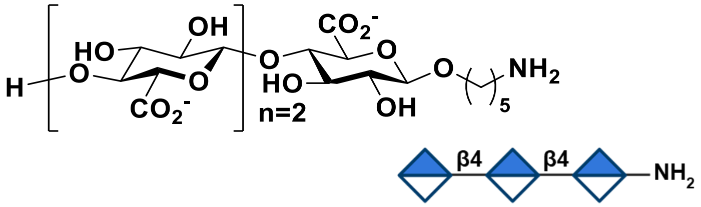

15

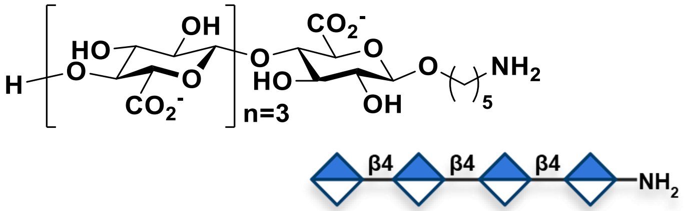

17

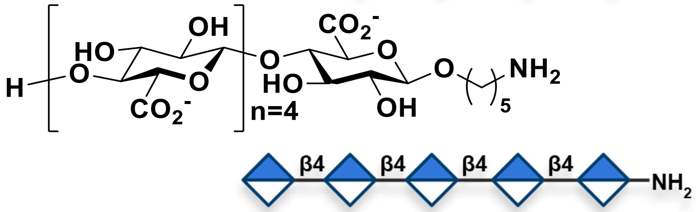

19

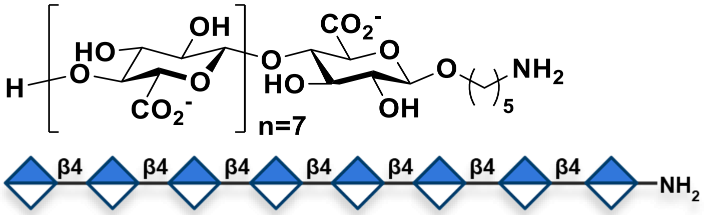

21

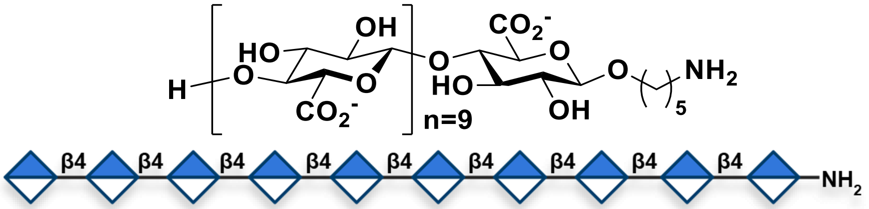

23

Figure S2. Oligosaccharides synthesized by AGA.

4.1 Synthesis and analytical data of 11

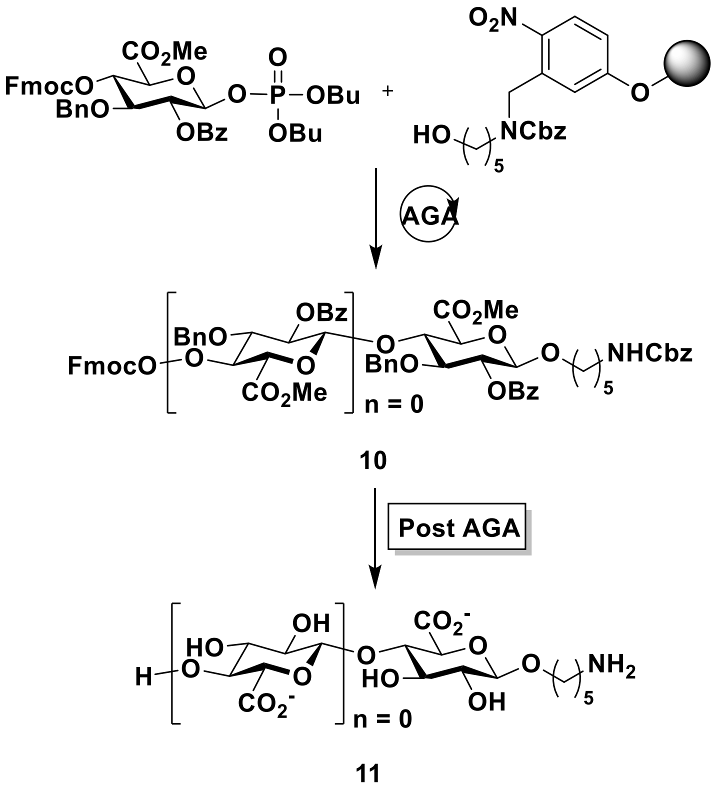

Steps		Modules	Notes
AGA		**A**	**L** swelling
	**BB-7**	**B, 2xD, E, G**	**D:** (-15 °C for 30 min, 0 °C for 30 min)
Post-AGA	Photocleavage	H	
	Purification	**K_{1P}**	
	Hydrolysis	**J**	Six hours (2 mL, 1:1 H_2O/THF)
	Hydrogenolysis	**K**	Five hours (2.5 mL, 4:1 H_2O/^{t}BuOH)
	Purification	**L_{1D}**	

Analytical data for 10

After AGA, photocleavage and NP-purification compound **10** was obtained as a colorless oil (5.0 mg, 42%). **^{1}H NMR** (700 MHz, $CDCl_3$): δ 7.98 (d, *J* = 7.5 Hz, 2H), 7.76 (t, *J* = 8.4 Hz, 2H), 7.66 – 7.54 (m, 3H), 7.41 (m, 4H), 7.37 – 7.28 (m, 7H), 7.15 – 7.03 (m, 5H), 5.32 (t, *J* = 8.1 Hz, 1H), 5.19 (t, *J* = 9.3 Hz, 1H), 5.07 (d, *J* = 5.6 Hz, 2H), 4.68 – 4.55 (m, 4H), 4.47 (dd, *J* = 10.7, 6.9 Hz, 1H), 4.37 (dd, *J* = 10.6, 7.4 Hz, 1H), 4.24 (t, *J* = 7.2 Hz, 1H), 4.12 (d, *J* = 9.7 Hz, 1H), 3.92 (m, 2H), 3.71 (s, 3H), 3.44 (d, *J* = 8.4 Hz, 1H), 2.94 (q, *J* = 6.8 Hz, 2H), 1.50 (m, 2H), 1.32 (m, 2H),

1.23 – 1.16 (m, 2H). **^{13}C NMR** (176 MHz, $CDCl_3$): δ 167.88, 164.99, 156.43, 154.18, 143.44, 143.15, 141.43, 137.31, 136.83, 133.51, 129.92, 129.67, 128.65, 128.61, 128.37, 128.25, 128.20, 128.09, 127.88, 127.37, 125.27, 125.20, 120.25, 101.26, 78.66, 75.25, 74.14, 73.07, 72.70, 70.52, 70.09, 66.64, 53.02, 46.77, 40.94, 29.85, 29.49, 28.92, 23.18. **HRMS QTOF-MS**: calcd. $C_{49}H_{49}NNaO_{12}$ for $[M+Na]^+$, 866.3152, found 866.3185.

^{1}H NMR of 10 (400 MHz, $CDCl_3$)

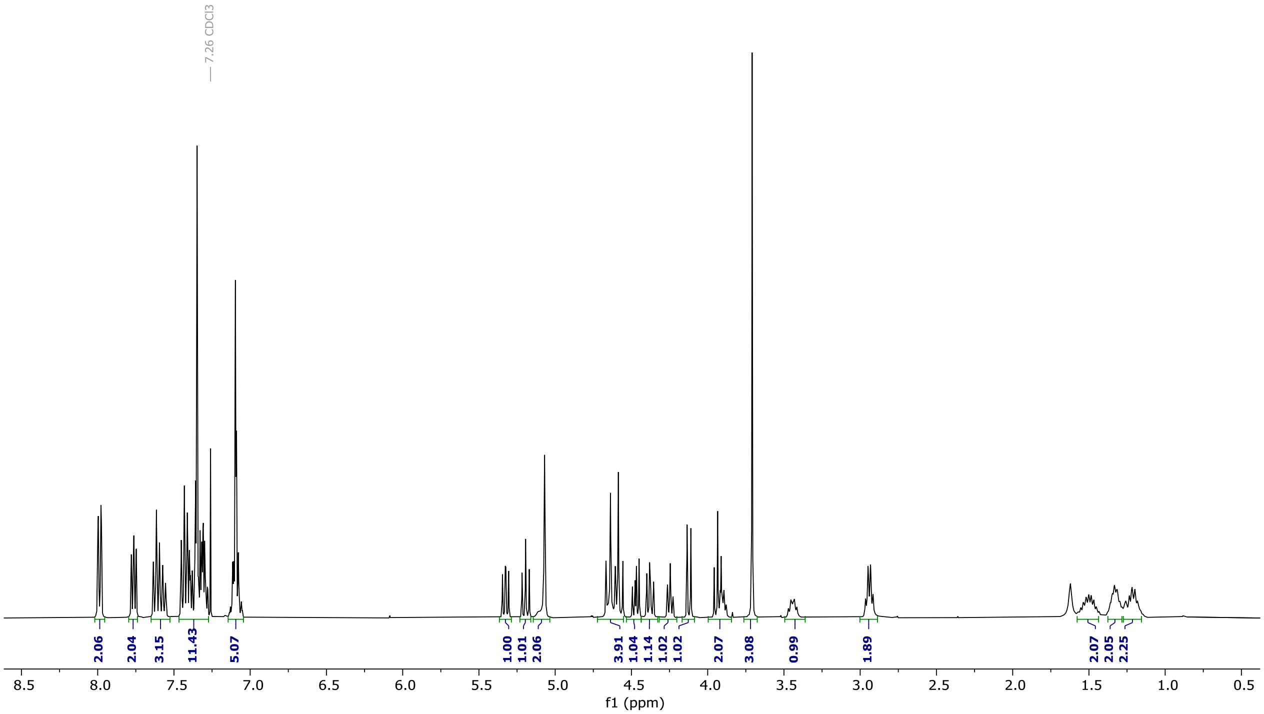

^{13}C NMR of 10 (176 MHz, $CDCl_3$)

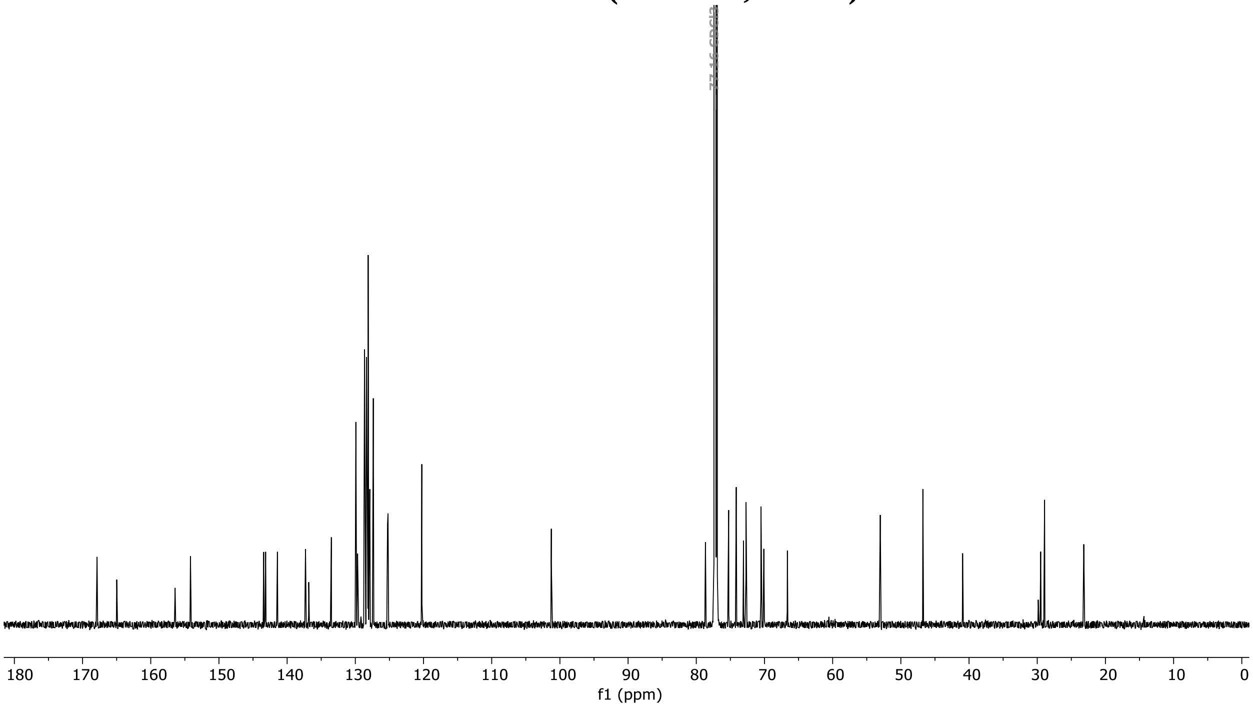

DEPT-135 of 10 (176 MHz, $CDCl_3$)

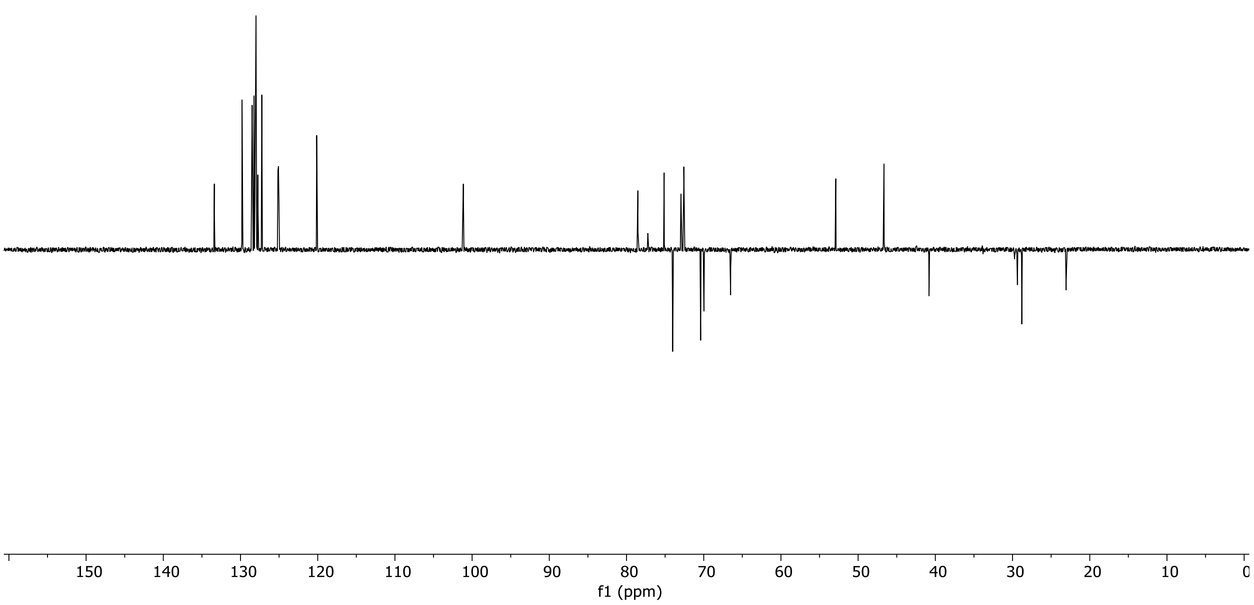

^{1}H-^{13}C HSQC of 10 (700 MHz, $CDCl_3$)

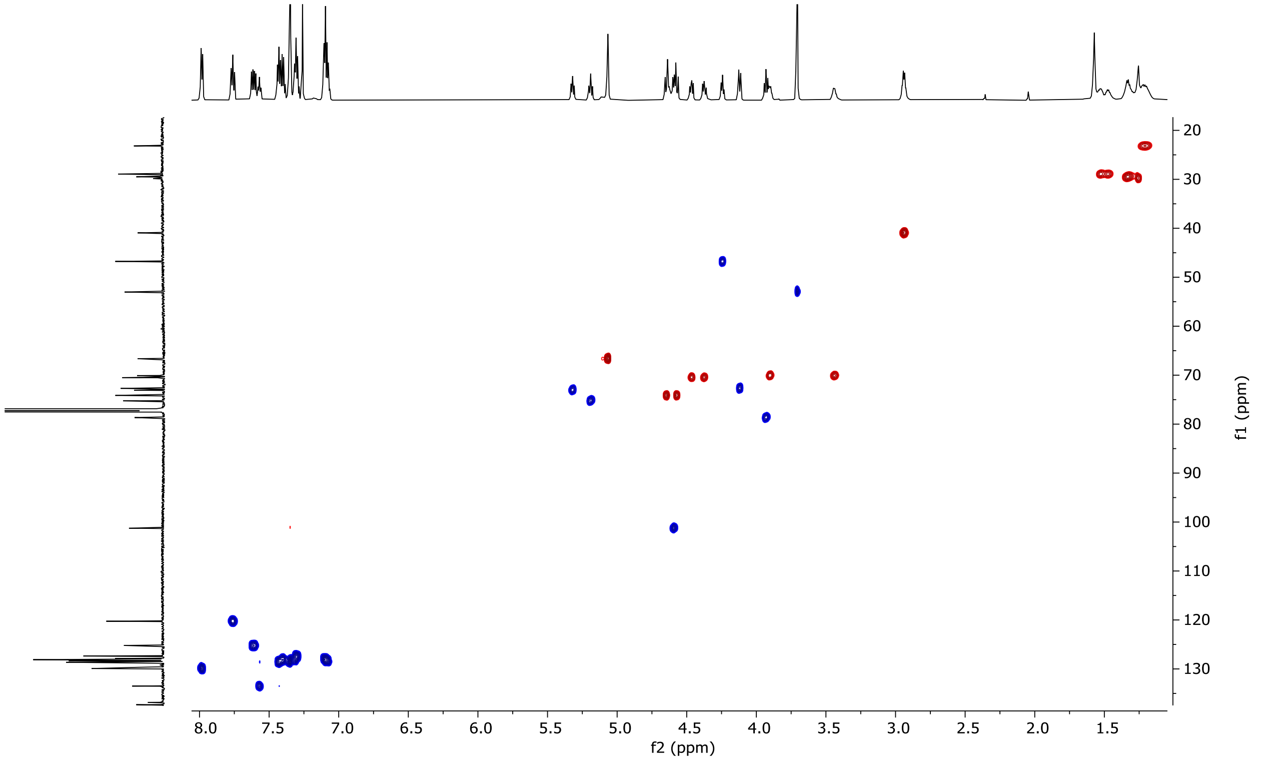

^{1}H-^{13}C coupled HSQC of 10 (700 MHz, $CDCl_3$)

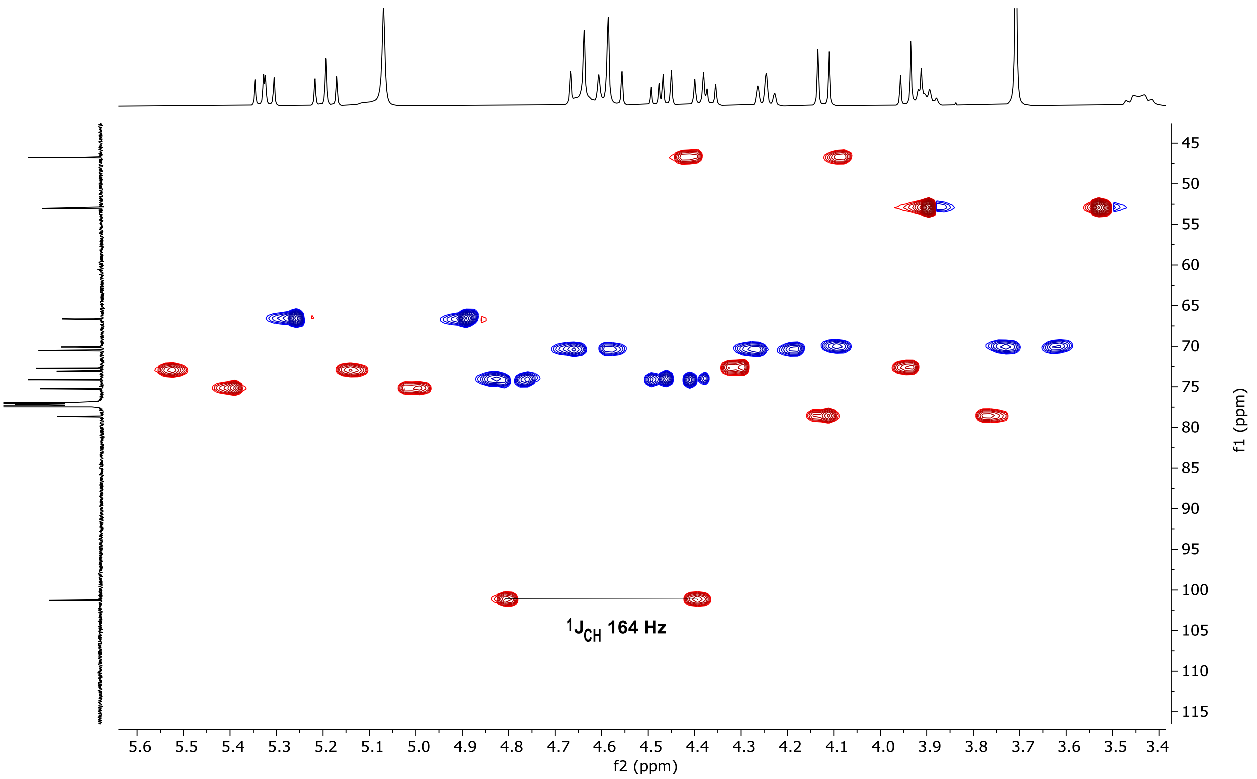

^{1}H-^{1}H COSY NMR of 10 (400 MHz, $CDCl_3$)

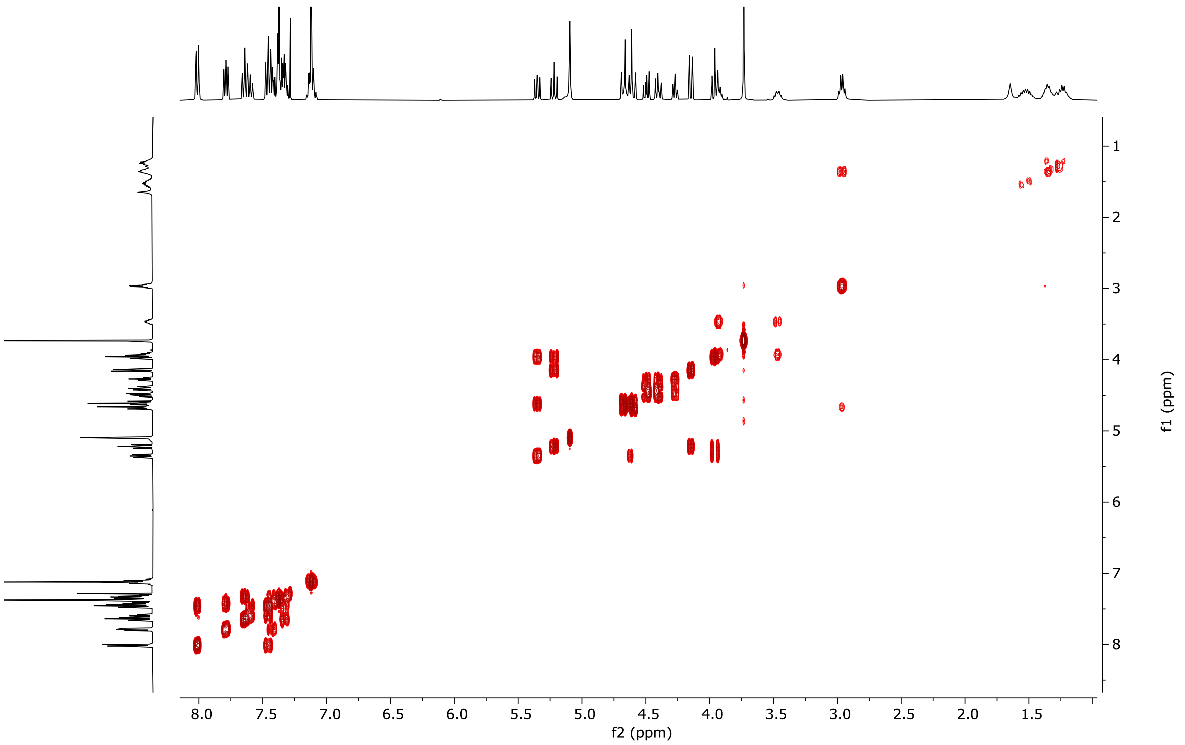

Analytical data for 11

After post-AGA and purification compound **11** was obtained as a white solid (0.84 mg, 57%). **^{1}H NMR** (700 MHz, D_2O): δ 4.35 (d, *J* = 8.0 Hz, 1H), 3.82 (dt, *J* = 10.1, 6.5 Hz, 1H), 3.59 (td, *J* = 9.7, 4.9 Hz, 2H), 3.42 – 3.36 (m, 2H), 3.22 – 3.15 (m, 1H), 2.89 (t, *J* = 7.5 Hz, 2H), 1.63 – 1.51 (m, 4H), 1.35 (m, 2H). **^{13}C NMR** (176 MHz, D_2O): δ 175.68, 102.03, 76.09, 75.57, 72.90, 71.74, 69.97, 39.30, 28.05, 26.22, 21.92. **HRMS QTOF-MS**: calcd. $C_{11}H_{20}NO_7^-$ for $[M-H]^-$ 278.1245, found 278.1247.

^{1}H NMR of 11 (700 MHz, D_2O)

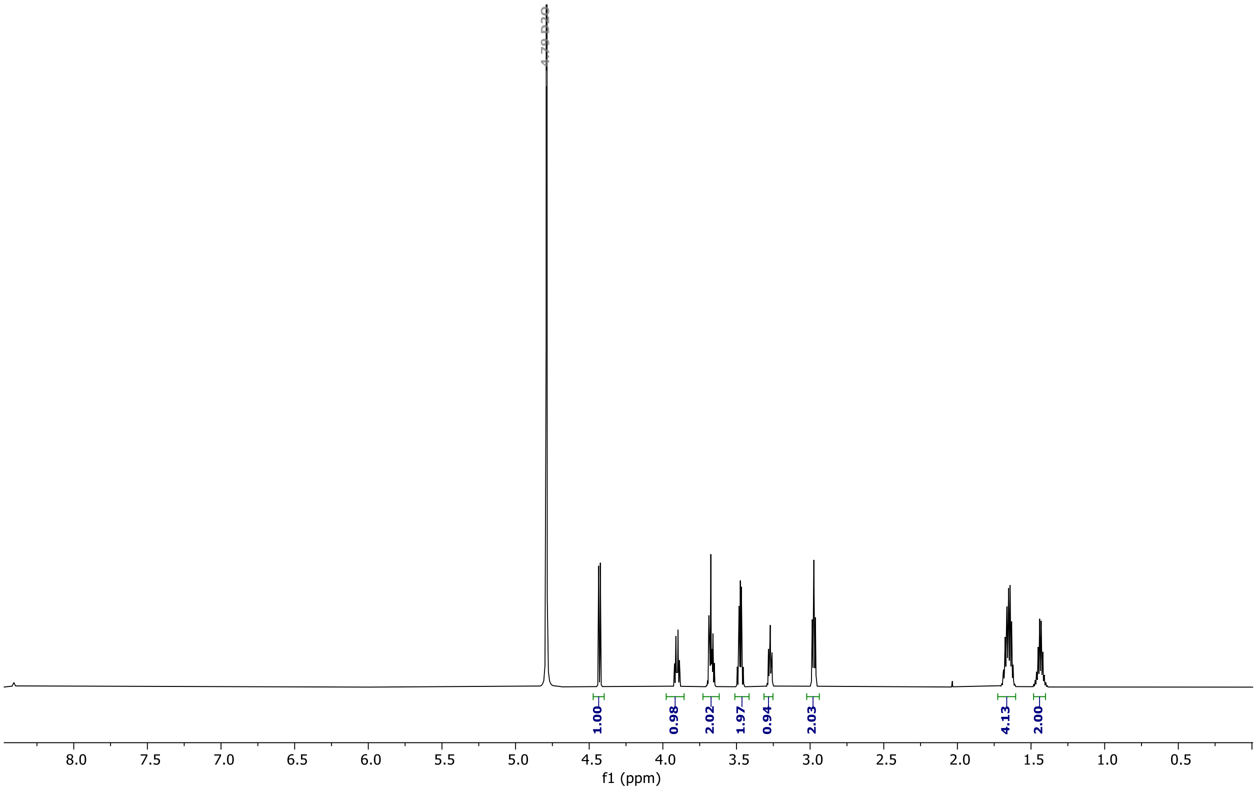

^{13}C NMR of 11 (176 MHz, D_2O)

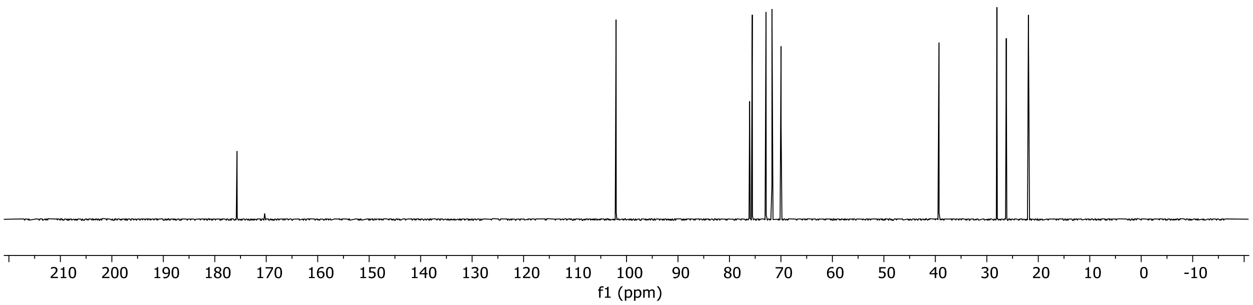

DEPT-135 of 11 (176 MHz, D_2O)

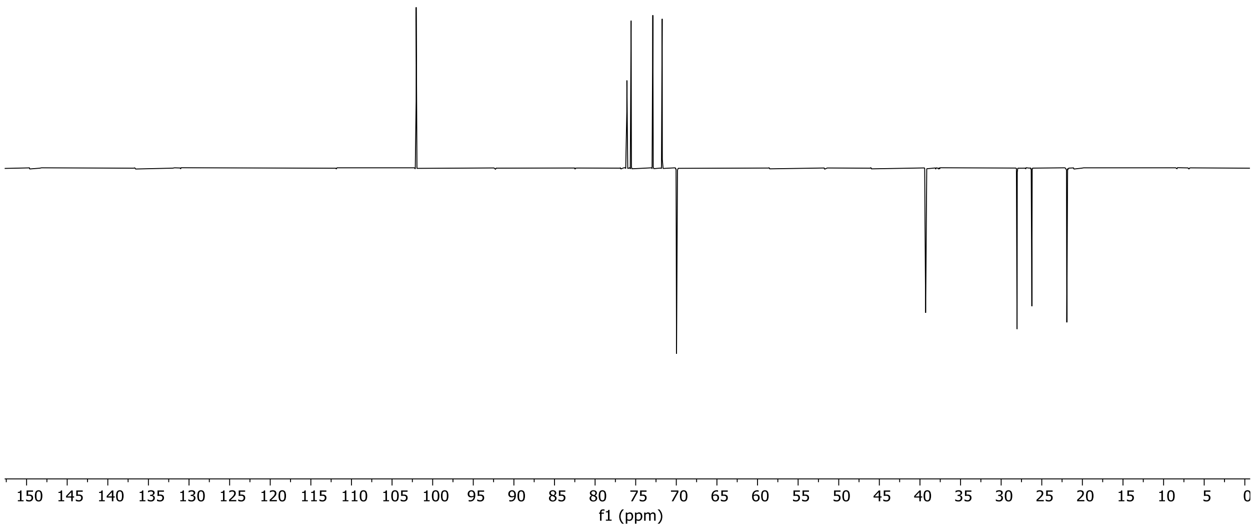

^{1}H-^{13}C HSQC of 11 (400 MHz, D_2O)

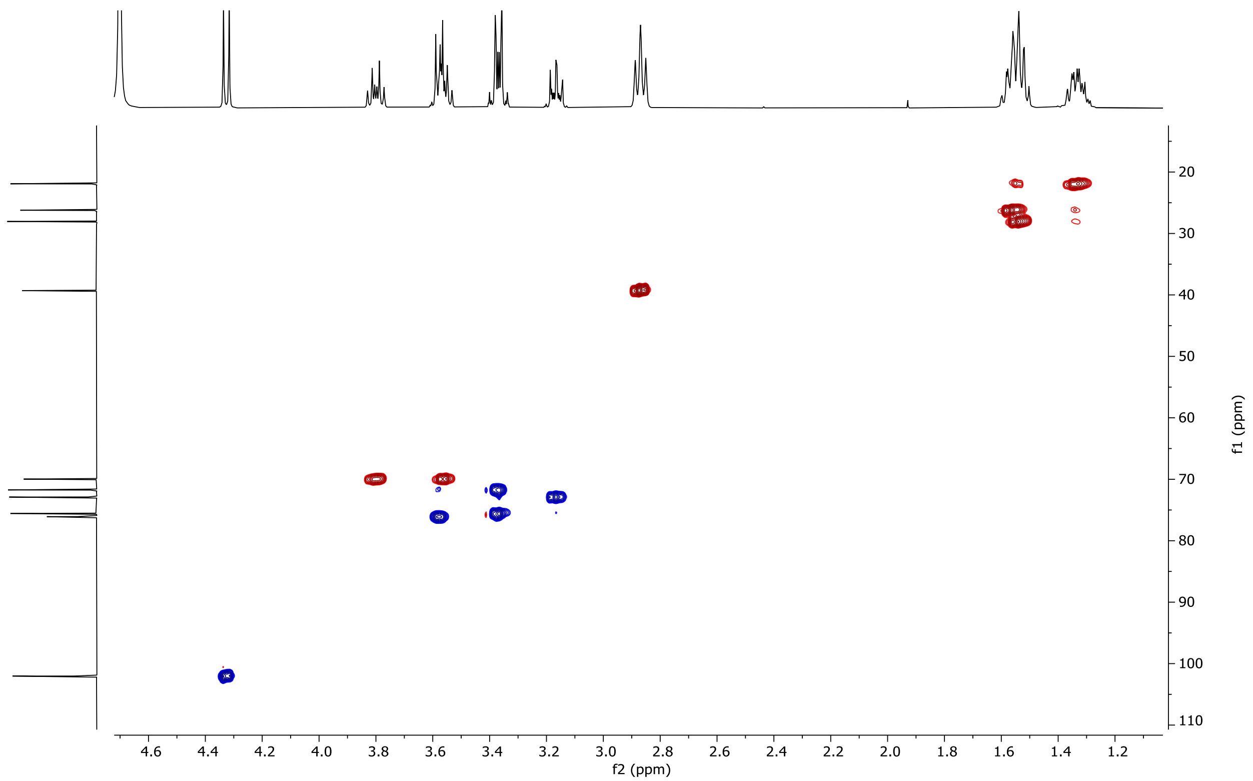

^{1}H-^{13}C coupled HSQC of 11 (700 MHz, D_2O)

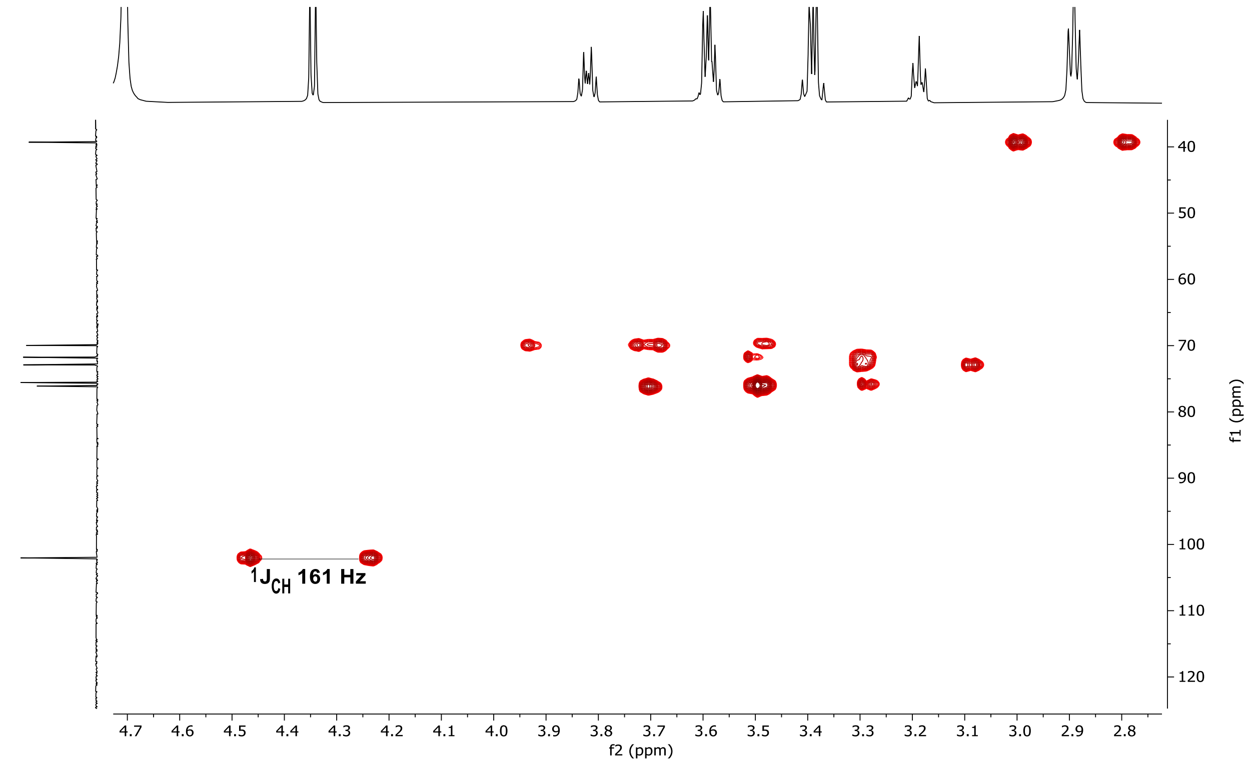

^{1}H-^{1}H COSY NMR of 11 (400 MHz, D_2O)

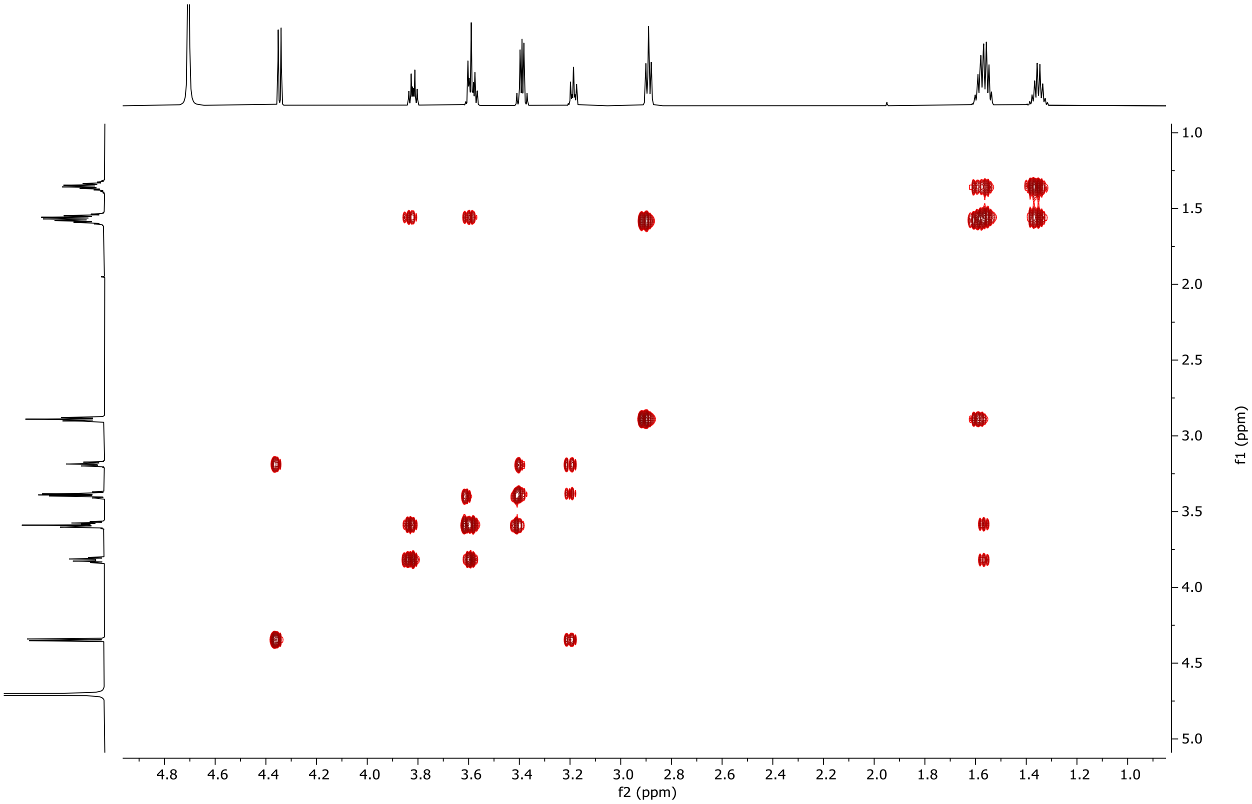

4.2 Synthesis and analytical data of 13

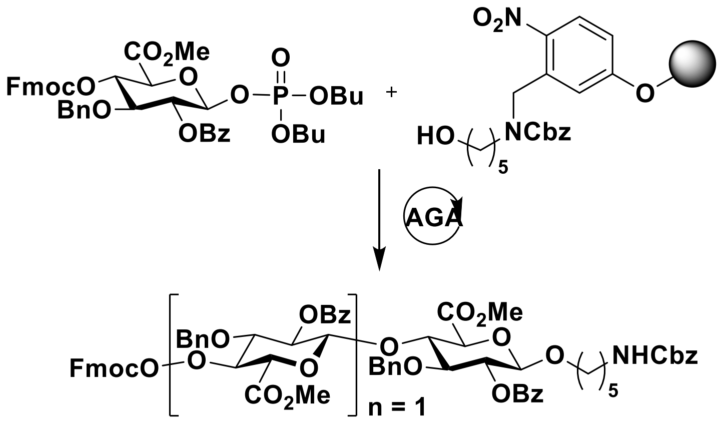

12

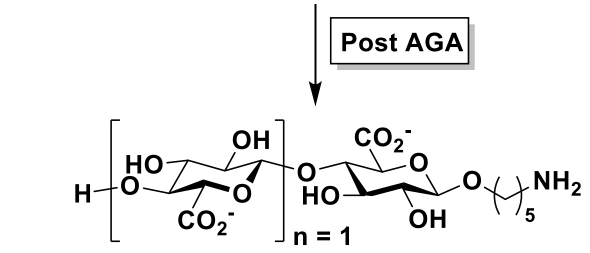

13

Steps		Modules	Notes
AGA		**A**	
	**BB-7**	**B, 2x D, E, G**	**D:** (-15 °C for 30 min, 0 °C for 30 min)
	**BB-7**	**B, 2xD,**	**D:** (-15 °C for 30 min, 0 °C for 30 min)
Post-AGA	Photocleavage	H	
	Purification	$\mathbf{L_{1P}}$	
	Hydrolysis	**J**	Six hours (2 mL, 1:1 H_2O/THF)
	Hydrogenolysis	**K**	Six hours (2.5 mL, 4:1 H_2O/^{t}BuOH)
	Purification	$\mathbf{L_{1D}}$	

Analytical data for 12

After AGA, photocleavage and NP-purification compound **12** was obtained as a colorless oil (7.4 mg, 40%). ^{1}**H NMR** (700 MHz, $CDCl_3$): δ 7.99 (d, *J* = 7.6 Hz, 2H), 7.88 (d, *J* = 7.6 Hz, 2H), 7.75

(t, J = 8.3 Hz, 2H), 7.59 (dd, J = 16.9, 7.2 Hz, 3H), 7.52 (t, J = 7.3 Hz, 1H), 7.46 (t, J = 7.5 Hz, 2H), 7.39 (p, J = 7.5 Hz, 4H), 7.35 (d, J = 5.7 Hz, 4H), 7.32 – 7.28 (m, 3H), 7.20 – 7.16 (m, 2H), 7.11 – 7.09 (m, 1H), 7.07 (t, J = 6.3 Hz, 6H), 5.32 (s, 1H), 5.16 (s, 1H), 5.13 – 5.06 (m, 2H), 5.01 – 4.92 (m, 2H), 4.70 – 4.53 (m, 4H), 4.50 – 4.41 (m, 2H), 4.36 – 4.27 (m, 2H), 4.23 (d, J = 7.2 Hz, 1H), 4.04 (d, J = 9.7 Hz, 1H), 3.92 (s, 1H), 3.88 – 3.82 (m, 2H), 3.74 – 3.69 (m, 1H), 3.50 (d, J = 5.4 Hz, 3H), 3.43 (d, J = 6.0 Hz, 3H), 3.34 – 3.25 (m, 1H), 2.90 (q, J = 6.9 Hz, 2H), 1.47 – 1.30 (m, 4H), 1.14 (m, J = 16.6, 11.2, 5.2 Hz, 2H). **^{13}C NMR** (176 MHz, $CDCl_3$): δ 168.73, 167.32, 165.17, 165.02, 156.40, 154.13, 143.43, 143.12, 141.44, 141.40, 138.46, 137.26, 136.83, 133.48, 133.29, 130.01, 129.86, 129.78, 129.72, 128.64, 128.56, 128.52, 128.47, 128.36, 128.24, 128.20, 128.11, 128.08, 128.00, 127.87, 127.77, 127.68, 127.36, 127.33, 125.30, 125.21, 120.23, 101.41, 100.95, 79.88, 79.07, 78.79, 75.44, 74.58, 74.53, 74.23, 73.32, 73.13, 72.60, 70.54, 69.88, 66.62, 52.81, 52.55, 46.72, 40.91, 29.85, 29.48, 28.87, 23.13. **HRMS QTOF-MS**: calcd. $C_{70}H_{69}NNaO_{19}$ for $[M+Na]^+$ 1250.4361, found 1250.4410.

^{1}H NMR of 12 (700 MHz, D_2O)

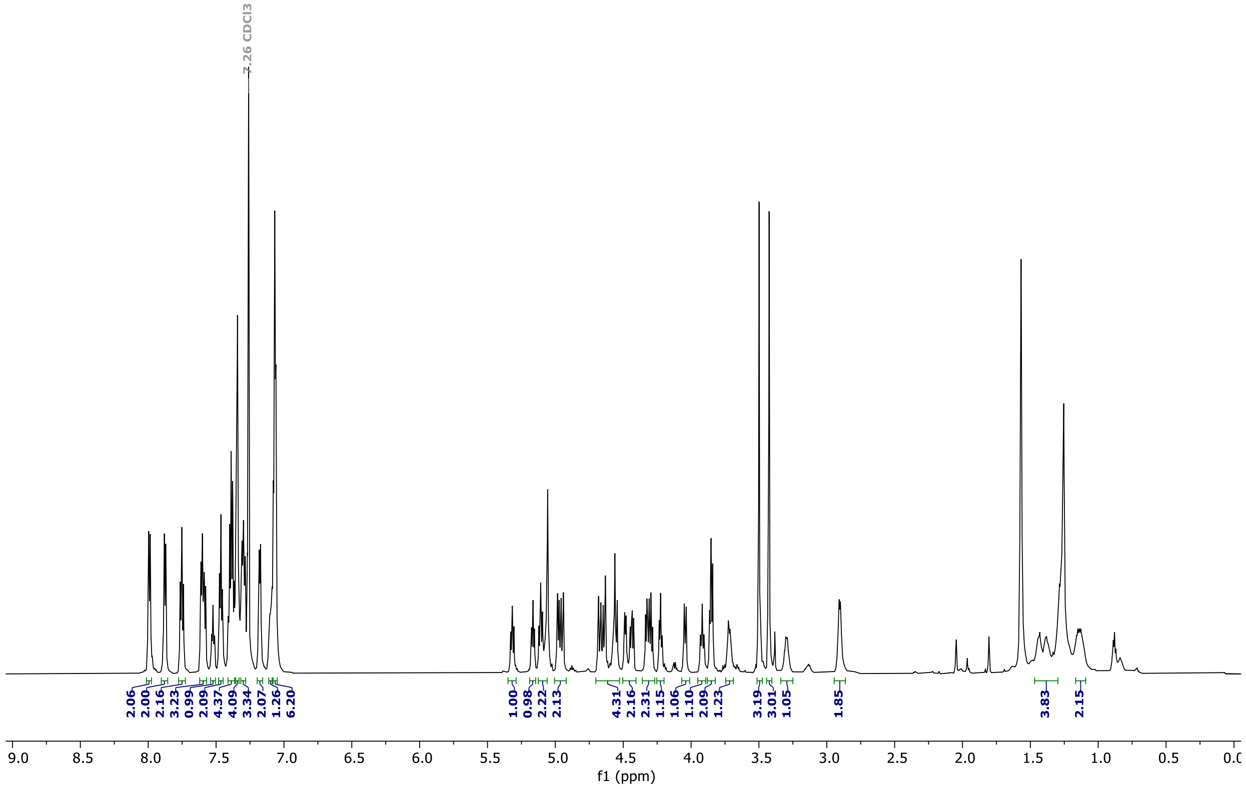

^{13}C NMR of 12 (176 MHz, D_2O)

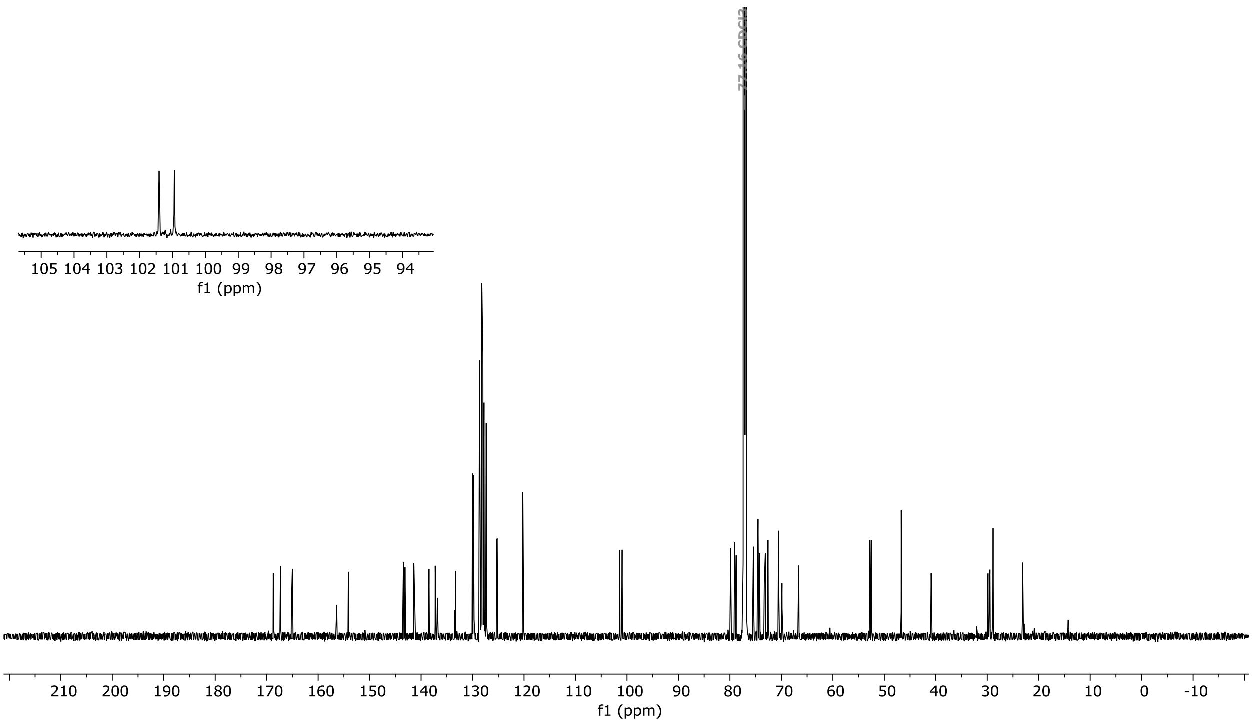

DEPT-135 of 12 (176 MHz, D_2O)

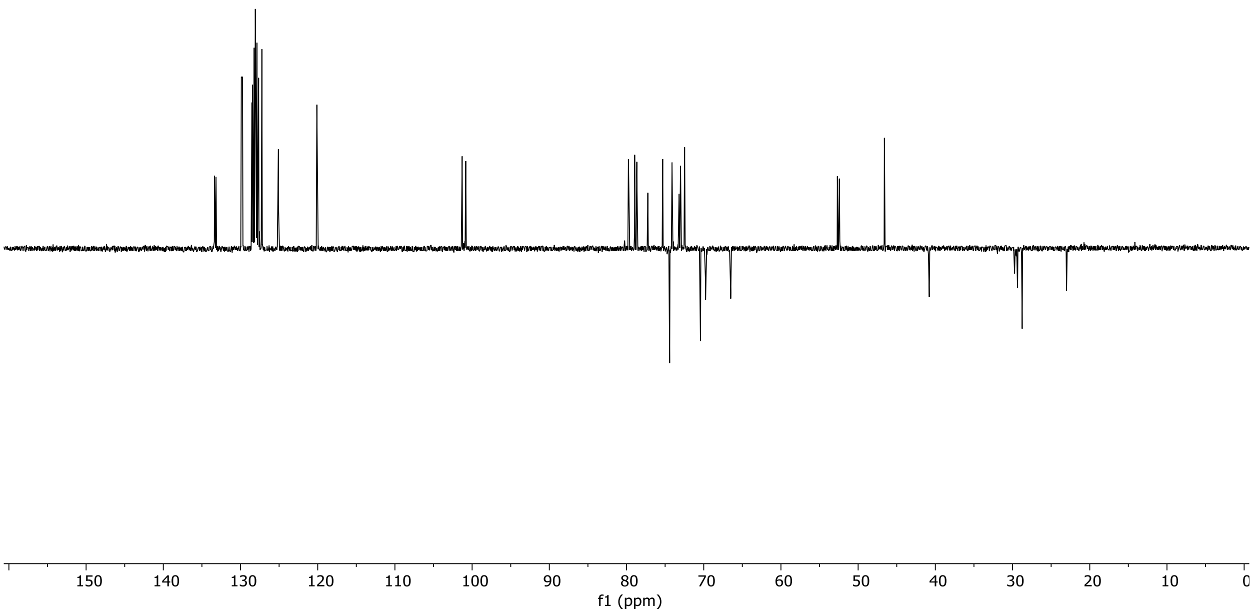

^{1}H-^{13}C HSQC of 12 (700 MHz, D_2O)

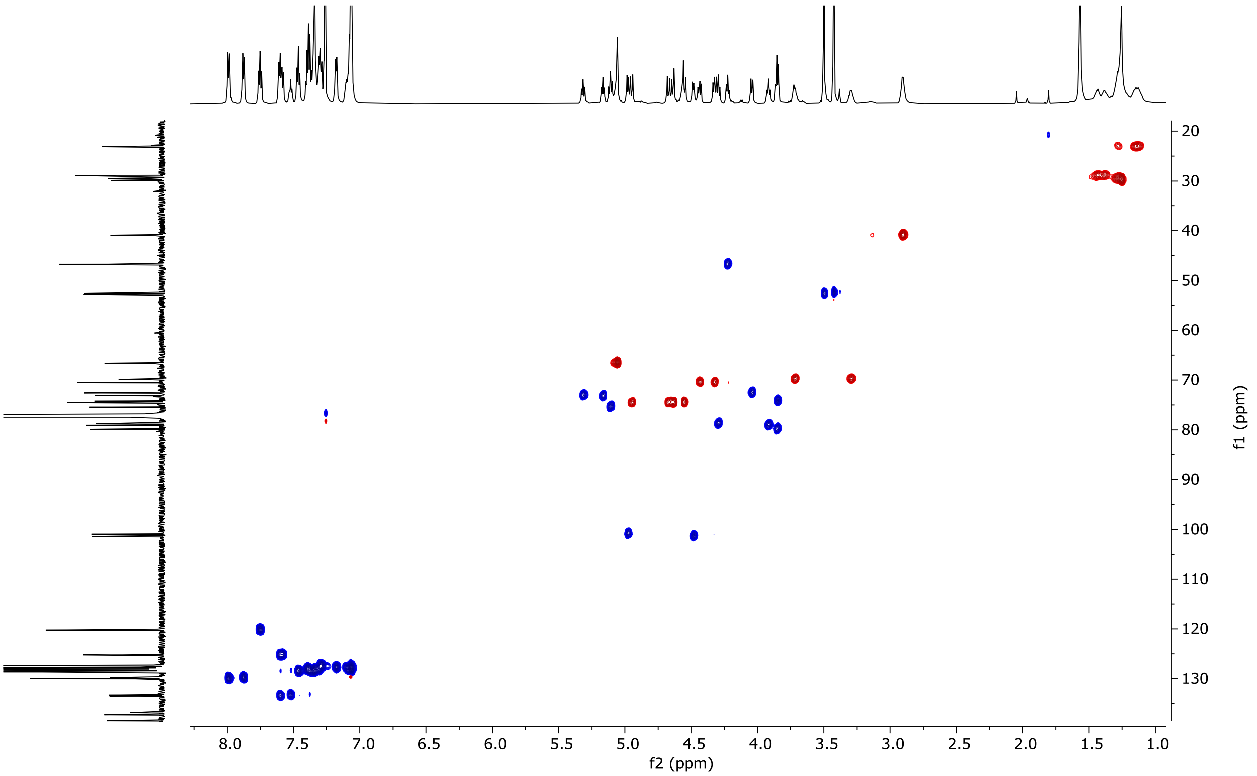

^{1}H-^{13}C coupled HSQC of 12 (400 MHz, D_2O)

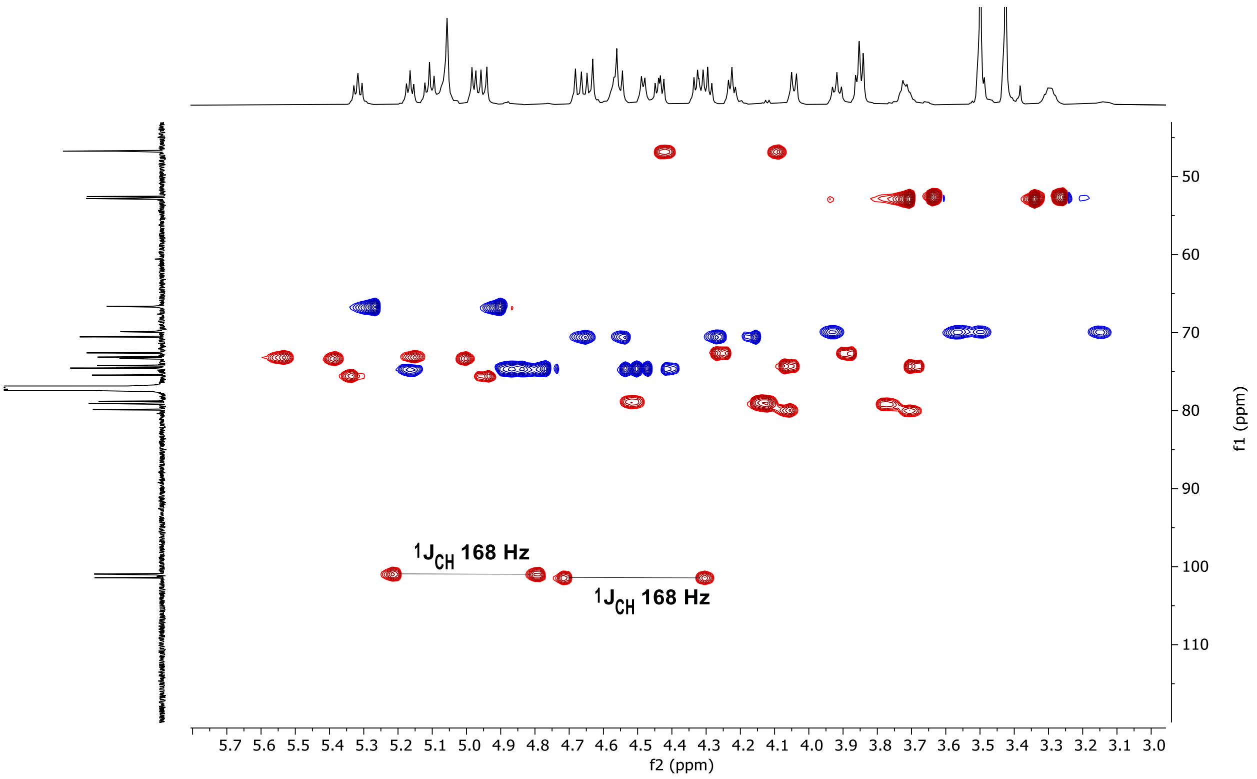

^{1}H-^{1}H COSY NMR of 12 (700 MHz, D_2O)

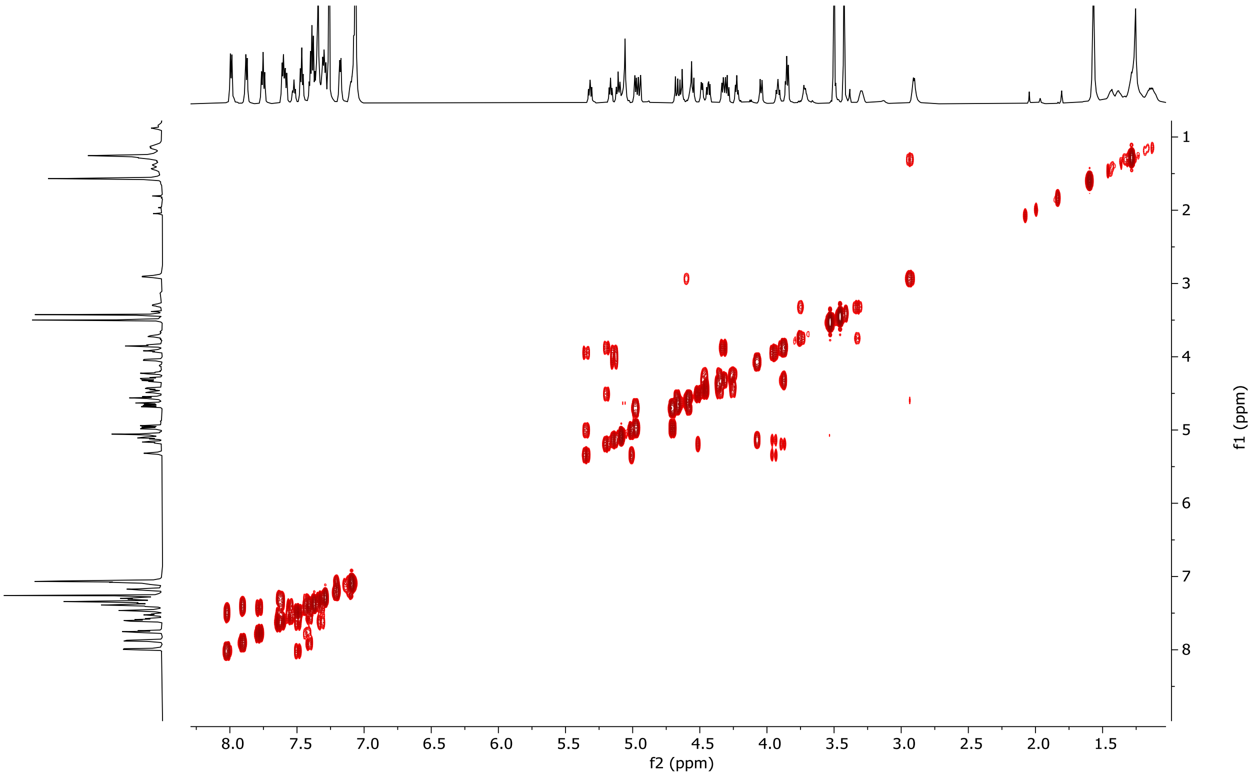

Analytical data for 13

After post-AGA and purification compound **13** was obtained as a white solid (1.45 mg, 58%). **^{1}H NMR** (700 MHz, D_2O): δ 4.48 (dd, *J* = 10.9, 7.9 Hz, 2H), 3.89 (dt, *J* = 10.2, 6.4 Hz, 1H), 3.82 (d, *J* = 9.8 Hz, 1H), 3.74 (d, *J* = 9.2 Hz, 1H), 3.68 (dt, *J* = 12.1, 7.7 Hz, 2H), 3.61 (t, *J* = 9.1 Hz, 1H), 3.49 (p, *J* = 8.8 Hz, 2H), 3.34 – 3.27 (m, 2H), 2.97 (t, *J* = 7.5 Hz, 2H), 1.65 (m, 4H), 1.44 (m, 2H). **^{13}C NMR** (176 MHz, D_2O): δ 175.38, 174.98, 102.30, 101.92, 80.88, 75.77, 75.59, 75.18, 74.44, 73.01, 72.57, 71.57, 70.01, 39.30, 28.03, 26.18, 21.89. **HRMS QTOF-MS**: calcd. $C_{17}H_{28}NO_{13}^-$ for $[M-H]^-$ 454.1566, found 454.1553.

^{1}H NMR of 13 (700 MHz, D_2O)

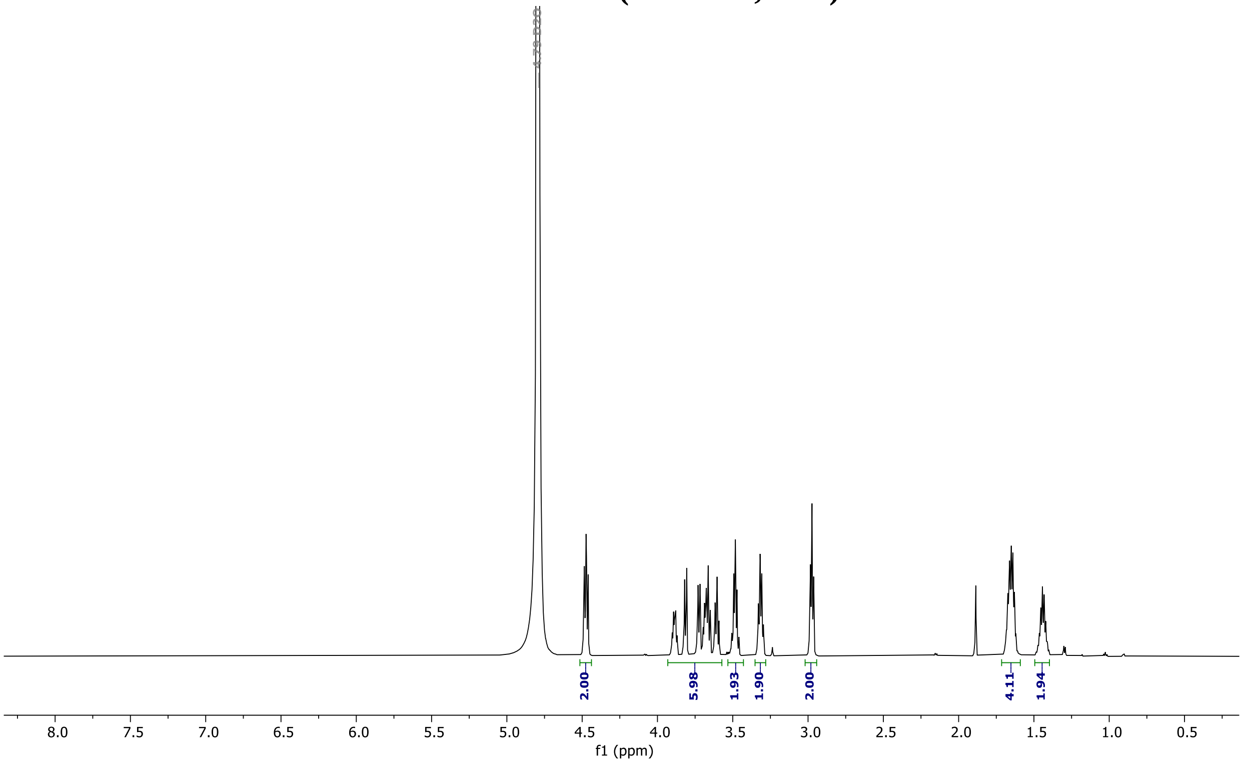

^{13}C NMR of 13 (176 MHz, D_2O)

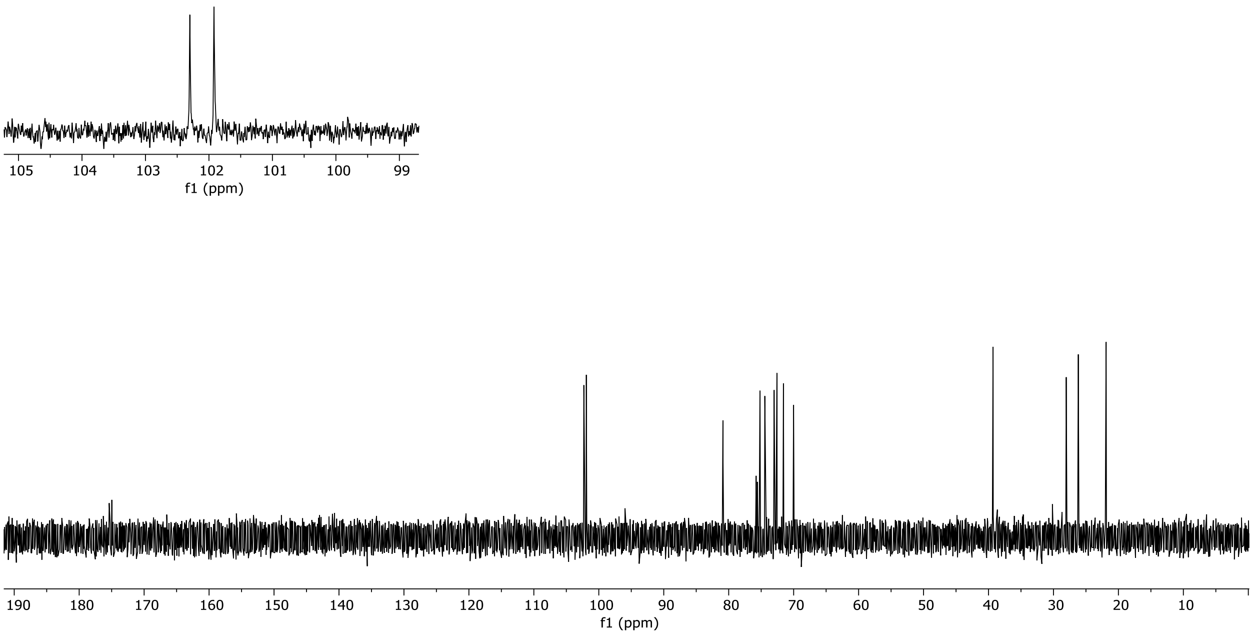

DEPT-135 NMR of 13 (176 MHz, D_2O)

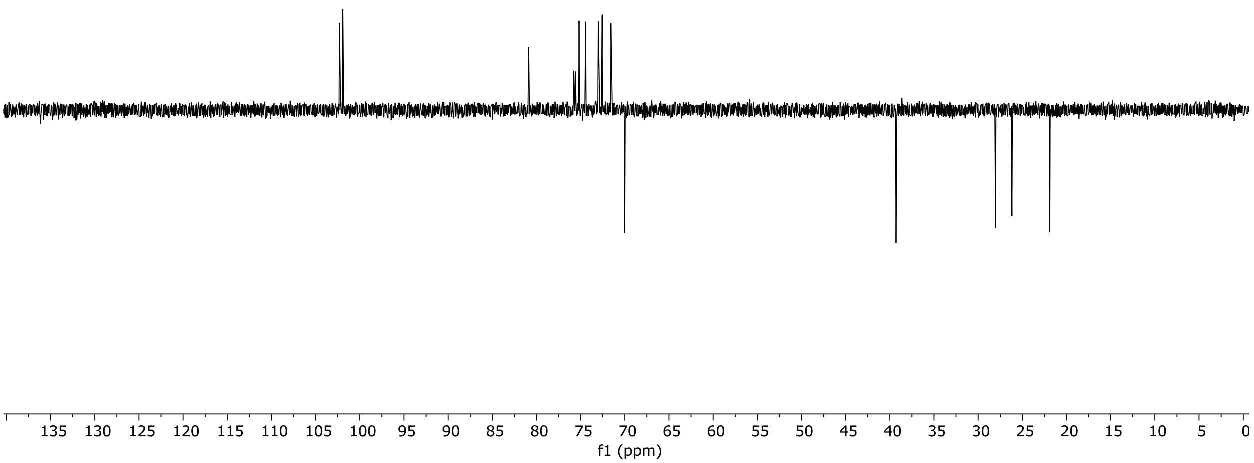

^{1}H-^{13}C HSQC NMR of 13 (700 MHz, D_2O)

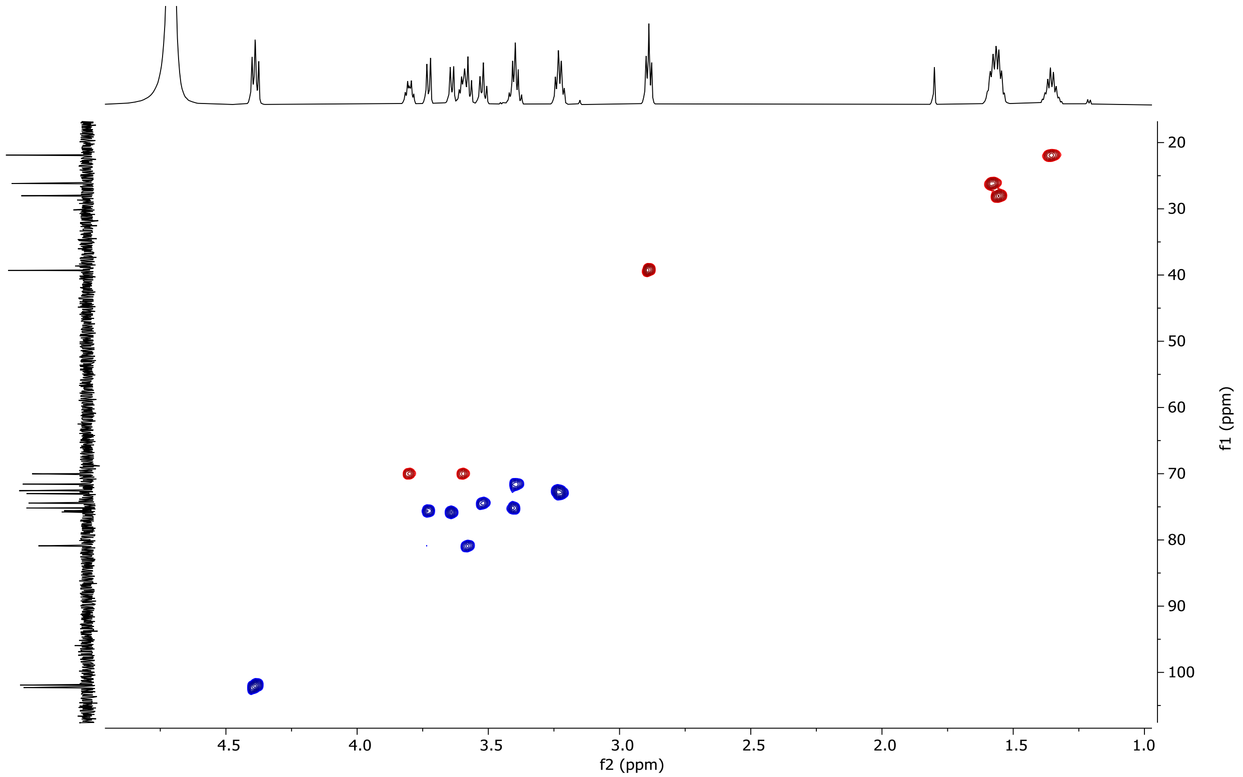

^{1}H-^{13}C coupled HSQC NMR of 13 (400 MHz, D_2O)

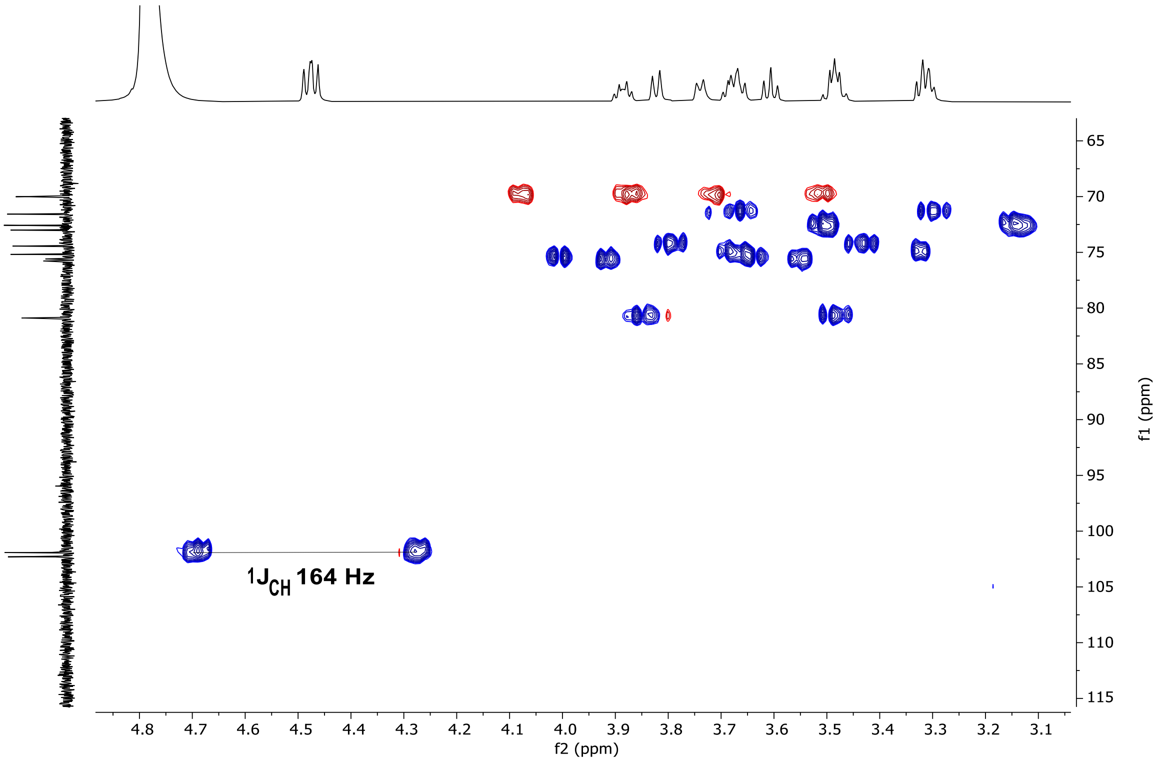

^{1}H-^{1}H COSY NMR of 13 (700 MHz, D_2O)

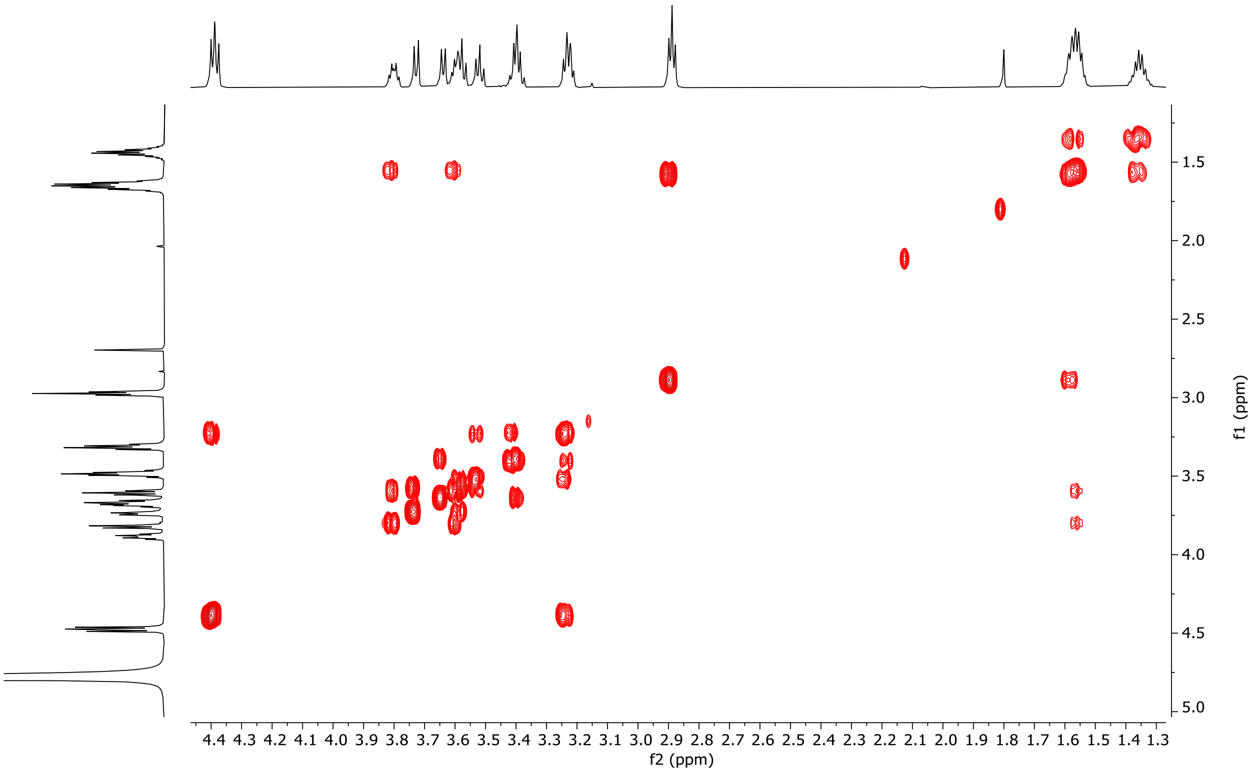

4.3 Synthesis and analytical data of 15

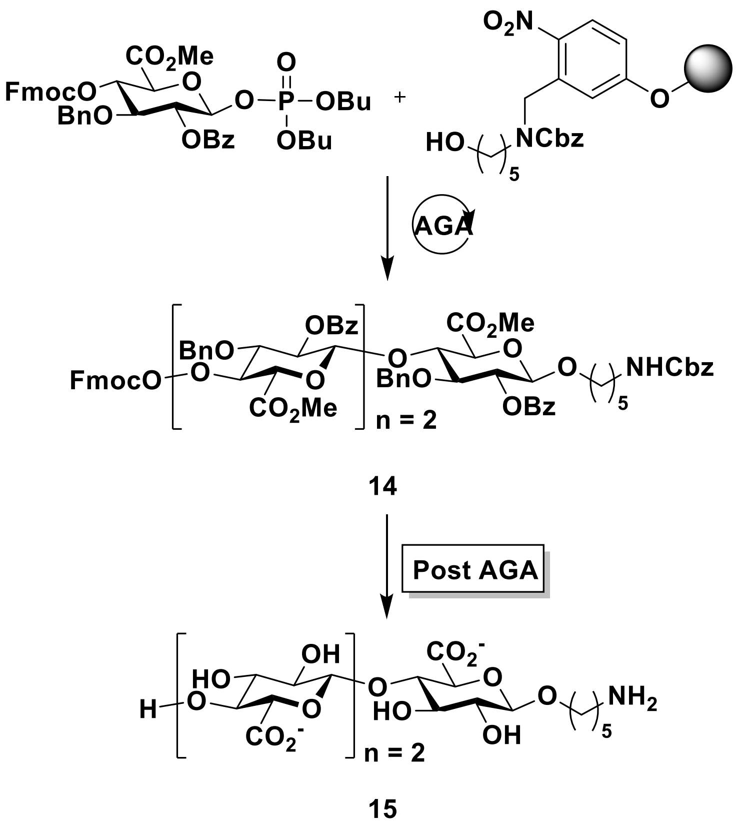

Step		Modules	Notes
AGA		**A**	**L** swelling
	**BB-7**	**2x (B, 2x D, E, G)**	**D:** (-15 °C for 30 min, 0 °C for 30 min)
	**BB-7**	**B, 2xC**	**D:** (-15 °C for 30 min, 0 °C for 30 min)
Post-AGA	Photocleavage	**H**	
	Purification	**L_{1P}**	
	Hydrolysis	**J**	Twelve hours (2mL, 1:1 H_2O/THF)
	Hydrogenolysis	**K**	Twelve hours (2.5 mL, 4:1 H_2O/^{t}BuOH)
	Purification	**L_{1D}**	

Analytical data for 14

After AGA, photocleavage and NP-purification compound **14** was obtained as a colorless oil (8.7 mg, 36%). **^{1}H NMR** (600 MHz, $CDCl_3$): δ 7.98 (dd, *J* = 8.1, 1.4 Hz, 2H), 7.88 (m, 4H), 7.75 (t, *J* = 7.6 Hz, 2H), 7.63 – 7.50 (m, 5H), 7.47 (t, *J* = 7.7 Hz, 2H), 7.44 – 7.32 (m, 11H), 7.29 (qd, *J* = 7.5, 2.3 Hz, 3H), 7.16 (tt, *J* = 6.8, 3.5 Hz, 3H), 7.13 – 7.02 (m, 13H), 5.31 (dd, *J* = 9.3, 7.8 Hz, 1H), 5.21 (t, *J* = 8.3 Hz, 1H), 5.14 – 5.06 (m, 4H), 5.02 (d, *J* = 11.9 Hz, 1H), 4.92 (d, *J* = 7.8 Hz, 1H), 4.80 – 4.74 (m, 2H), 4.64 (d, *J* = 11.8 Hz, 2H), 4.54 (dd, *J* = 21.8, 11.6 Hz, 3H), 4.43 (d, *J* = 7.2 Hz, 1H), 4.34 – 4.21 (m, 3H), 4.16 (t, *J* = 8.6 Hz, 1H), 4.05 (d, *J* = 9.9 Hz, 1H), 3.91 (t, *J* = 9.2 Hz, 1H), 3.84 (t, *J* = 8.6 Hz, 1H), 3.79 – 3.68 (m, 4H), 3.46 (d, *J* = 11 Hz, 6H), 3.31 – 3.25 (m, 1H), 3.18 (s, 3H), 2.90 (q, *J* = 6.9 Hz, 2H), 1.28 (m, 4H), 1.18 – 1.09 (m, 2H). **^{13}C NMR** (151 MHz, $CDCl_3$): δ 168.63, 168.21, 167.26, 165.18, 165.03, 156.38, 154.11, 143.44, 143.13, 141.44, 141.40, 138.45, 138.32, 137.28, 136.84, 133.52, 133.26, 133.23, 129.97, 129.90, 129.85, 129.82, 129.66, 128.62, 128.58, 128.53, 128.48, 128.44, 128.39, 128.36, 128.21, 128.17, 128.12, 128.09, 128.07, 127.97, 127.94, 127.86, 127.77, 127.71, 127.34, 127.28, 125.30, 125.20, 120.21, 101.43, 100.94, 100.75, 80.29, 79.56, 79.06, 78.57, 78.12, 75.45, 75.04, 74.57, 74.51, 74.39, 73.98, 73.20, 73.12, 73.07, 72.67, 70.53, 69.87, 66.60, 52.76, 52.62, 52.45, 46.73, 40.89, 29.84, 29.45, 28.86, 23.10. **HRMS QTOF-MS**: calcd. $C_{91}H_{89}NNaO_{26}$ for $[M+Na]^+$ 1634.5571, found 1634.5591.

^{1}H NMR of 14 (600 MHz, $CDCl_3$)

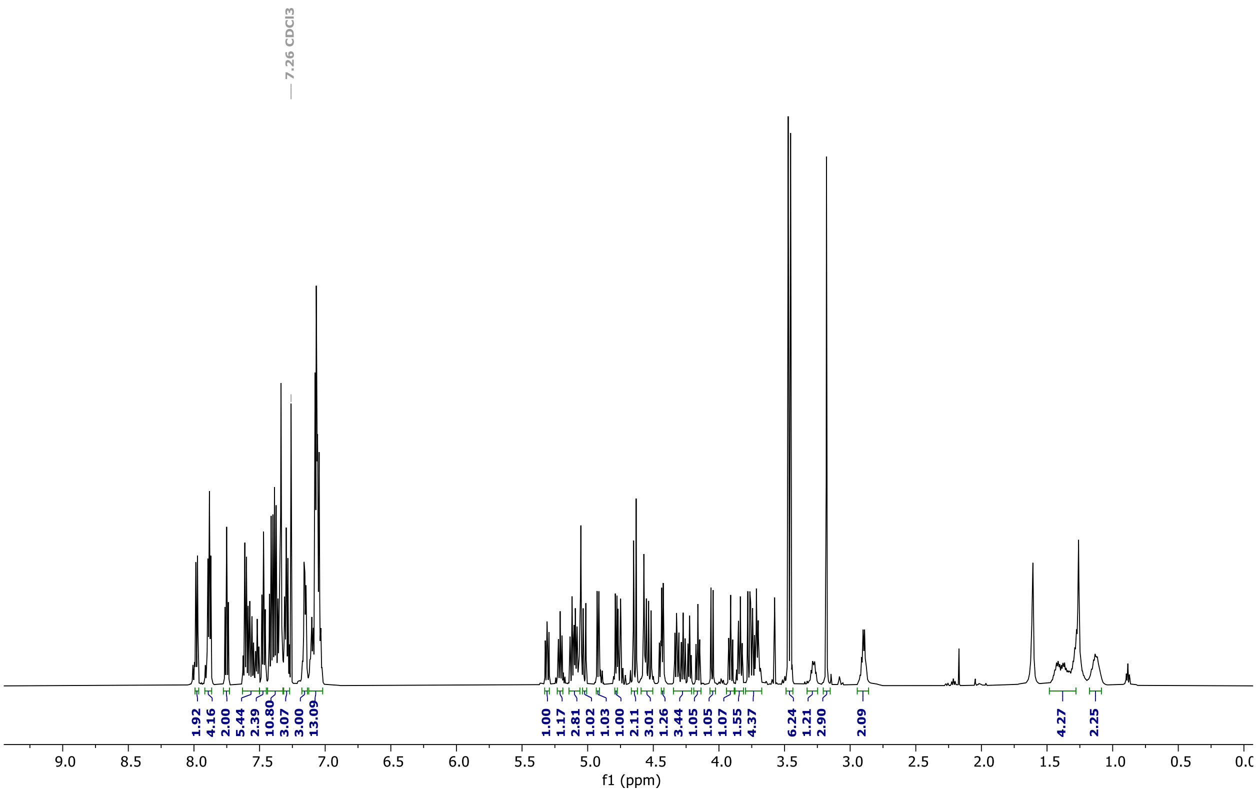

^{13}C NMR of 14 (152 MHz, $CDCl_3$)

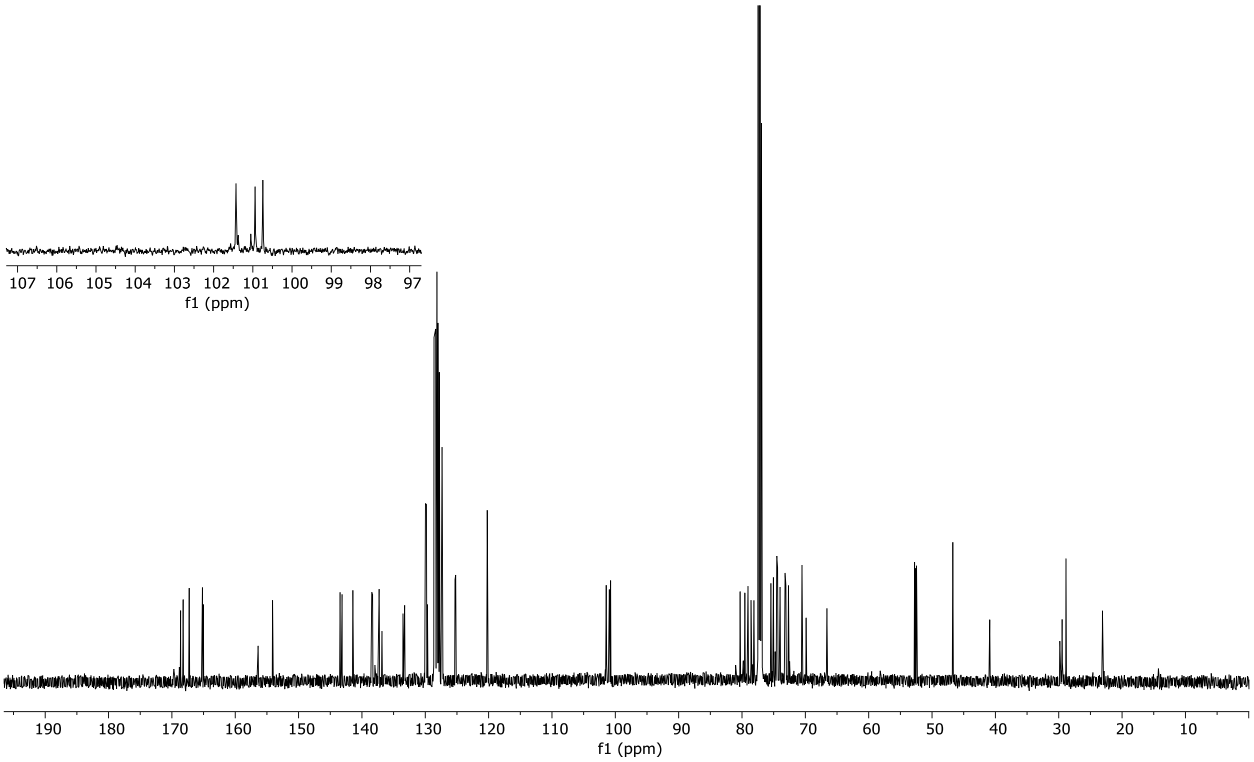

DEPT-135 of 14 (152 MHz, $CDCl_3$)

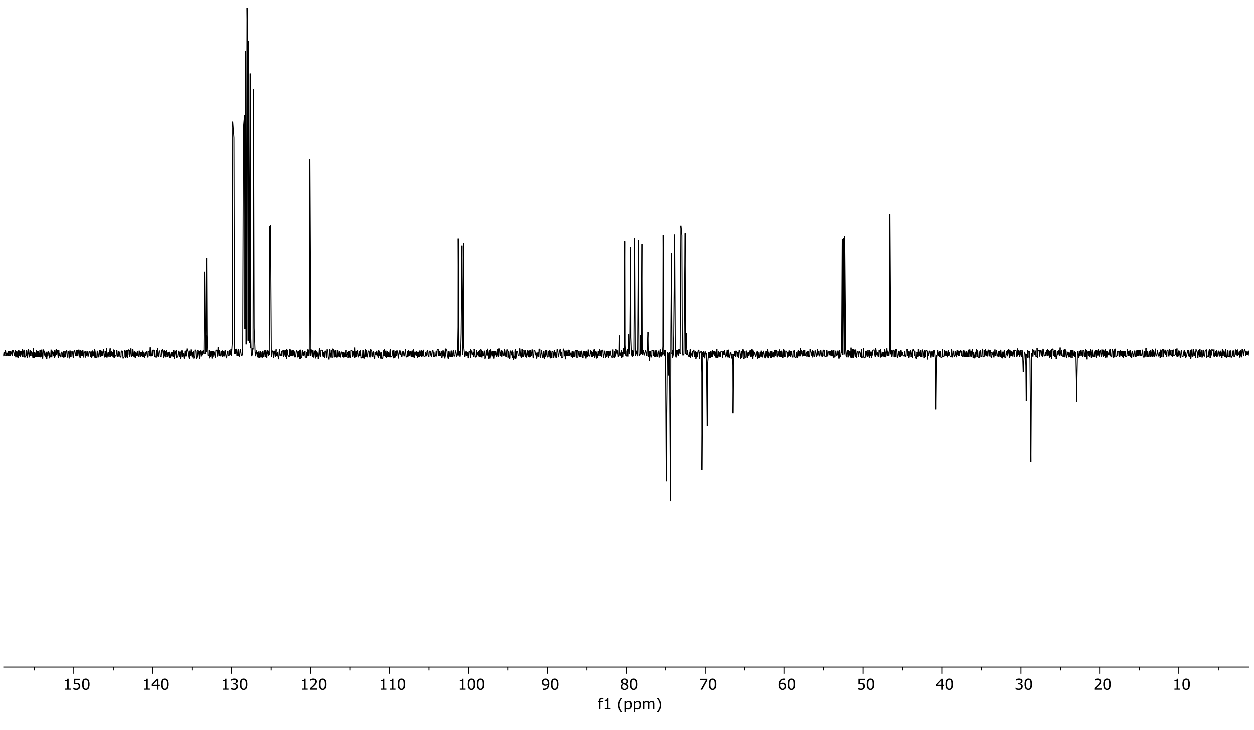

^{1}H-^{13}C HSQC of 14 (600 MHz, $CDCl_3$)

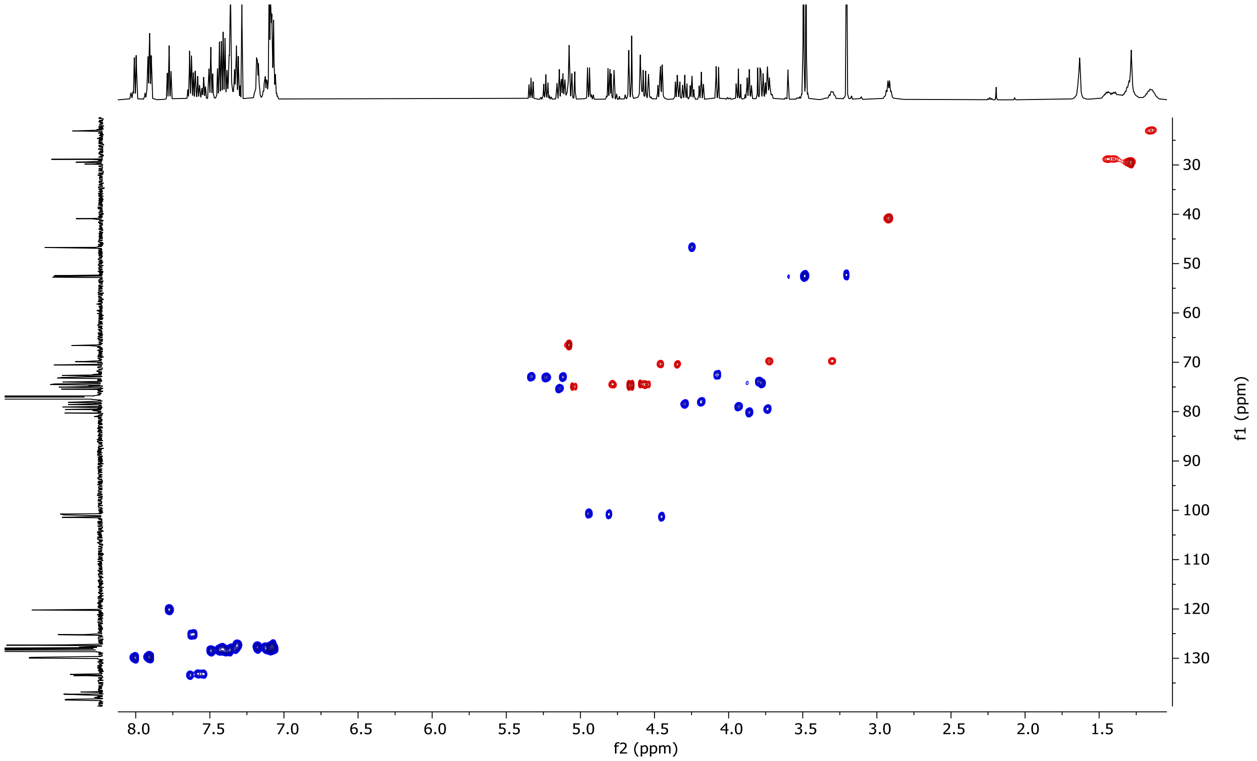

^{1}H-^{13}C coupled HSQC of 14 (400 MHz, $CDCl_3$)

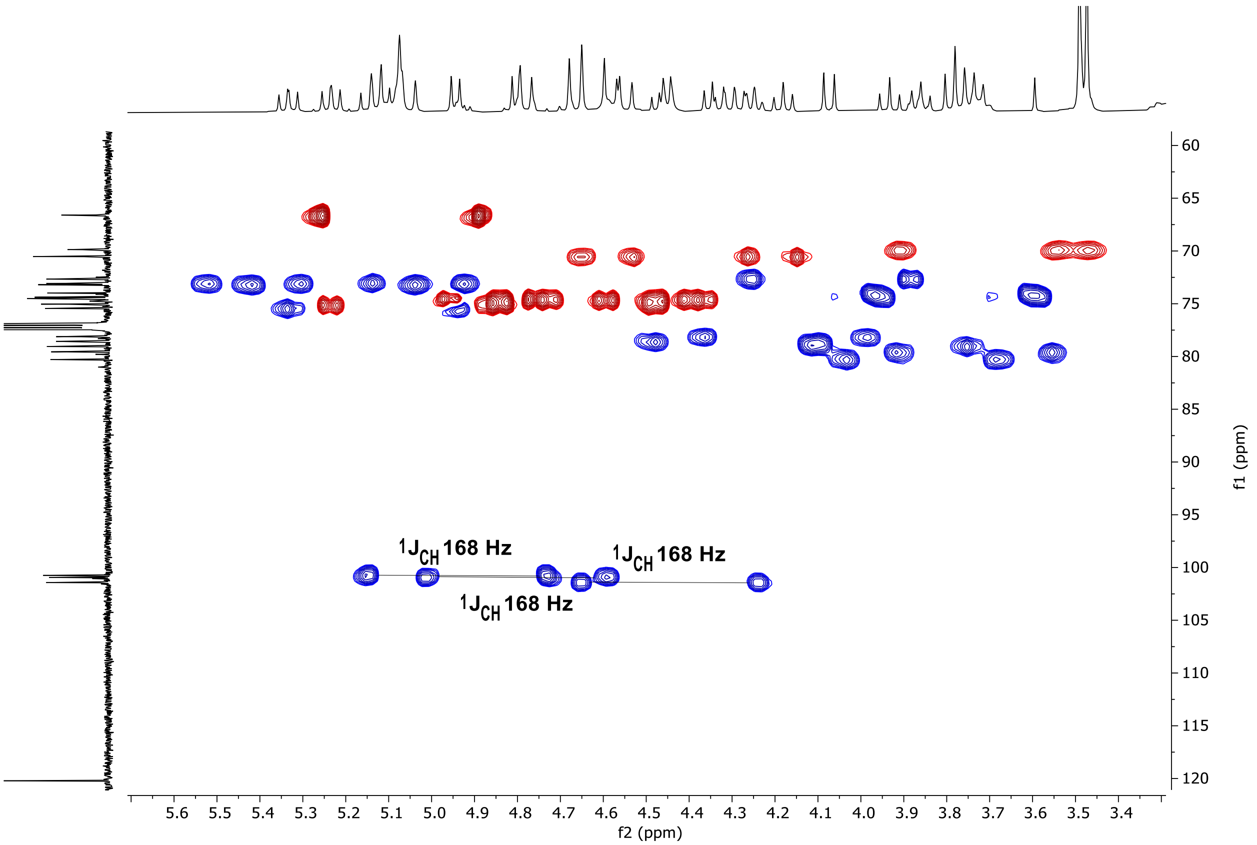

^{1}H-^{1}H COSY NMR of 14 (600 MHz, $CDCl_3$)

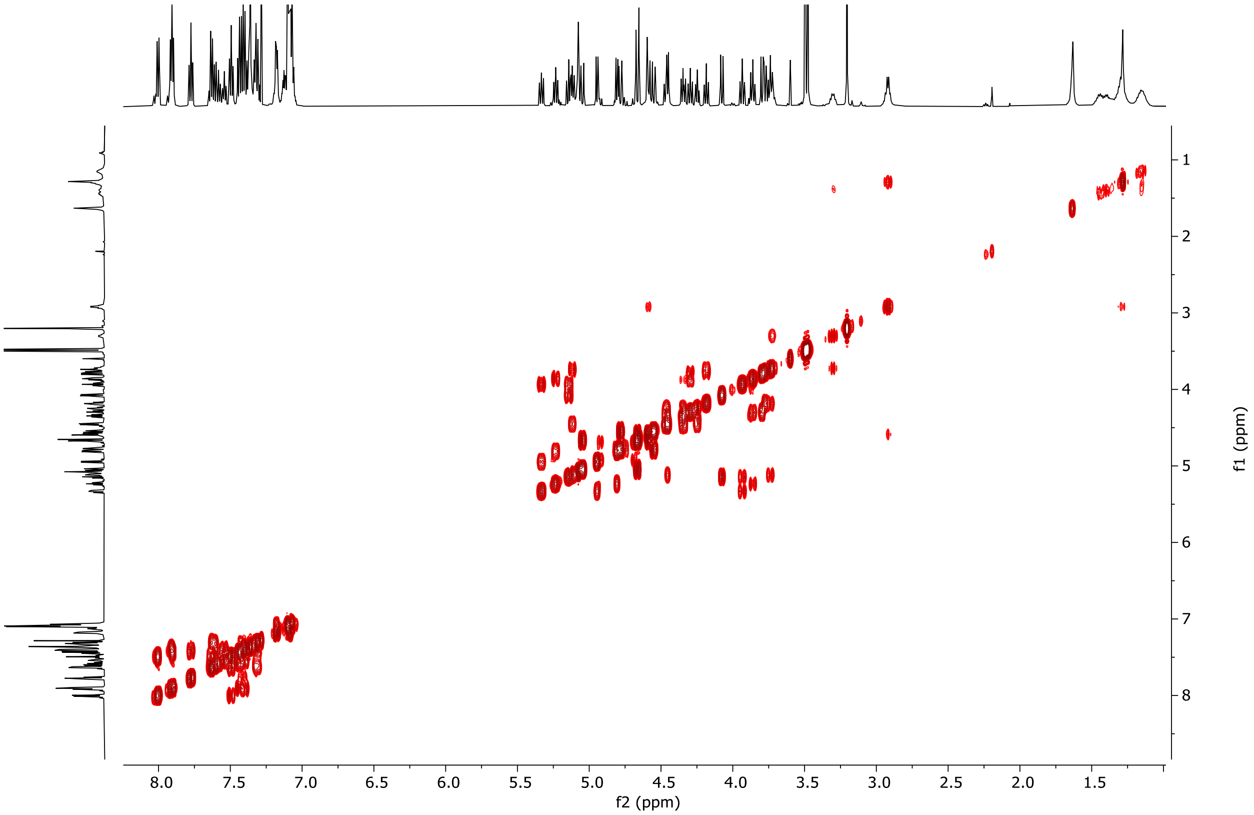

Analytical data for 15

After post-AGA and purification compound **15** was obtained as a white solid (1.77 mg, 55%). **^{1}H NMR** (700 MHz, D_2O): δ 4.59 (d, *J* = 7.9 Hz, 1H), 4.55 (dd, *J* = 7.9, 3.9 Hz, 2H), 3.98 – 3.92 (m, 2H), 3.89 (d, *J* = 9.7 Hz, 1H), 3.80 (d, *J* = 1.6 Hz, 1H), 3.78 – 3.72 (m, 3H), 3.71 – 3.65 (m, 2H), 3.56 (dd, *J* = 8.6, 6.7 Hz, 2H), 3.45 – 3.37 (m, 3H), 3.05 (t, *J* = 7.5 Hz, 2H), 1.79 – 1.68 (m, 4H), 1.52 (m, *J* = 15.1, 7.2 Hz, 2H). **^{13}C NMR** (176 MHz, D2O): δ 175.53, 175.02, 174.93, 102.36, 102.09, 101.91, 80.78, 75.84, 75.61, 75.20, 75.17, 74.37, 74.10, 73.01, 72.67, 72.53, 71.58, 69.98, 39.29, 28.01, 26.15, 21.87. **HRMS QTOF-MS**: calcd. $C_{23}H_{36}NO_{19}^{-}$ for $[M\text{-}H]^{-}$ 630.1887, found 630.1885.

^{1}H NMR of 15 (700 MHz, D_2O)

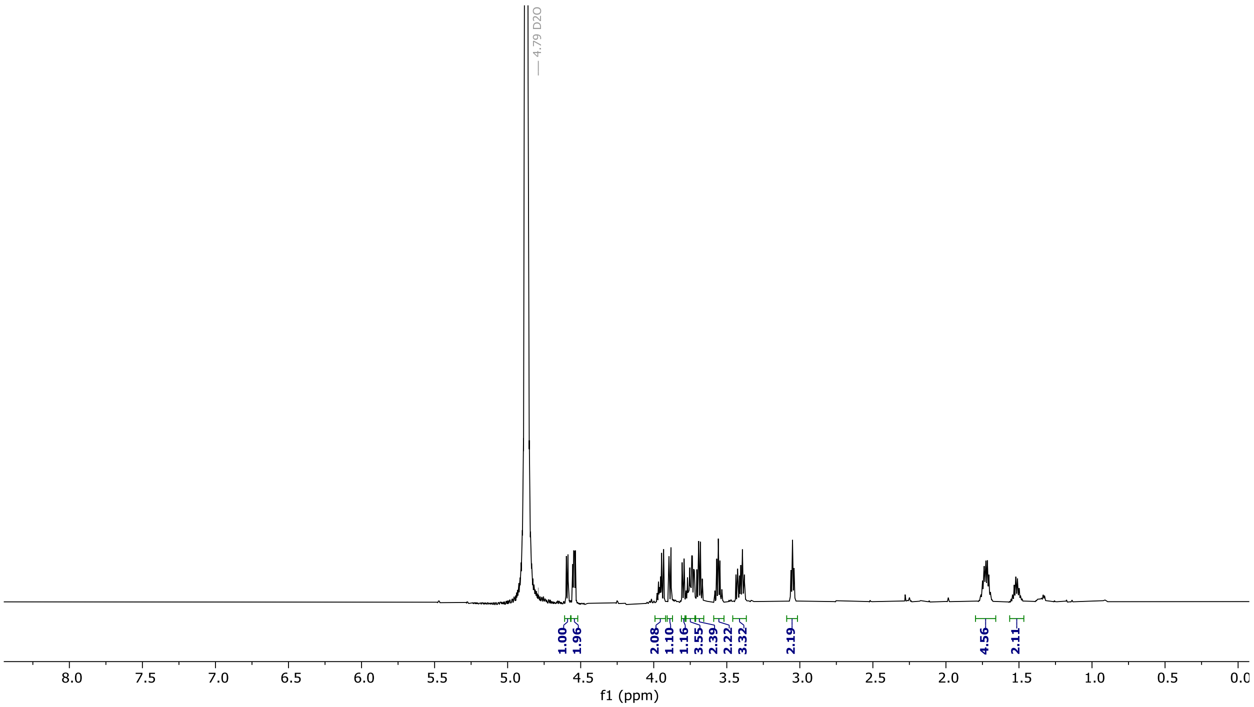

^{13}C NMR of 15 (176 MHz, D_2O)

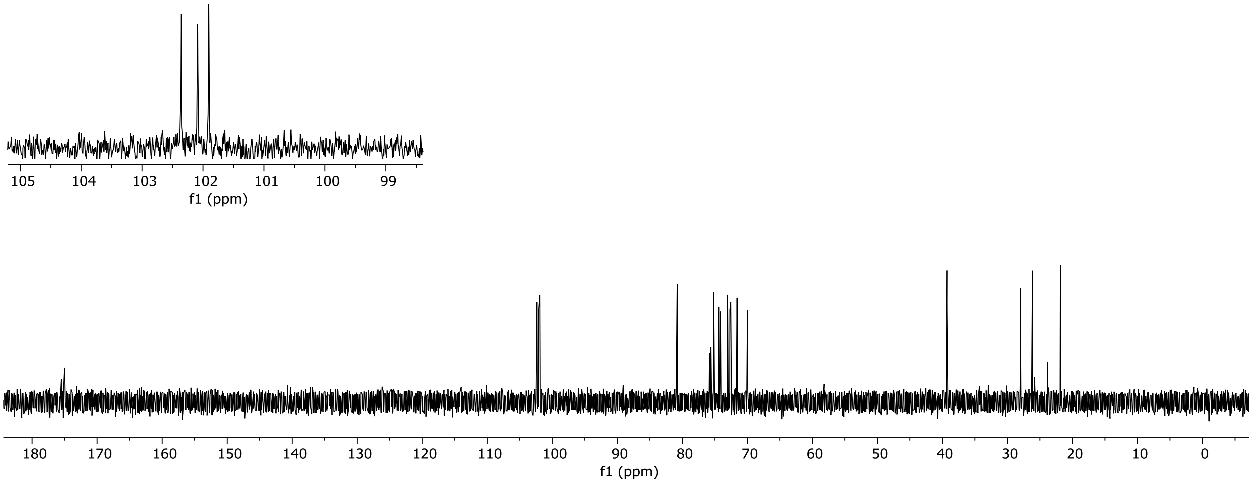

DEPT-135 NMR of 15 (176 MHz, D_2O)

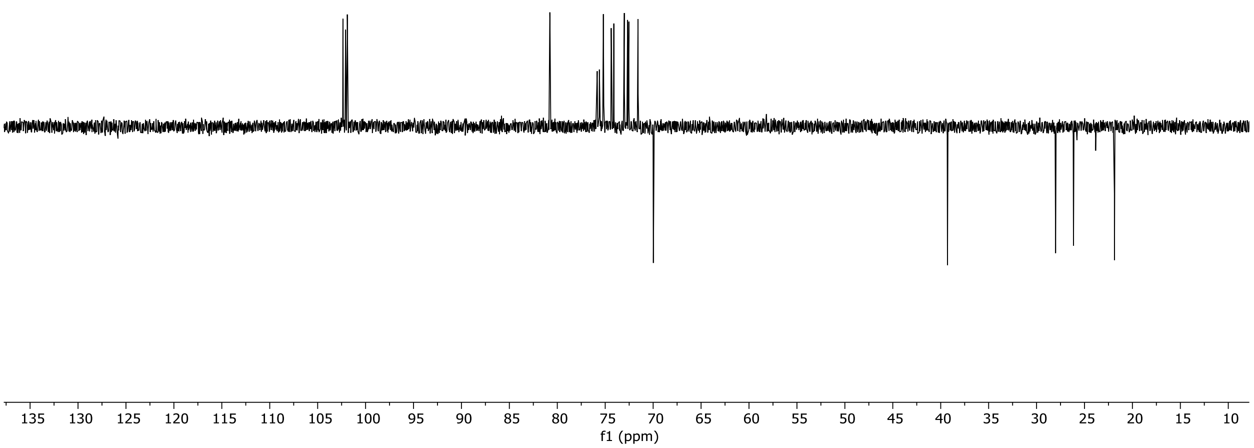

^{1}H-^{13}C HSQC NMR of 15 (700 MHz, D_2O)

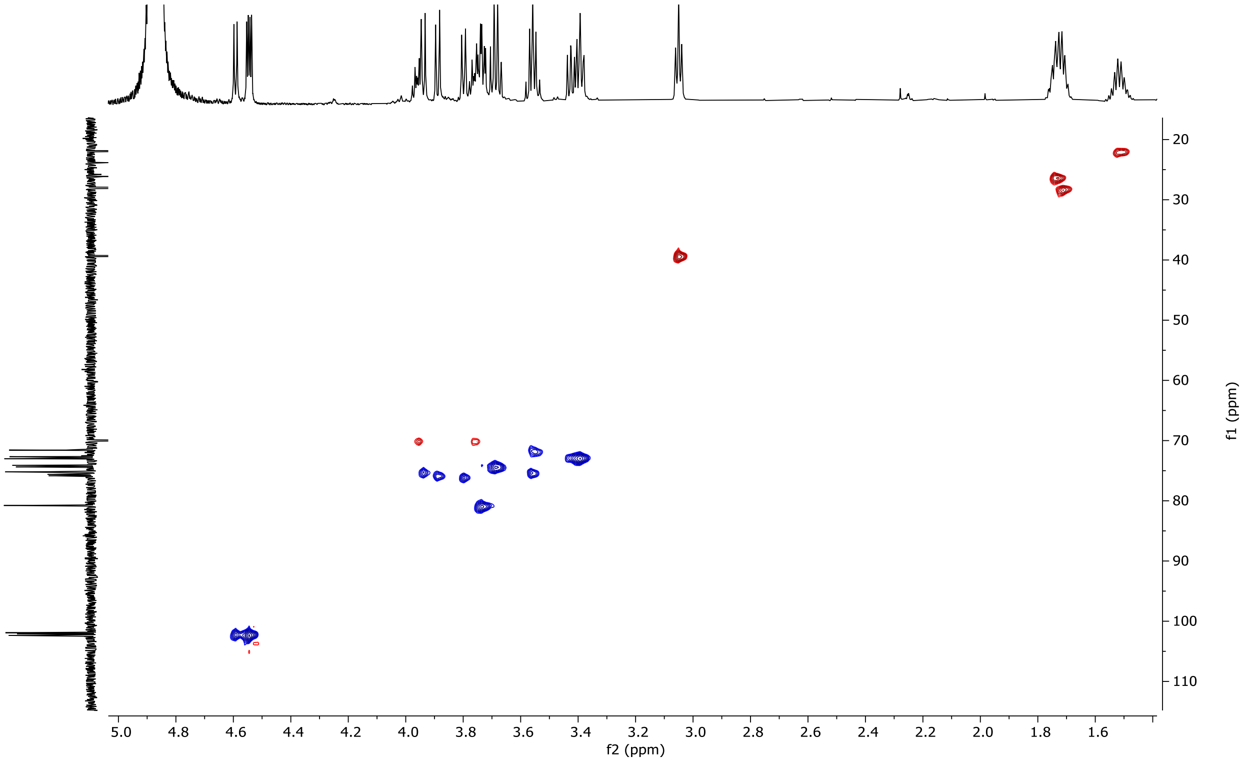

^1H-^{13}C coupled HSQC NMR of 15 (400 MHz, D_2O)

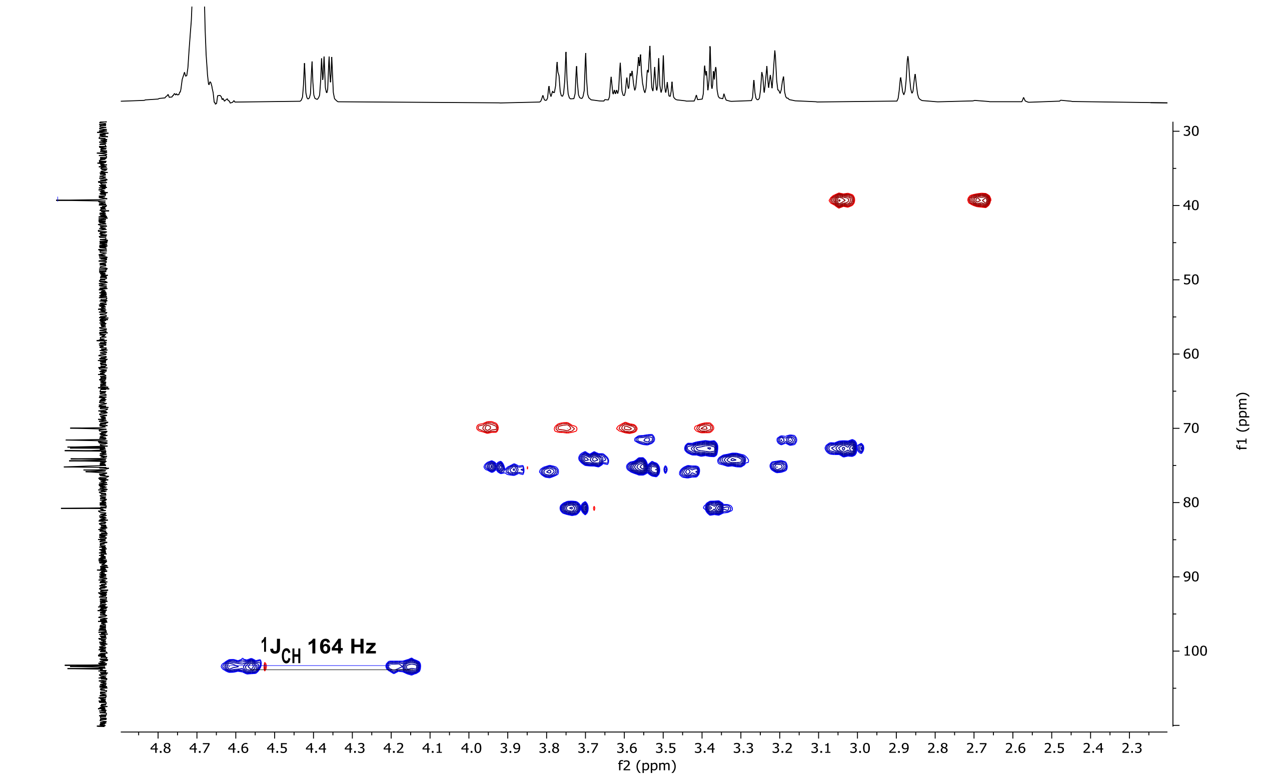

^1H-^1H COSY NMR of 15 (400 MHz, D_2O)

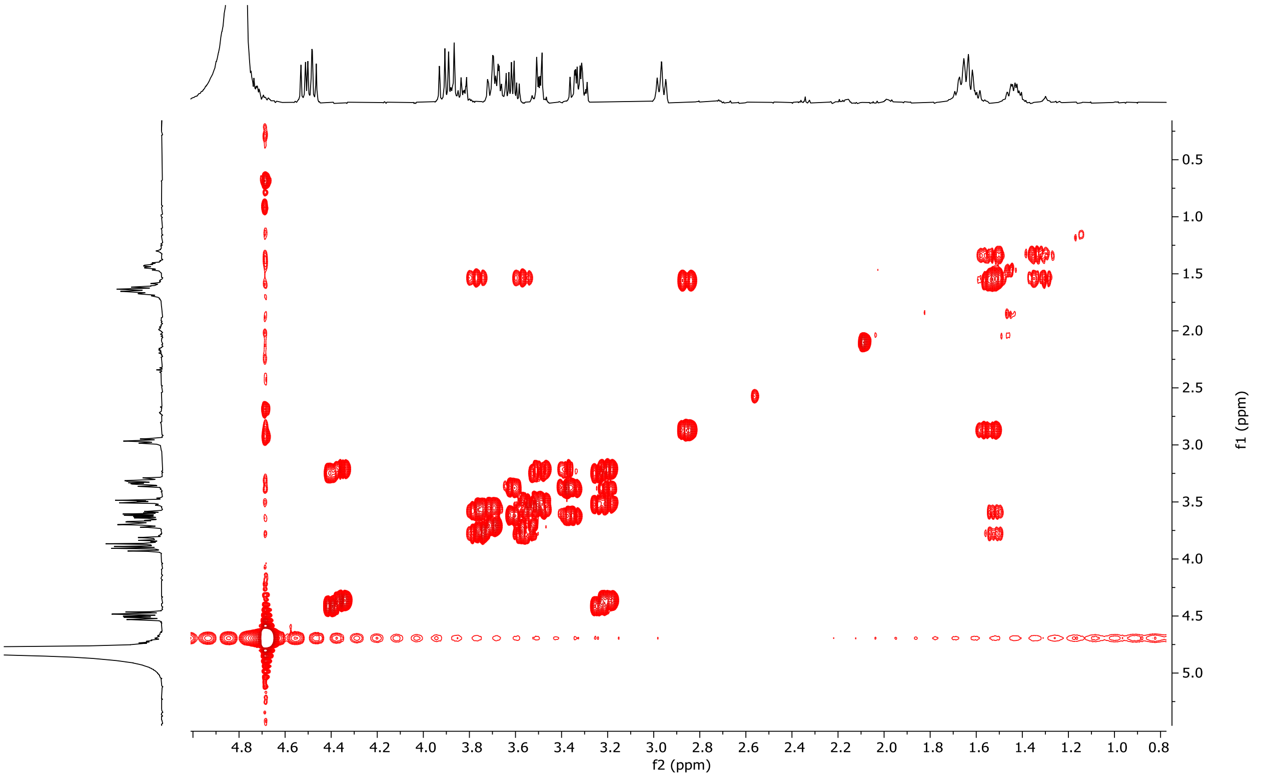

4.4 Synthesis and analytical data of 17

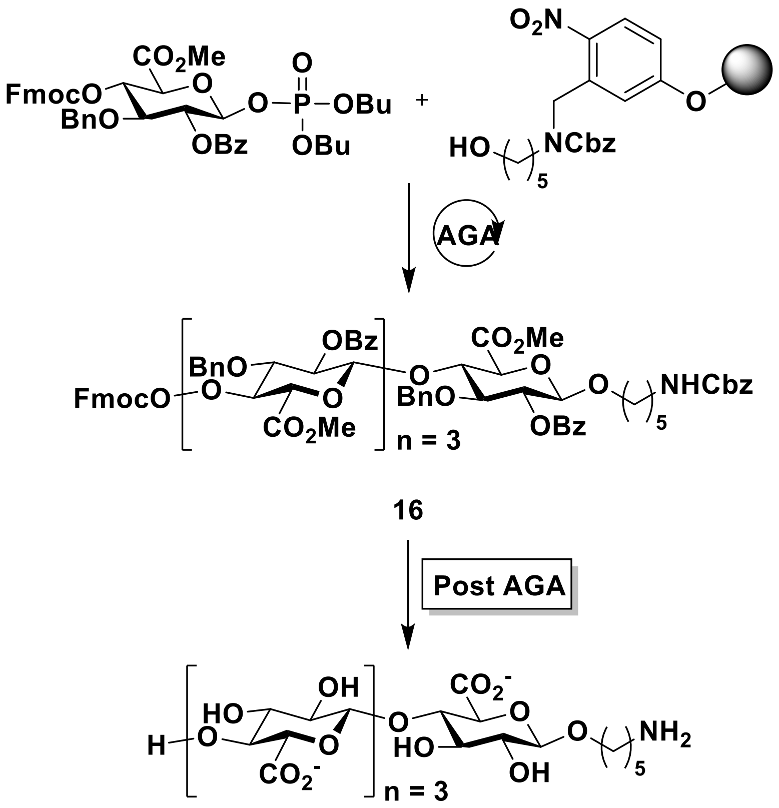

16

17

Step		Modules	Notes
AGA		**A**	
	**BB-7**	**3x (B, 2x D, E, G)**	**D:** (-15 °C for 30 min, 0 °C for 30 min)
	**BB-7**	**B, 2xD**	**D:** (-15 °C for 30 min, 0 °C for 30 min)
Post-AGA	Photocleavage	**H**	
	Purification	$\mathbf{L_{1P}}$	
	Hydrolysis	**J**	Twelve hours (2mL, 1:1 H_2O/THF)
	Hydrogenolysis	**K**	Twelve hours (2.5 mL, 4:1 H_2O/^{t}BuOH)
	Purification	$\mathbf{L_{1D}}$	

Analytical data for 16

After AGA, photocleavage and NP-purification compound **16** was obtained as a colorless oil (10.4 mg, 35%). **^{1}H NMR** (400 MHz, $CDCl_3$): δ 7.98 – 7.94 (m, 2H), 7.91 – 7.83 (m, 6H), 7.75 (dd, *J* = 5.2, 1.0 Hz, 2H), 7.62 – 7.51 (m, 6H), 7.51 – 7.48 (m, 1H), 7.47 – 7.35 (m, 11H), 7.35 – 7.27 (m, 8H), 7.16 – 7.13 (m, 2H), 7.09 – 7.00 (m, 19H), 5.30 (dd, *J* = 9.4, 8.0 Hz, 1H), 5.22 – 5.00 (m, 8H), 4.92 (d, *J* = 7.9 Hz, 1H), 4.82 – 4.70 (m, 4H), 4.66 – 4.60 (m, 2H), 4.57 – 4.39 (m, 6H), 4.35 – 4.19 (m, 3H), 4.16 – 4.09 (m, 2H), 4.04 (d, *J* = 9.9 Hz, 1H), 3.90 (t, *J* = 9.2 Hz, 1H), 3.85 – 3.78 (m, 1H), 3.76 – 3.65 (m, 6H), 3.46 (s, 3H), 3.42 (s, 3H), 3.17 (s, 3H), 3.11 (s, 3H), 2.88 (q, *J* = 6.8 Hz, 2H), 1.39 (m, 4H), 1.11 (q, *J* = 6.8 Hz, 2H). **^{13}C NMR** (176 MHz, $CDCl_3$): δ 168.57, 168.15, 167.27, 165.26, 165.22, 165.17, 165.00, 156.38, 154.11, 143.45, 143.12, 141.44, 141.39, 138.45, 138.27, 138.26, 137.28, 136.82, 133.47, 133.28, 129.97, 129.87, 129.81, 129.75, 129.65, 128.63, 128.55, 128.47, 128.45, 128.42, 128.36, 128.23, 128.19, 128.12, 128.09, 128.07, 127.97, 127.95, 127.89, 127.86, 127.74, 127.35, 127.30, 127.27, 125.32, 125.21, 120.22, 101.42, 100.95, 100.90, 100.76, 80.29, 79.96, 79.53, 79.03, 78.64, 78.23, 78.06, 75.43, 75.12, 75.01, 74.58, 74.49, 74.37, 74.01, 73.92, 73.13, 73.10, 73.06, 72.64, 70.53, 69.87, 66.61, 52.78, 52.60, 52.52, 52.40, 46.71, 40.88, 29.85, 29.45, 28.84, 23.09. **HRMS QTOF-MS**: calcd. $C_{112}H_{109}NNaO_{33}$ for $[M+Na]^+$ 2018.6780, found 2018.6767.

^{1}H NMR of 16 (400 MHz, $CDCl_3$)

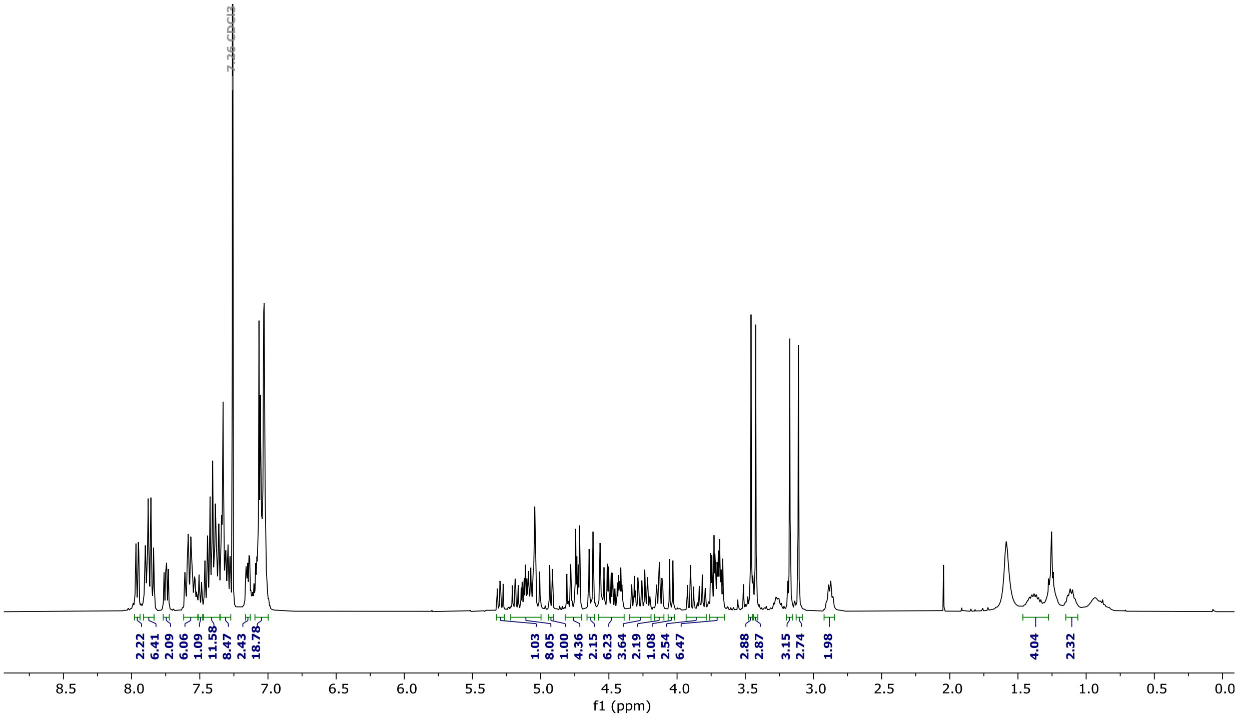

^{13}C NMR of 16 (176 MHz, $CDCl_3$)

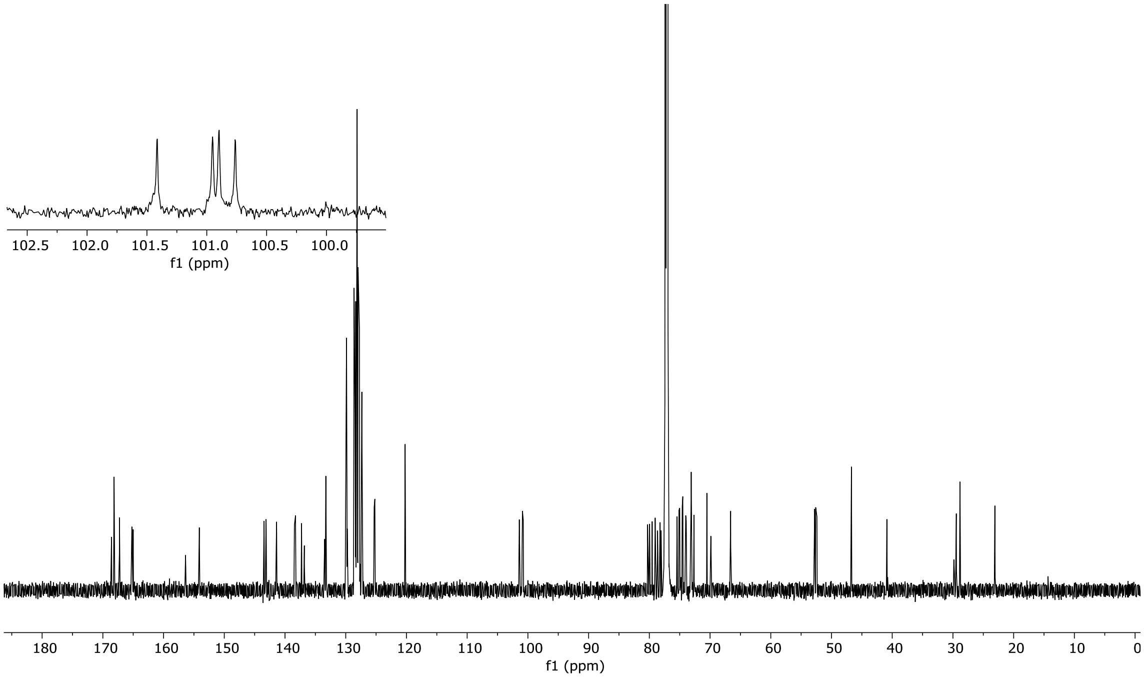

DEPT-135 NMR of 16 (176 MHz, $CDCl_3$)

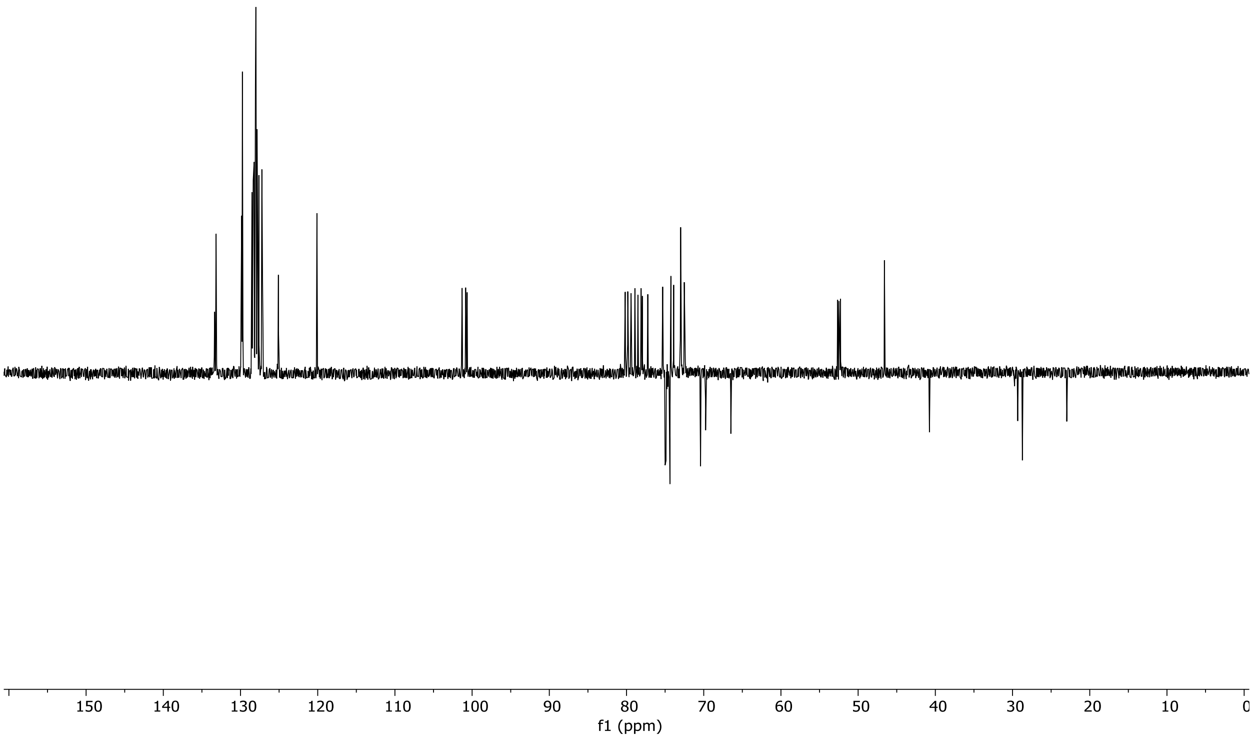

^{1}H-^{13}C HSQC NMR of 16 (700 MHz, $CDCl_3$)

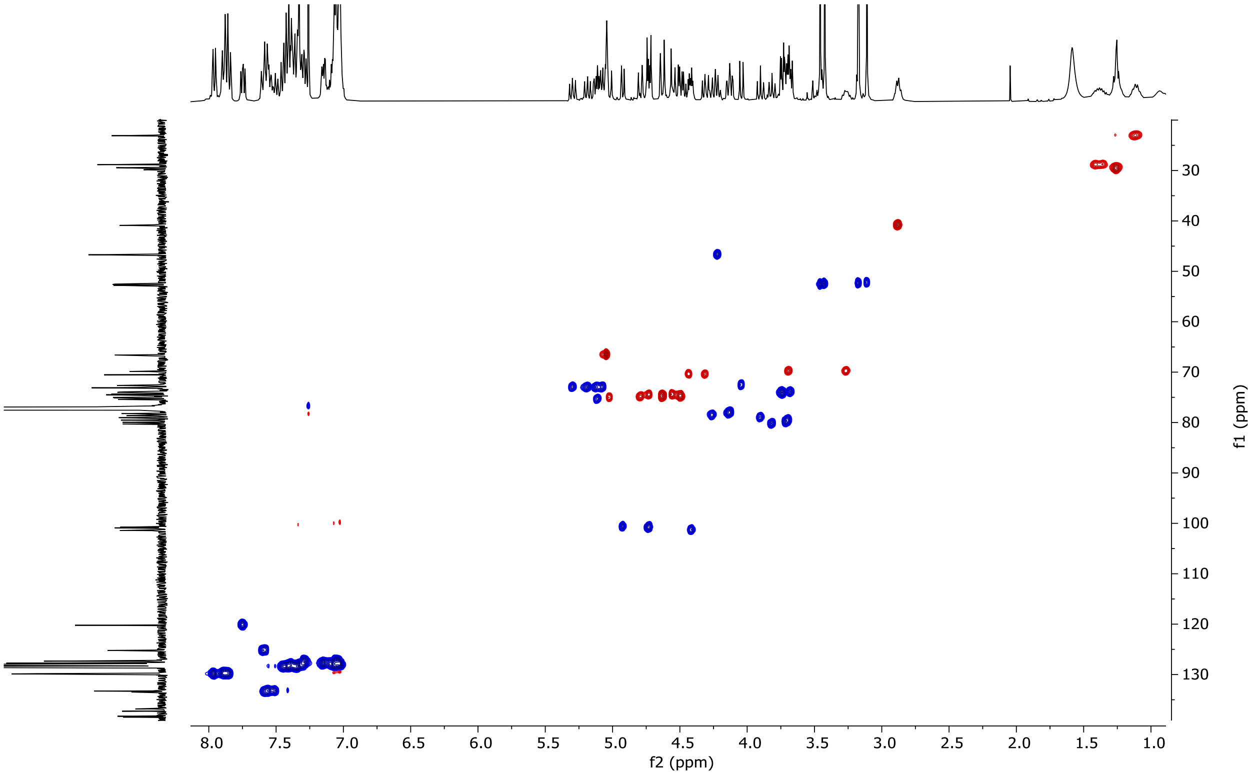

^{1}H-^{13}C coupled HSQC NMR of 16 (400 MHz, $CDCl_3$)

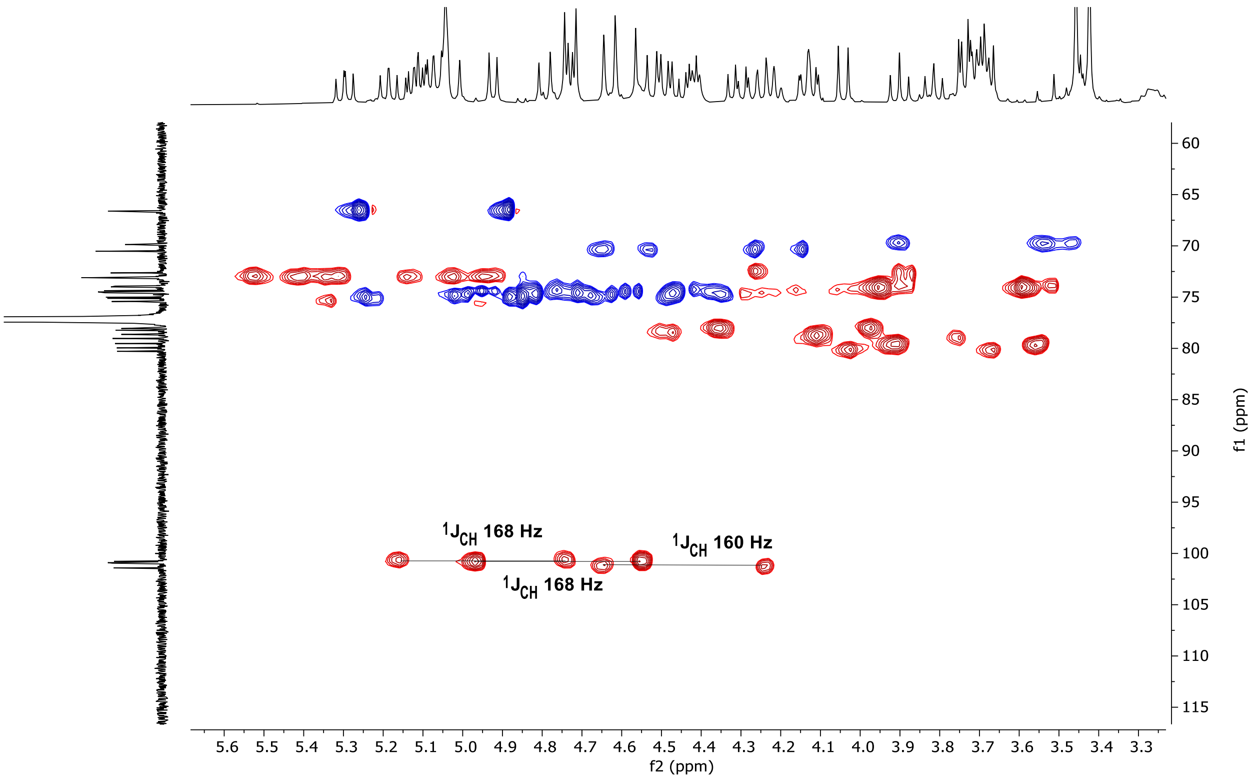

^{1}H-^{1}H COSY NMR of 16 (400 MHz, $CDCl_3$)

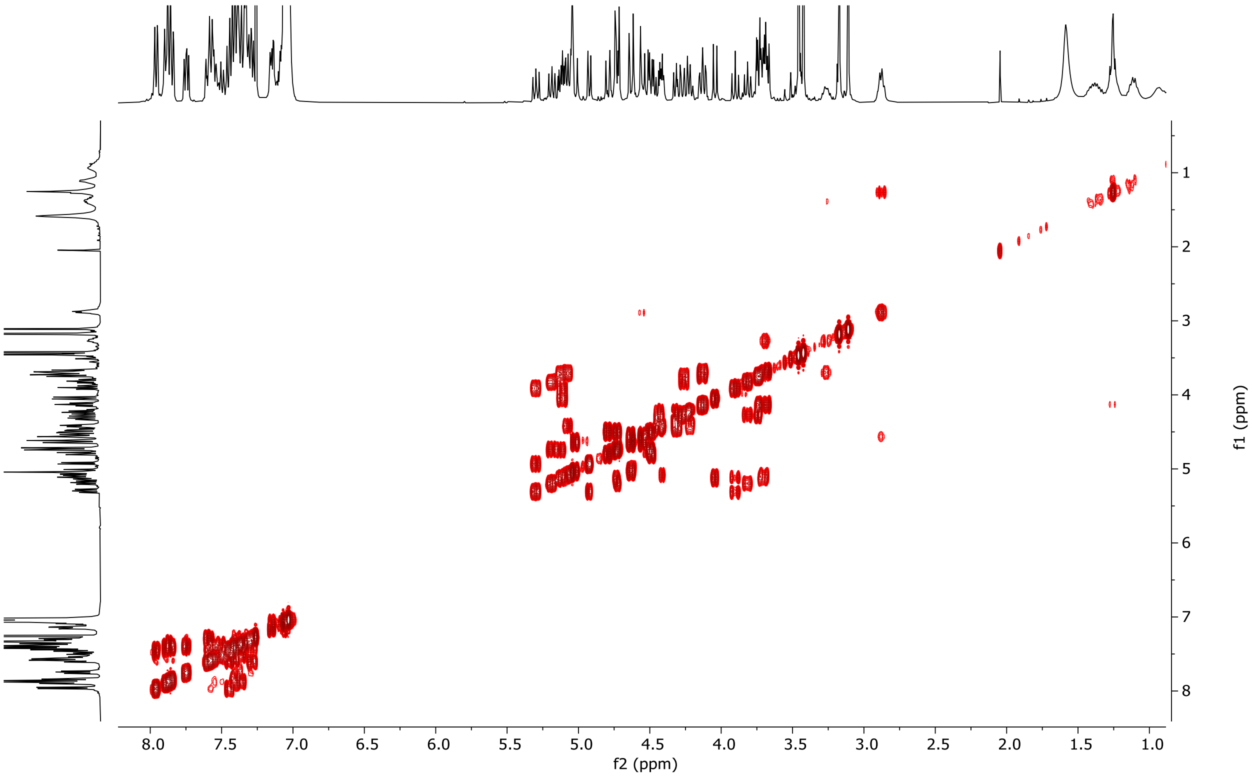

Analytical data for 17

After post-AGA and purification compound **17** was obtained as a white solid (2.1 mg, 43%). **^{1}H NMR** (700 MHz, D_2O) δ 4.56 (dd, J = 7.8, 5.6 Hz, 2H), 4.51 (dd, J = 7.9, 5.8 Hz, 2H), 3.96 – 3.82 (m, 4H), 3.79 – 3.63 (m, 9H), 3.53 (dd, J = 8.7, 7.3 Hz, 2H), 3.42 – 3.33 (m, 4H), 3.02 (t, J = 7.5 Hz, 2H), 1.73 – 1.67 (m, 4H), 1.49 (d, J = 8.0 Hz, 2H). **^{13}C NMR** (176 MHz, D_2O) δ 175.24, 175.24,174.81, 174.80, 102.49, 102.25, 102.19, 101.97, 80.95, 80.91, 80.77, 75.86, 75.70, 75.28, 75.26, 74.48, 74.23, 74.13, 73.10, 72.73, 72.61, 71.65, 70.01, 39.38, 28.07, 26.19, 21.93. **HRMS QTOF-MS:** calcd. $C_{29}H_{44}NO_{25}^-$ for $[M-H]^-$ 806.2208, found 806.2203.

^1H NMR of 17 (400 MHz, D_2O)

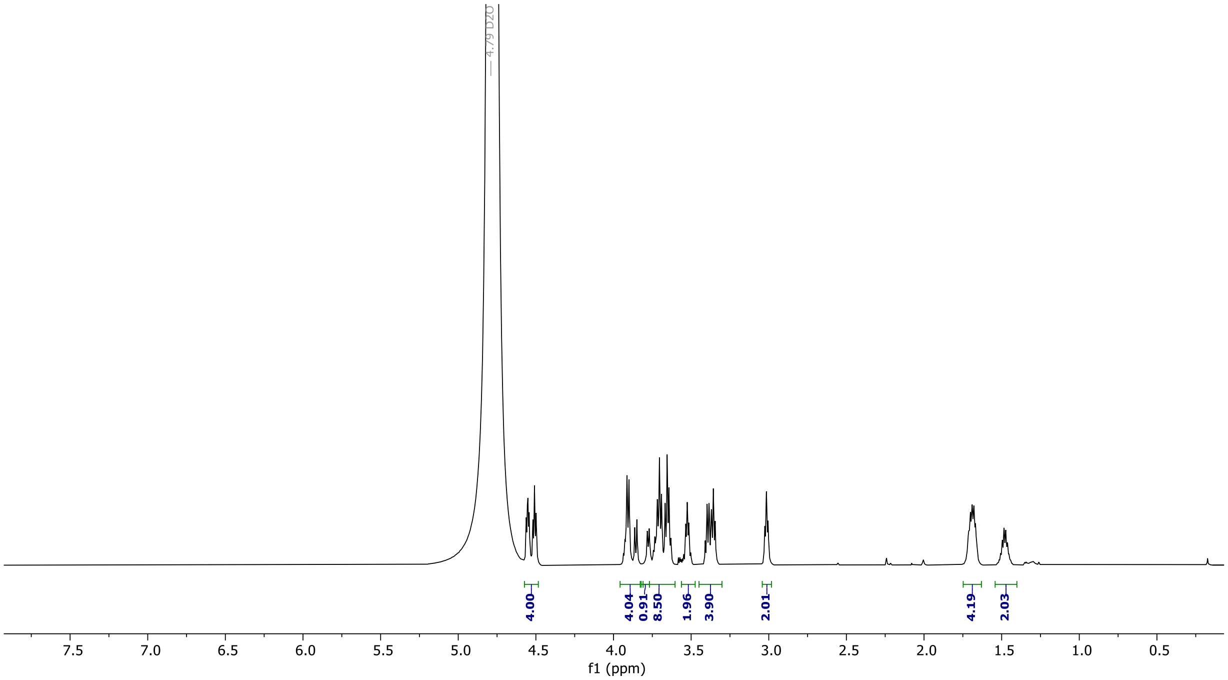

DEPT-135 NMR of 17 (176 MHz, D_2O)

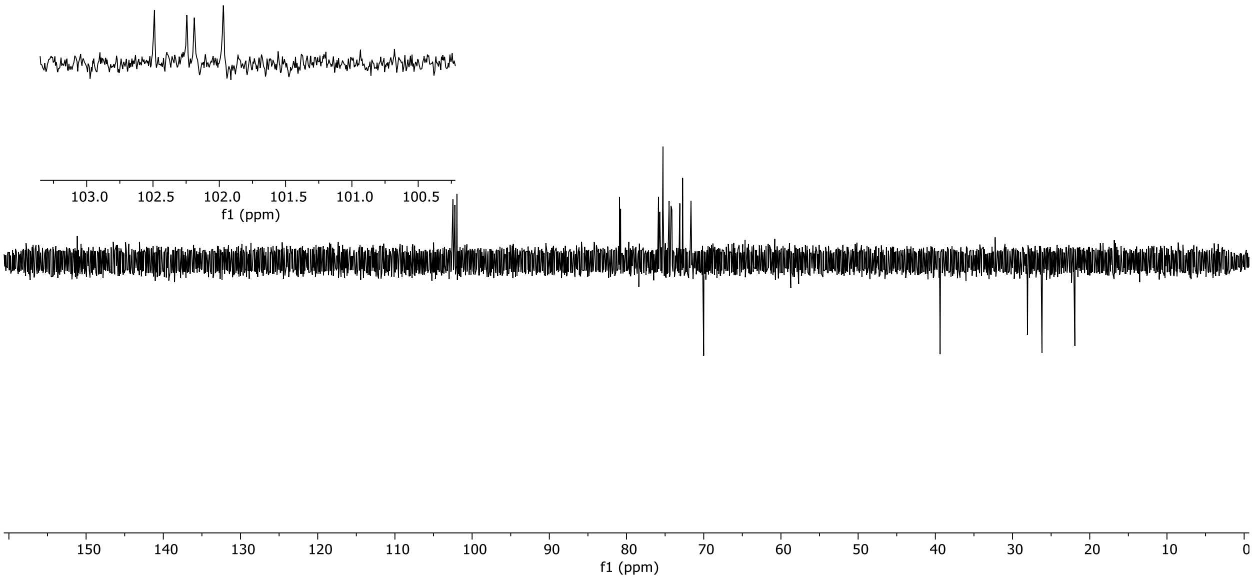

^{1}H-^{13}C HSQC NMR 17 (D_2O)

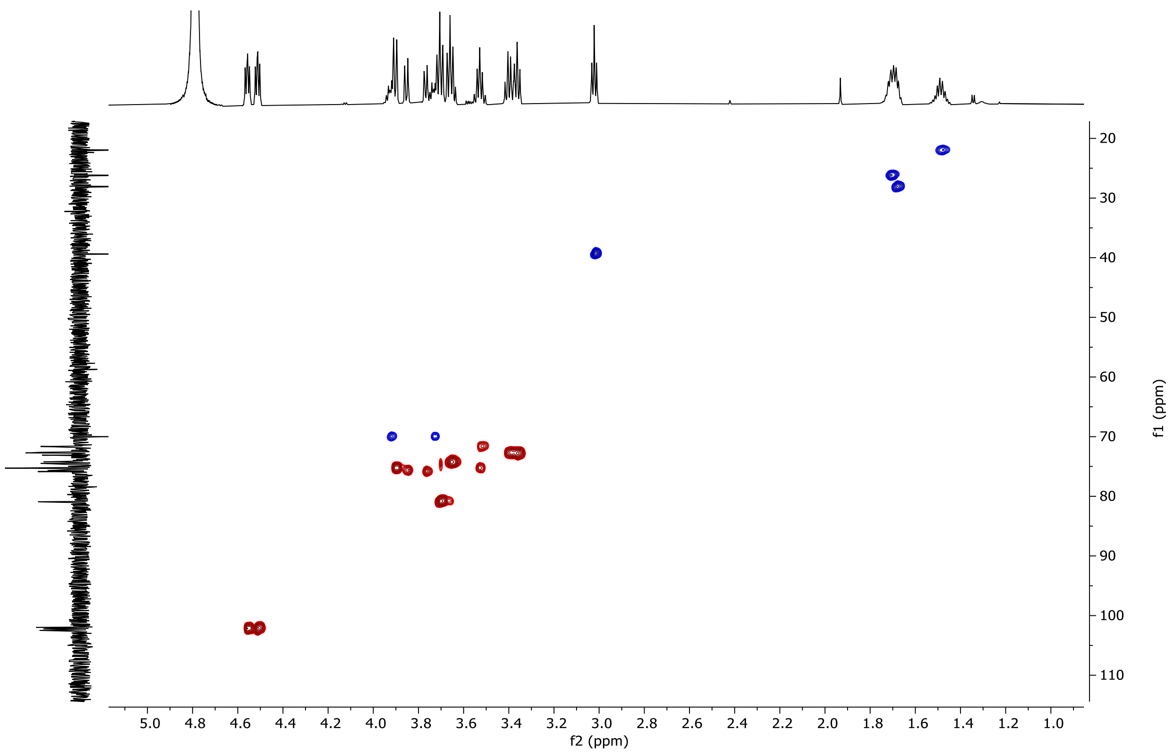

^{1}H-^{13}C coupled HSQC NMR of 17 (700 MHz, D_2O)

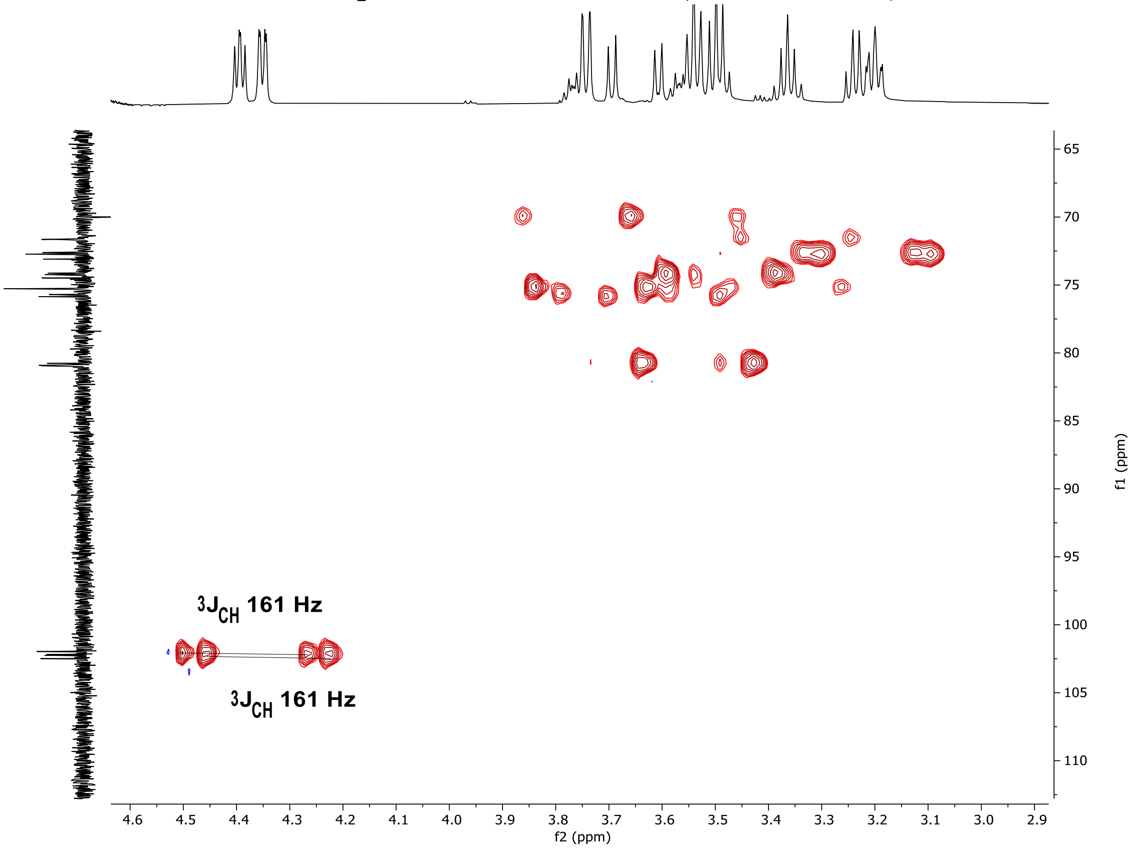

^{1}H-^{1}H COSY of 17 (400 MHz, D_2O)

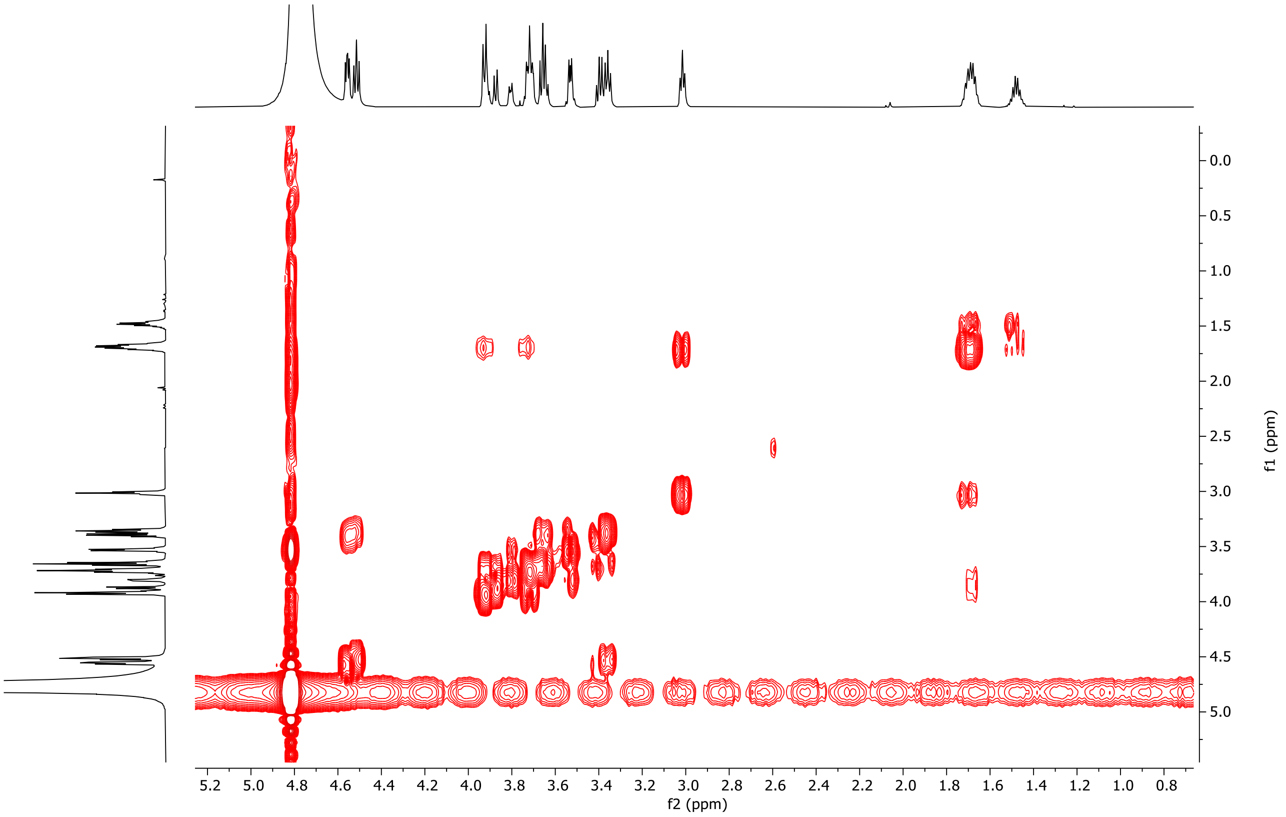

4.5 Synthesis and analytical data of 19

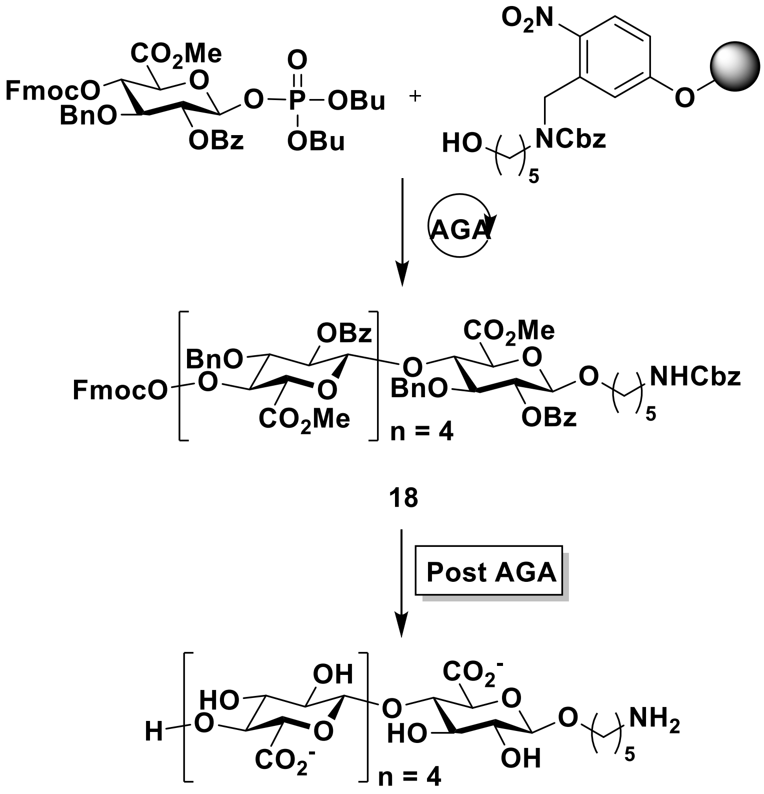

19

Step		Modules	Notes
AGA		**A**	
	**BB-7**	**4x (B, 2x D, E, G)**	**D:** (-15 °C for 30 min, 0 °C for 30 min)
	**BB-7**	**B, 2xD**	**D:** (-15 °C for 30 min, 0 °C for 30 min)
Post-AGA	Photocleavage	**H**	
	Purification	**L_{1P}**	
	Hydrolysis	**J**	18 h (2 mL, 1:1 H_2O/THF)
	Hydrogenolysis	**K**	24 h (2.5 mL, 4:1 H_2O/^{t}BuOH)
	Purification	**K_{1D}**	

Analytical data for 18

After AGA, photocleavage and NP-purification compound **18** was obtained as a colorless oil (12.5 mg, 35%). **^{1}H NMR** (400 MHz, $CDCl_3$): δ 7.98 – 7.92 (m, 2H), 7.91 – 7.82 (m, 8H), 7.75 (dd, *J* = 7.6, 5.2 Hz, 2H), 7.62 – 7.48 (m, 7H), 7.46 – 7.27 (m, 20H), 7.14 (dd, *J* = 6.9, 3.2 Hz, 2H), 7.09 – 6.99 (m, 23H), 5.30 (dd, *J* = 9.4, 7.9 Hz, 1H), 5.18 (dd, *J* = 9.1, 7.9 Hz, 1H), 5.14 – 4.98 (m, 7H), 4.93 (d, *J* = 7.9 Hz, 1H), 4.82 – 4.67 (m, 6H), 4.66 – 4.60 (m, 2H), 4.58 – 4.39 (m, 7H), 4.34 – 4.20 (m, 3H), 4.17 – 4.08 (m, 3H), 4.04 (d, *J* = 9.8 Hz, 1H), 3.90 (t, *J* = 9.2 Hz, 1H), 3.81 (t, *J* = 8.7 Hz, 1H), 3.70 (m, 8H), 3.45 (s, 3H), 3.42 (s, 3H), 3.15, 3.22 (m, 2H), (s, 3H), 3.11 (s, 3H), 3.09 (s, 3H), 2.88 (q, *J* = 6.8 Hz, 2H), 1.28 (s, 4jH), 1.11 (q, *J* = 7.6 Hz, 2H). **^{13}C NMR** (151 MHz, $CDCl_3$): δ 168.59, 168.16, 168.11, 167.26, 165.28, 165.22, 165.19, 165.01, 156.39, 154.12, 143.46, 143.14, 141.45, 141.40, 133.46, 133.30, 133.24 (d, *J* = 5.4 Hz), 129.88, 129.85, 129.82, 129.69, 128.63, 128.55, 128.47, 128.43, 128.39, 128.35, 128.22, 128.18, 128.13, 128.11, 128.09, 128.07, 127.97, 127.95, 127.90, 127.87, 127.86, 127.74, 127.63, 127.35, 127.29, 127.28, 127.26, 125.32, 125.21, 120.21, 101.42, 100.93, 100.91, 100.77, 80.32, 79.99, 79.55, 79.08, 78.64, 78.21, 78.16, 78.13, 75.44, 75.09, 75.05, 74.97, 74.57, 74.50, 74.39, 74.05, 74.01, 73.16, 73.14, 73.08, 72.66, 69.86, 66.58, 52.76, 52.58, 52.48, 52.45, 52.39, 46.74, 40.90, 29.84, 29.46, 28.85, 23.10, 22.84. **HRMS QTOF-MS**: calcd. $C_{133}H_{129}NNaO_{40}$ for $[m/z+Na]^+$ 2403.8022, found 2403.8042.

NP-HPLC of crude 18 (ELSD trace, Method K_{1D} tR = 23.73 min)

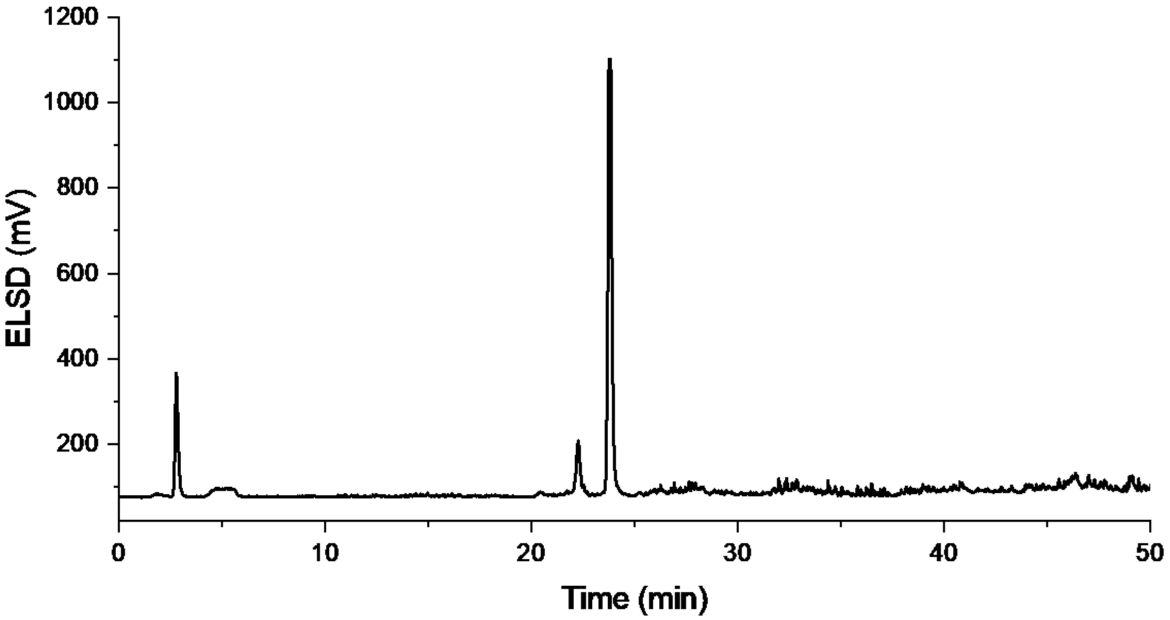

NP-HPLC of pure 18 (ELSD trace, Method K_{1D} t_R = 23.73 min)

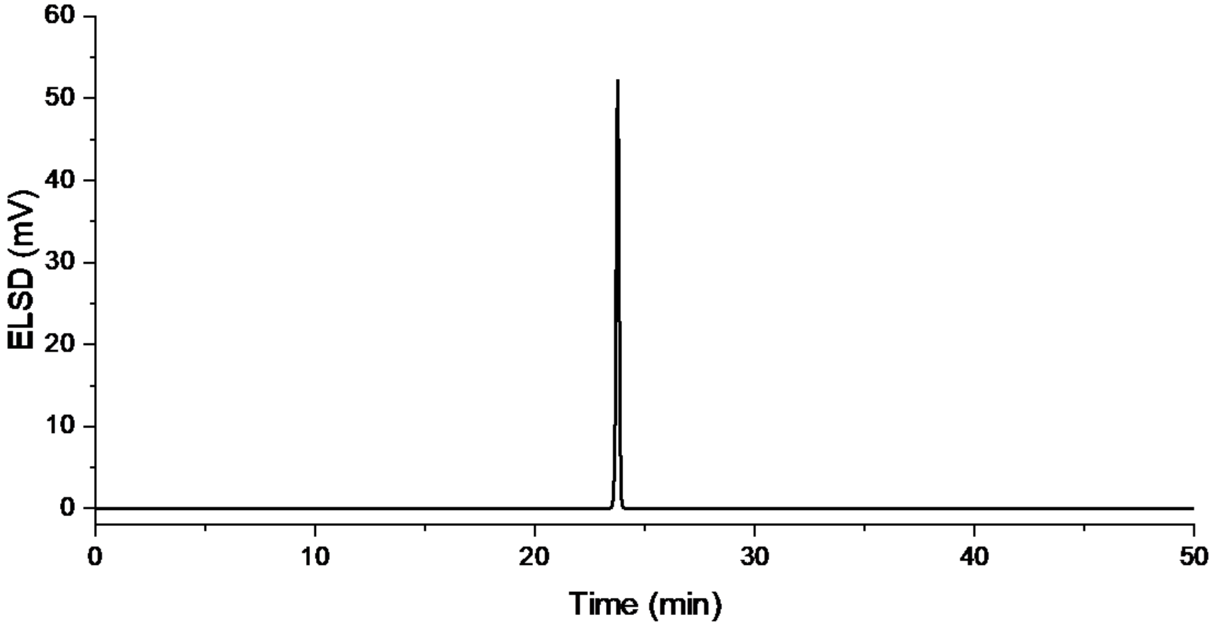

^{1}H NMR of 18 (400 MHz, $CDCl_3$)

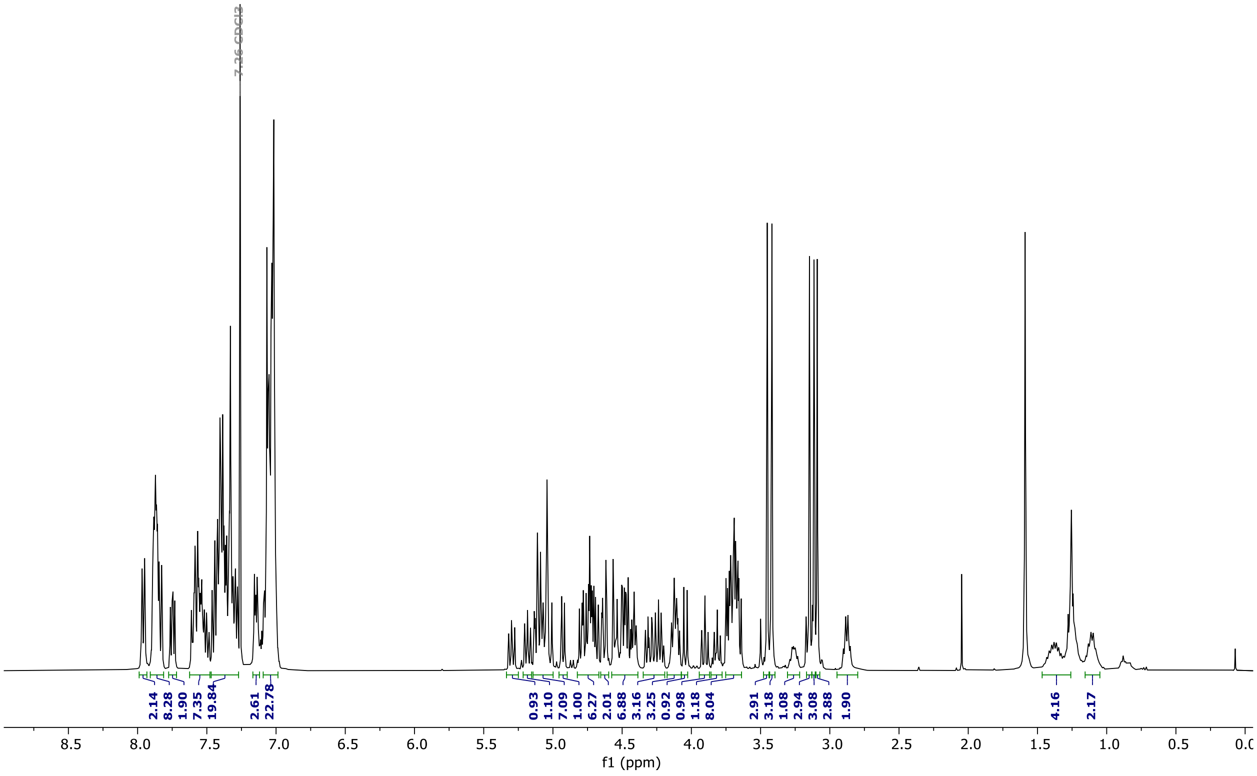

^{13}C NMR of 18 (151 MHz, CDCl$_3$)

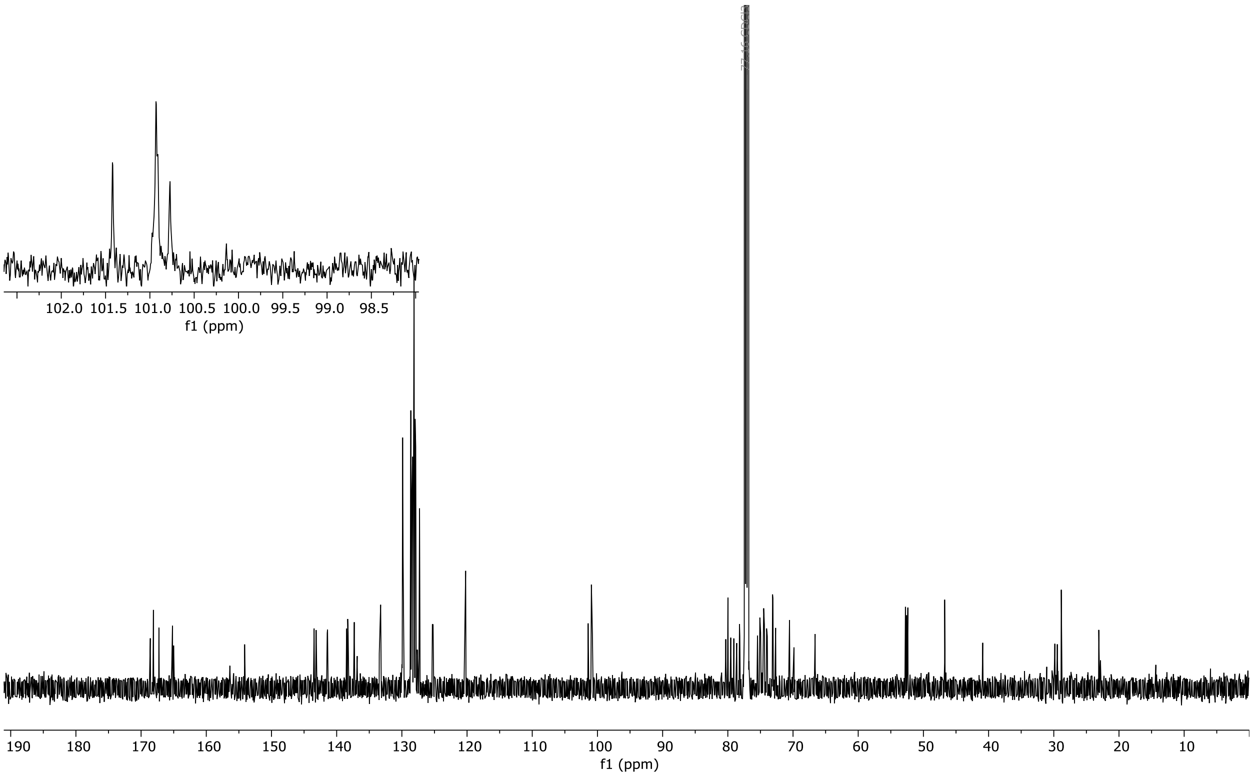

DEPT-135 NMR of 18 (151 MHz, CDCl$_3$)

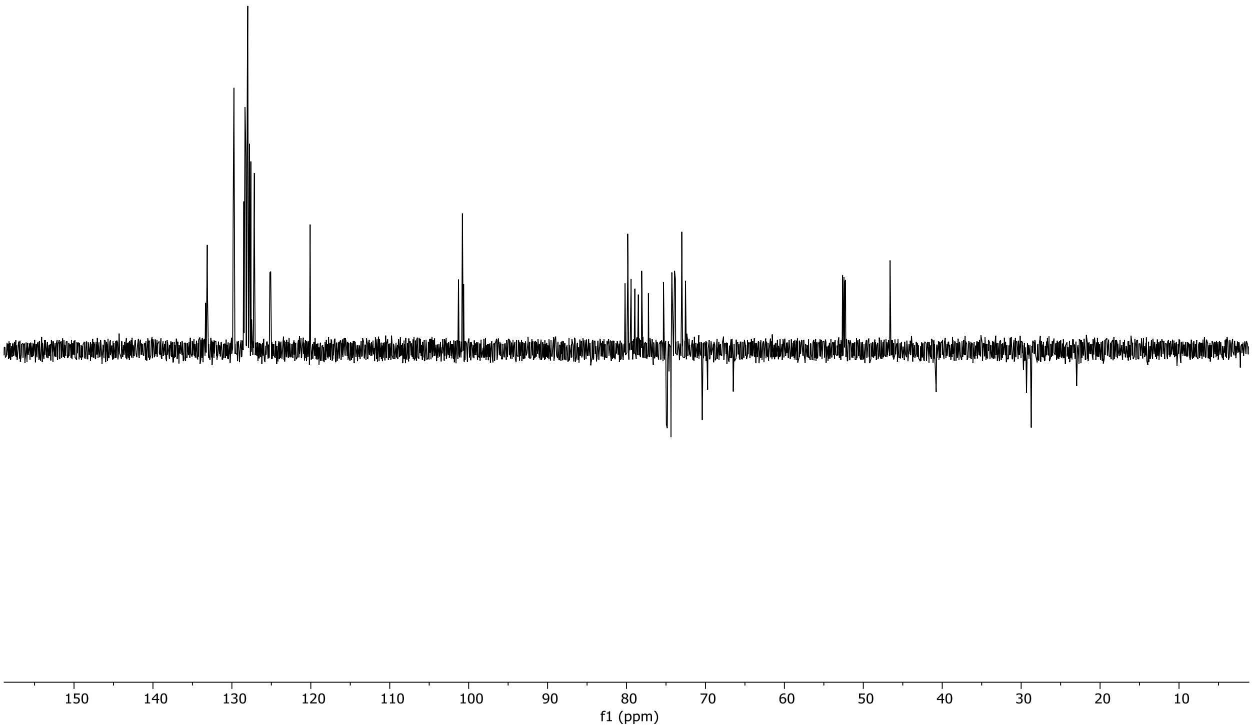

^{1}H-^{13}C HSQC NMR of 18 (600 MHz, $CDCl_3$)

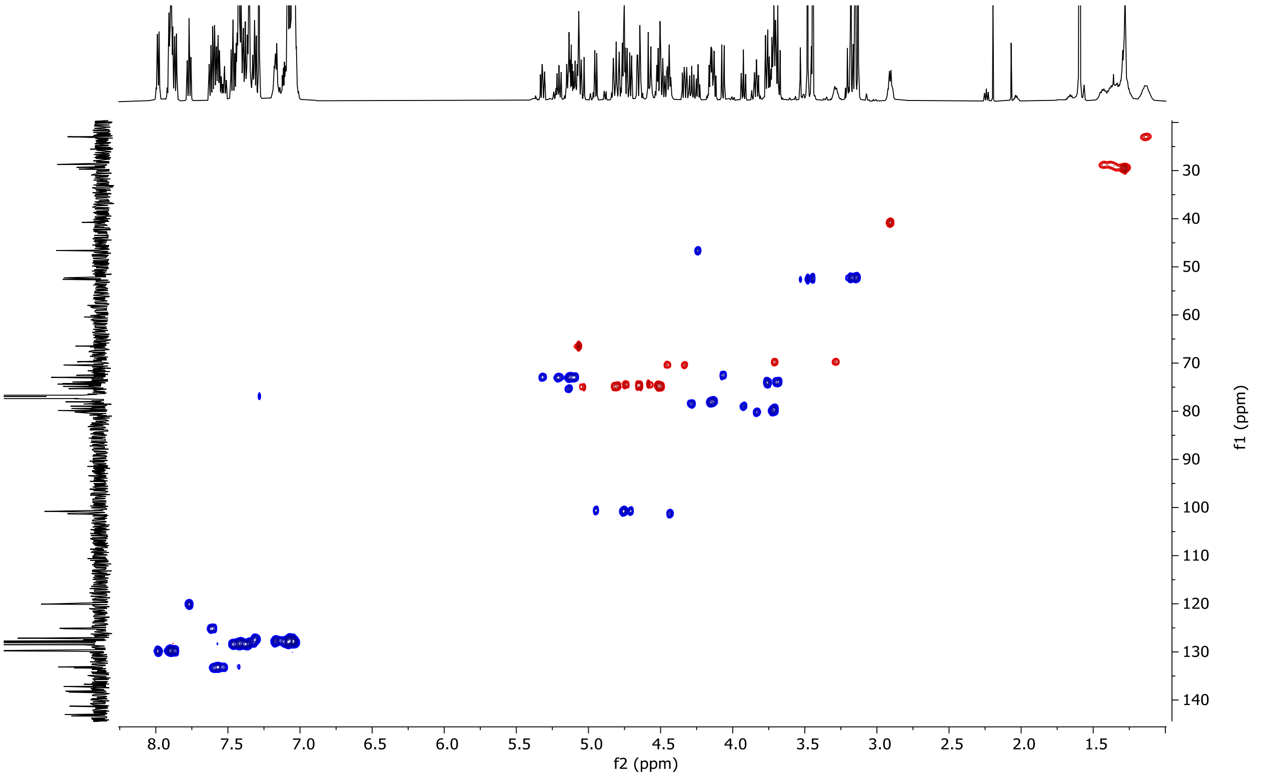

^{1}H-^{13}C coupled HSQC NMR of 18 (400 MHz, $CDCl_3$)

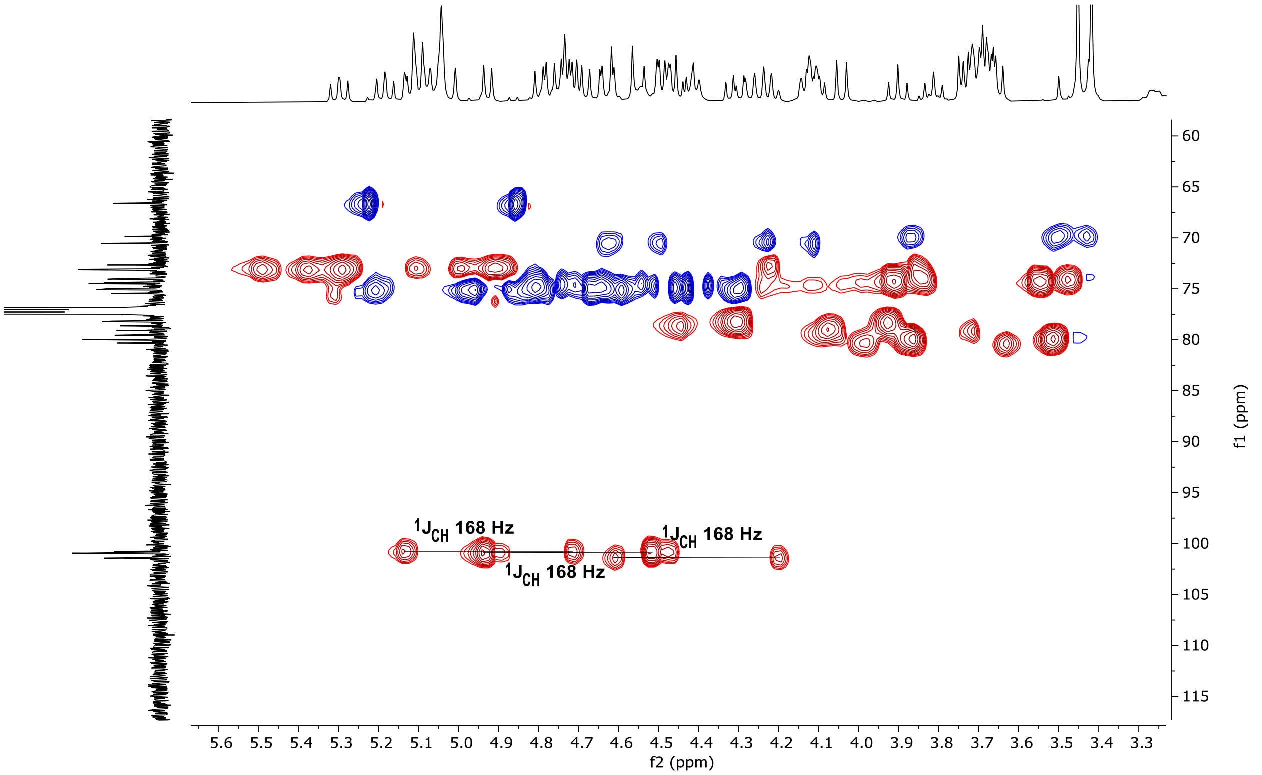

^1H-^1H COSY NMR of 18 (600MHz, $CDCl_3$)

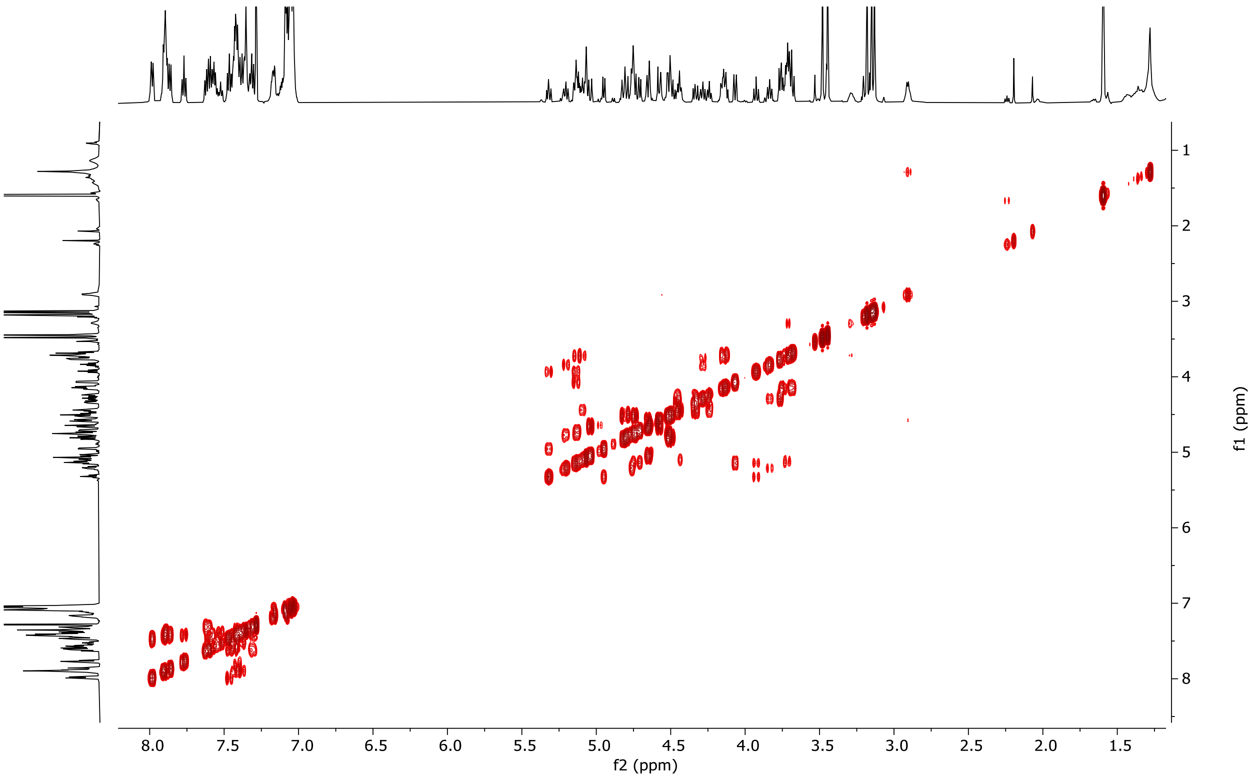

Analytical data for 19

After post-AGA and purification compound **19** was obtained as a white solid (2.1 mg, 43%). **^{1}H NMR** (400 MHz, D_2O): δ 4.54 – 4.40 (m, 5H), 3.82 (m, 5H), 3.74 – 3.55 (m, 10H), 3.52 – 3.41 (m, 2H), 3.40 – 3.25 (m, 5H), 2.96 (t, *J* = 7.5 Hz, 2H), 1.71 – 1.56 (m, 4H), 1.43 (dt, *J* = 7.4, 3.3 Hz, 2H). **^{13}C NMR** (151 MHz, D_2O): δ 175.54, 175.03, 174.94, 174.88, 102.38, 102.17, 102.09, 101.88, 80.81, 80.79, 80.70, 80.66, 75.81, 75.60, 75.17, 74.36, 74.10, 74.03, 74.01, 73.00, 72.64, 72.62, 72.51, 71.57, 69.95, 39.28, 27.99, 26.13, 21.86. **HRMS QTOF-MS**: calcd. $C_{35}H_{52}NO_{31}^-$ for $[M-H]^-$ 982.2529, found 982.2505.

^{1}H NMR of 19 (400 MHz, D_2O)

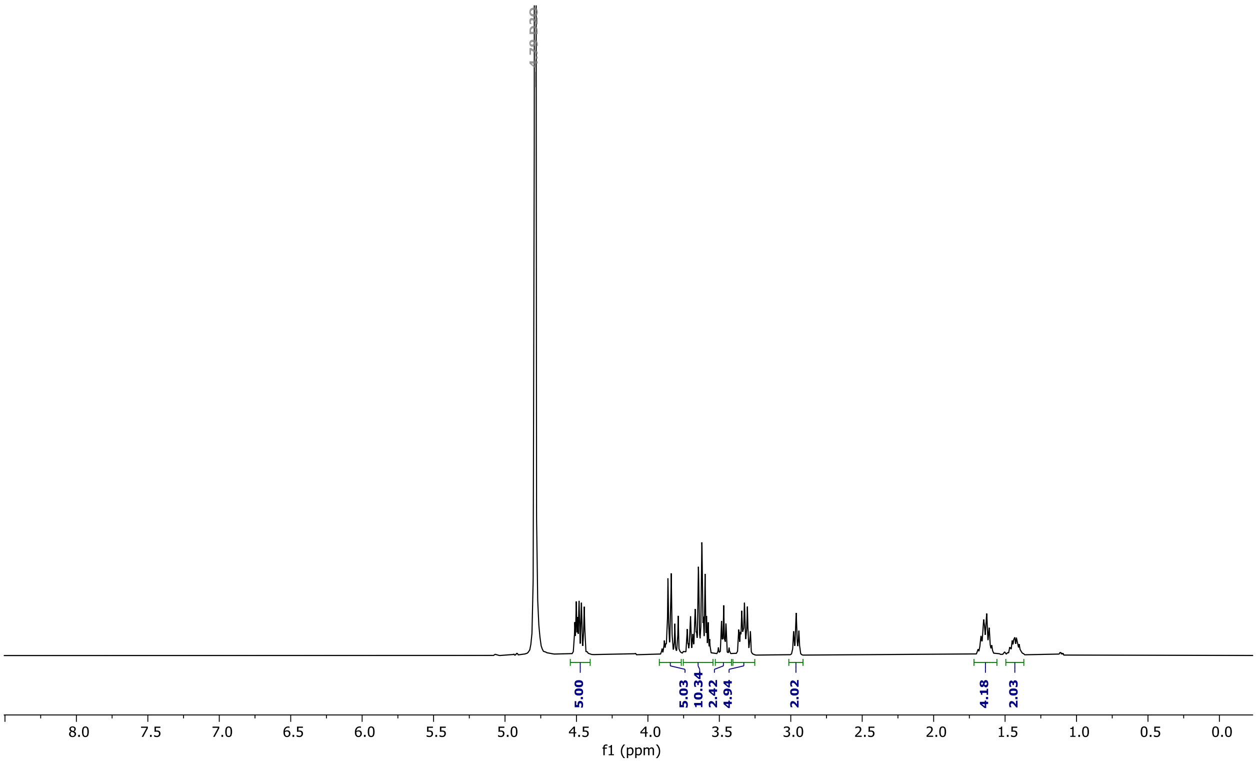

^{13}C NMR of 19 (151 MHz, 151 MHz, D_2O)

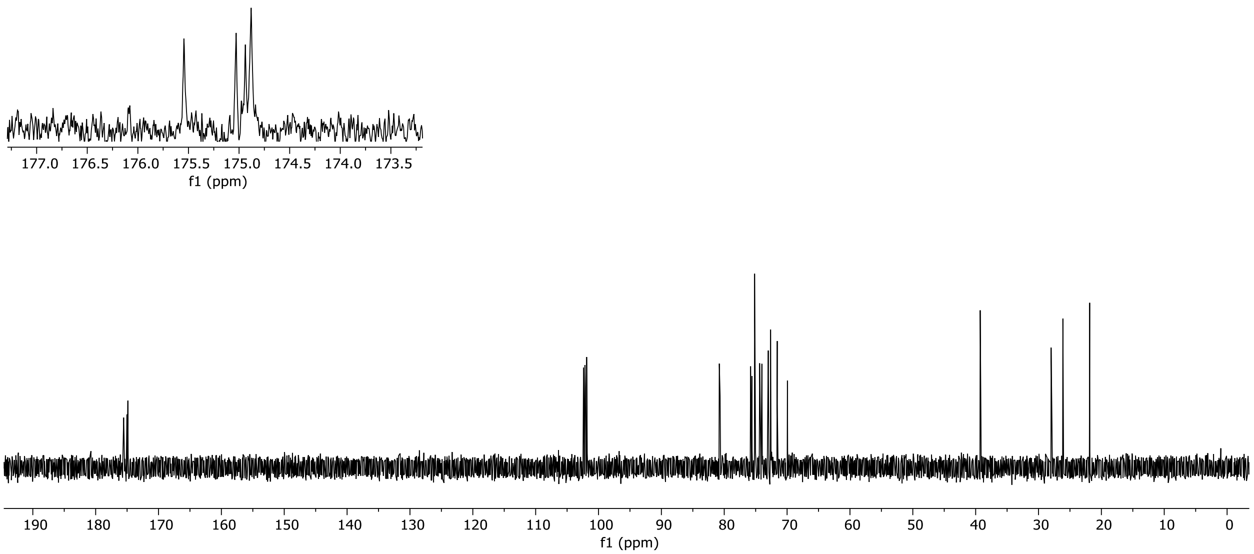

DEPT-135 NMR of 19 (151 MHz, D_2O)

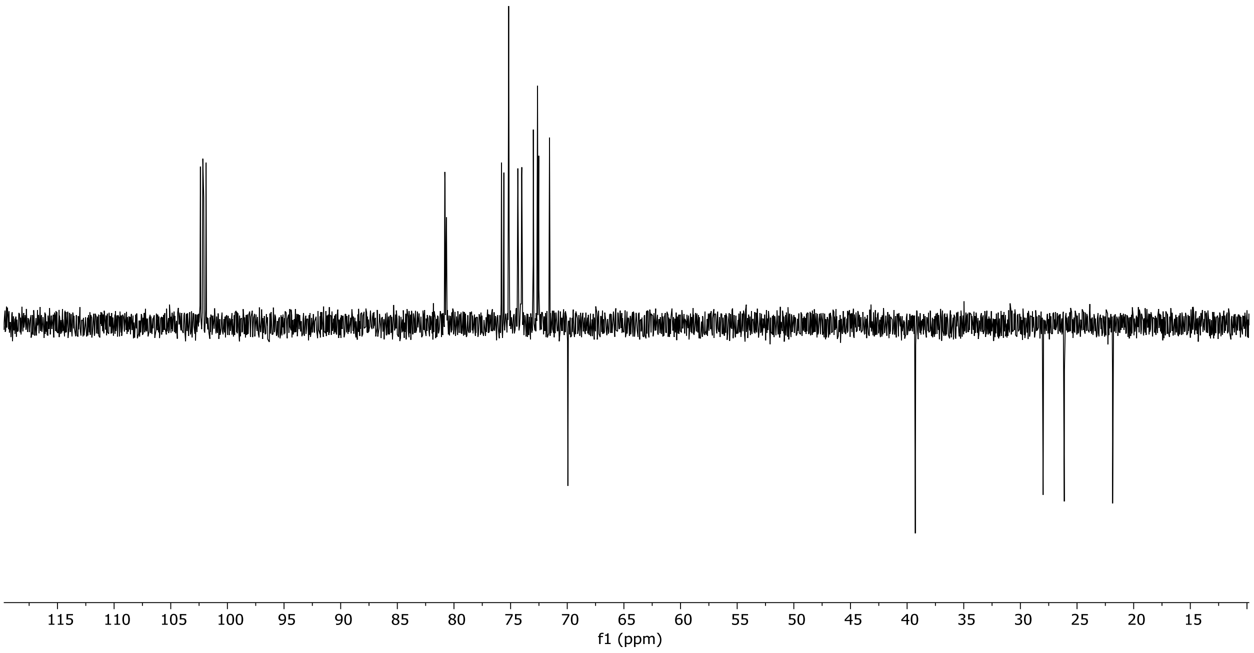

^{1}H-^{13}C HSQC NMR of 19 (600 MHz, D_2O)

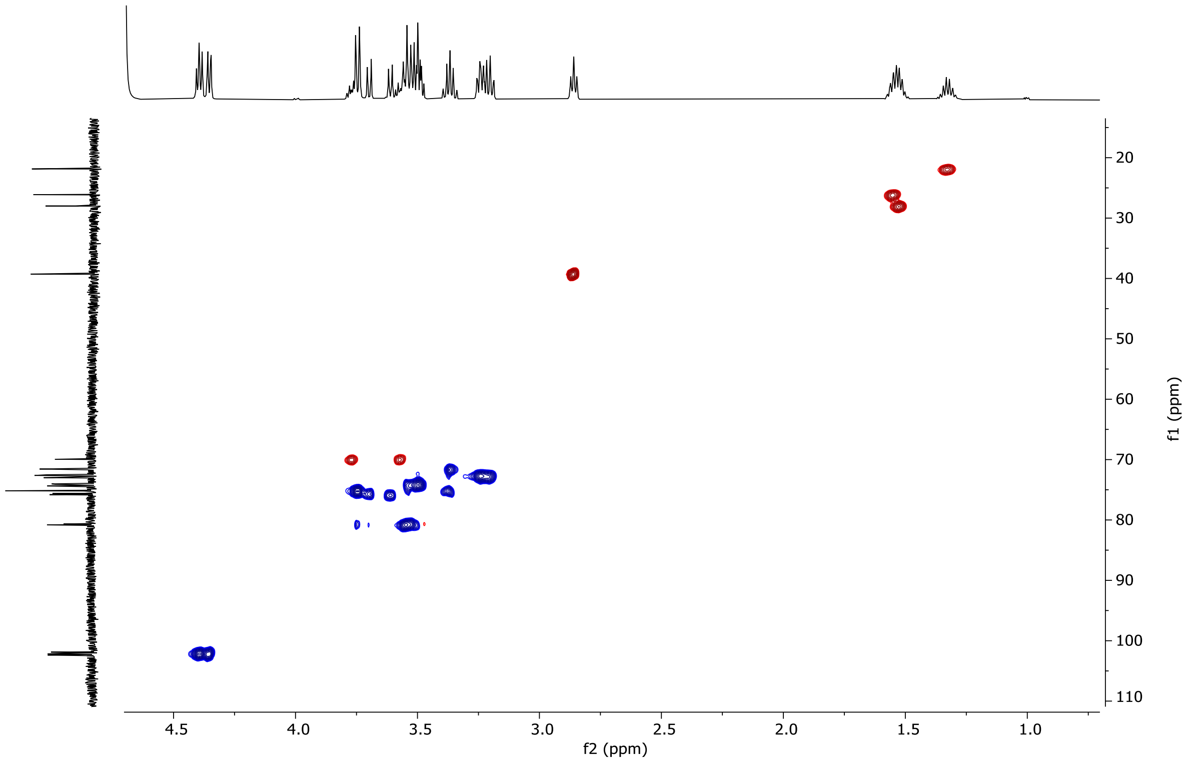

^{1}H-^{13}C coupled HSQC NMR of 19 (400 MHz, D_2O)

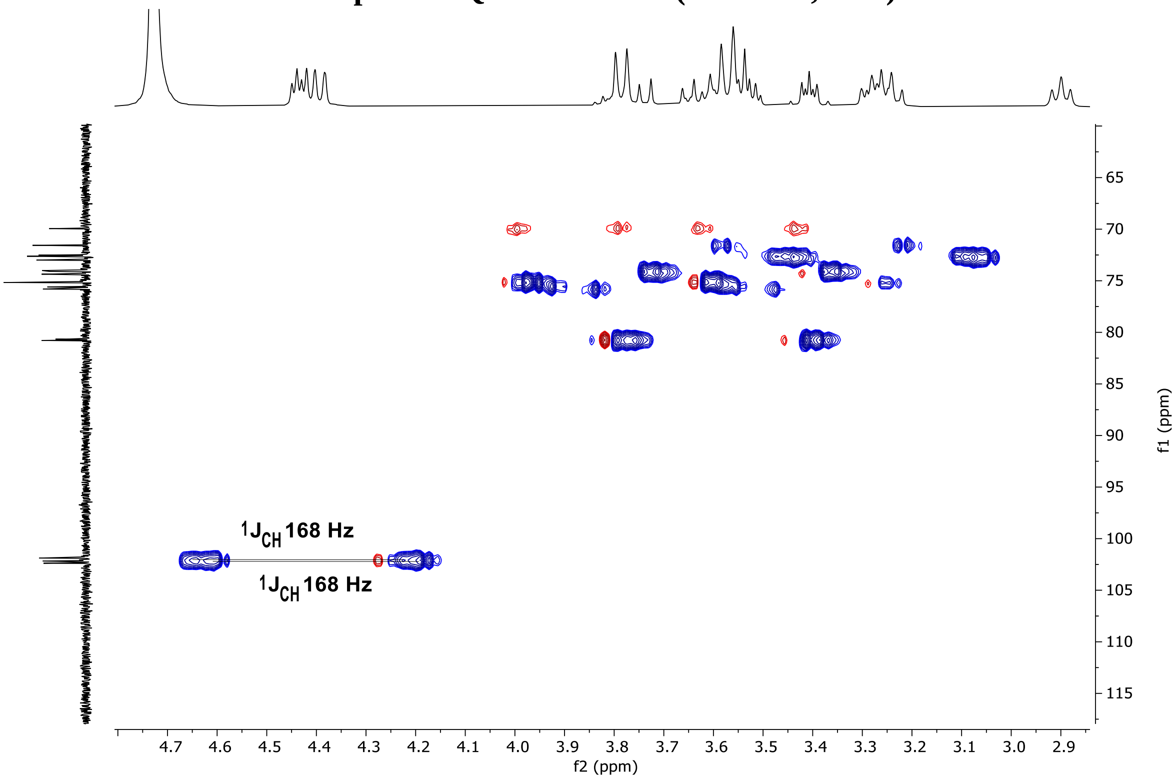

4.6 Synthesis and analytical data of 21

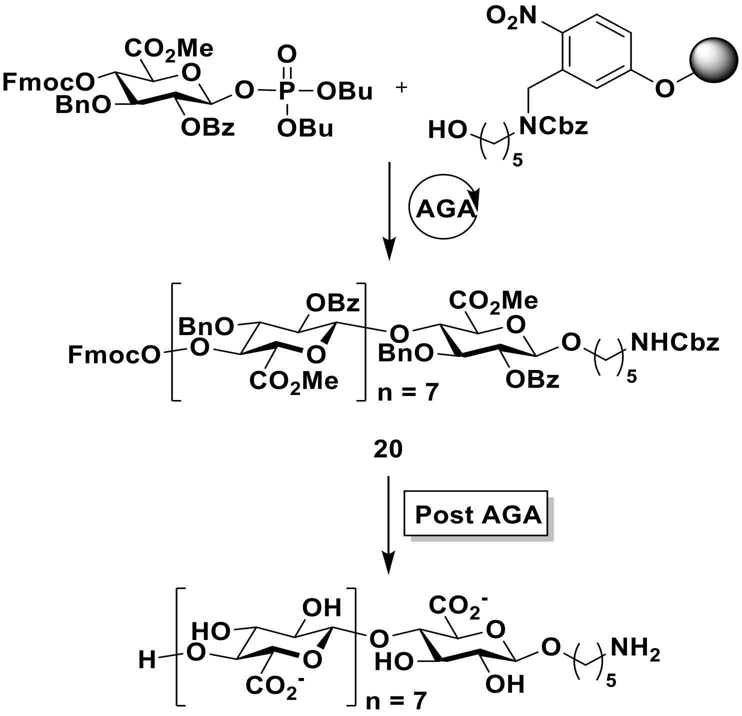

20

21

Step		Modules	Notes
AGA		**A**	
	**BB-7**	**7x (B, 2x D, E, G)**	**D:** (-15 °C for 30 min, 0 °C for 30 min)
	**BB-7**	**B, 2xD**	**D:** (-15 °C for 30 min, 0 °C for 30 min)
Post-AGA	Photocleavage	**H**	
	Purification	$\mathbf{L_{1P}}$	
	Hydrolysis	**J**	24 h (2 mL, 1:1 H_2O/THF)
	Hydrogenolysis	**K**	36 h (2.5 mL, 4:1 H_2O/^{t}BuOH)
	Purification	$\mathbf{L_{1D}}$	

Analytical data for 20

After AGA, photocleavage and NP-purification compound **20** was obtained as a colorless oil (12 mg, 22%). ^{1}**H NMR** (700 MHz, D_2O) δ 7.95 (d, *J* = 7.8 Hz, 2H), 7.85 (m, 16H), 7.74 (t, *J* = 8.4

Hz, 2H), 7.59 – 7.47 (m, 13H), 7.42 – 7.28 (m, 33H), 7.14 (dd, *J* = 7.0, 2.1 Hz, 3H), 7.11 – 6.95 (m, 46H), 5.29 (t, *J* = 8.7 Hz, 1H), 5.17 (t, *J* = 8.5 Hz, 1H), 5.12 – 5.00 (m, 11H), 4.92 (d, *J* = 7.8 Hz, 1H), 4.80 – 4.60 (m, 14H), 4.57 – 4.53 (m, 2H), 4.58-4.52 (d, 2H), 4.50 – 4.38 (m, 9H), 4.34 – 4.29 (m, 1H), 4.23 (m, 2H), 4.15 – 4.02 (m, 6H), 3.90 (t, *J* = 9.2 Hz, 1H), 3.81 (t, *J* = 8.7 Hz, 1H),3.83-3.73 (t, 1H), 3.75 – 3.65 (m,16H), 3.43 (d, *J* = 25.8 Hz, 6H), 3.25 (s, 1H), 3.13 (s, 3H), 3.10 – 3.00 (m, 15H), 2.87 (d, *J* = 6.8 Hz, 2H), 1.40 – 1.32 (m, 4H), 1.09 (q, *J* = 8.0 Hz, 2H). **^{13}C NMR** (151 MHz, $CDCl_3$) δ 168.45, 168.03, 167.97, 167.92, 167.12, 165.11, 165.08, 165.04, 164.86, 156.25, 153.98, 143.33, 143.01, 141.31, 141.27, 138.36, 138.20, 138.14, 137.17, 136.71, 133.32, 133.32, 133.11, 129.84, 129.73, 129.70, 129.55, 128.49, 128.41, 128.33, 128.28, 128.25, 128.22, 128.08, 128.04, 127.98, 127.93, 127.83, 127.81, 127.74, 127.72, 127.60, 127.21, 127.13, 125.19, 125.08, 120.08, 101.28, 100.80, 100.79, 100.77, 100.74, 100.64, 80.18, 79.84, 79.41, 78.95, 78.50, 78.06, 78.00, 75.32, 74.95, 74.87, 74.81, 74.42, 74.35, 74.26, 73.90, 73.87, 73.81, 73.03, 73.00, 72.95, 72.54, 70.40, 69.71, 66.47, 52.61, 52.44, 52.33, 52.29, 52.27, 52.24, 46.60, 40.76, 29.71, 29.32, 28.72, 22.95. **MALDI-TOF**: calcd. $C_{196}H_{189}NNaO_{61}$ for $[m/z+Na+H]^{2+}$ 3557.1683 found 3558.2570.

^{1}H NMR of 20 (700 MHz, D_2O)

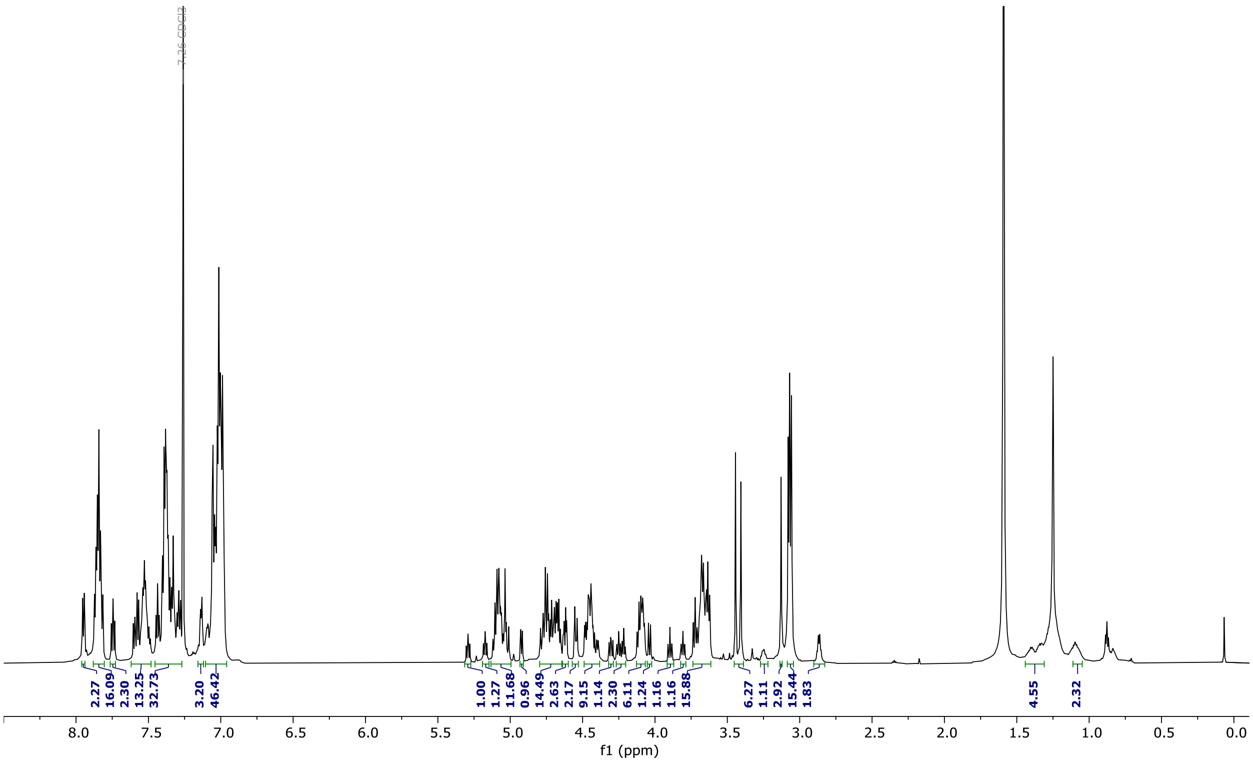

^{13}C NMR of 20 (151 MHz, $CDCl_3$)

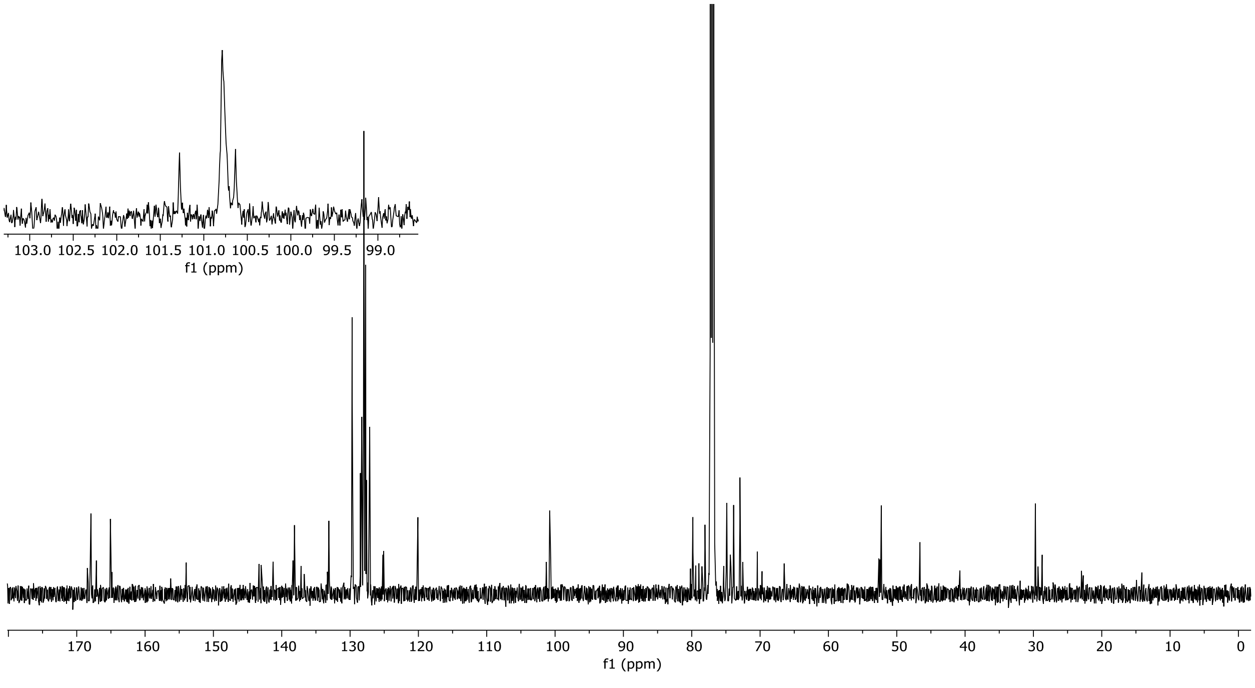

DEPT-135 NMR of 20 (151 MHz, $CDCl_3$)

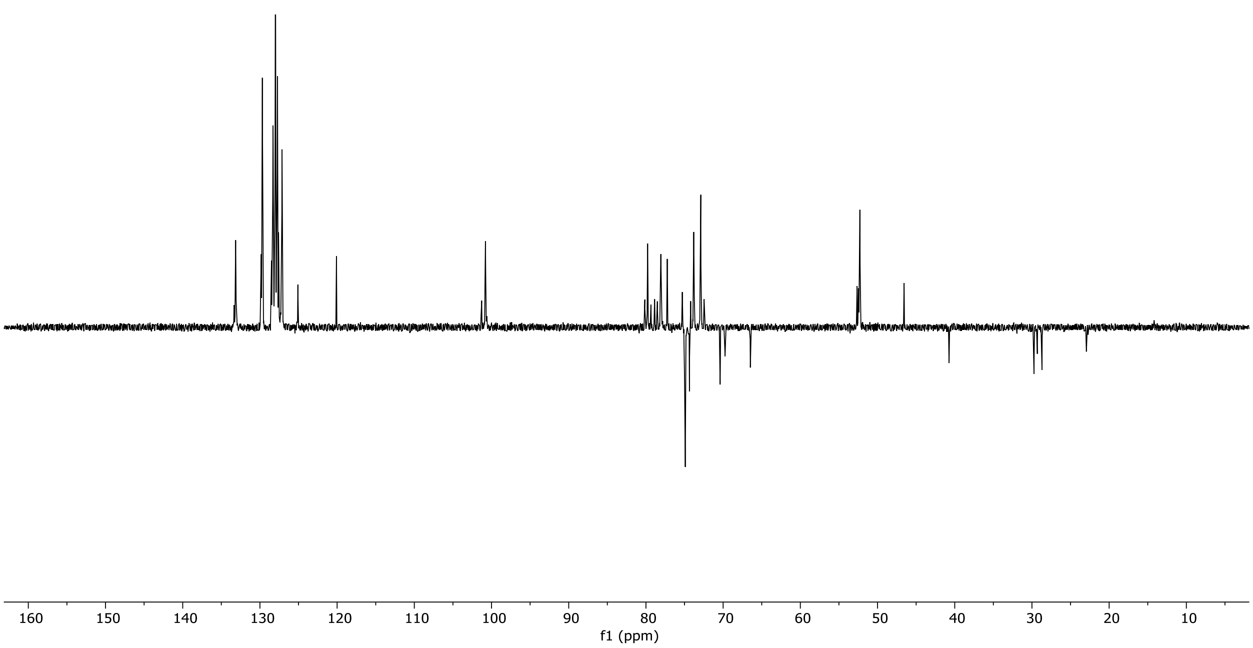

^1H-^{13}C HSQC NMR of 20 (700 MHz, $CDCl_3$)

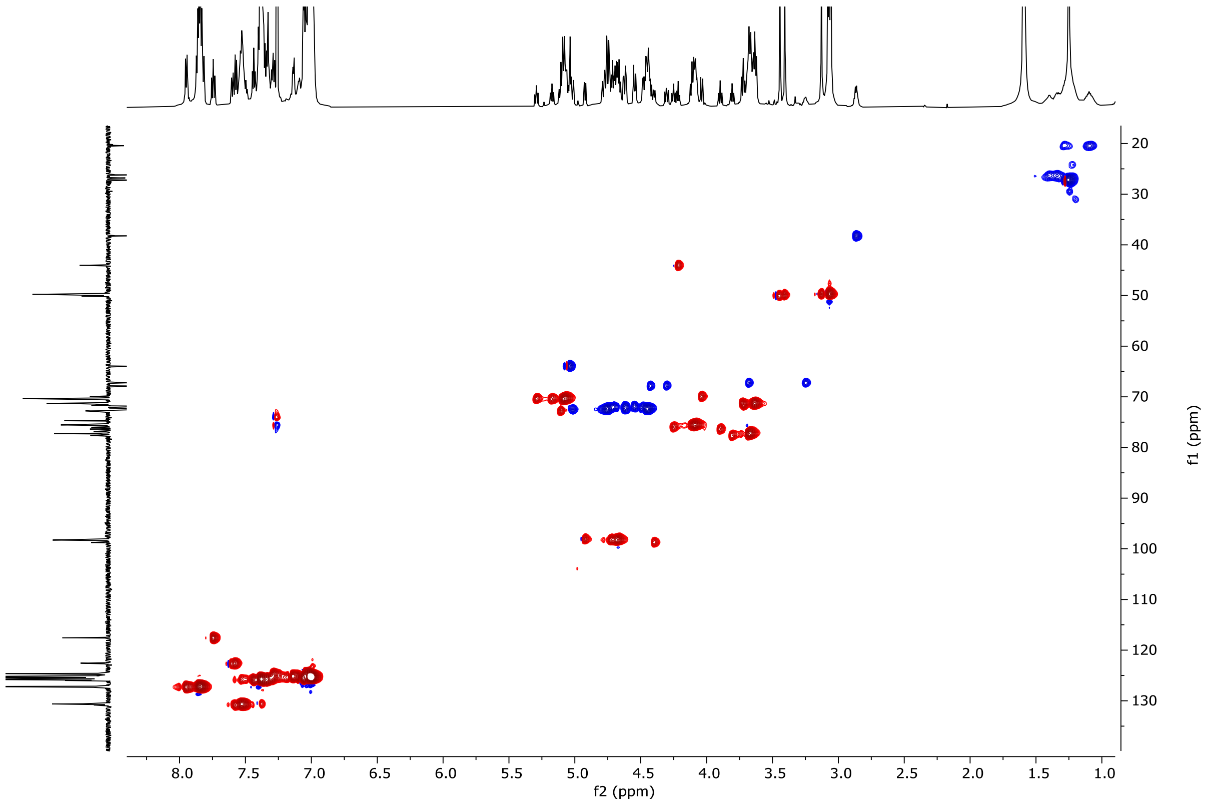

^{1}H-^{13}C coupled HSQC NMR of 20 (700 MHz, $CDCl_3$)

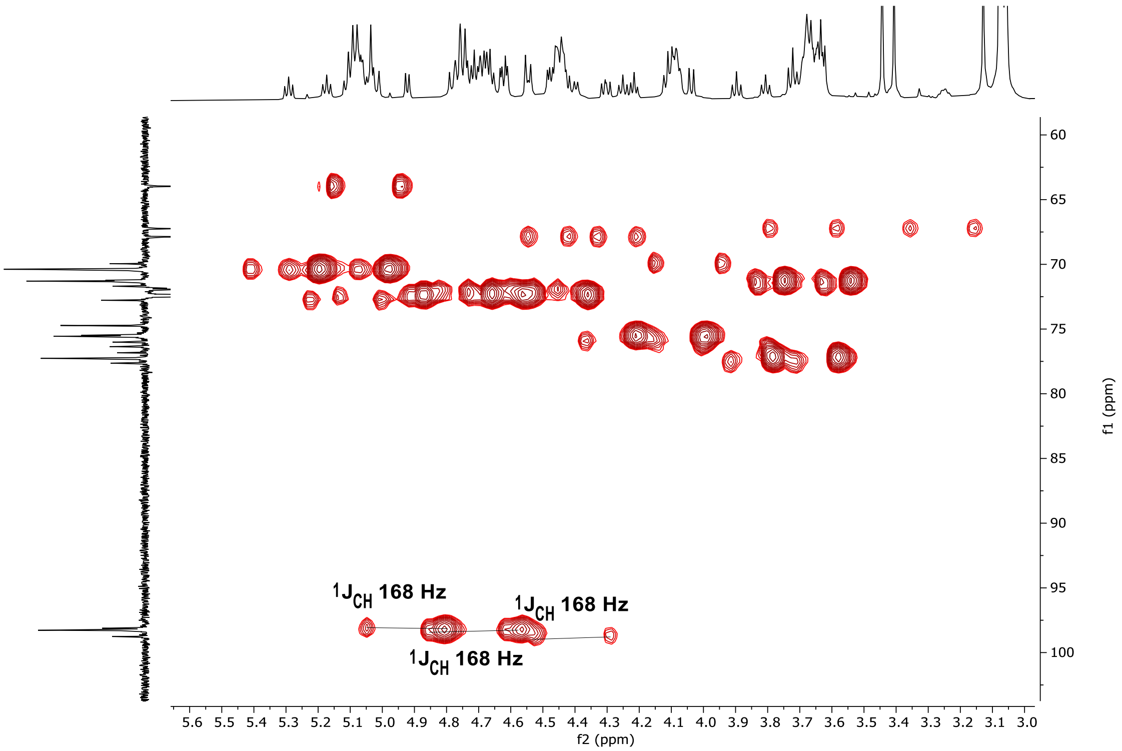

^{1}H-^{1}H COSY NMR of 20 (700MHz, $CDCl_3$)

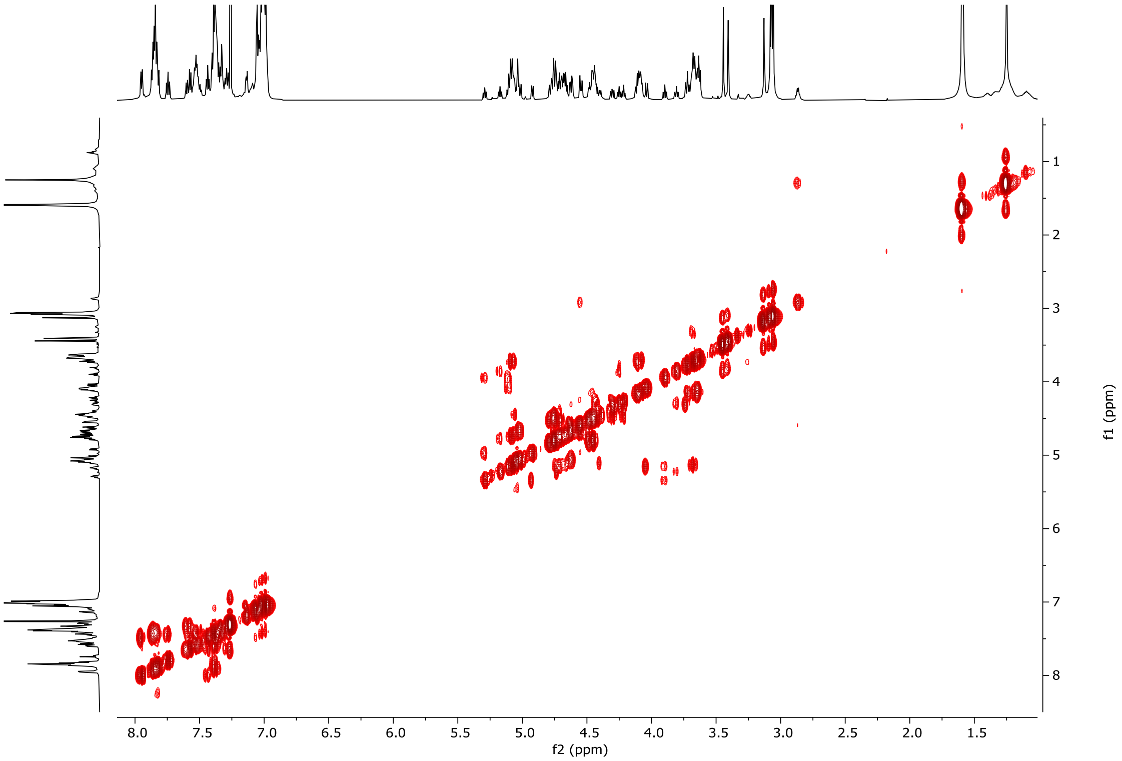

MALDI-TOF for 20

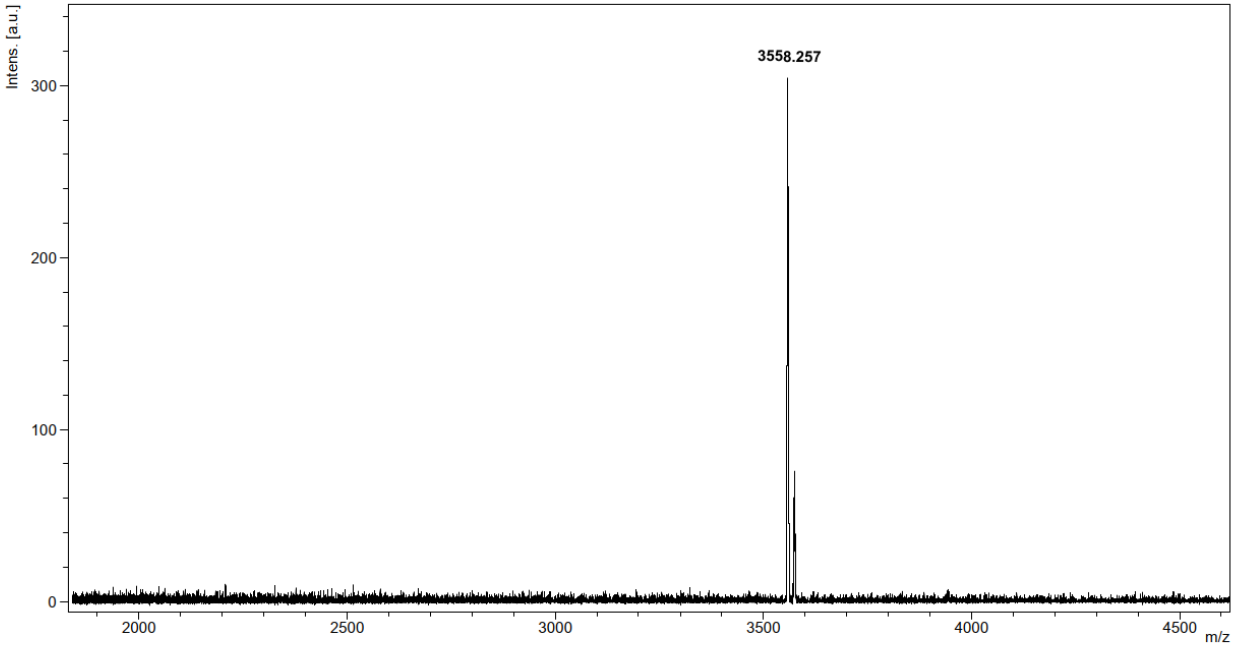

Analytical data for 21

After post-AGA and purification compound **21** was obtained as a white solid (1.8 mg, 55%). **^{1}H NMR** (400 MHz, D_2O): δ 4.47 – 4.32 (m, 8H), 3.83 – 3.69 (m, 10H), 3.67 – 3.46 (m, 19H), 3.43 – 3.37 (m, 2H), 3.25 (dt, *J* = 16.9, 8.2 Hz, 8H), 2.89 (t, *J* = 7.4 Hz, 2H), 1.62 – 1.50 (m, 4H), 1.41 – 1.29 (m, 2H). **^{13}C NMR** (176 MHz, D_2O): δ 175.07, 175.06, 175.05, 174.99, 102.41, 102.33, 102.23, 102.20, 102.12, 102.10, 101.90, 80.84, 80.81, 80.71, 80.68, 75.83, 75.63, 75.20, 74.39, 74.04, 73.02, 72.65, 72.63, 72.54, 71.58, 69.96, 39.31, 28.01, 26.14, 21.87. In ^{13}C NMR carbonyl peaks extracted from HMBC NMR **HRMS QTOF-MS**: calcd. $C_{53}H_{76}NO_{49}^{-}$ for $[M-H]^{-}$ 1510.3491, found 1510.3420.

^{1}H NMR of 21 (400 MHz, D_2O)

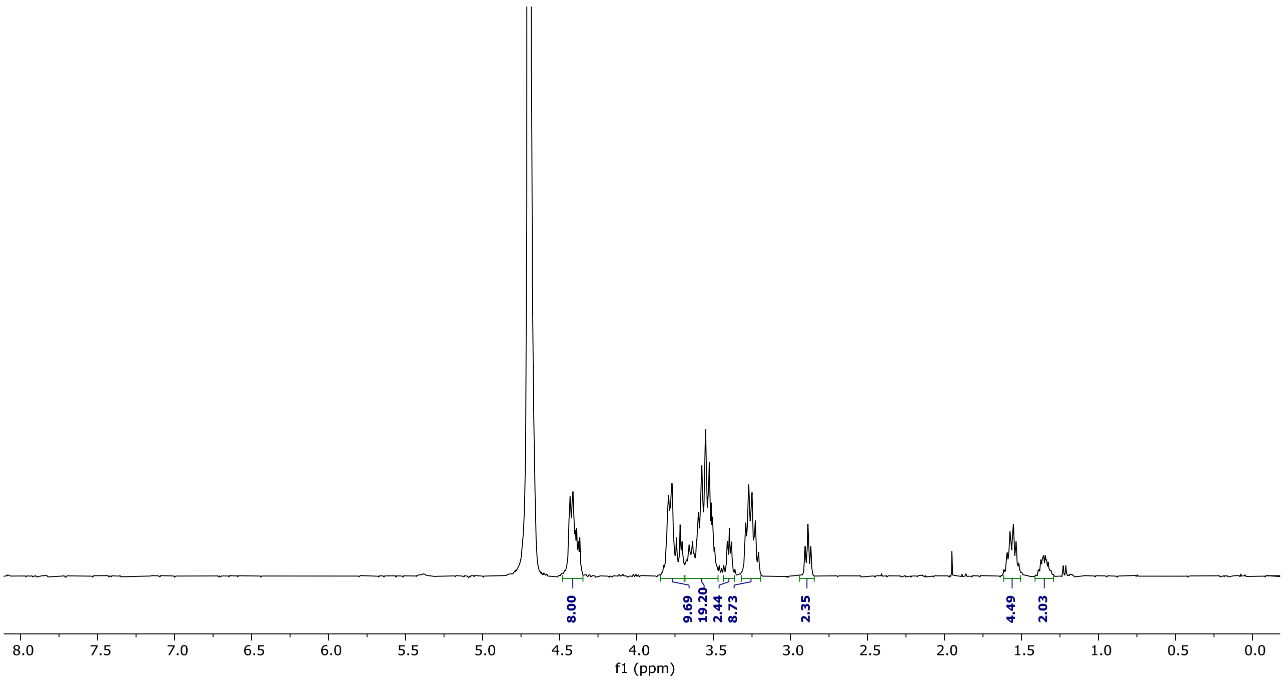

^{13}C NMR of 21 (176 MHz, D_2O)

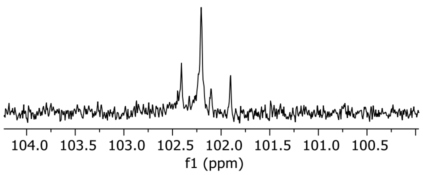

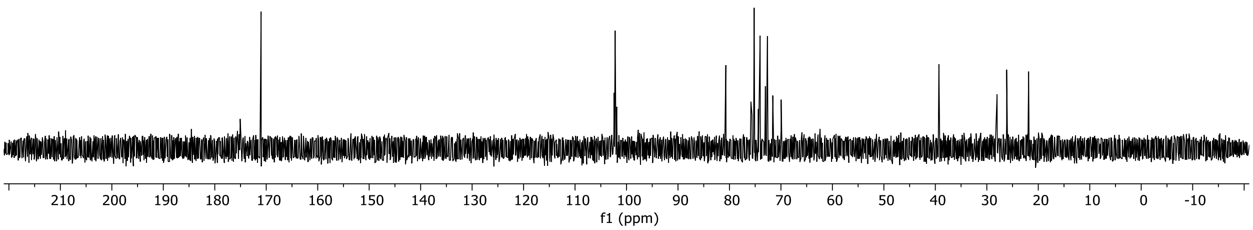

DEPT-135 NMR of 21 (176 MHz, D_2O)

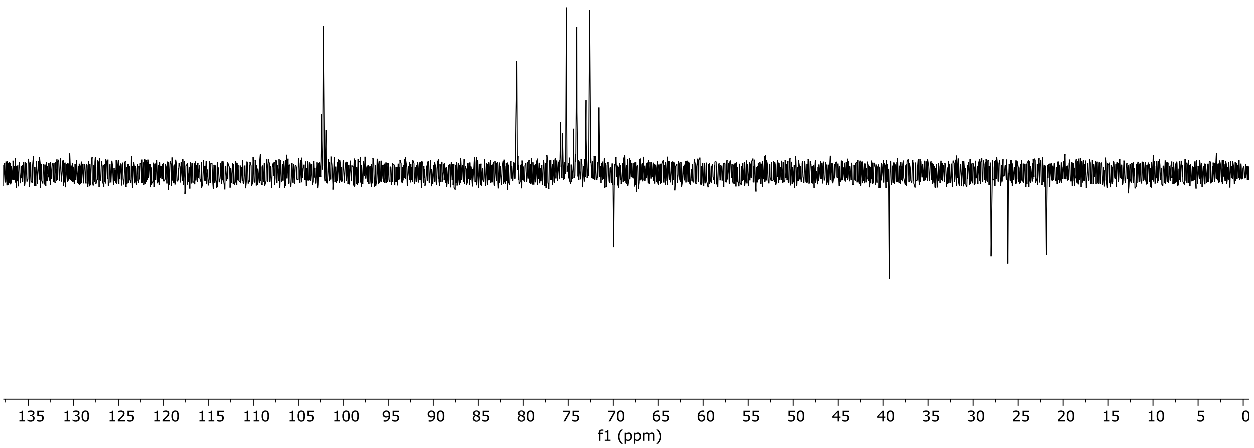

^{1}H-^{13}C HSQC NMR of 21 (700 MHz, D_2O)

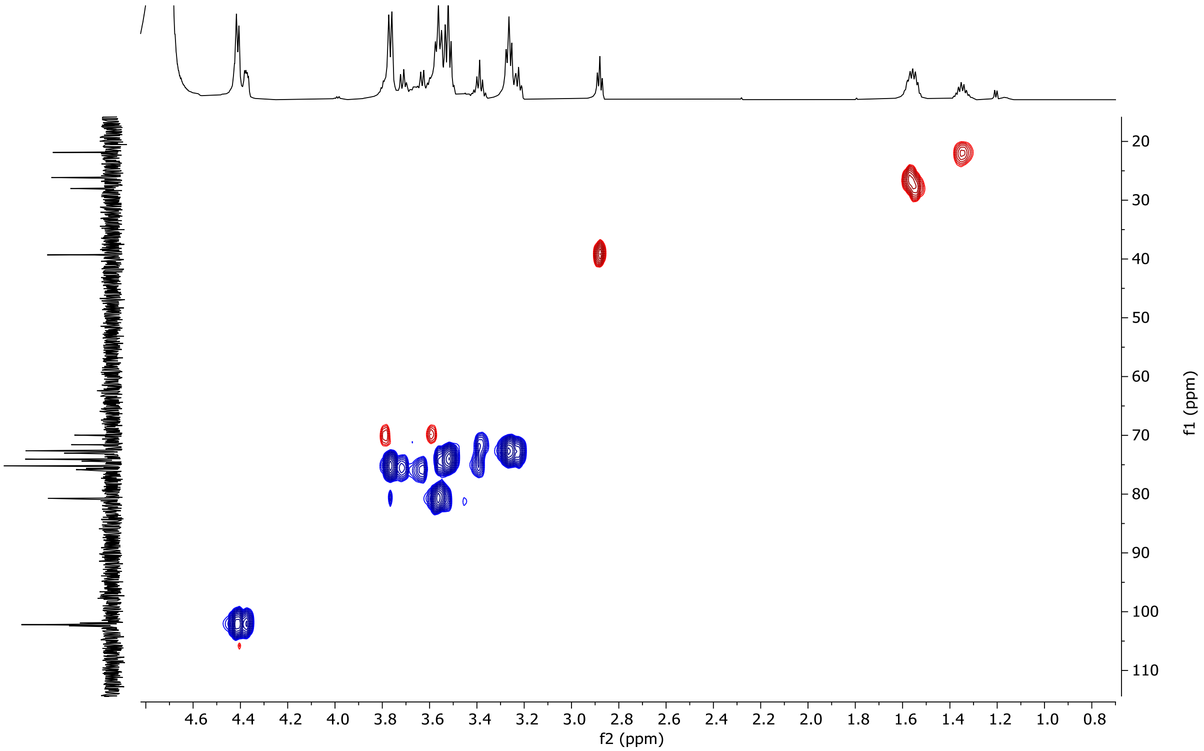

^{1}H-^{1}H COSY NMR of 21 (700 MHz, D_2O)

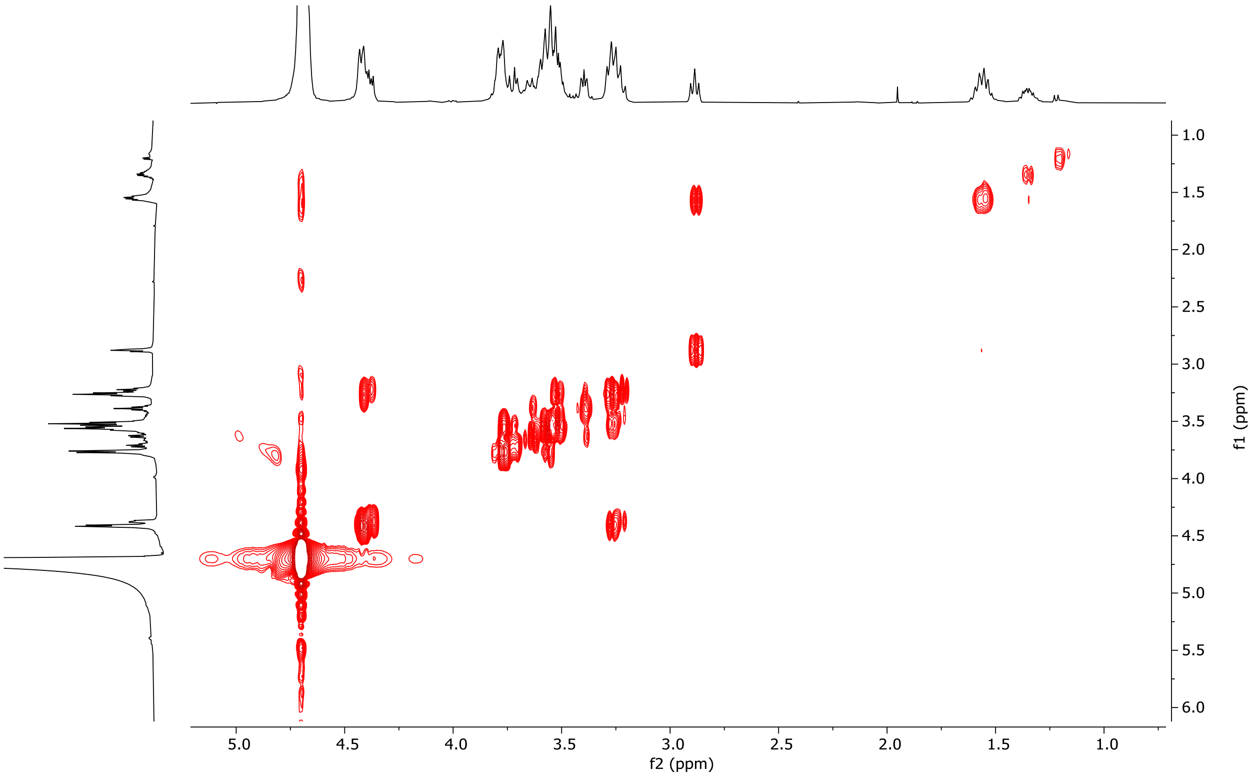

4.7 Synthesis and analytical data of 23

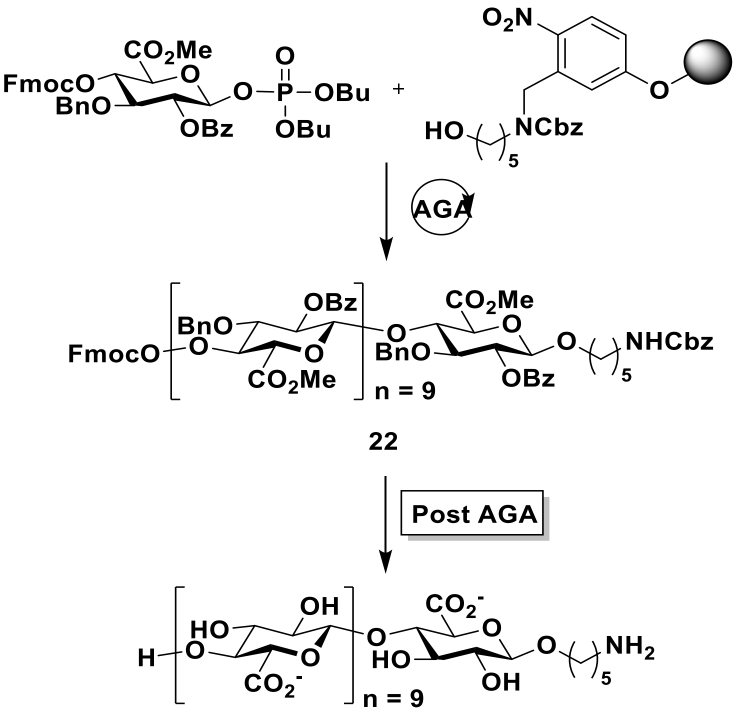

Step		Modules	Notes
AGA		**A**	
	**BB-7**	**9x (B, 2x D, E, G)**	**D:** (-15 °C for 30 min, 0 °C for 30 min)
	**BB-7**	**B, 2xD**	**D:** (-15 °C for 30 min, 0 °C for 30 min)
Post-AGA	Photocleavage	**H**	
	Purification	**L_{1P}**	
	Hydrolysis	**J**	30 h (2mL, 1:1 H_2O/THF)
	Hydrogenolysis	**K**	36 h (2.5 mL, 4:1 H_2O/ ^{t}BuOH)
	Purification	**L_{1D}**	

Analytical data for 22

After AGA, photocleavage and NP-purification compound **22** was obtained as a colorless oil (13 mg, 20%). **^{1}H NMR** (400 MHz, $CDCl_3$): δ 7.98 (dt, *J* = 7.0, 1.4 Hz, 2H), 7.91 – 7.84 (m, 18H), 7.77 (dd, *J* = 7.6, 5.1 Hz, 2H), 7.65 – 7.50 (m, 13H), 7.47 (d, *J* = 7.9 Hz, 2H), 7.45 – 7.30 (m,

30H), 7.17 (dt, J = 5.5, 1.6 Hz, 3H), 7.10 – 6.99 (m, 48H), 5.32 (dd, J = 9.4, 7.9 Hz, 1H), 5.22 – 5.03 (m, 13H), 4.95 (d, J = 8.0 Hz, 1H), 4.83 – 4.61 (m, 19H), 4.57 (m, 2H), 4.53 – 4.41 (m, 10H), 4.36 – 4.21 (m, 3H), 4.17 – 4.05 (m, 9H), 3.92 (t, J = 9.2 Hz, 1H), 3.83 (t, J = 8.7 Hz, 1H), 3.77 – 3.63 (m, 18H), 3.46 (d, J = 15.6 Hz, 6H), 3.31 – 3.25 (m, 1H), 3.16 (s, 3H), 3.13 – 3.06 (m, 19H), 2.90 (q, J = 6.6 Hz, 2H), 1.47 – 1.29 (m, 4H), 1.13 (q, J = 5.8 Hz, 2H). **^{13}C NMR** (151 MHz, $CDCl_3$): δ 168.16, 168.10, 168.05, 167.26, 165.25, 165.21, 165.18, 165.16, 165.00, 156.38, 154.11, 143.46, 143.14, 141.44, 141.40, 138.48, 138.32, 138.26, 138.23, 137.30, 136.84, 133.46, 133.23, 129.97, 129.87, 129.83, 128.62, 128.55, 128.41, 128.35, 128.22, 128.17, 128.11, 127.96, 127.94, 127.89, 127.85, 127.73, 127.35, 127.26, 127.24, 125.32, 125.21, 120.21, 101.41, 100.92, 100.89, 100.76, 79.96, 79.54, 79.08, 78.63, 78.19, 78.12, 75.45, 75.08, 75.00, 74.95, 74.55, 74.49, 74.39, 73.99, 73.94, 73.12, 73.07, 72.66, 70.53, 69.85, 66.61, 52.75, 52.57, 52.46, 52.44, 52.39, 46.73, 40.89, 36.06, 32.07, 29.84, 29.50, 29.46, 29.39, 22.84. MALDI-TOF**:** calcd. $C_{238}H_{229}NNaO_{75}$ for $[M+Na+2H]^{3+}$ 4323.4034 found 4320.8460.

NP-HPLC of crude 22 (ELSD trace, Method K_{1D} t_R = 24.54 min)

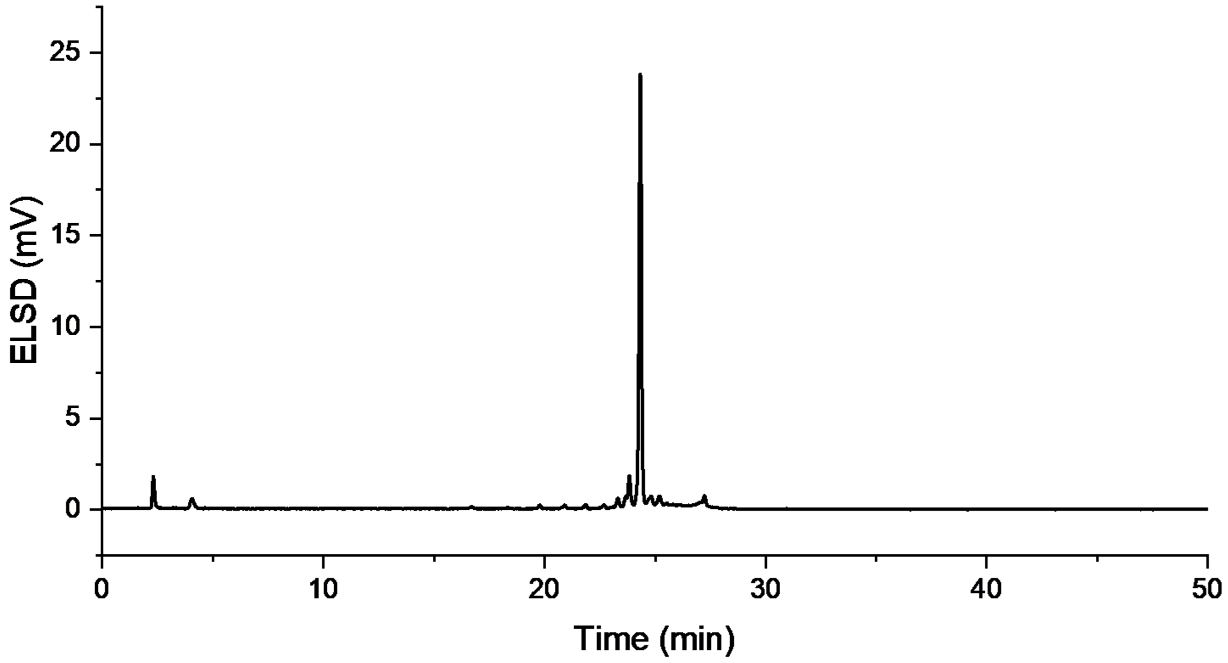

NP-HPLC of pure 22 (ELSD trace, Method K_{1D} t_R = 24.54 min)

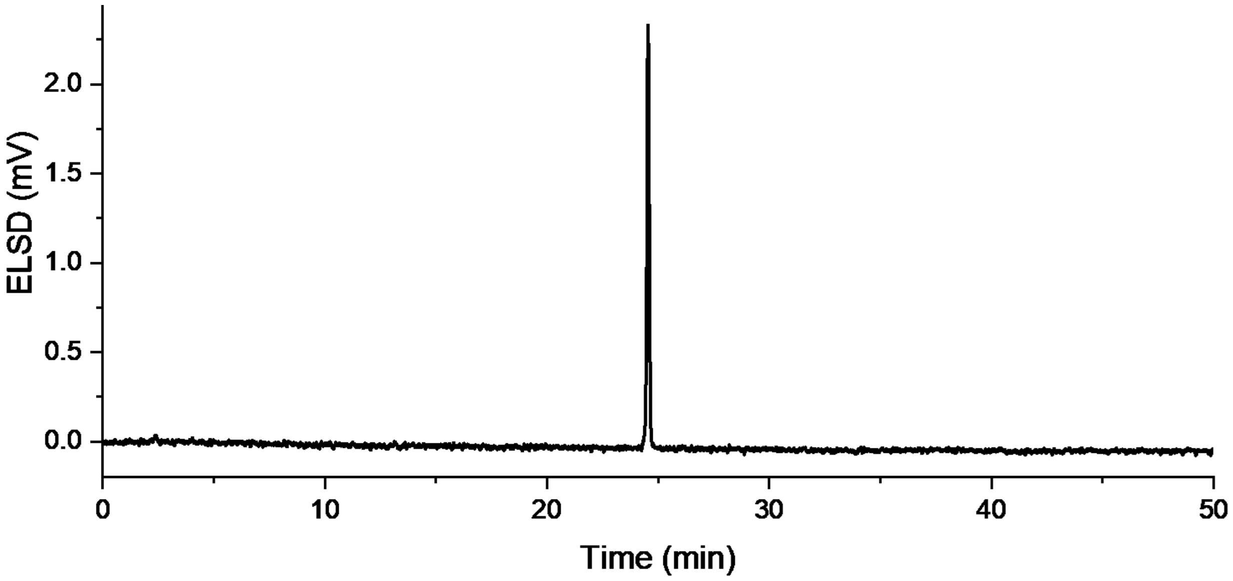

^{1}H NMR of 22 (400 MHz, $CDCl_3$)

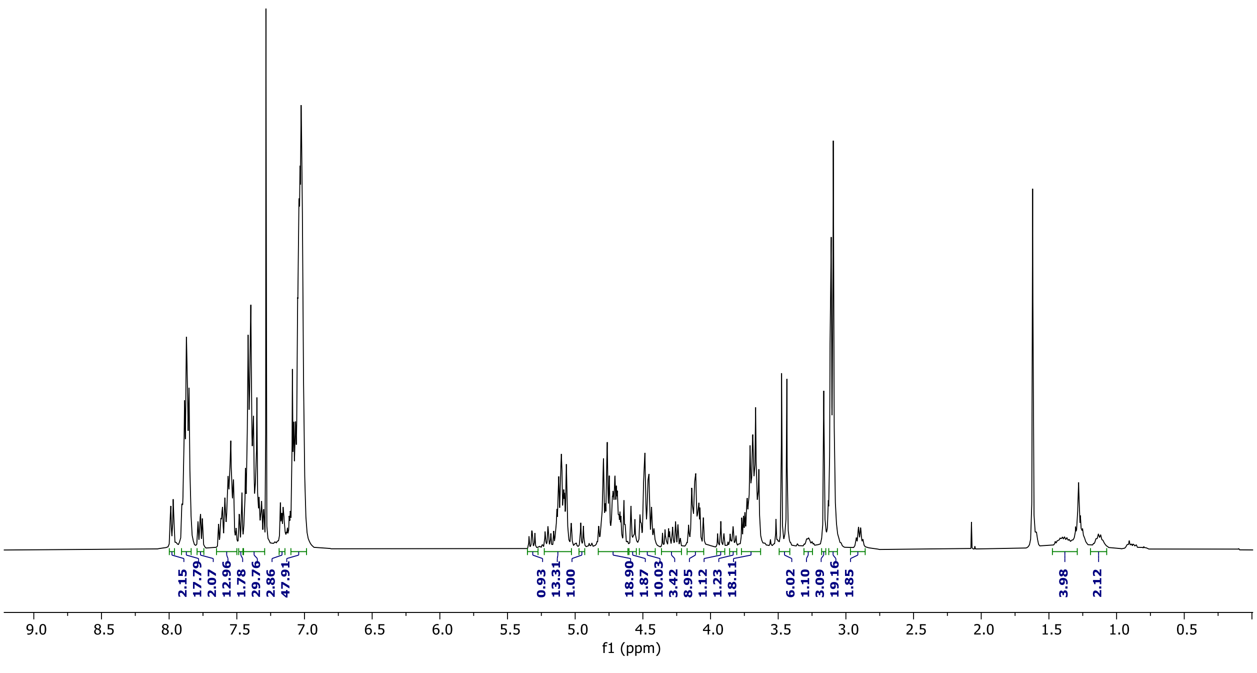

^{13}C NMR of 22 (151 MHz, $CDCl_3$)

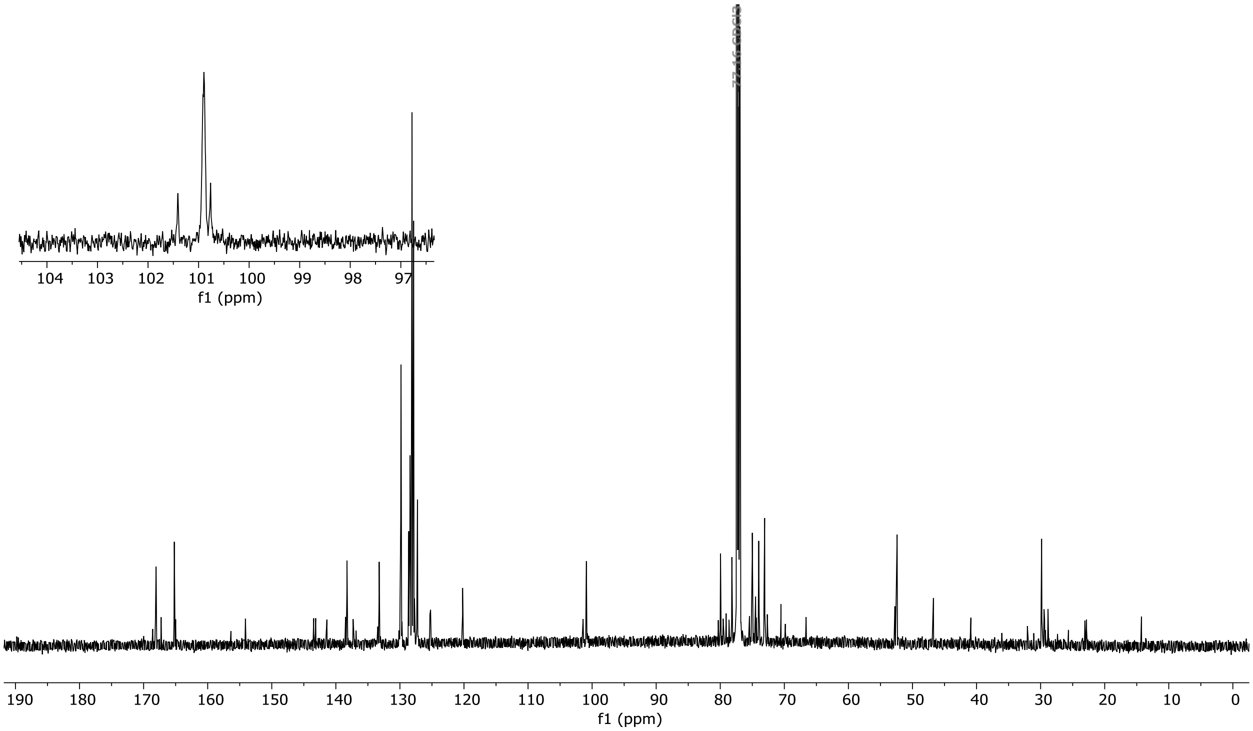

DEPT-135 NMR of 22 (151 MHz, $CDCl_3$)

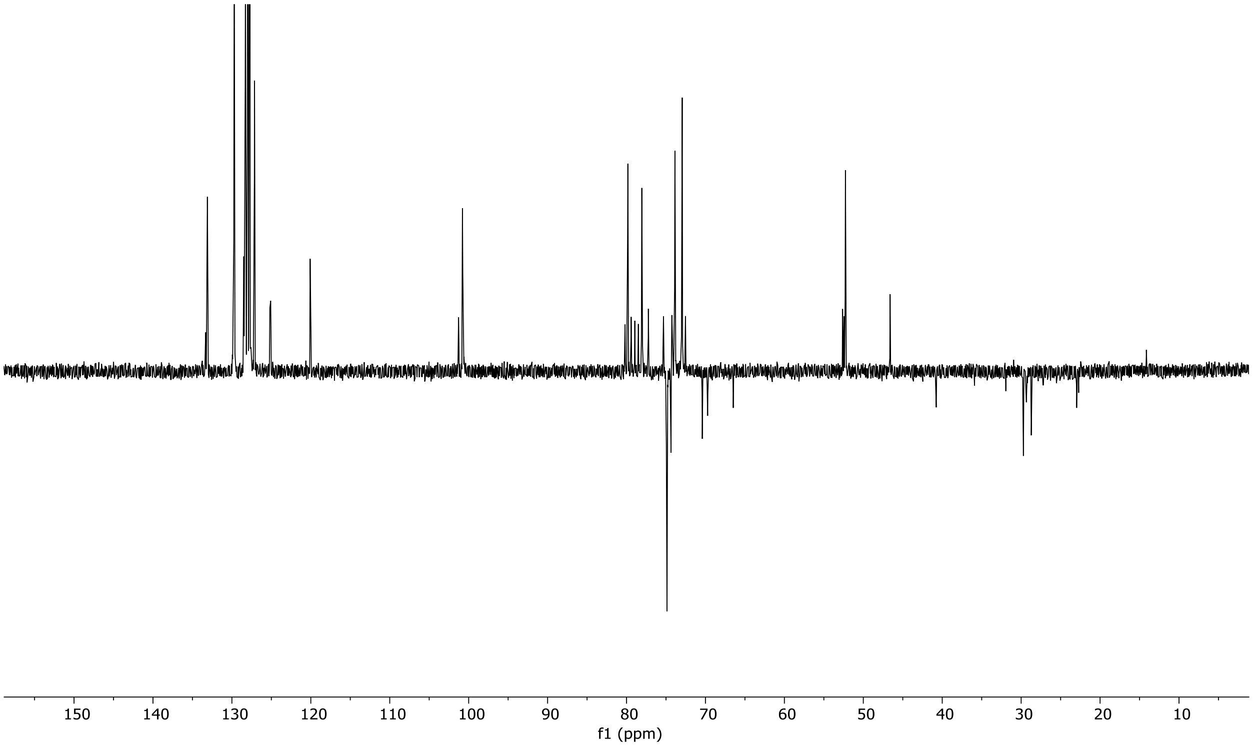

^{1}H-^{13}C HSQC NMR of 22 (600 MHz, $CDCl_3$)

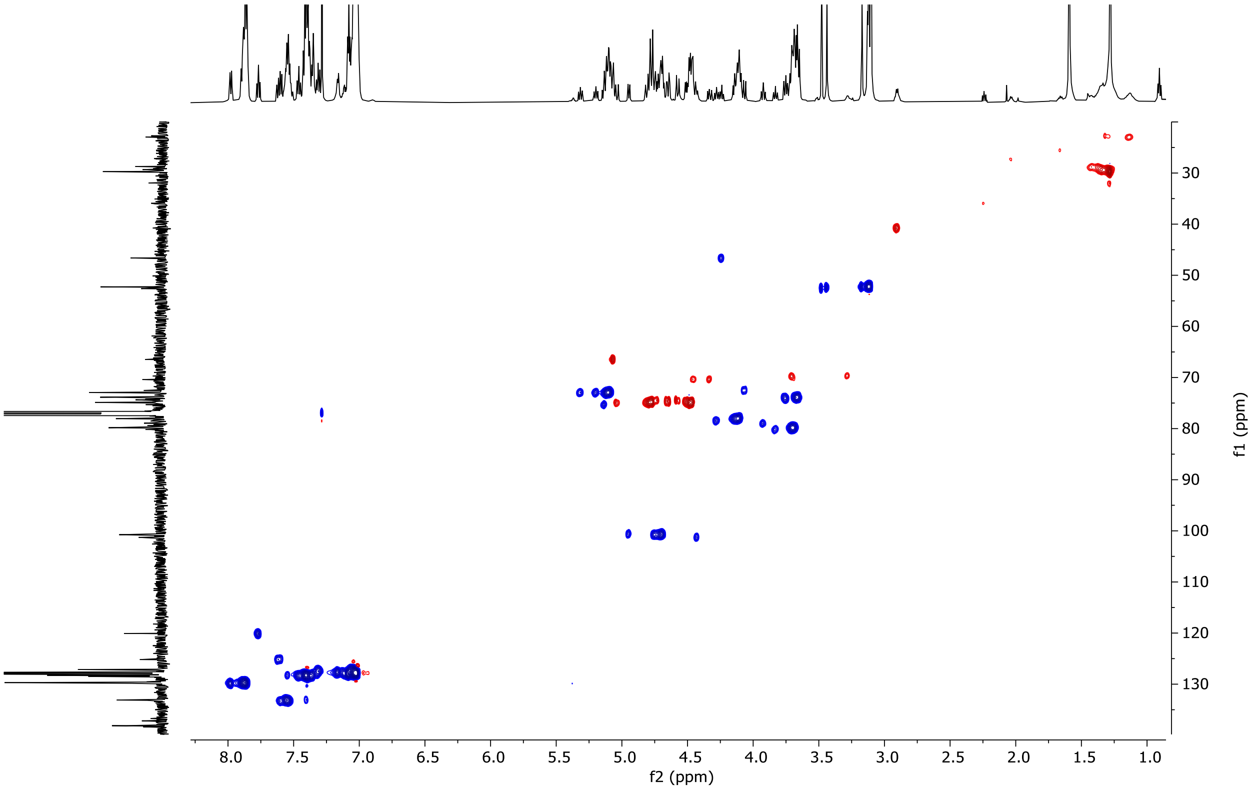

^{1}H-^{13}C coupled HSQC NMR of 22 (400 MHz, $CDCl_3$)

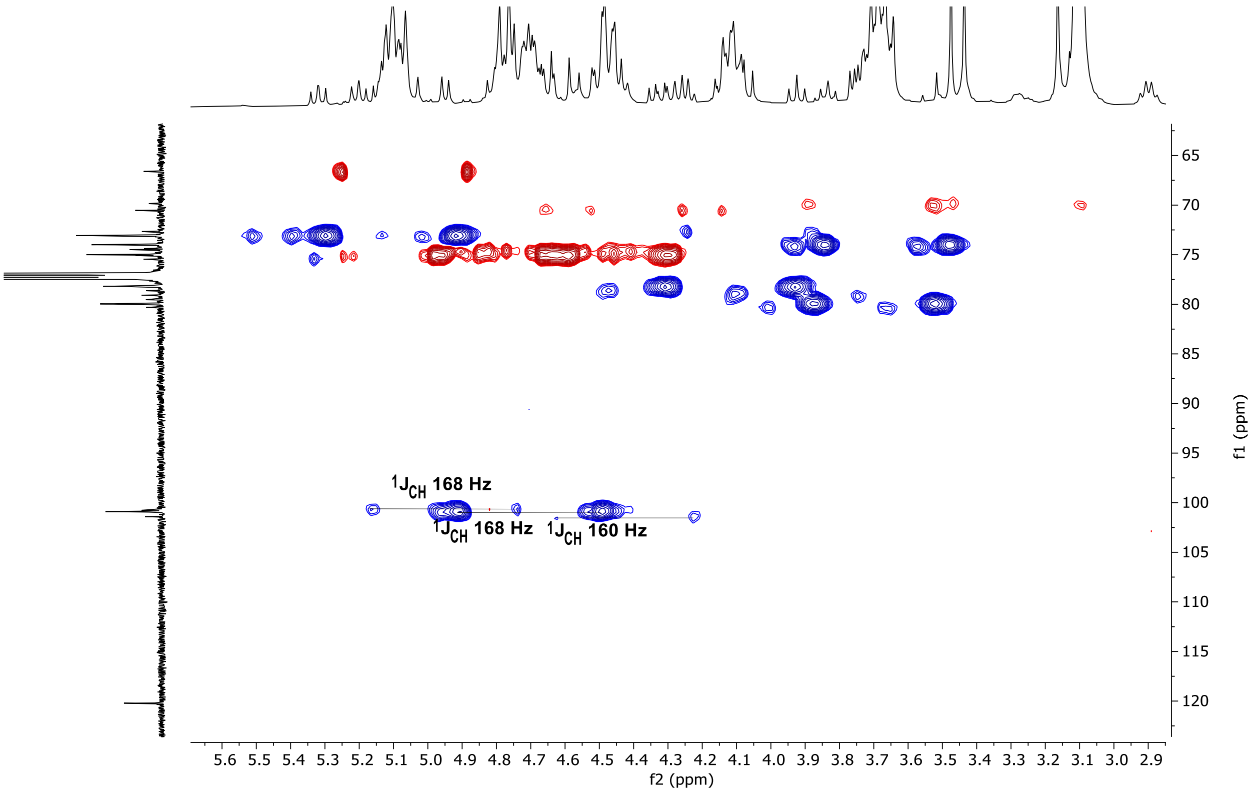

^{1}H-^{1}H COSY NMR of 22 (600 MHz, CDCl$_3$)

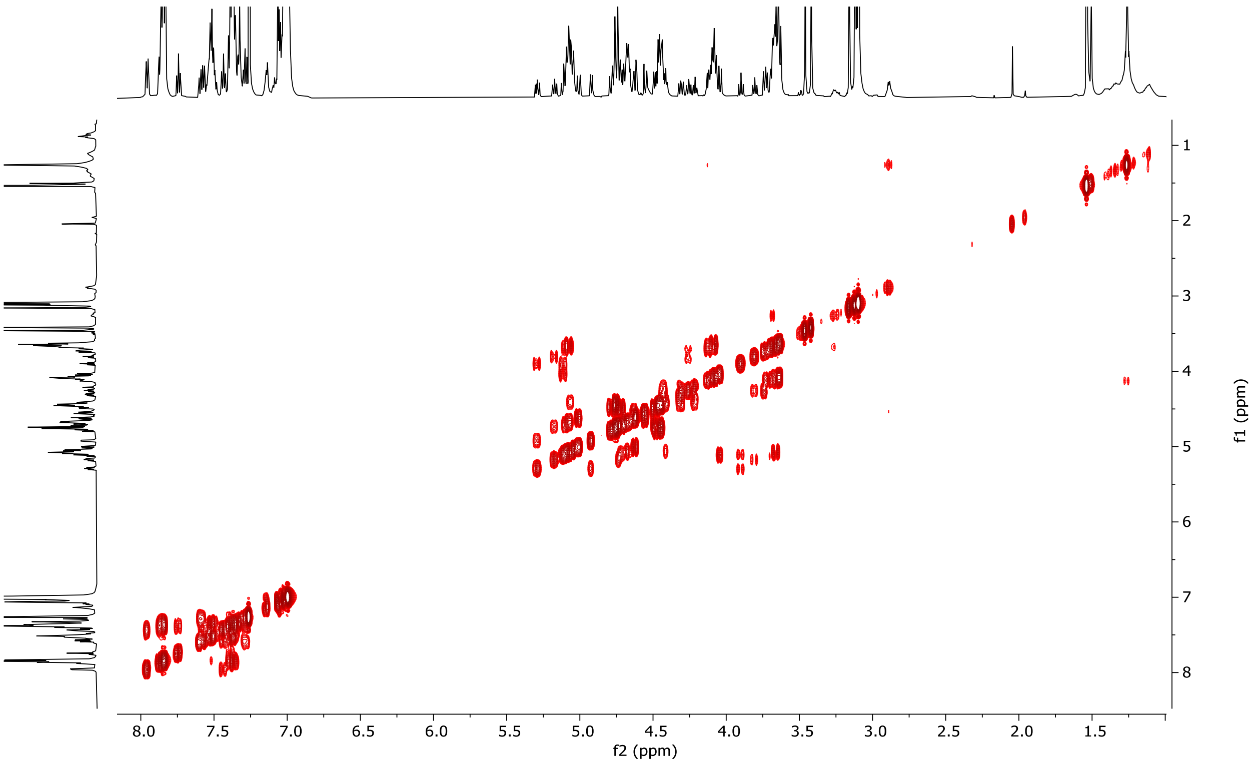

MALDI-TOF for 22

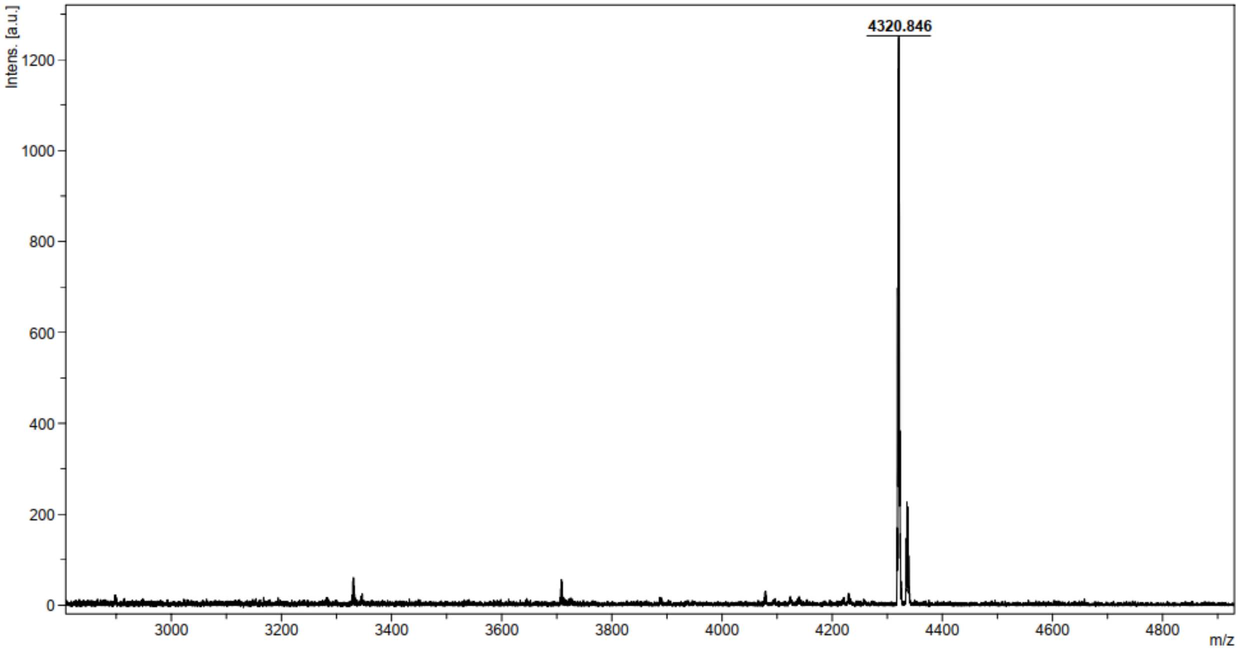

Analytical data for 23

After post-AGA and purification compound **23** was obtained as a white solid (2.4 mg, 50%). **^{1}H NMR** (400 MHz, D_2O): δ 4.44 – 4.36 (m, 10H), 3.77 (d, *J* = 8.9 Hz, 10H), 3.66 – 3.48 (m, 24H), 3.42 – 3.36 (m, 2H), 3.29 – 3.20 (m, 11H), 2.88 (t, *J* = 7.5 Hz, 2H), 1.65 – 1.45 (m, 4H), 1.43 – 1.30 (m, 2H). **^{13}C NMR** (176 MHz, D_2O): δ 174.60, 174.46, 174.23, 174.16, 174,02, 173.81, 173.73, 102.39, 102.18, 102.08, 101.89, 80.92, 80.81, 80.67, 75.80, 75.60, 75.20, 75.17, 74.38, 74.10, 74.01, 73.00, 72.60, 72.52, 71.56, 69.93, 39.28, 27.99, 26.11, 21.85. In ^{13}C NMR carbonyl peaks extracted from HMBC NMR **HRMS QTOF-MS**: calcd. $C_{17}H_{28}NO_{13}^-$ for $[m/z\text{-}2H]^{2-}$ 930.7030, found 930.7009.

^{1}H NMR of 23 (400 MHz, D_2O)

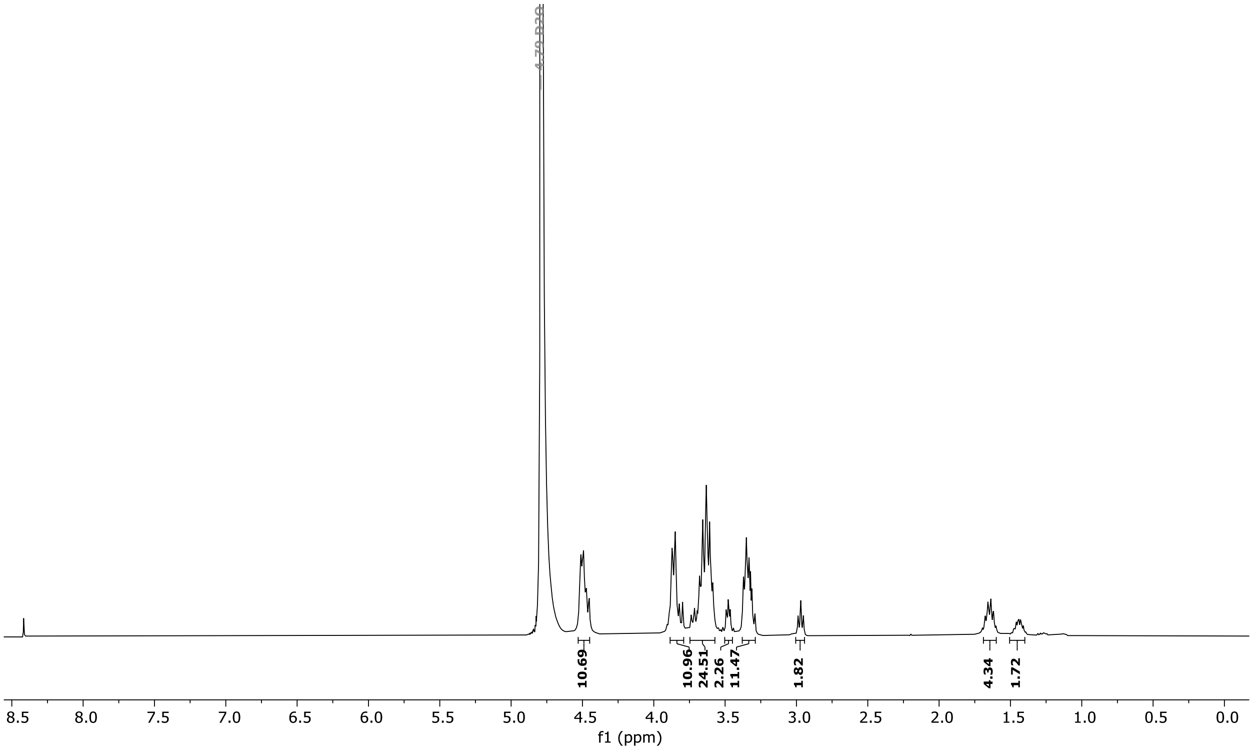

^{13}C NMR of 23 (176 MHz, D_2O)

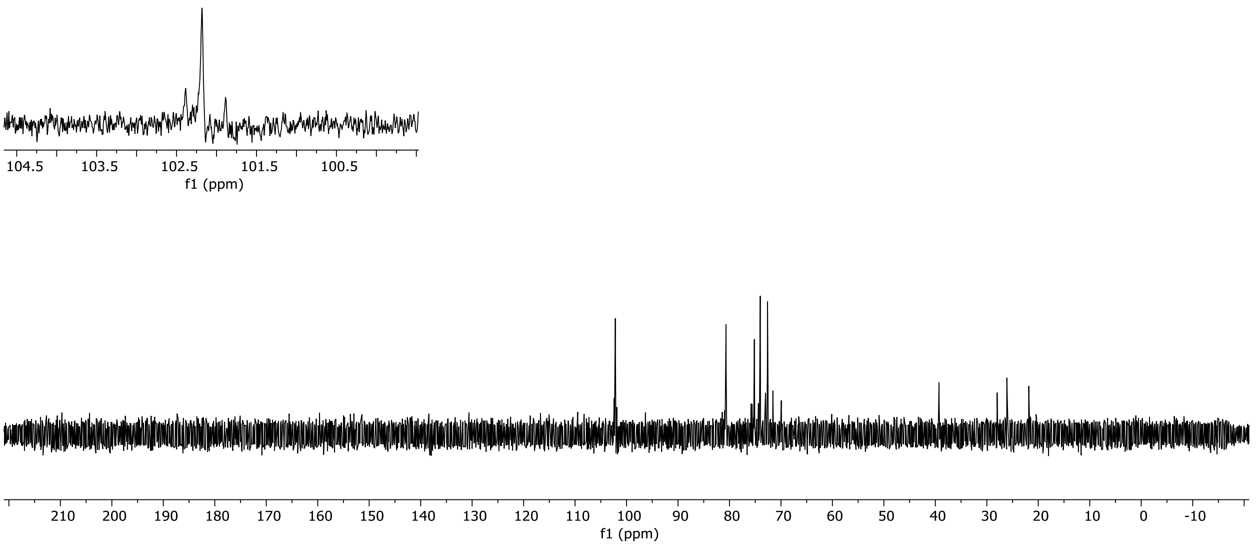

DEPT-135 NMR of 23 (176 MHz, D_2O)

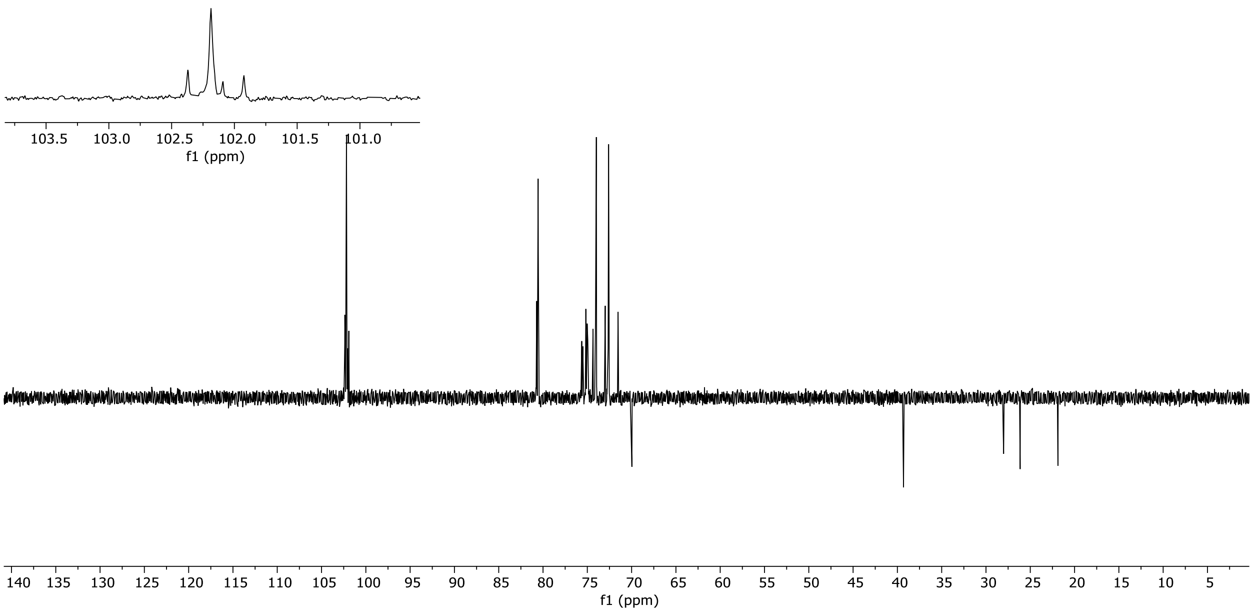

^{1}H-^{13}C HSQC NMR of 23 (600 MHz, D_2O)

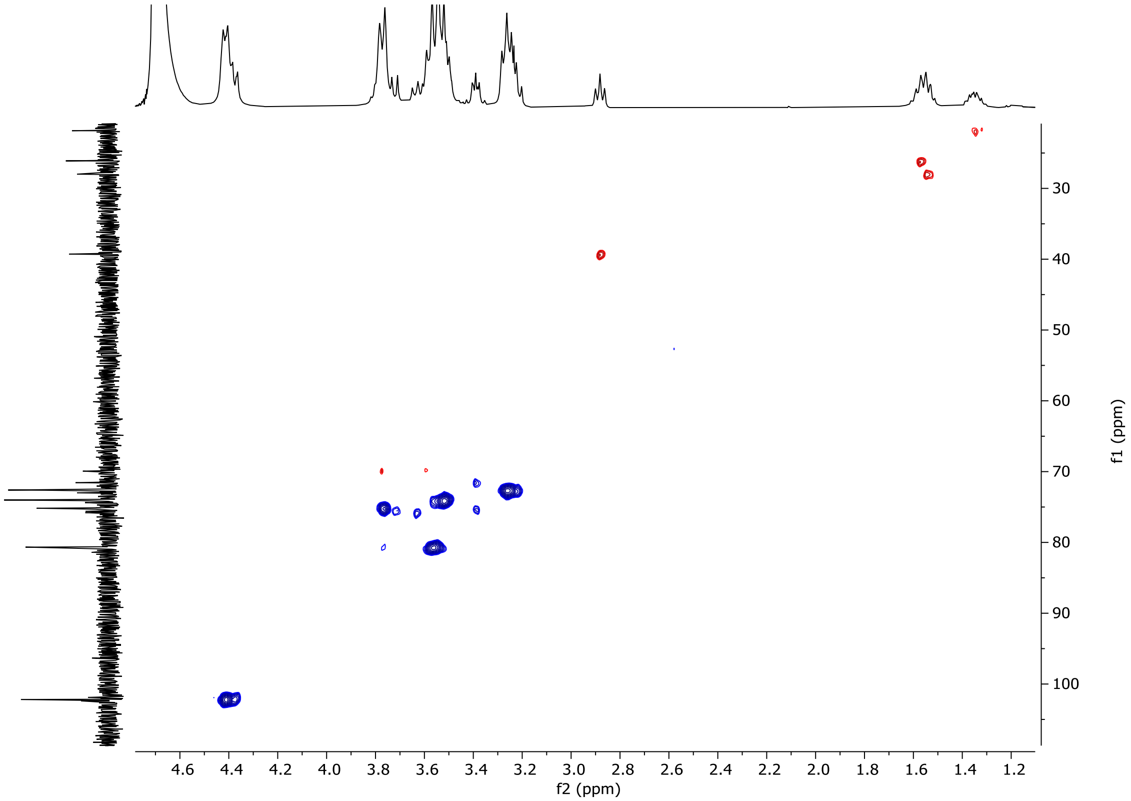

^{1}H-^{13}C coupled HSQC NMR of 23 (400 MHz, D_2O)

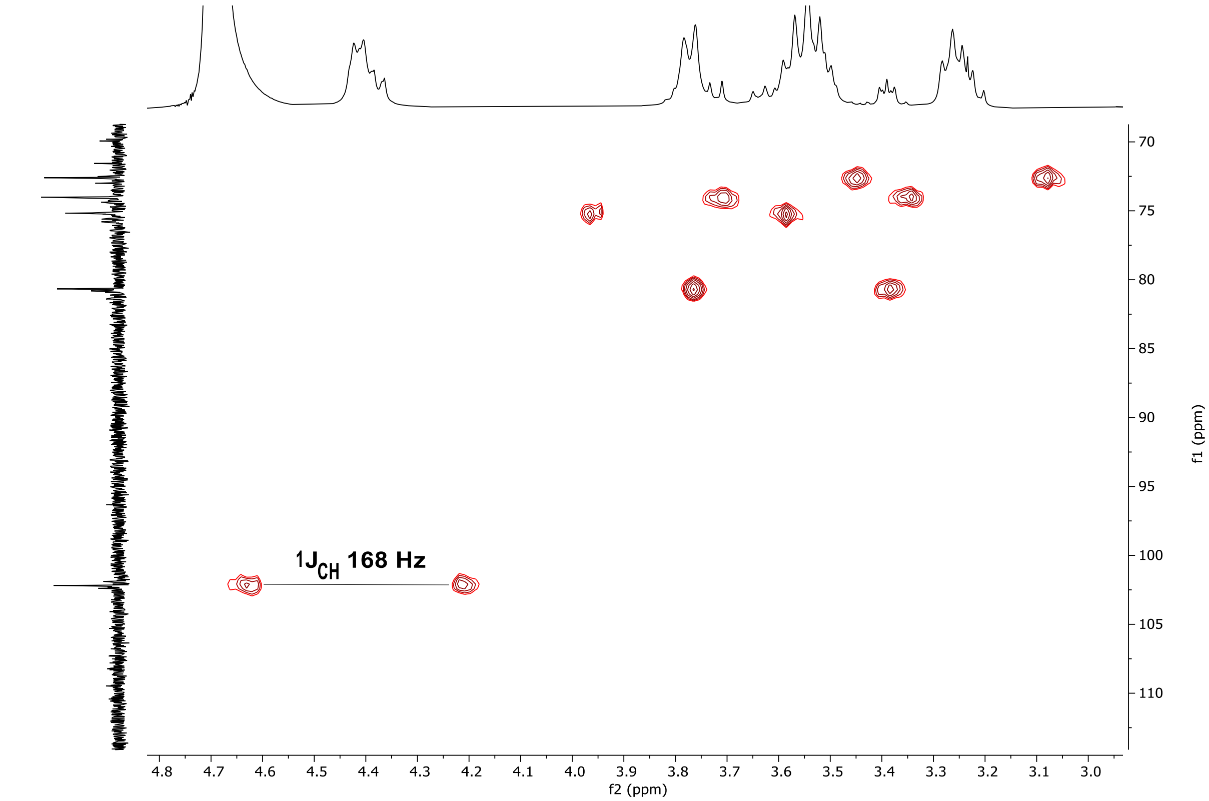

5 Structural Analysis

5.1 General materials and methods for molecular dynamics simulations

All molecular dynamics simulations were performed using Gromacs 5.1.2.[3] The oligosaccharides were modeled using a modified version of GLYCAM06$_{OSMO,r14}$[4] force field and were performed in water using TIP5P[5]as water model. Initial conformations of oligosaccharides were constructed with tleap and the topology was then converted using the python script acpype. A Nosé-Hoover[6] thermostat kept temperature constant at 303 K while a Parrinello-Rahman barostat[7] ensured a constant pressure of 1 bar. Non-bonded interactions were cut-off at 1.4 nm, long range electrostatics were calculated using the particle mesh Ewald method Bonds involving hydrogens were constrained using the LINCS[8] to allow a 2-fs time step; water molecules were kept rigid with SETTLE.[9] The analysis was visualized using OriginPro 2021b. The conformation clustering algorithm GlycanAnalysisPipeline[10] was used to support conformational analysis of oligosaccharides. After energy minimization (steepest descent algorithm) and before the production run, the systems were equilibrated at 300 K for 50 ns in a canonical (NVT) ensemble (constant number of particles, volume and temperature) and subsequently at 300 K and 1 bar for 50 ns in an isothermal-isobaric (NPT) ensemble. The simulation time for modelled polyglucuronic acid oligosaccharide structures **17**, **19**, **21** and **23** was 500 ns, 1000 ns for simulation of **15** and 1500 ns for simulation of **13**.

5.2 General materials and methods for NMR titrations

1H, HSQC, 2D-COSY, 1D TOCSY, 1D NOESY NMR spectra were recorded on a Bruker Biospin AVANCE700 (700 MHz) spectrometer. Samples were prepared by dissolving lyophilized samples in D_2O (concentration ≈ 2 mM) using the solvent as the internal standard in 1H NMR (D_2O: 4.79 ppm 1H). All spectra were recorded at the same temperature i.e. 298 K. Proton resonances of the oligosaccharides were assigned using a combination of ^{1}H, 2D COSY, HSQC, 1D TOCSY and 1D NOESY. Selective 1D TOCSY (pulse program: selmlgp) spectra were recorded using different mixing times to assign all the resonances (d8 = 20, 40, 60, 80 and 120 ms). Selective 1D-NOESY (pulse program: selnogp) spectra were recorded using d8 = 600 ms mixing time. Oligosaccharides were named as follows: D-Glucoronic acid (GlcA) 5-mer **19** and D-GlcA 10-mer **23**. Monosaccharides within a chain were labelled as follows: e.g. A – E in **19**, starting from non-

reducing end (A) to reducing end (E). Protons in a monosaccharide are labelled as follows: e.g. proton attached to C-1 of GlcA monosaccharide A is named “A-1”. Overlapping protons in the oligosaccharide are labelled as follows: e.g. protons attached to C-3 and C-4 of GlcA monosaccharides B, C, D and E are named B−E-3/4. Titration experiments were performed keeping constant concentration for oligosaccharides (2 mM) and TRIS-d11 buffer (10 mM) while $CaCl_2$ concentration was increased gradually (2 mM, 4 mM, 10 mM and 20 mM). For each concentration level of $CaCl_2$ 1H and HSQC-NMR were performed. All spectra were recorded at the same temperature i.e. 298 K.

5.3 **Molecular Dynamics**

5.3.1 Compound **13**

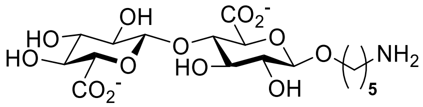

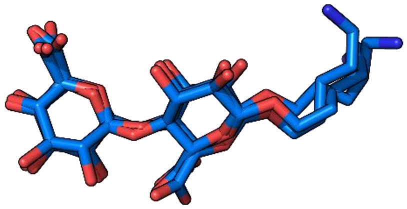

Figure S10. Stacked representative snapshots of **13**.

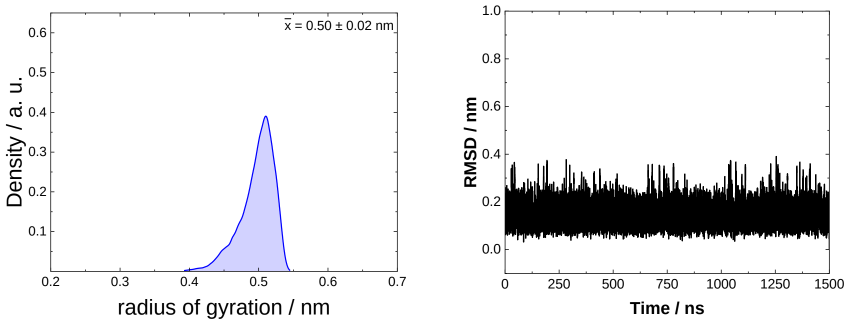

Figure S11. Radius of gyration (RoG) and root-mean-square deviation (RMSD) analysis of **13**.

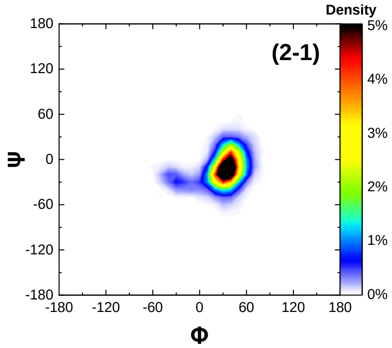

Figure S12. Ramachandran plot of glycosidic linkage of **13**.

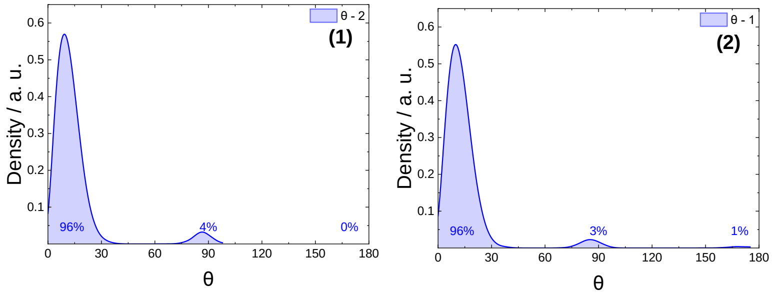

Figure S13 Puckering analysis of **13**. Labelling for glycan pyranose rings from reducing end (1) to non-reducing end (2).

5.3.2 Compound **15**

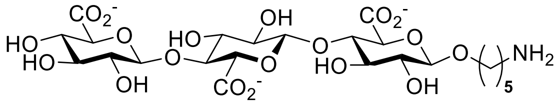

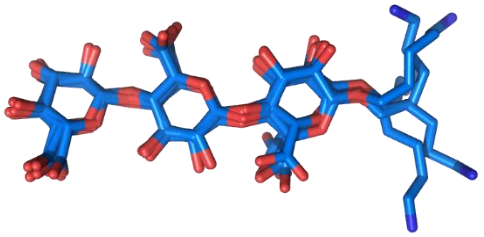

Figure S14 Stacked Representative snapshots of **15**.

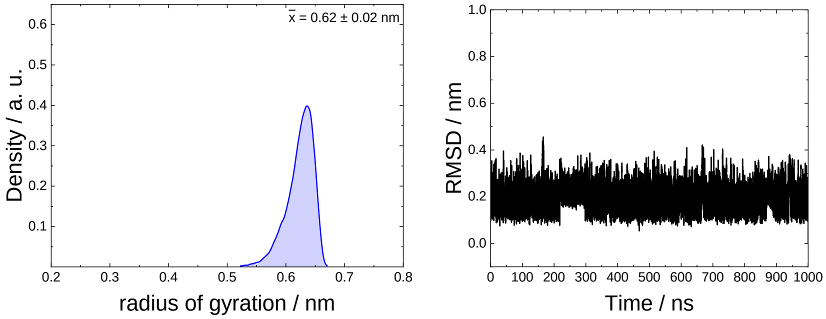

Figure S15. Radius of gyration and root-mean-square deviation (RMSD) analysis of **15**.

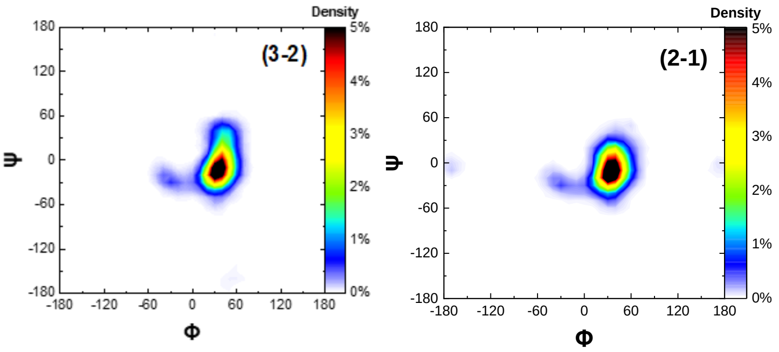

Figure S16. Ramachandran plot of glycosidic linkage of **15**.

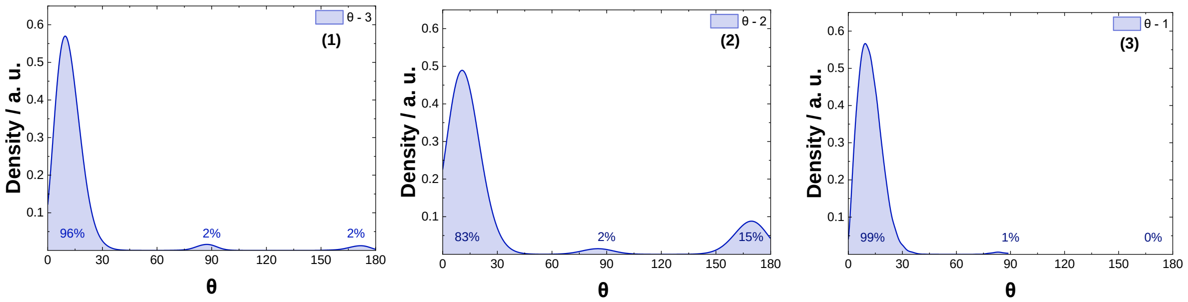

Figure 17: Puckering analysis of **15**. Labelling for glycan pyranose rings from reducing end (1) to non-reducing end (3)

5.3.3 Compound **17**

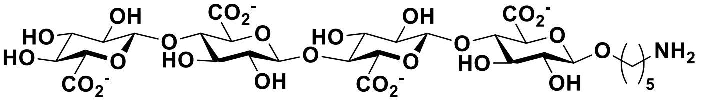

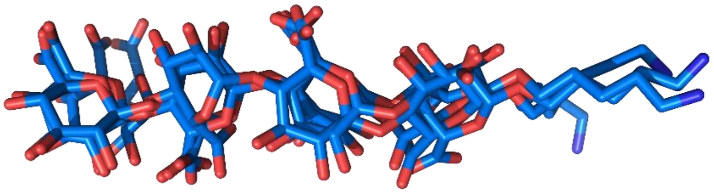

Figure S18. Stacked representative snapshot of **17**.

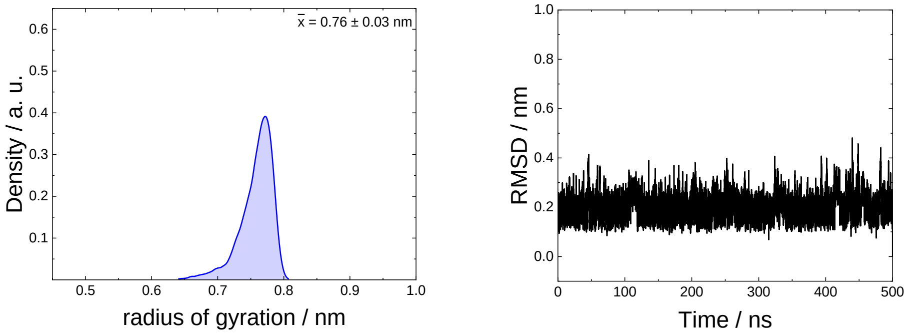

Figure S19. Radius of gyration (RoG) and root-mean-square deviation (RMSD) analysis of **17**.

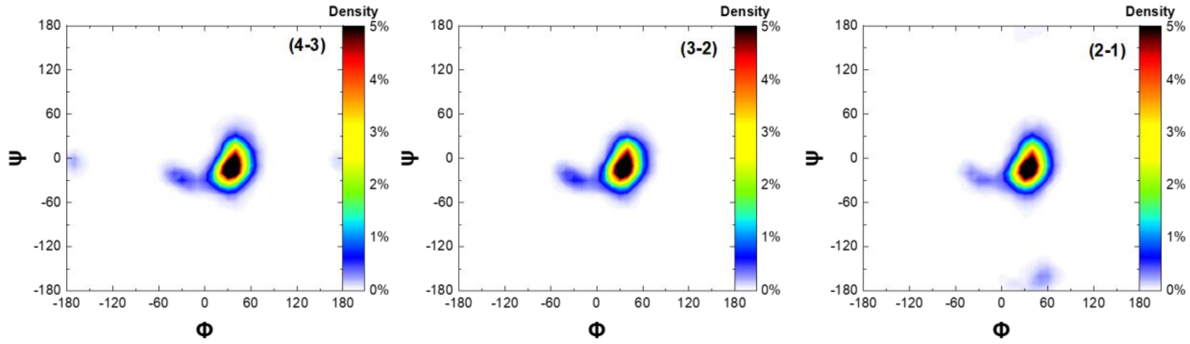

Figure S20. Ramachandran plot of glycosidic linkages of **17**

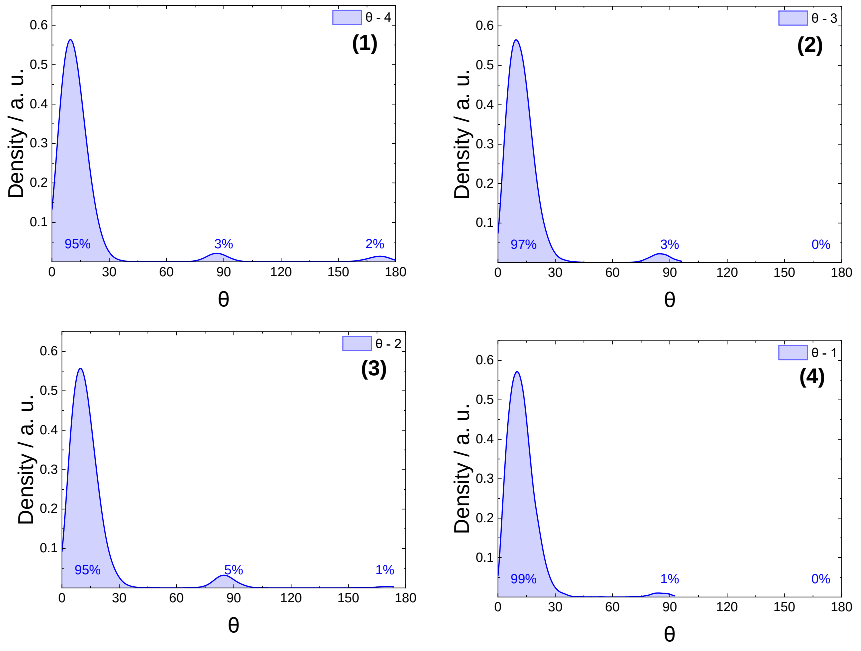

Figure S21. Puckering Analysis of **17**. Labelling for glycan pyranose rings from reducing end (1) to non-reducing end (4).

5.3.4 Compound **19**

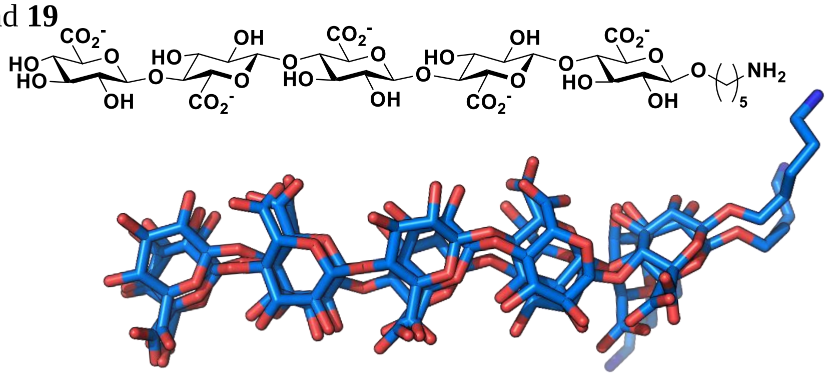

Figure S22. Representative snapshot of **19**.

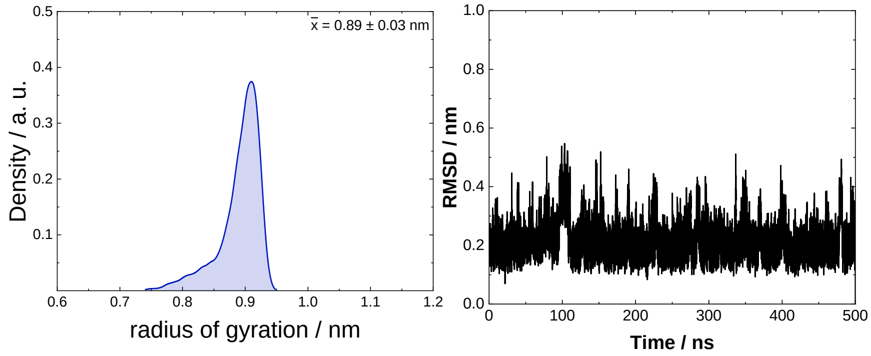

Figure S23. Radius of gyration (RoG) and root-mean-square deviation (RMSD) analysis of **19**.

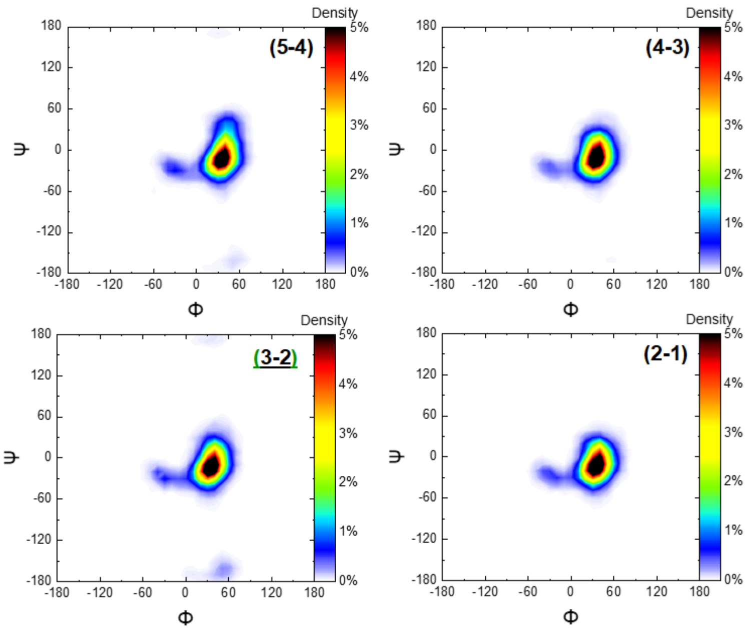

Figure S24. Ramachandran plot of glycosidic linkages of **19**.

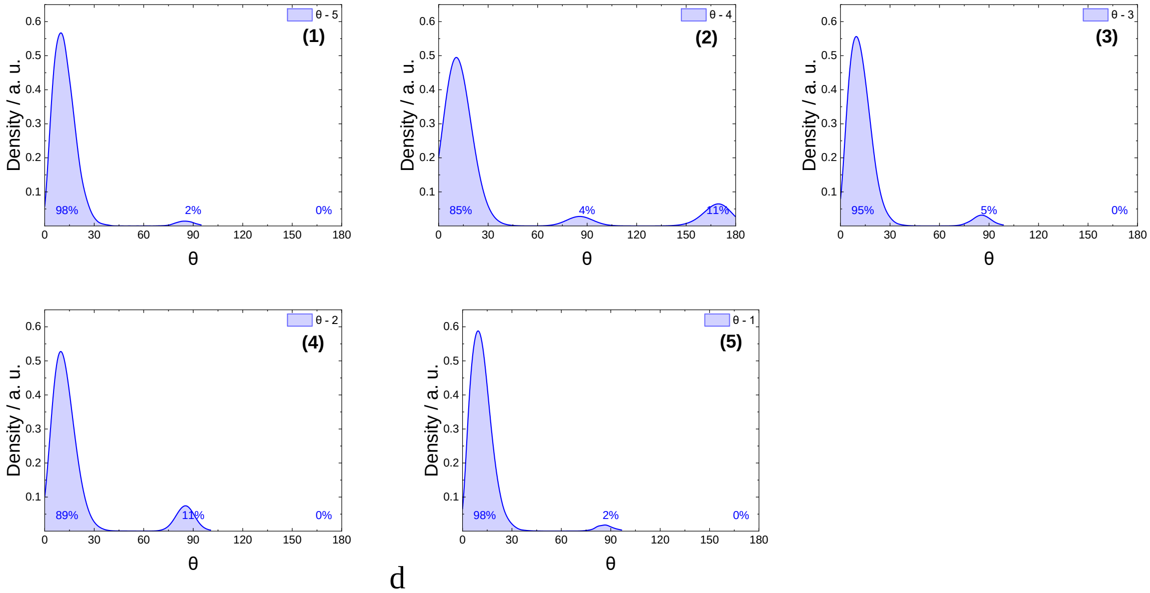

d

Figure S25. Puckering Analysis of **19**. Labelling for glycan pyranose rings from reducing end (1) to non-reducing end (5).

5.3.5 Compound **21**

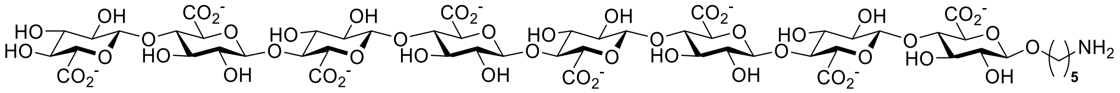

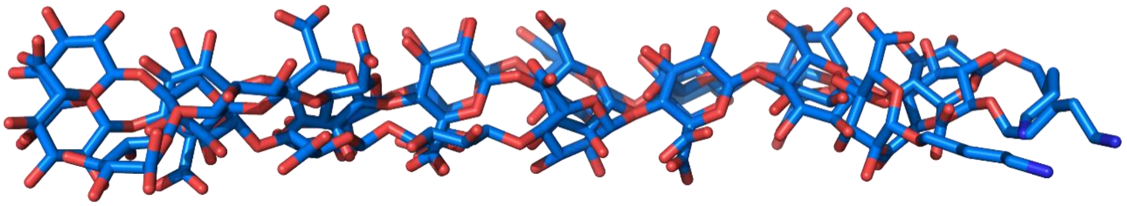

Figure S26. Stacked representative snapshot of **21**

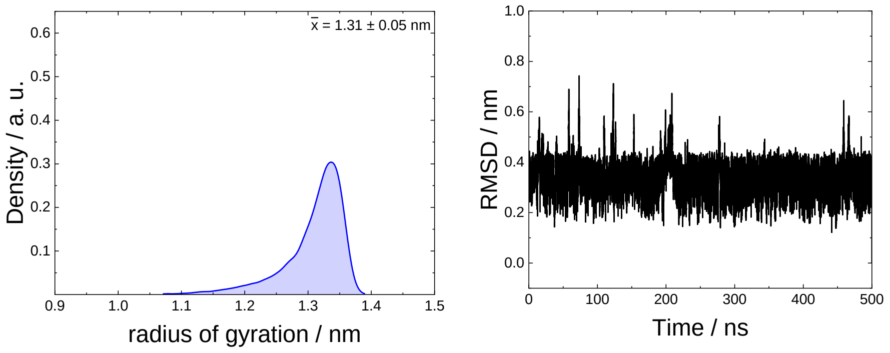

Figure S27. Radius of gyration (RoG) and root-mean-square deviation (RMSD) analysis of **21**.

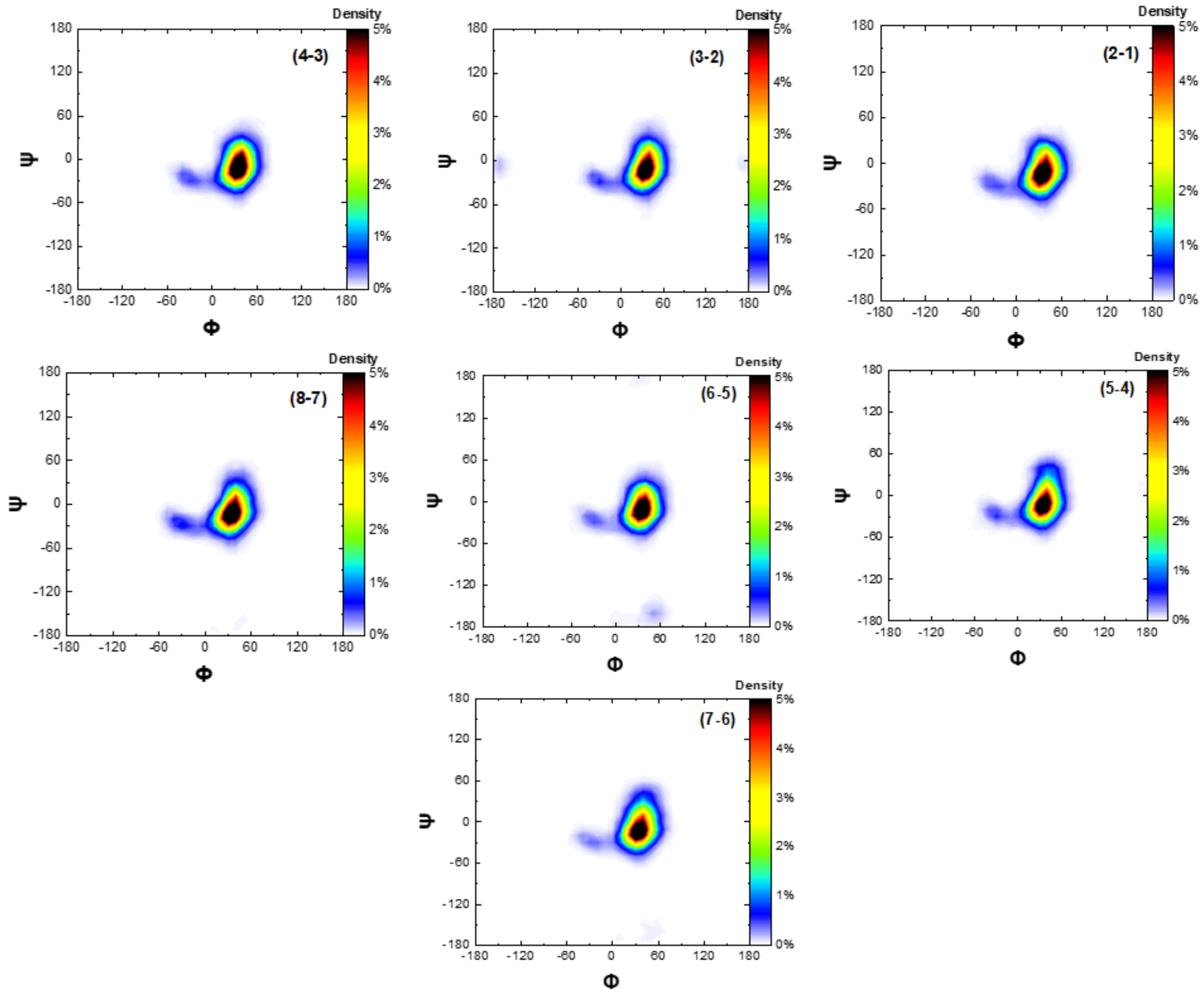

Figure S28. Ramachandran plot of glycosidic linkages of **21**.

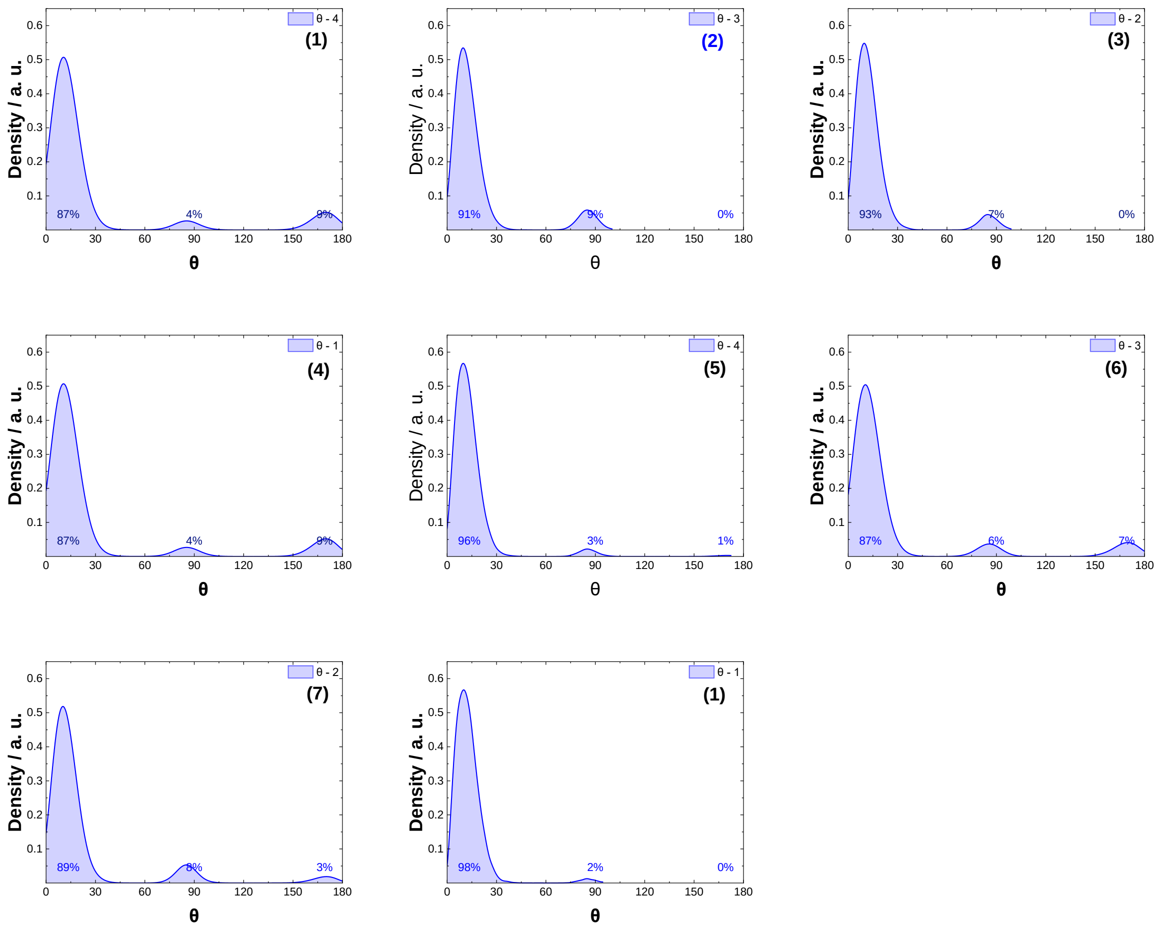

Figure S29. Puckering Analysis of **21**. Labelling for glycan pyranose rings from reducing end (1) to non-reducing end (8).

5.3.6 Compound **23**

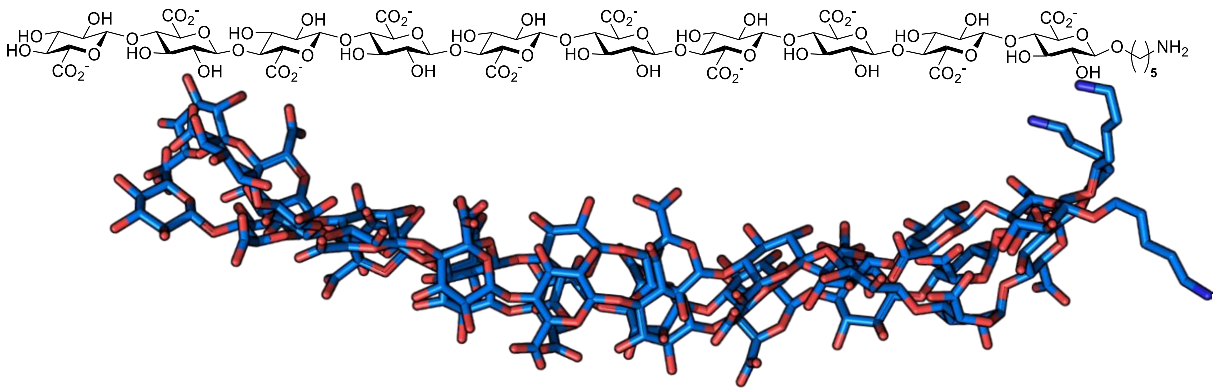

Figure S30. Stacked representative snapshot of **23**

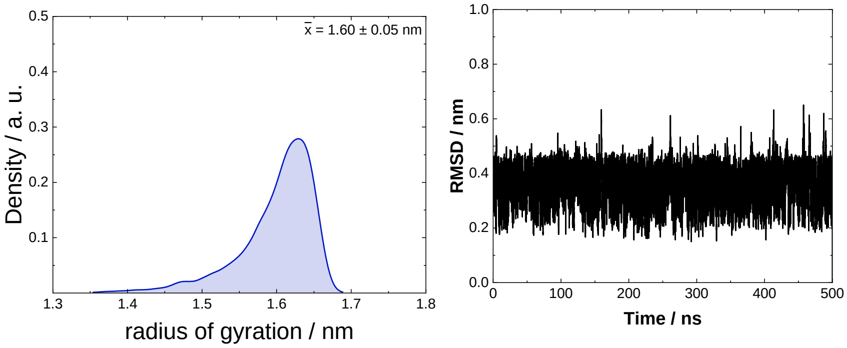

Figure S31. Radius of gyration (RoG) and root-mean-square deviation (RMSD) analysis of **23**.

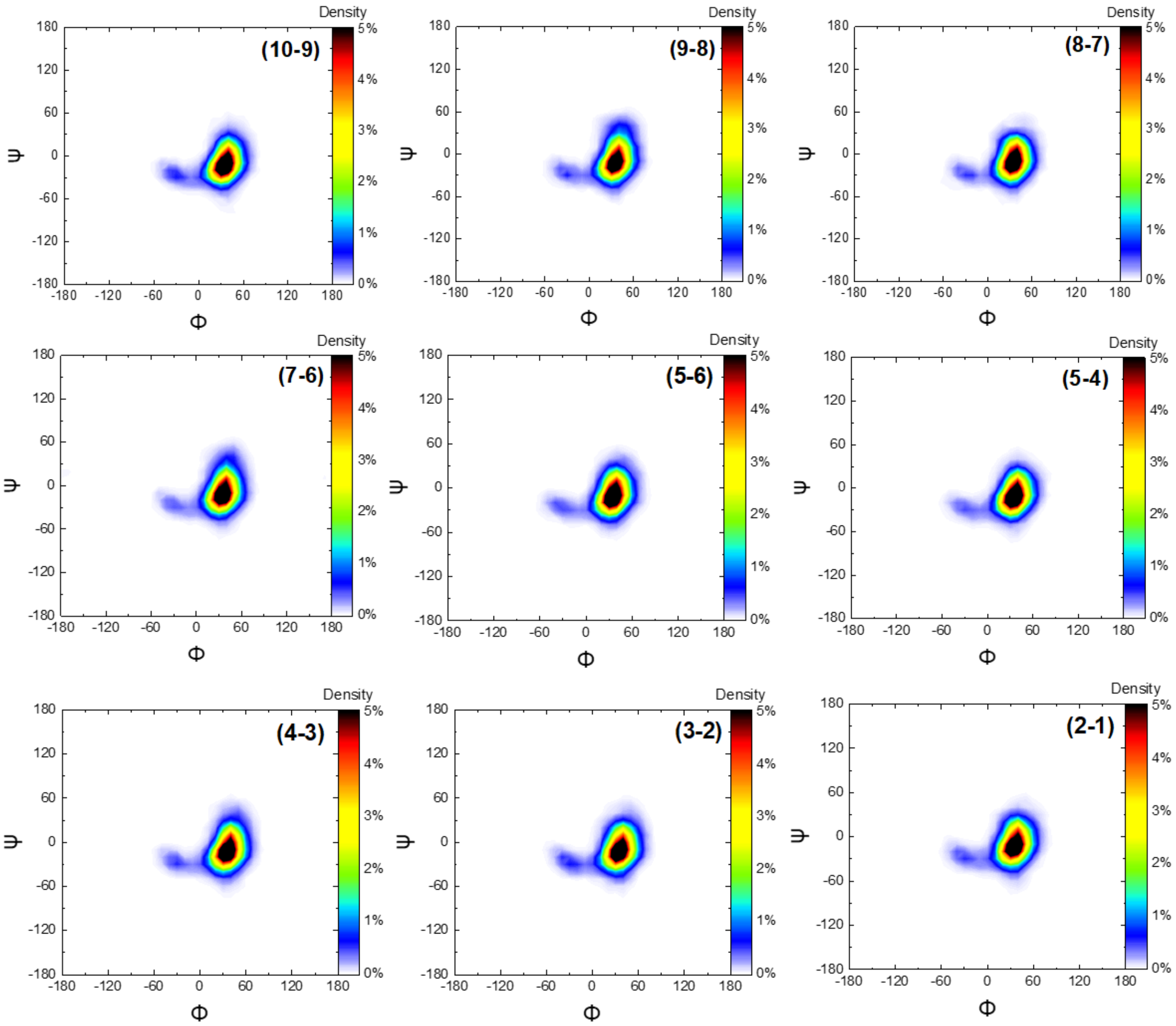

Figure S32. Ramachandran plot of glycosidic linkages of **23**

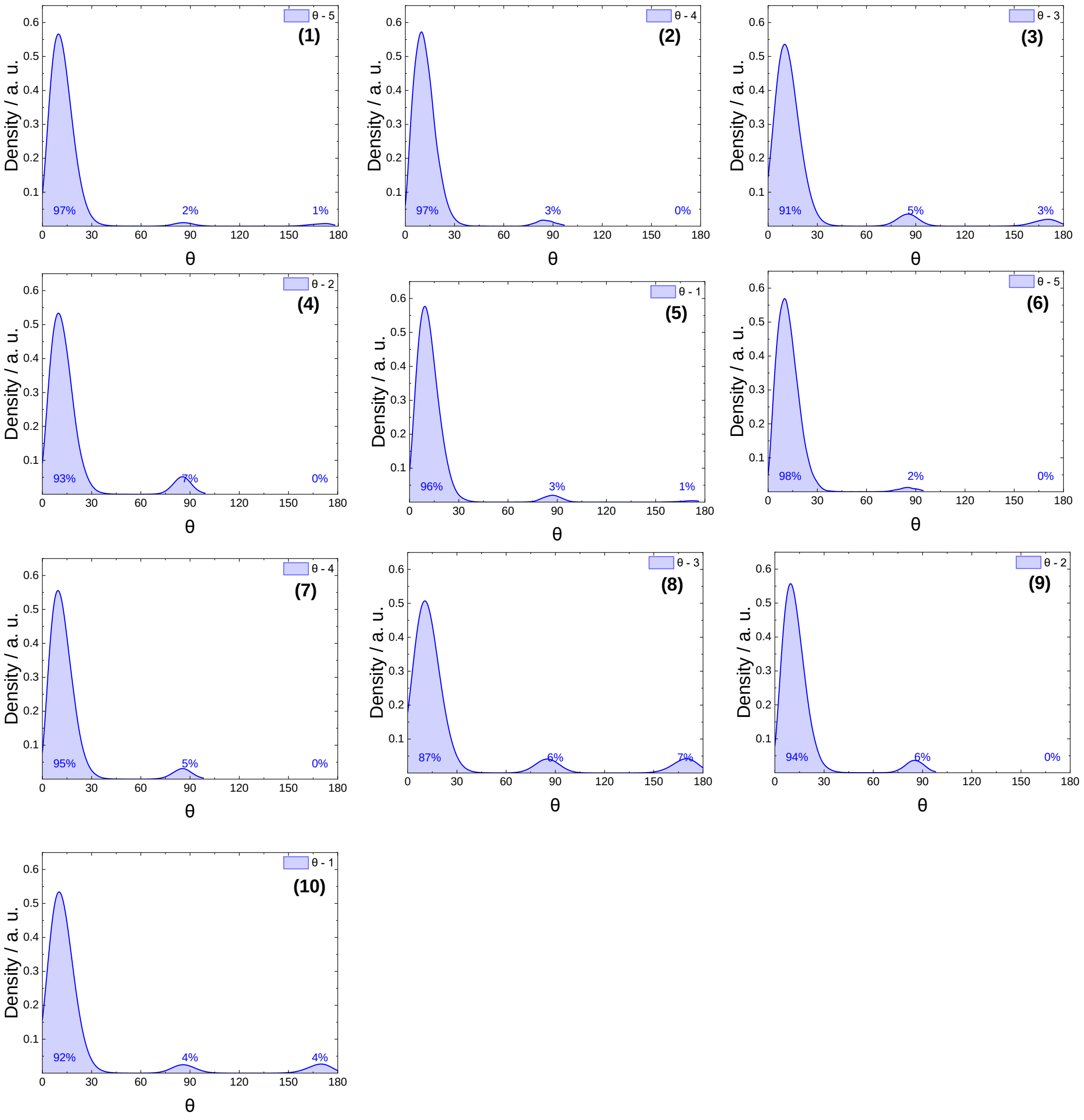

Figure S33. Puckering Analysis of **23**. Labelling for glycan pyranose rings from reducing end (1) to non-reducing end (10).

5.4 **NMR titration study**

5.4.1 NMR characterization and Ca^{2+} titration study of **19**

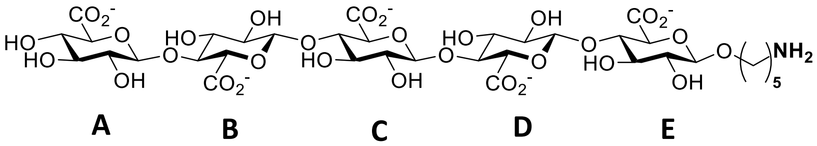

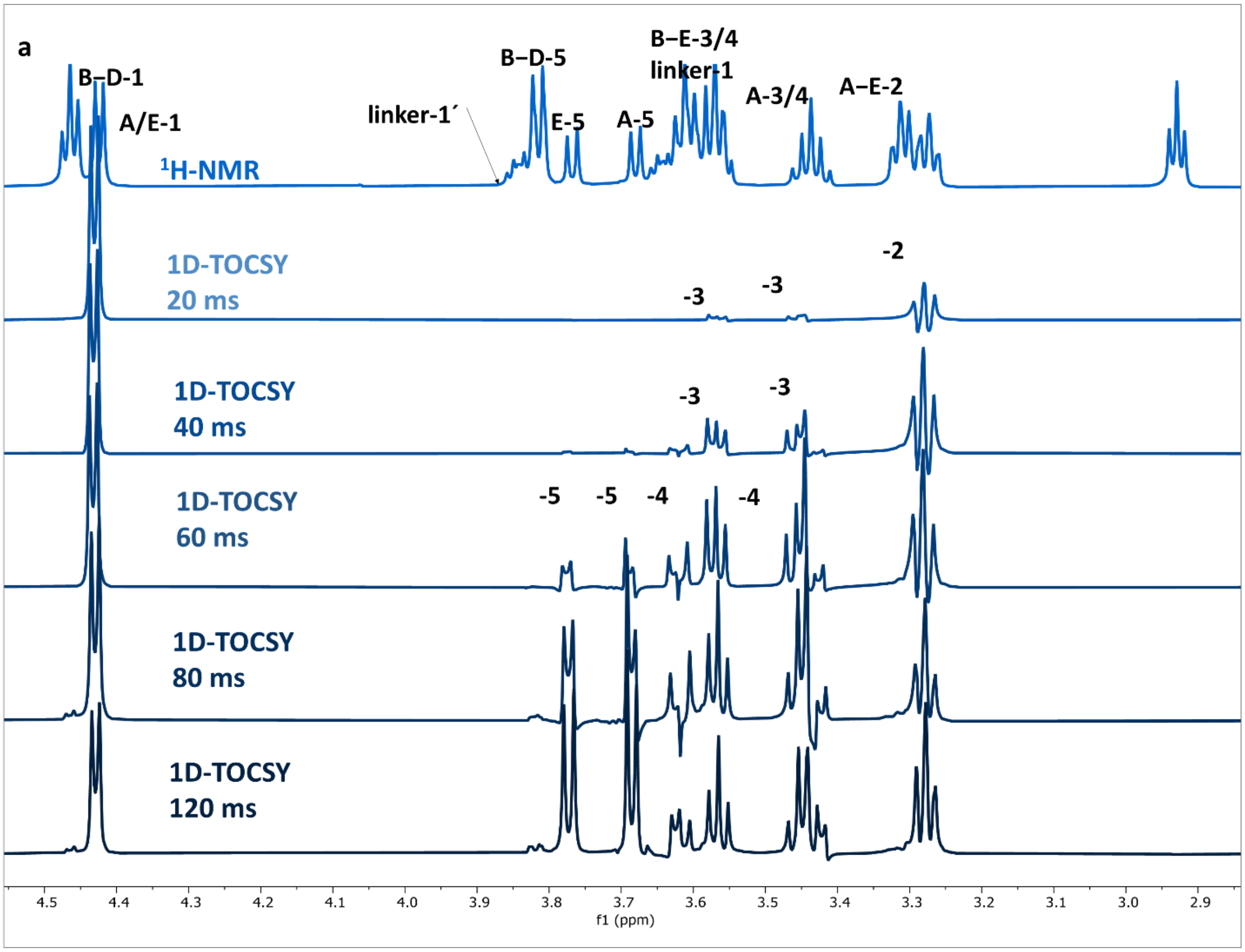

Figure S34. 1D TOCSY (700 MHz, d8 = 20, 40, 60, 80, 120 ms, D_2O) of **19** (A and E) with assignments.

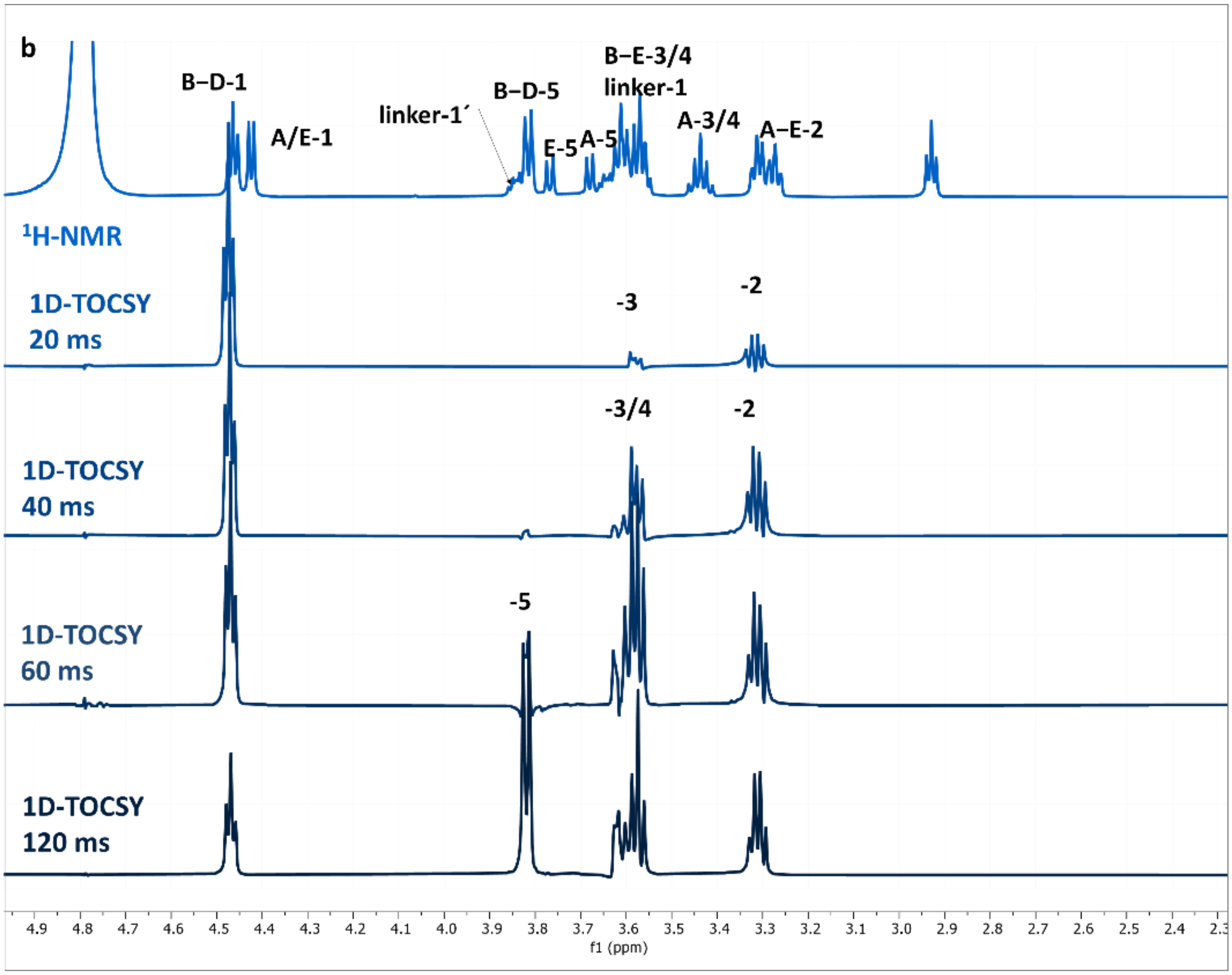

Figure S35. Selective 1D TOCSY (700 MHz, d9 = 20, 40, 60, 120 ms, D_2O) of **19** (B/C/D) with assignments.

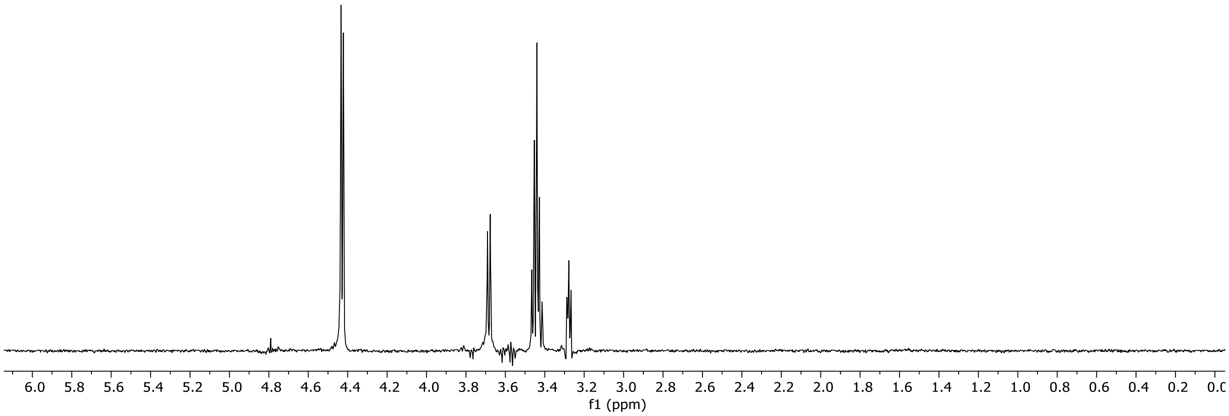

Figure S36. Selective 1D TOCSY (700 MHz, d9 = 120 ms, D_2O) irradiating A-5 (3.62 ppm) of **19.**

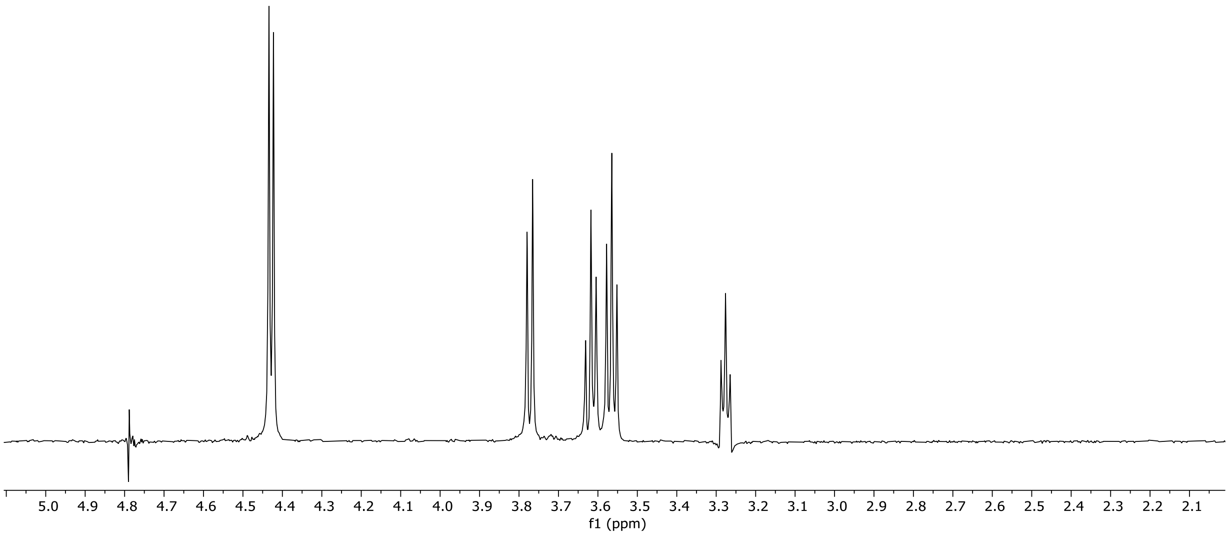

Figure S37. Selective 1D TOCSY (700 MHz, d9 = 120 ms, D_2O) irradiating E-5 (3.70 ppm) of **19.**

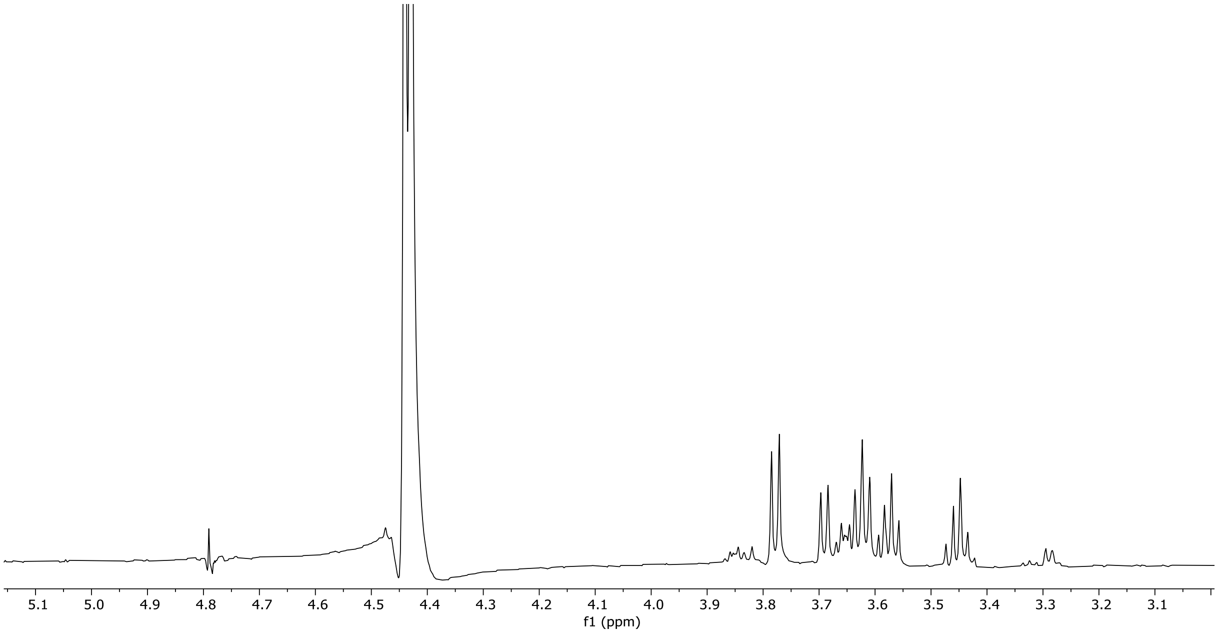

Figure S38. Selective 1D NOESY (700 MHz, d8 = 600 ms, D_2O) irradiating A/1-1 (4.35 ppm) of **19.**

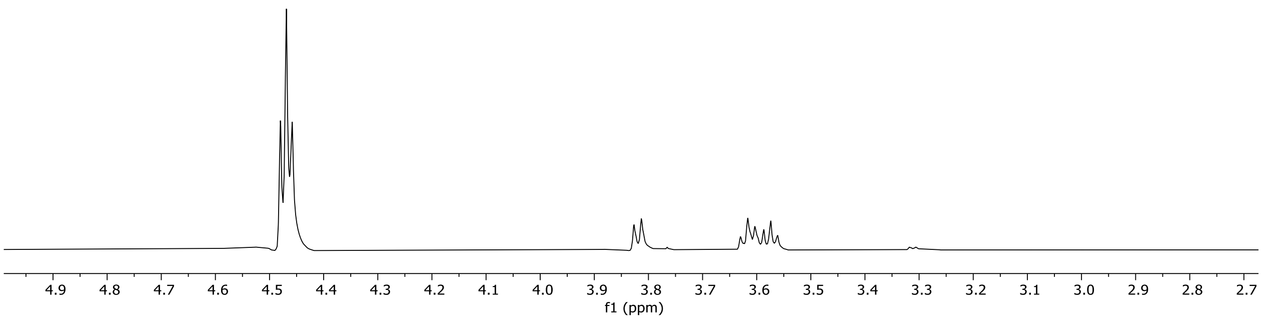

Figure S39. Selective 1D NOESY (700 MHz, d8 = 600 ms, D_2O) irradiating B/C/D-1 (4.41 ppm) of **19**.

**19 TRIS**	**Residue A**	**Residue F**	**Residue B-E**
**δH1**	4.420	4.420	4.461
**δH2**	3.283 – 3.256	3.283 – 3.256	3.321 − 3.285
**δH3**	3.459 – 3.407	3.459 – 3.407	nd
**δH4**	3.459 – 3.407	3.459 – 3.407	nd
**δH5**	3.676	3.764	3.812; 3.808

**19 TRIS $CaCl_2$ (2mM)**	**Residue A**	**Residue F**	**Residue B-E**
**δH1**	0.012; 0.007	0.012; 0.007	nd
**δH2**	nd	nd	nd
**δH3**	0.010	nd	nd
**δH4**	0.010	nd	nd
**δH5**	0.011	0.008	0.010

**19 TRIS $CaCl_2$ (4mM)**	**Residue A**	**Residue F**	**Residue B-E**
**δH1**	0.021; 0.015	0.021; 0.015	nd
**δH2**	nd	nd	nd
**δH3**	0.019	nd	nd
**δH4**	0.019	nd	nd

**δH5**	0.02	0.017	0.018

**19 TRIS $CaCl_2$ (10mM)**	**Residue A**	**Residue F**	**Residue B-E**
**δH1**	0.024	0.024	0.028
**δH2**	nd	nd	nd
**δH3**	0.026	nd	nd
**δH4**	0.026	nd	nd
**δH5**	0.27	0.021	0.025

**19 TRIS $CaCl_2$ (20mM)**	**Residue A**	**Residue F**	**Residue B-E**
**δH1**	0.025	0.025	0.03
**δH2**	nd	nd	nd
**δH3**	0.027	nd	nd
**δH4**	0.027	nd	nd
**δH5**	0.029	0.022	0.029

Table. S9. ΔδH (ppm) extracted out of ^{1}H-NMR of **19** upon titration of $CaCl_2$.

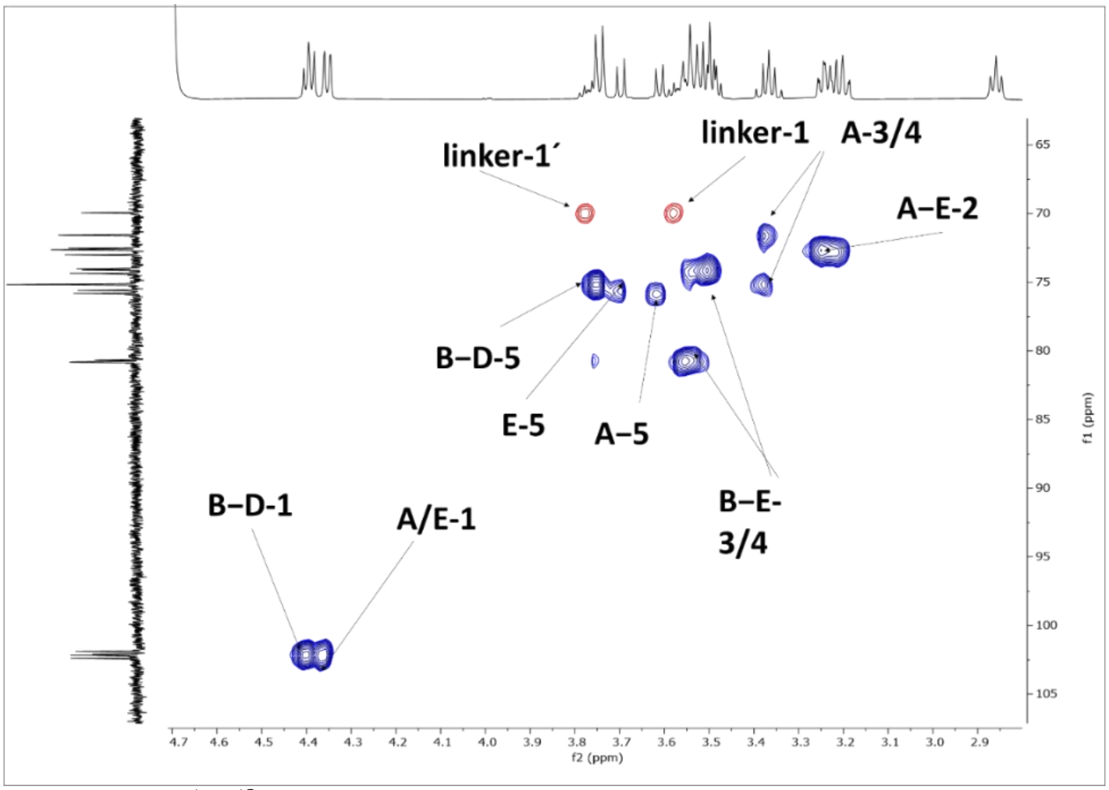

Figure S40. 2D ^{1}H-^{13}C| HSQC (700 MHz, D_2O) of **19** with all peak assignments.

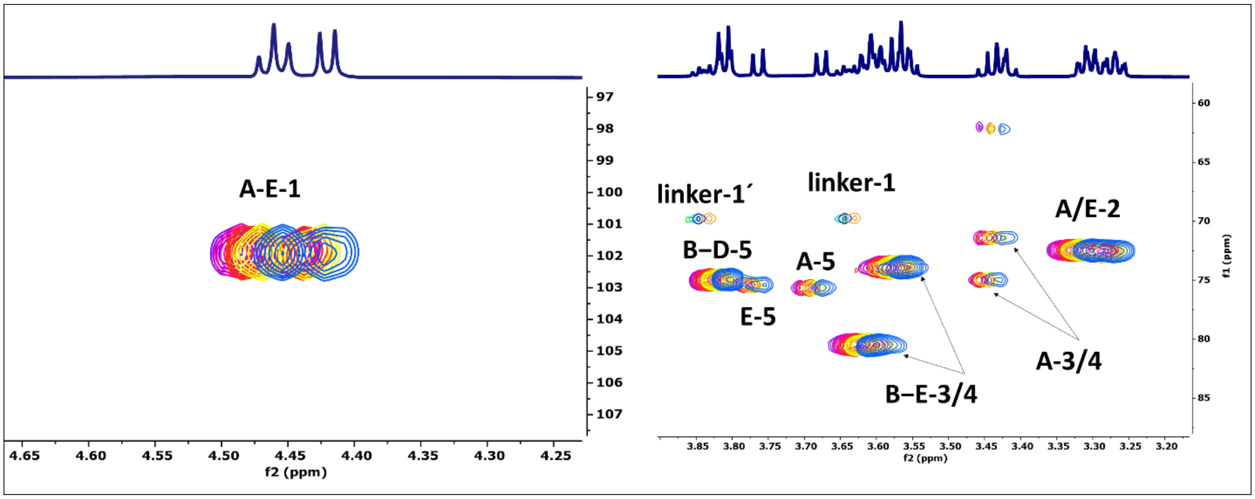

Figure S41. Superimposed ^{1}H-^{13}C HSQC spectra of **19** during Ca^{2+} titration (blue: **19** in TRIS d-11; yellow: 2 mM Ca^{2+}; red: 4 mM Ca^{2+}; pink: 10 mM Ca^{2+}; purple: 20 mM Ca^{2+}).

19 TRIS	Residue A	Residue F	Residue B-E
**ΔδH/C-1**	4.425/102.042	4.425/102.042	4.457/102.042
**ΔδH/C-2**	3.275/72.691	3.275/72.691	3.305/72.330
**ΔδH/C-3**	3.421/71.568<br>3.440/74.954	3.421/71.568<br>3.440/74.938	nd
**ΔδH/C-4**	3.421/71.568<br>3.440/74.954	3.421/71.568<br>3.440/74.938	nd
**ΔδH/C-5**	3.676/75.760	3.762/75.437	3.808/75.115

19 TRIS $CaCl_2$ (2mM)	Residue A	Residue F	Residue B-E
**δH/C-1**	nd/0.029	nd/0.029	nd/0.027
**δH/C-2**	0.009/0.034	0.009/0.034	0.011/0.234
**δH/C-3**	0.014/0.028<br>0.004/0.189	nd	nd
**δH/C-4**	0.014/0.028<br>0.004/0.189	nd	nd
**δH/C-5**	0.010/0.029	0.010/0.029	0.014/0.029

19 TRIS $CaCl_2$ (4mM)	Residue A	Residue F	Residue B-E
**δH/C-1**	nd/0.038	nd/0.038	nd/0.038
**δH/C-2**	0.018/0.044	0.018/0.044	nd/0.243
**δH/C-3**	0.028/0.038<br>0.024/0.199	nd	nd

**δH/C-4**	0.028/0.038<br>0.024/0.199	nd	nd
**δH/C-5**	0.022/0.038	nd	0.022/0.031

**19 TRIS CaCl$_2$ (10mM)**	**Residue A**	**Residue F**	**Residue B-E**
**δH/C-1**	nd/-0.115	nd/-0.115	0.029/0.048
**δH/C-2**	0.015/0.052	0.015/0.052	0.028/0.251
**δH/C-3**	0.032/0.046<br>0.017/0.207	nd	nd
**δH/C-4**	0.032/0.046<br>0.017/0.207	nd	nd
**δH/C-5**	0.023/0.046	0.022/0.046	0.026/0.046

**19 TRIS CaCl$_2$ (10mM)**	**Residue A**	**Residue F**	**Residue B-E**
**δH/C-1**	0.024/0.208	0.024/0.208	0.030/-0.115
**δH/C-2**	0.017/0.052	0.017/0.052	0.030/0.251
**δH/C-3**	0.032/0.046;<br>0.018/0.207	nd	nd
**δH/C-4**	0.032/0.046;<br>0.018/0.207	nd	nd
**δH/C-5**	0.028/0.046	0.023/0.047	0.03/0.046

Table. S10. ΔδH (ppm) extracted out of **19** HSQC-NMR upon titration of $CaCl_2$.

5.4.2 NMR characterization and Ca^{2+} titration study of **23**

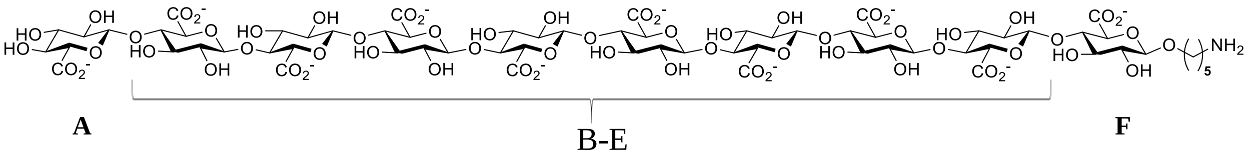

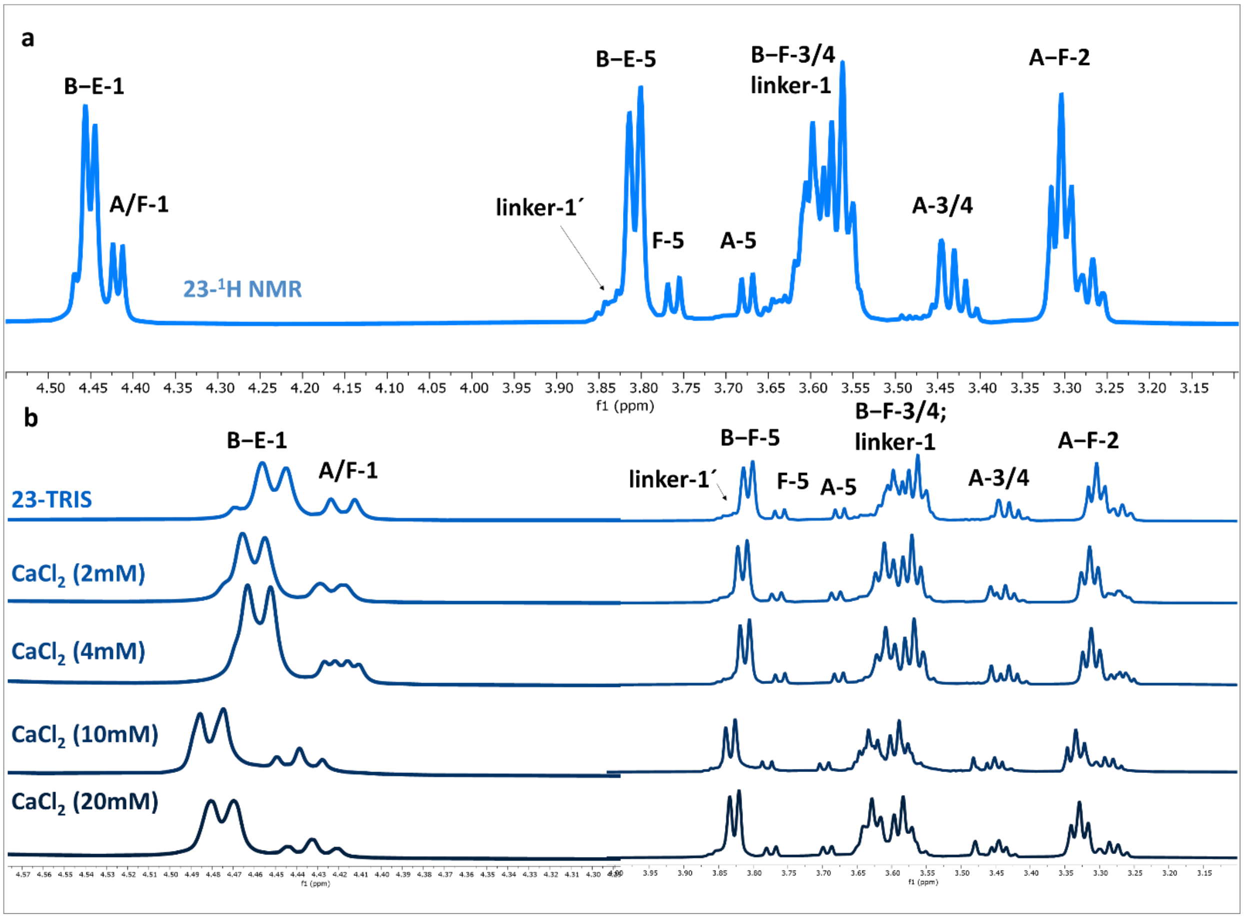

Figure.S42 a) Proton resonance assignment of glycan residues for **23**. b) Titration experiments to evaluate interactions of **23** with Ca^{2+}. The concentration of **23** and Tris d_{11} buffer was kept constant at 2 mM and 10 mM, respectively, while Ca^{2+} concentration was increased in steps of 2, 4, 10, and 20 mM. Two spectral regions of the ^1H NMR spectra are shown, comprising all detectable glycan protons: Left: 4.38-4.51 ppm (anomeric region); Right: 3.90-3.20 ppm.

23 TRIS	Residue A	Residue F	Residue B-E
**δH1**	4.419	4.419	4.456
**δH2**	3.266	3.266	3.304
**δH3**	3.46-3.404	nd	nd
**δH4**	3.46-3.404	nd	nd
**δH5**	3.674	3.761	3.808

23 TRIS $CaCl_2$ (2mM)	Residue A	Residue F	Residue B-E
**δH1**	-	-	-
**δH2**	0.002	nd	0.010
**δH3**	0.002	nd	nd
**δH4**	0.002	nd	nd
**δH5**	0.005	0.005	0.008

23 TRIS $CaCl_2$ (4mM)	Residue A	Residue F	Residue B-E
**δH1**	-	-	-
**δH2**	nd	nd	0.008
**δH3**	0.002	nd	nd
**δH4**	0.002	nd	nd
**δH5**	0.002	-	0.003

23 TRIS $CaCl_2$ (10mM)	Residue A	Residue F	Residue B-E
**δH1**	0.019	0.019	0.025
**δH2**	nd	nd	0.03
**δH3**	0.023	nd	nd
**δH4**	0.023	nd	nd
**δH5**	0.0023	0.018	0.019

23 TRIS $CaCl_2$ (20mM)	Residue A	Residue F	Residue B-E
**δH1**	0.013	0.013	0.019
**δH2**	nd	nd	0.025
**δH3**	0.02	nd	nd
**δH4**	0.020	nd	nd
**δH5**	0.0020	-	0.013

Table. S10. ΔδH (ppm) extracted out of **23** ^{1}H-NMR upon titration of $CaCl_2$.

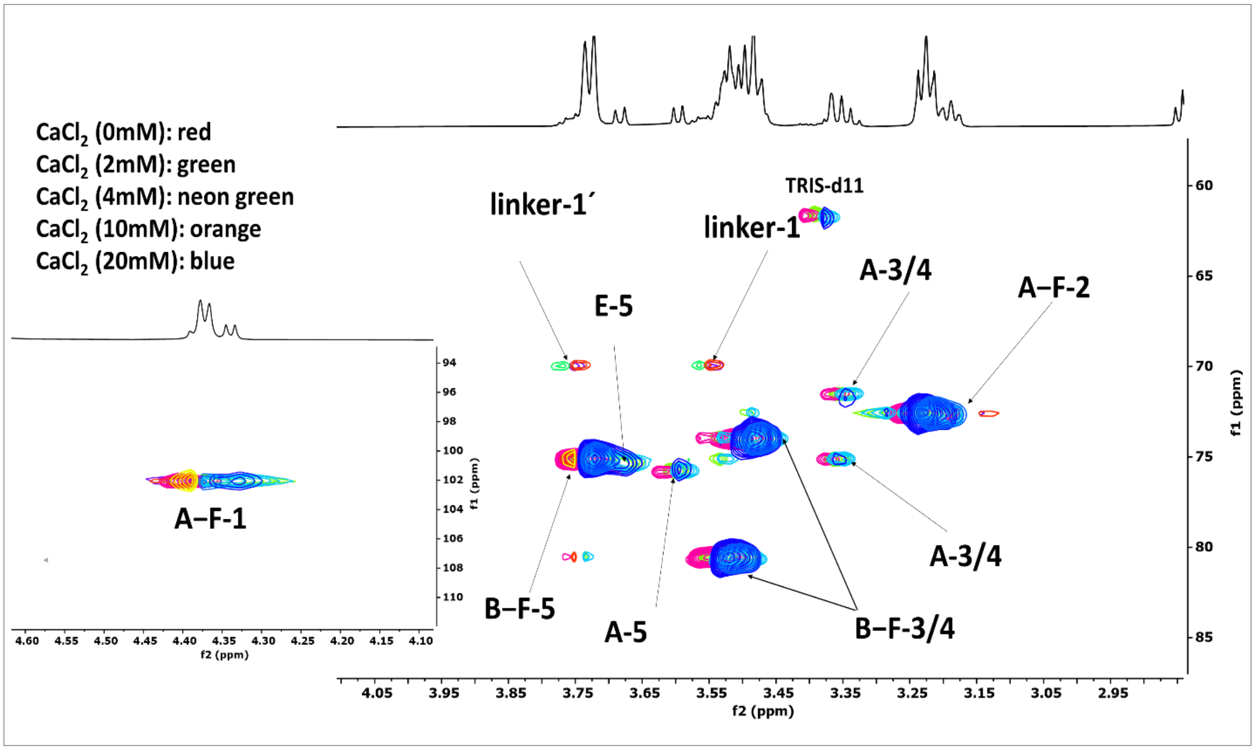

Figure S43. Superimposed ^{1}H-^{13}C HSQC spectra of **23** during Ca^{2+} titration (blue: **23** in TRIS d-11; yellow: 2 mM Ca^{2+}; red: 4 mM Ca^{2+}; pink: 10 mM Ca^{2+}; purple: 20 mM Ca^{2+}).

23 TRIS	Residue A	Residue F	Residue B-E
**ΔδH/C-1**	nd	nd	4.457/102.087
**ΔδH/C-2**	nd	nd	3.302/72.577
**ΔδH/C-3**	3.423/71.605<br>3.423/71.605	nd	nd
**ΔδH/C-4**	3.423/71.605<br>3.434/75.159	nd	nd
**ΔδH/C-5**	3.679/75.800	nd	3.809/75.142

23 TRIS<br>$CaCl_2$ (2mM)	Residue A	Residue F	Residue B-E
**δH/C-1**	nd	nd	0.003/0.003
**δH/C-2**	nd	nd	0.01/0.005
**δH/C-3**	0.008/-0.004<br>0. 004/-0.01	nd	nd
**δH/C-4**	0.008/-0.004<br>0.004/-0.01	nd	nd
**δH/C-5**	0.003/0.006	nd	0.008/0.016

23 TRIS $CaCl_2$ (4mM)	Residue A	Residue F	Residue B-E
**δH/C-1**	nd	nd	0.003/0.003
**δH/C-2**	nd	nd	0.001/-
**δH/C-3**	nd	nd	0.006/-0.003
**δH/C-4**	0.002/0.002<br>0.001/-0.01	nd	nd
**δH/C-5**	0.002/0.002<br>0.001/-0.01	nd	nd

23 TRIS $CaCl_2$ (10mM)	Residue A	Residue F	Residue B-E
**δH/C-1**	nd	nd	0.032/-0.002
**δH/C-2**	nd	nd	0.036/0.004
**δH/C-3**	0.023/-0.01<br>0.28/0.006	nd	nd
**δH/C-4**	0.023/-0.01<br>0.28/0.006	nd	nd
**δH/C-5**	0.017/0.006	nd	0.027/0.019

23 TRIS $CaCl_2$ (10mM)	Residue A	Residue F	Residue B-E
**δH/C-1**	nd	nd	0.024/-0.002
**δH/C-2**	nd	nd	0.031/0.002
**δH/C-3**	0.015/0.003<br>0.019/75.164	nd	nd
**δH/C-4**	0.015/0.003<br>0.019/75.164	nd	nd
**δH/C-5**	0.014/0.006	3.780/75.478	0.018/0.019

Table S11. ΔδH (ppm) extracted out of **23** HSQC-NMR upon titration of $CaCl_2$.

6 References

(1) a) Danglad-Flores J.; Leichnitz, S.; Sletten E. T.; Abragam J. A.; Bienert K.; Le Mai Hoang K.; Seeberger P. H. Microwave-Assisted Automated Glycan Assembly. *J. Am. Chem. Soc*. **2021**, *143* (23), 8893-8901. b) Pardo-Vargas A.; Delbianco M.; Seeberger P. H. Automated Glycan Assembly as an Enabling Technology. *Curr. Opin. Chem. Biol*. **2018**, *46*, 48-55.

(2) a) Le Mai Hoang K.; Pardo-Vargas A.; Zhu, Y.; Yu Y.; Loria M.; Delbianco, M.; Seeberger P. H. Traceless Photolabile Linker Expedites the Chemical Synthesis of Complex Oligosaccharides by Automated Glycan Assembly. *J. Am. Chem. Soc.* **2019**, *141*, (22), 9079-9086. b) Delbianco M.; Kononov A.; Poveda A.; Yu Y.; Diercks T.; Jiménez-Barbero J.; Seeberger P. H. Well-Defined Oligo- and Polysaccharides as Ideal Probes for Structural Studies. *J. Am. Chem. Soc.* **2018**, *140* (16), 5421-5426 c) Fosso M.; Findee M. N.; Zhang Q.; Nziko V. D. P. N.; Kawasaki Y.; Shrestha S. K.; Bearss J.; Gregory R.; Takemoto J. Y.; Chang C. W. T. Structure-Activity Relationships for Antibacterial to Antifungal Conversion of Kanamycin to Amphiphilic Analogues. *J. Org. Chem*. **2015**, *80* (9), 4398-4411. d) Johannes M.; Reindl M.; Gerlitzki B.; Schmitt E.; Hoffmann-Röder A. Synthesis and Biological Evaluation of a Novel MUC1 Glycopeptide Conjugate Vaccine Candidate Comprising a 4'-Deoxy-4'-Fluoro-Thomsen-Friedenreich Epitope. Beilstein *J. Org. Chem.* **2015**, *11*, 155-161. e) Kröck L.; Esposito D.; Castagner B.; Wang C. C.; Bindschädler, P.; Seeberger P. H. Streamlined Access to Conjugation-Ready Glycans by Automated Synthesis. *Chem. Sci*. **2012**, *3*, 1617-1622. f) Tyrikos-Ergas T.; Bordoni V.; Fittolani G.; Chaube M. A.; Grafmüller A.; Seeberger P. H.; Delbianco M. Systematic Structural Characterization of Chitooligosaccharides Enabled by Automated Glycan Assembly. *Chem. A Eur. J.* **2021**, *27*, 2321-2325.

(3) Spoel, D. V. D.; Lindahl, E.; Hess, B.; Groenhof, G.; Mark, A. E.; Berendsen H. J. C.; Gromacs: Fast, Flexible, and Free. *Journal of Computational Chemistry* **2005**, *26* (16), 1701-1718.

(4) Karl N. K.; Austin B. Y.; Sarah M.; Jorge G.; Charlisa R. D.; Lachele F. B.; Woods R. J.; GLYCAM06: A generalizable biomolecular force field. Carbohydrates. *Journal of Computational Chemistry*, **2007**, *29* (4), 622-655.

(5)Mahoney, M. W.; Jorgensen, W. L.; A Five-site Model for Liquid Water and Reproduction of the Density Anomaly by Rigid, Nonpolarizable Potential Functions. *J. Chem. Phys.* **2000**, *112*, 8910–8922.

(6) Hoover W. G.; Canonical Dynamics: Equilibrium Phase-space Distributions. *Phys. Rev. A 31* **1985**, 1695-1697**.**

(7) Parrinello M.; Rahman A.; Crystal Structure and Pair Potentials: A Molecular-Dynamics Study. *Phys. Rev. Lett.* **1980,** *45*, 1196-1199.

(8) Hess B.; Bekker H.; Berendsen H. J. C.; Fraaije J. G. E. M. LINCS: A Linear Constraint Solver for Molecular Simulations. *Journal of Computational Chemistry* **1998**, *18* (12) 1463-1472.

(9) Miyamoto S.; Kollman P. A. Settle: An Analytical Version of the SHAKE and RATTLE Algorithm for Rigid Water Models. *Journal of Computational Chemistry* **1992**, *13* (8), 952-962.

(10) Ives C. M.; Singh O.; D'Andrea S.; Fogarty C. A.; Harbison A. M.; Satheesan A.; Tropea B.; Fadda E. Restoring Protein Glycosylation with GlycoShape. *Nat Methods* **2024**, *21*, 2117-2127.
